# Characteristic central nervous system fingerprints and related molecular signatures in difficult-to-treat rheumatoid arthritis

**DOI:** 10.1186/s12974-026-03992-z

**Published:** 2026-08-03

**Authors:** Lilla Gunkl-Tóth, Gergely Orsi, Noémi Császár-Nagy, Lili Duzsik, Gábor Mátay, Krisztina Csókási, Gábor Sütő, Gábor Kumánovics, Szabolcs Takács, Zoltán Vidnyánszky, József Kun, Krisztina Takács-Lovász, Anett Pintér, Panna Királyhidi, Georg Schett, György Nagy, Zsuzsanna Helyes

**Affiliations:** 1 Department of Rheumatology and Immunology, Semmelweis University, Budapest, Hungary; 2 Department of Pharmacology and Pharmacotherapy, Medical School, University of Pécs, Pécs, Hungary; 3 HUN-REN–PTE Chronic Pain Research Group, Pécs, Hungary; 4 HUN-REN-PTE Clinical Neuroscience MR Research Group, Pécs, Hungary; 5 Department of Neurology, Medical School, University of Pécs, Pécs, Hungary; 6 Psychosomatic Outpatient Clinic, Budapest, Hungary; 7 Ludovika University of Public Service, Budapest, Hungary; 8 Institute of Psychology, Faculty of Humanities and Social Sciences, University of Pécs, Pécs, Hungary; 9 Second Department of Medicine and Nephrology-Diabetes Centre, University of Pécs, Pécs, Hungary; 10 Department of Rheumatology, Immunology and Musculoskeletal Rehabilitation, Medical School, University of Pécs, Pécs, Hungary; 11 Institute of Psychology, Department of General Psychology and Methodology Faculty of Humanities and Social Sciences, Károli Gáspár University of the Reformed Church in Hungary, Budapest, Hungary; 12 Brain Imaging Centre, HUN-REN Research Centre for Natural Sciences, Budapest, Hungary; 13 Hungarian Centre for Genomics and Bioinformatics, Szentágothai Research Centre, University of Pécs, Pécs, Hungary; 14 Department of Genetics, Cell and Immunobiology, Semmelweis University, Budapest, Hungary; 15 Department of Internal Medicine 3 – Rheumatology and Clinical Immunology, Friedrich-Alexander-University (FAU) Erlangen-Nürnberg and Universitätsklinikum Erlangen, Erlangen, Germany; 16 Heart and Vascular Centre, Semmelweis University, Budapest, Hungary; 17 National Laboratory for Drug Research and Development, Budapest, Hungary

**Keywords:** Difficult-to-treat rheumatoid arthritis, Pain, Central nervous system, Neuroimaging, Psychology

## Abstract

**Background:**

Despite guideline-recommended treatment, approximately 30% of rheumatoid arthritis (RA) patients remain symptomatic and are classified as difficult-to-treat (D2T), representing an unmet clinical need. D2T RA is a heterogeneous state potentially involving differences across several biological and psychological levels, but the exact underlying mechanisms remain unclear. To capture this complexity, our multimodal cross-sectional study aimed to characterize the contributing factors of the D2T state with a multimodal approach.

**Methods:**

31 D2T RA (defined by EULAR, 2021), 18 non-D2T RA patients, and 32 healthy controls (HCs) were included. All participants underwent a clinical assessment, psychological analysis (in-depth interviews, the Rorschach test, and questionnaires), two resting-state functional MRI (fMRI) scans with standardized heat pain stimulation in between, and peripheral blood transcriptomic analysis.

**Results:**

The activity and connectivity of brain regions, especially the somatosensory cortex and the posterior cingulate cortex, one of the main hubs of the default mode network (DMN), showed significant alterations in D2T RA. Furthermore, acute pain stimulation influenced connectivity patterns of D2T patients differently compared to non-D2T patients and HCs. These results are in line with the psychological profiles of D2T patients characterized by cognitive, coping, and emotional dysregulations as well as altered pain processing. Transcriptomic analysis identified mediators, pathways, and signaling mechanisms such as neuregulin 1 (NRG1) and type I interferon signaling associated with neuroinflammation and synaptic plasticity.

**Conclusion:**

Our findings identify central nervous system (CNS) alterations as a meaningful contributor to the D2T RA phenotype, helping to explain persistent symptom burden beyond inflammation. These characteristic neuroimmune signatures support multidisciplinary, personalized interventions that target CNS processes alongside inflammation control to improve outcomes.

**Supplementary Information:**

The online version contains supplementary material available at https://doi.org/10.1186/s12974-026-03992-z.

## Background

Rheumatoid arthritis (RA) is a systemic, autoimmune disease affecting up to 1% of the population [1]. Targeted anti-inflammatory and immune-modulating therapies have provided a breakthrough in the treatment of RA; however, symptoms persist in up to ~ 27.5% of patients, even with state-of-the-art anti-inflammatory therapy [2]. This „difficult-to-treat” (D2T) RA state, as defined by the European Alliance of Associations for Rheumatology (EULAR) [3–5], is characterized by symptom persistence despite guideline-recommended treatment, which represents a substantial unmet medical need. Though D2T RA refers to a heterogeneous population [6] with factors such as treatment non-compliance, comorbidities, fibromyalgia, and health-care related issues contributing, the underlying mechanisms are not fully understood. Central nervous system (CNS) factors, however, may be particularly relevant in relation to sustained pain, and could therefore play an important role in the background of symptom persistence [2–4, 7, 8].

Complex bidirectional crosstalk between chronic joint inflammation and the brain, referred as „joint-brain axis” is considered to contribute to symptoms in RA [9]. Autoantibodies and pro-inflammatory cytokines produced by peripheral immune cells and activated microglia in the spinal dorsal horn and other pain-related brain regions may lead to sustained neuroinflammation and consequent central sensitization in arthritis [10–13]. CNS changes associated with psychosocial distress, maladaptive coping mechanisms, cognitive malfunctions, or mood disorders are likely to contribute to D2T development, but these processes have not been thoroughly investigated [14–16]. The Rorschach inkblot test [17] is a performance-based psychological assessment in which responses to ambiguous visual stimuli are evaluated to characterize aspects of personality functioning, affective processing and cognitive organization, which, although debated, has recently regained scientific interest [18]. Neuroimaging studies indicate that its complex and ambiguous stimuli activate multiple brain networks, including those involved in vision, attention, emotion, and social cognition, supporting its value for exploring implicit cognitive-emotional processes beyond self-report tools [18, 19].

Functional magnetic resonance imaging (fMRI) revealed changes in brain activation patterns in RA related to pain and inflammation [20–24] as well as in response to treatment [25, 26]. Furthermore, it has also been demonstrated that CNS alterations are reflected in the peripheral blood [27–35]. Unbiased transcriptomics of peripheral blood mononuclear cells (PBMC) proved to be a valuable tool for determining neuroinflammatory pathways involved in CNS diseases such as migraine [27].

To explore the nature of the D2T state in RA, we performed an integrative, multimodal characterization of patients by determining (I) activity and connectivity changes with fMRI baseline and after acute painful stimulation, (II) bulk RNA sequencing (RNA-seq) analysis of PBMCs, and (III) psychodiagnostic profiling, including questionnaires, the Rorschach projective test, and in-depth interviews. Furthermore, potential molecular mechanisms of therapy resistance and links between persistent inflammation and pain were investigated to identify novel prognostic biomarkers and drug targets for D2T RA.

## Methods

### Participant selection

**Fig. 1:**
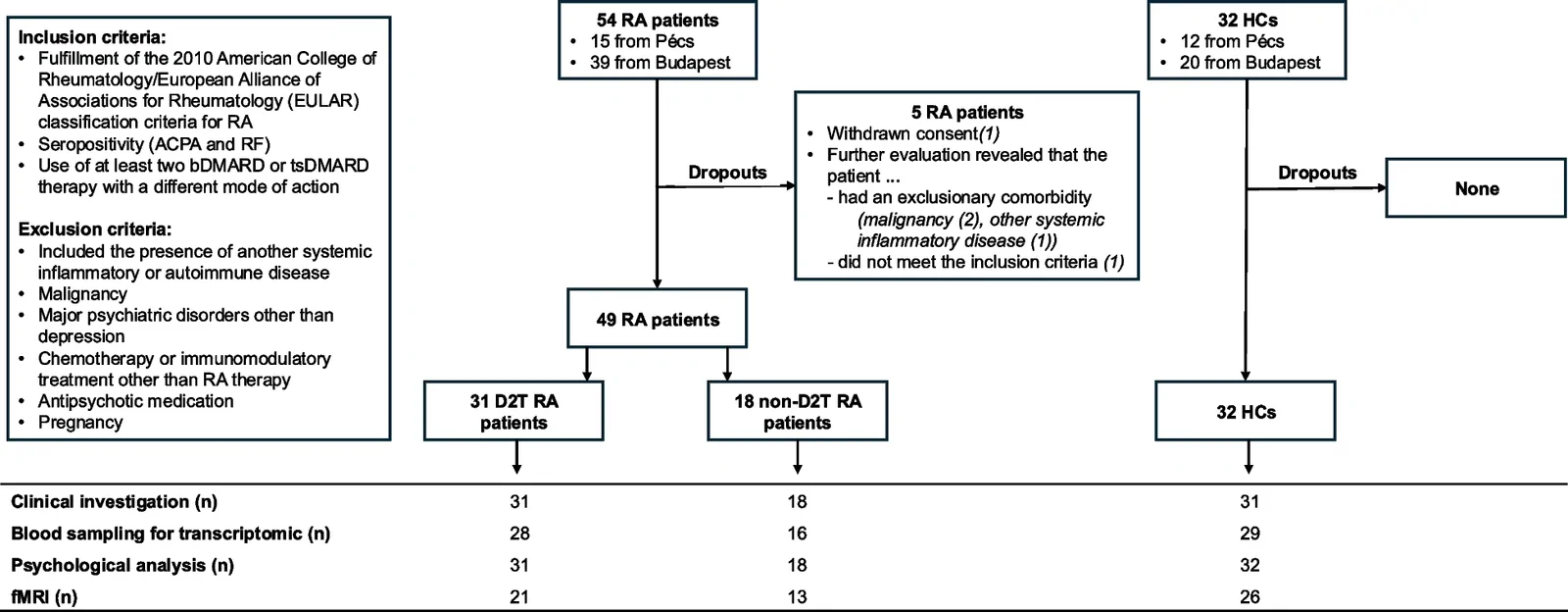
Summary of patient inclusion. The reasons for dropping out were the following: a) failure to attend one of the examination sessions due to personal reasons and lack of cooperation in rescheduling (fMRI, clinical examination, blood sampling) b) the blood sample was inadequate (clotted sample or non-fasting at the time of sampling) c) in case of fMRI additional consent was needed which was declined due to personal reasons, or contraindications (metal implantation) or examinations could not be conducted due to claustrophobia. *fMRI: functional magnetic resonance imaging, HC: healthy controls, RA: rheumatoid arthritis*

RA patients (both male and female) were identified from databases at the University of Pécs and the Hospital of the Hospitaller Brothers, Budapest. After pre-screening, eligible patients were contacted, gave written consent, and underwent clinical, psychological, blood, and fMRI assessments within 3 weeks. The participants had 3 separate visits during this time period: one visit for the clinical examination and blood collection, one visit for the fMRI investigation, and one separate visit for the psychological evaluation. Patients were classified as D2T RA (*n* = 31) or non-D2T RA (*n* = 18) based on EULAR D2T RA definition [4]. D2T patients were further divided into subgroups: high inflammation–high pain (*n* = 10), high inflammation–low pain (*n* = 11), and low inflammation–high pain (*n* = 10). High inflammation was defined as C-reactive protein (CRP) > 5 mg/L and/or erythrocyte sedimentation rate (ESR) > 25 mm/hr; high pain as visual analogue scale (VAS) > 50 mm (past 4 weeks). The non-D2T group had ≥ 2 prior biological/targeted synthetic disease-modifying antirheumatic drugs (b/tsDMARDs) and similar disease duration, but did not fulfill D2T criteria based on their overall clinical status and well-being. Healthy controls (HCs, *n* = 32) were age- and sex-matched. The study complied with the Declaration of Helsinki and was approved by the Hungarian National Ethics Committee (ETT-TUKEB; BMEÜ/3788–1/2022/EKU). Demographic characteristics including age, sex, and disease duration were comparable between sites, and all assessments (fMRI, transcriptomics, psychology) were performed using identical protocols regardless of recruitment site, which limits protocol-driven confounding. (Fig. 1, Suppl. Mat.1).

### Procedures and data analysis

**Fig. 2:**
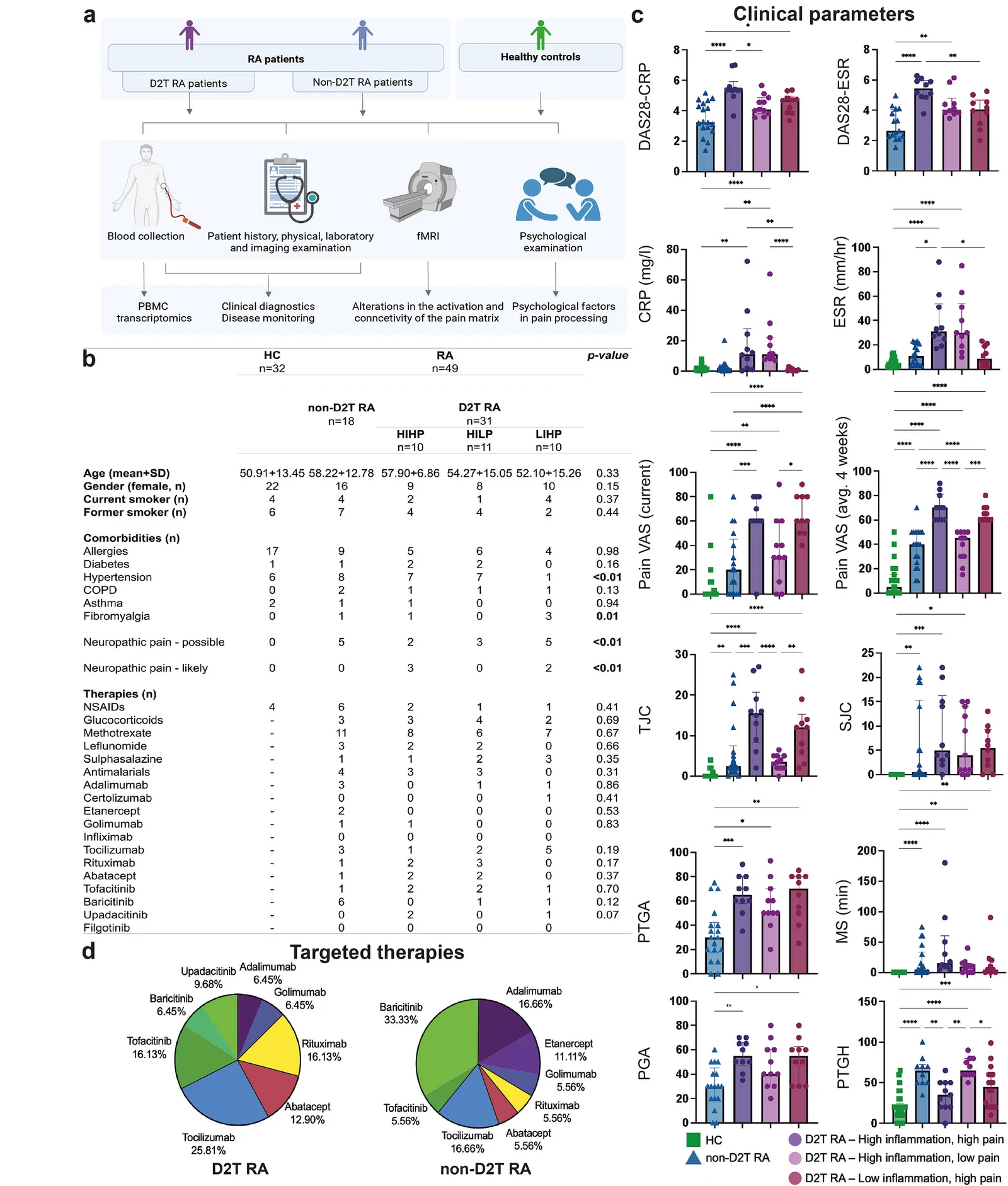
Clinical and demographic characteristics of the study cohort. Study design (**a**) Clinical and demographical information of the participants. Continuous variables are presented as mean + standard deviation (SD) and are compared using one-way ANOVA. Categorical variables are presented as number (n) of patients affected and were compared with Fisher’s exact test. **b** Comparative analysis of the clinical parameters of participants. Bars represent the median (whiskers are set to interquartile range, IQR). After testing for normality, group differences were assessed using one-way ANOVA for PTGA, PGA, PTGH, TJC, PainVAS (average and current), and DAS28-CRP/ESR, and using the Kruskal–Wallis test for SJC, MS, ESR, and CRP. P-values < 0.05 were considered statistically significant (*p < 0.05, **p < 0.01, ***p < 0.001, **p < 0.0001) (**c**) Specific targeted therapies the patients received. **d** *COPD, chronic obstructive pulmonary disease; CRP, C-reactive protein; DAS28, Disease Activity Score in 28 joints; ESR, erythrocyte sedimentation rate; HC, healthy control; MS, morning stiffness; PBMC, peripheral blood mononuclear cell; PGA, physician global assessment; PTGA, patient global assessment; PTGH, patient global health; RA, rheumatoid arthritis; SJC, swollen joint count; TJC, tender joint count; VAS, visual analog scale*

The study consisted of four major parts for all participants: (1) a clinical examination, (2) fMRI examinations, (3) a psychological evaluation and (4) PBMC transcriptomics. (Fig. 2a, Suppl. Mat.1).

Briefly, all participants underwent a comprehensive clinical evaluation, including a detailed medical interview, physical examination, laboratory test to assess inflammatory parameters and joint ultrasound when indicated. Pain characteristics were further investigated using the PainDetect Questionnaire [36] and 2016 ACR fibromyalgia criteria [37]. The detailed psychological analysis consisted of an in-depth interview, the Rorschach test, and self-reporting questionnaires, the results of which were individually analyzed and incorporated into the qualitative analysis.

Subjects were scanned using a 3 T MRI scanner (MAGNETOM Prisma^Fit^, Siemens-Healthineers, Erlangen, Germany) using a 64-ch head-neck coil. The MRI study protocol included the following sequences: 3D T1 magnetization-prepared rapid acquisition with gradient echo (MPRAGE), 2D FID-EPI resting-state functional MRI (rs-fMRI) with field mapping to reduce image distortions due to B0 inhomogeneities.

Rs-fMRI evaluation was carried out by using CONN (21.a) [38, 39] and SPM (12.7771) [40]. For the neuroimaging analyses, sex, age and recruitment site were originally included as covariates in the general linear models. First-level analyses included local correlation maps (LCOR) calculation estimated as the weighted average of all short-range connections between a voxel and a 25 mm FWHM Gaussian neighborhood area; intrinsic connectivity (ICC) representing a measure of node centrality at each voxel, characterized by the strength of connectivity between a given voxel and the rest of the brain; interhemispheric correlation maps (IHC) calculation characterizing the strength of homotopic functional connectivity between the two hemispheres; fALFF analysis representing a relative measure of blood-oxygen-level dependent (BOLD) signal power within the frequency band of interest (0.008–0.09 Hz) compared to that over the entire frequency spectrum; ROI-to-ROI connectivity analysis characterizing the functional connectivity between each pair of regions, and seed-based connectivity maps estimation characterizing the spatial pattern of functional connectivity with a seed area. ROIs were defined by the modified AAL atlas distributed with CONN and the MIST122 atlas [41]. Between scans, standardized thermal pain stimulation was applied to both forearms. Thermal pulses were generated by the combined fMRI model of Pathway Pain Evaluation System (Medoc Advanced Medical Systems, Ramat Yishai, Israel) with a CHEPS (Contact Heat Evoked Potential Stimulator) thermode (Suppl. Mat.1).

Peripheral blood was collected once during the study period for the bulk RNA-seq measurements in EDTA containing tube (Vacuette, Greiner Bio-One, Kremsmünster, Austria). PBMCs were isolated by density gradient centrifugation using Ficoll Histopaque-1077 (Sigma-Aldrich, Merck KGaA, Darmstadt, Germany). The purification of total RNA was carried out as previously described [27]. RNA libraries were generated using the QuantSeq 3' mRNA-Seq Library Prep Kit FWD for Illumina (Lexogen, Vienna, Austria). Illumina sequencing was performed on the NovaSeq 6000 instrument (Illumina, San Diego, CA, USA) with 1 × 75 run configuration (Suppl. Mat.1).

### Statistics

For both the clinical and psychological analyses, comparisons between two groups were conducted using a two-sample t-test and a non-parametric Mann–Whitney test, while comparisons involving more than two groups employed ANOVA or the Kruskal–Wallis test. Cohen’s d was used to quantify effect size, with values > 0.5 considered medium to support the interpretation of the magnitude and clinical relevance of group differences (Suppl. Mat.1). As a sensitivity analysis, we assessed the potential influence of sex on the psychological findings by testing interaction effects between group and sex where model estimation was feasible (Suppl. Mat.2).

Transcriptomic data were further analysed with bioinformatic tools. Functional analysis (gene annotations) was prepared using the Gene Ontology (GO) and Reactome databases. While multiple testing correction reduces the likelihood of false positives, it may also mask biologically meaningful signals; therefore, in our transcriptomic analysis, results with fold change ≥ 1.2 and raw *p* < 0.05 were considered for interpretation to capture potentially relevant findings (Suppl. Mat.1). For transcriptomic sensitivity analyses, statistical power was estimated using RNASeqPower based on the biological variability of the voom-normalised expression data (Suppl.Mat.3), and differential expression analyses were repeated with sex included as a covariate in the limma design matrix (Suppl.Mat.4). Ingenuity Pathway Analysis (IPA) software (QIAGEN, Venlo, Netherlands), version 111,725,566, was used to run a core analysis on differentially expressed genes to identify implicated canonical pathways, regulatory networks, and upstream regulators.

## Results

### Clinical and demographic characteristics

A total of 81 individuals were included: 32 healthy controls (HCs) and 49 RA patients, who were divided into D2T (*n* = 31) and non-D2T (also defined as low inflammation, low pain (LI-LP)) (*n* = 18) subgroups based on the EULAR’s 2020 D2T RA definition [4] (Fig. 1, Suppl.Mat.1). The D2T group was further categorized based on the level of inflammation and pain into *high inflammation, high pain (HI-HP)*, *high inflammation, low pain (HI-LP), low inflammation, high pain (LI-HP)* subgroups. Groups were broadly similar in age and sex distribution. However, comorbidities such as hypertension, fibromyalgia, and neuropathic pain were more frequent in the RA population, particularly among D2T patients. Disease activity markers (DAS28-CRP/ESR), inflammatory parameters (CRP, ESR), as well as symptom- and pain-related outcomes (VAS, PGA, PGH, PTGA, MS) showed several differences both among the D2T subgroups and in comparison to non-D2T RA and HCs. Detailed demographic and clinical characteristics are summarized in Fig. 2 and Suppl.Mat.5,6.

### Altered resting-state functional activity and connectivity patterns in the brain of D2T-RA patients

**Fig. 3:**
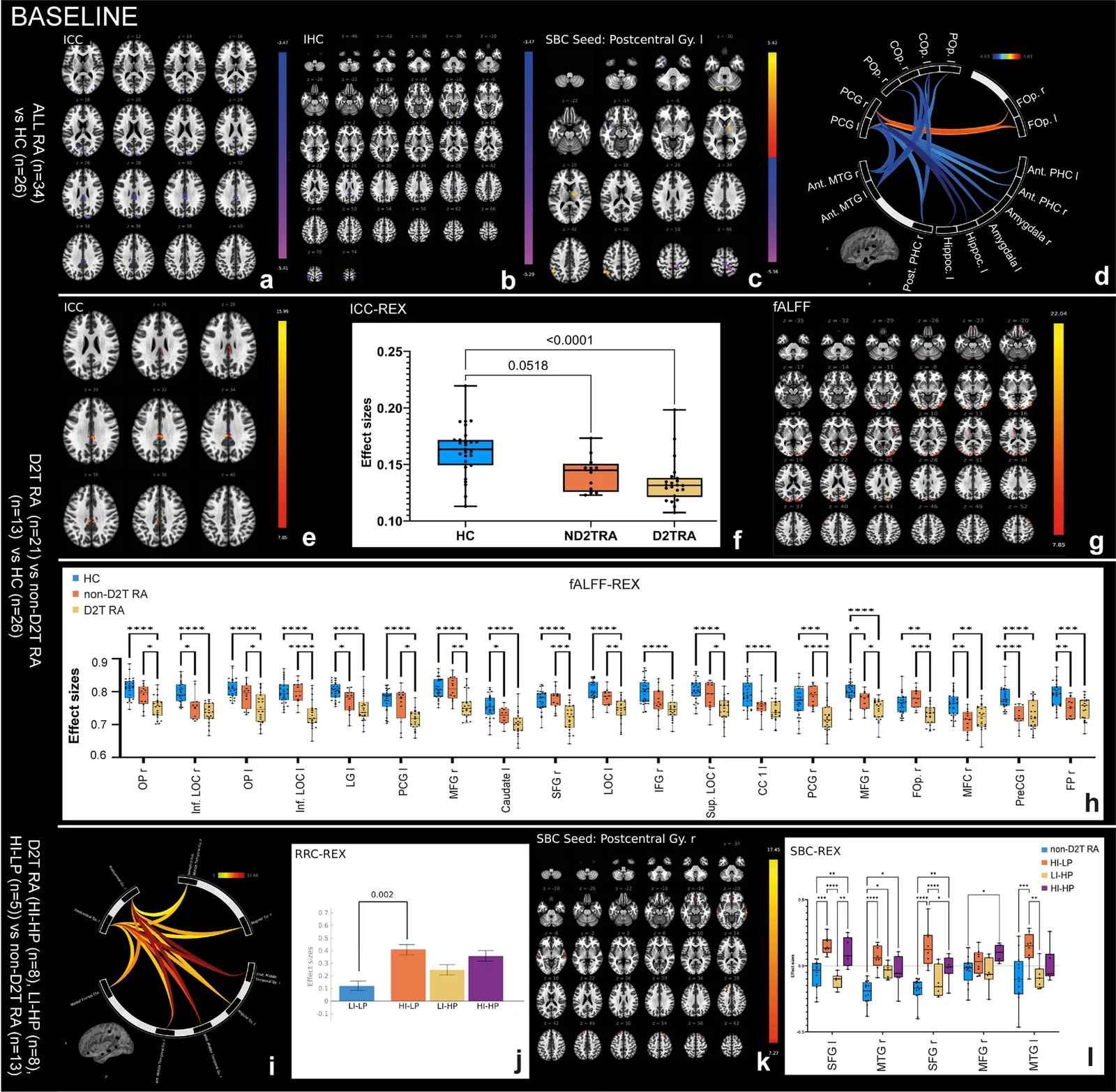
**fMRI reveals distinct connectivity and activity patterns in D2T RA and its subgroups.** The first row summarizes the most important findings of all RA vs. HC comparisons: intrinsic connectivity (**a**) and interhemispheric connectivity (**b**) identify the PCG as one of the most significantly different areas. Seed-based analysis of PCG (**c**) and ROI-to-ROI analysis (**d**) reveal significantly different connectivity patterns. The seed is not marked, brain areas showing positive associations are red-yellow, and inverse associations are blue-purple. The second and thirds rows exhibit HC vs. D2T vs. non-D2T RA comparison: The ANOVA analysis of intrinsic connectivity and fALFF patterns (**e** and **g**) and their subsequent pairwise analyses (**f** and **h**, respectively). The fourth row shows the analysis of different RA subpopulations: ROI-to-ROI connectivity analysis (**i**, **j**) and seed-based connectivity analysis using the PCG as seed (**k**, **l**). The seed is not marked, brain areas showing positive associations are red-yellow. Detailed statistical results, including exact comparisons with *p* values are provided in Suppl.Mat. 7-8. *ant, anterior; CC, cerebellum crus; COp, central operculum; D2T, difficult-to-treat; fALFF, fractional amplitude of low-frequency fluctuations; FOp, frontal operculum; HC, healthy controls; Hippoc, hippocampus; HI, high inflammation; HP, high pain; ICC, intrinsic connectivity; IHC, interhemispheric connectivity; IFG, inferior frontal gyrus; LG, lingual gyrus; LI, low inflammation; LOC, lateral occipital cortex; LP, low pain; MFG, middle frontal gyrus; MTG, middle temporal gyrus; OP, occipital pole; PCG, postcentral gyrus; PHC, parahippocampal cortex; POp, parietal operculum; post, posterior; PreCG, precentral gyrus; RA, rheumatoid arthritis; RRC, ROI-to-ROI connectivity; SBC, seed-based connectivity; SFG, superior frontal gyrus; sup, superior; tem-occ, temporo-occipital*

RA has been linked to altered brain connectivity, reflecting the impact of the disease on neural function [20–26]. To explore this further, we used a baseline rs- fMRI to investigate how brain connectivity alterations in RA may relate to the D2T state. Intrinsic connectivity measures how strongly a given voxel is functionally connected to the rest of the brain, allowing the identification of regions with altered neural activity. This revealed the importance of the posterior cingulate cortex (PCC), a central node of the default mode network (DMN), showing significantly reduced connectivity in all RA patients compared to HCs. (Fig. 3a, Suppl.Mat.7) When analyzing further, focusing on the differences of the D2T subgroup, ANOVA identified a significant cluster in the PCC with lower intrinsic connectivity in D2T RA patients compared to HCs (Fig. 3.e–f, Suppl.Mat.7–8).

Interhemispheric connectivity, reflecting the connections between corresponding areas across the two hemispheres, was also significantly reduced in all RA patients between many bilateral areas but most significantly in the sensory cortices, PCC, insula, and temporal regions (Fig. 3b, Suppl.Mat.7). Based on these findings, a seed-based connectivity analysis was conducted, using the left postcentral gyrus (PCG) as a seed to further explore the connections of the somatosensory areas, first in all RA patients compared to HCs. Functional connectivity strength was significantly increased in RA between the seed region and the right thalamus and left angular gyrus (AG). However, consistent with interhemispheric connectivity, connectivity strength was reduced between the seed and the contralateral somatosensory cortex and additionally between the seed and bilateral temporal poles and parts of the bilateral hippocampal formation (Fig. 3c, Suppl.Mat.7). To further explore whether these connectivity alterations are distinct in the D2T population, we conducted a seed-based connectivity analysis on the subgroups also using the PCG as seed (Fig. 3k, Suppl.Mat.7). This showed significantly altered connectivity between the PCG and several areas, including the medial prefrontal cortex (MPFC), superior frontal gyrus (SFG), and medial temporal gyrus (MTG), identifying the somatosensory cortex as the center of this altered functional network during baseline. Pairwise comparisons (Fig. 3l, Suppl.Mat.8) showed that high inflammation subgroups (HI-LP, HI-HP) differed significantly from low inflammation subgroups (non-D2T RA, LI-HP) in functional connectivity strength between the somatosensory cortex and the previously defined areas (MPFC, SFG, and MTG) suggesting that inflammation, rather than pain, may be an important factor associated with these changes in D2T RA.

Region of interest (ROI)-to-ROI analysis further confirmed this altered connectivity pattern in not only all RA patients (Fig. 3d, Suppl.Mat.7), but also when comparing RA subgroups with each other. In the four patient subgroups, significantly different connectivity strength was observed between the bilateral PCG and several temporal, parietal, and frontal regions (Fig. 3i, Suppl.Mat.7). Subsequent pairwise analysis (Fig. 3j, Suppl.Mat.8), explained the previous ANOVA results; the non-D2T RA subgroup (LI-LP) showed reduced connectivity strength as compared to the HI-LP subgroup of D2T RA population.

In addition to connectivity analyses, we further investigated regional brain activity using fractional amplitude of low-frequency fluctuations (fALFF) analysis. Given our focus on the D2T condition, fALFF analysis was conducted specifically in D2T and non-D2T RA subgroups to examine altered neural activity patterns beyond connectivity changes. Lower fALFF was identified in the non-D2T RA group compared to HCs in the following areas: lateral occipital cortex (LOC), lingual gyrus, caudate, middle frontal gyrus (MFG), MPFC, PCG, and frontal pole (FP). Compared to non-D2T RA, D2T RA patients exhibited significantly reduced fALFF in the frontal operculum, MFG, SFG, LOC, occipital pole (OP), and PCG (Fig. 3g,h, Suppl.Mat.7).

These results demonstrate that D2T RA is associated with characteristic connectivity alteration, particularly in brain regions involved in sensory processing, pain modulation, and cognitive control. Detailed statistical results are provided in Suppl.Mat.7–8.

### Distinct perception and processing of an acute pain stimulus of the brain of D2T RA patients and their subgroups

Pain processing involves a dynamic interaction of different brain areas related to sensory, affective, and cognitive networks, which are often altered in chronic inflammatory and pain conditions [20–26, 42–50]. Our baseline resting-state analysis revealed alterations affecting these networks. Therefore, we next investigated how acute painful stimuli may influence these connectivity patterns.

**Fig. 4:**
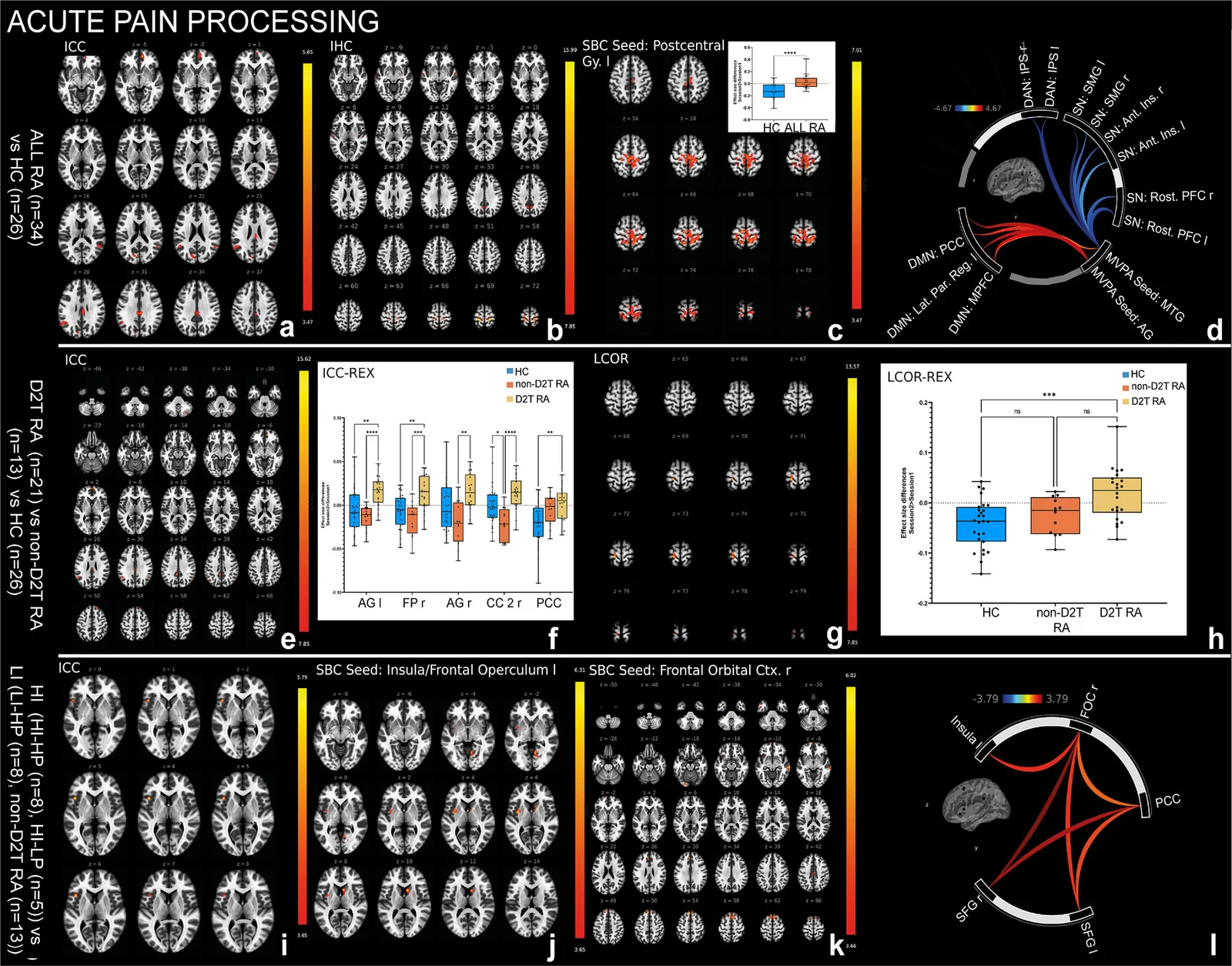
Functional connectivity and activity patterns in the brain of D2T RA patients and their subgroups in response to acute pain stimulus. The first row summarizes the most important findings of all RA vs. HC comparisons: intrinsic connectivity (**a**) and interhemispheric connectivity (**b**) identify the PCG as one of the most significantly different areas. Seed-based analysis of PCG (**c**) and ROI-to-ROI connectivity analysis (**d**) reveal significantly different connectivity patterns. (The seed is not marked, brain areas showing positive associations are red-yellow). The second row exhibits HC vs. D2T vs. non-D2T RA ANOVA analyses: intrinsic connectivity patterns (**e**), the corresponding pairwise posthoc test (**f**), and local correlation analysis along with pairwise posthoc analysis (**g** and **h**). The third row shows the analysis of different RA subpopulations: ROI-to-ROI connectivity analysis (**i**, **j**) and seed-based connectivity analysis using insula, frontal operculum and FOC as seed (**k**, **l**) (The seed is not marked, brain areas showing positive associations are red-yellow). Detailed statistical results, including exact comparisons with p-values are provided in Suppl.Mat. 9. *AG, angular gyrus; ant, anterior; CC, cerebellum crus; D2T, difficult-to-treat; DAN, dorsal attention network; DMN, default mode network; FOC, frontal operculum cortex; FP, frontal pole; HC, healthy controls; HI, high inflammation; HP, high pain; ICC, intrinsic connectivity; IHC, interhemispheric connectivity; Ins, insula; IPS, inferior parietal sulcus; LCOR, local correlation; LI, low inflammation; LP, low pain; lat, lateral; MPFC, medial prefrontal cortex; MTG, middle temporal gyrus; MVPA, multivariate pattern analysis; par, parietal; PCC, posterior cingulate cortex; PCG, postcentral gyrus; PFC, prefrontal cortex; RA, rheumatoid arthritis; RRC, ROI-to-ROI connectivity; SBC, seed-based connectivity; SFG, superior frontal gyrus; SMG, supramarginal gyrus; SN, salience network; rost, rostral*

Acute pain stimulation significantly enhanced the intrinsic connectivity of the PCC in contrast to baseline, along with MPFC, left AG, and left intracalcarine cortex in all RA patients compared to HCs (Fig. 4a, Suppl.Mat.9). Further analysis focusing on the D2T population revealed significantly different intrinsic connectivity in the main nodes of the DMN (MPFC, PCC, lateral parietal areas) between D2T RA, non-D2T RA, and HCs (Fig. 4e, Suppl.Mat.9), primarily driven by the D2T RA subgroup, as shown in post-hoc pairwise comparisons (Fig. 4f, Suppl.Mat.9).

Compared to baseline, interhemispheric connectivity was also significantly enhanced in all RA patients, primarily between the bilateral sensory areas, PCC, and MTG after acute pain stimulation (Fig. 4b, Suppl.Mat.9). To further explore the role of sensory areas, seed-based connectivity analysis was conducted using the left PCG as the seed, consistent with the baseline approach. This analysis showed altered resting-state connectivity within the bilateral somatosensory areas after painful stimulation driven by a marked reduction in HCs as compared to a slight increase in all RA patients (Fig. 4c, Suppl.Mat.9). Notably, the left sensorimotor cortex showed increased local correlation driven by the D2T RA subgroup, suggesting altered local functional integration in pain-related sensory areas potentially associated with the D2T state (Fig. 4g,h, Suppl.Mat.9).

To assess broader network-level changes related to RA, multivariate pattern analysis was conducted, which identified the left MTG and right AG as key areas showing altered connections in response to acute pain in all RA patients. These areas were used as seeds in a subsequent ROI-to-ROI analysis, including the nodes of the main resting-state networks (DMN, salience (SN), dorsal attention (DAN), sensorimotor (SMN) and frontoparietal (aka central executive, CEN) networks). This comparison (Fig. 4d, Suppl.Mat.9) reported increased resting-state connectivity in RA between the right AG and the DMN, along with weaker connectivity between the left MTG and DAN and SN.

To investigate how inflammation-driven connectivity changes observed at baseline evolved following pain stimulation, further subgroup analysis was conducted. Intrinsic connectivity was increased in the HI subgroups (HI-HP, HI-LP) in the left insula/frontal opercular area (Fig. 4i, Suppl.Mat.9), and seed-based connectivity analysis using this region as a seed revealed higher functional connectivity strength with the bilateral caudate after a painful event, compared to those with low inflammation (Fig. 4j, Suppl.Mat.9). Additionally, using the right orbitofrontal cortex (OFC) as a seed showed increased functional connectivity strength in patients with high inflammation (HI-HP, HI-LP) after pain, particularly with the PCC, MPFC/paracingulate cortex, SFG, and MTG. These findings were further supported by ROI-to-ROI connectivity analysis, suggesting a potential role of inflammation in modulating pain-related network connectivity (Fig. 4k,l, Suppl.Mat.9). Detailed statistical results are provided in Suppl.Mat.9.

### Major psychological alterations revealed in the background of the D2T disease state

**Fig. 5:**
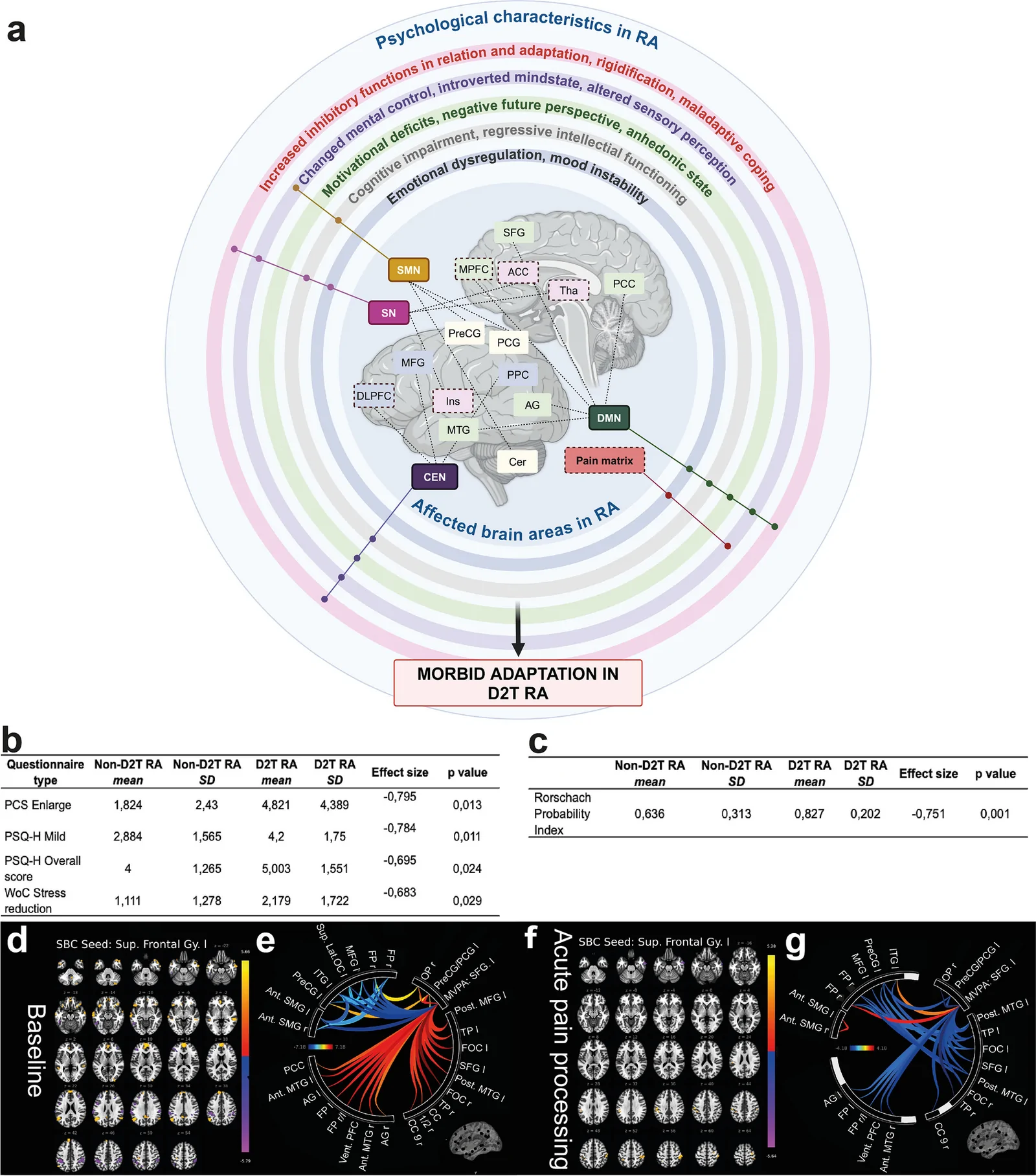
Characteristic psychological alterations of D2T RA patients. Schematic summary of the psychological analysis of all RA patients, with 'morbid adaptation' specifically characteristic of D2T RA. Brain areas found to be affected in RA, and their relation to major functional brain networks are marked as connecting lines. Dots indicate if there is a link between psychological symptoms and the affected brain networks. **a** Significantly different questionnaire results in the D2T vs non-D2T RA comparison (*p* < 0.05). **b** RPI determined a higher degree of psychological involvement in D2T patients. **c** Groups were compared with a two-sample t-test, and Cohen’s d was used to quantify effect size, with values > 0.5 considered medium. **b**-**c** Relation between RPI and baseline resting-state functional connectivity changes (**d**), as well as resting-state functional connectivity changes after acute painful stimulation (**f**) in all subjects. The seed is not marked, brain areas showing positive associations are red-yellow, and inverse associations are blue-purple. ROI-to-ROI connectivity analysis of the baseline network (**e**) and the network after acute painful stimulation (g) Detailed statistical results of the fMRI measurements are provided in Suppl.Mat. 10. *ant, anterior; CC, cerebellum crus; CEN, central executive network; DMN, default mode network; FOC, frontal opercular cortex; FP, frontal pole; ITG, inferior temporal gyrus; l, left; MTG, middle temporal gyrus; MVPA, multivariate pattern analysis; OP, occipital pole; PCG, postcentral gyrus; PCC, posterior cingulate cortex; PCS, Pain Catastrophizing Scale (enlargement subscale); post, posterior; PreCG, precentral gyrus; r, right; PSQ-H, Pain Sensitivity Questionnaire – Hungarian version (mild pain and overall score); ROI, region of interest; RRC, ROI-to-ROI connectivity; SBC, seed-based connectivity; SFG, superior frontal gyrus; SMG, supramarginal gyrus; sup, superior; TP, temporal pole; vent, ventral, WoC, Ways of Coping Questionnaire (stress reduction)*

Our psychological assessment combined individual clinical interviews, standardized questionnaires, and the Rorschach projective test to examine mental health profiles across RA subgroups. Based on the individual psychological profiling among the 31 D2T RA patients, only two were free of clinically relevant psychological conditions. The most frequent diagnoses included histrionic personality disorder (16%), anxiety disorders (10%), and other specified personality disorders (10%), with isolated cases of adjustment disorder, cyclothymic traits, and schizofreniform features. D2T RA patients frequently showed signs of emotional instability, emotional suppression, and interpersonal challenges. About one-third (32%) demonstrated cognitive difficulties, including rigid thinking and less flexible coping strategies. A tendency toward heightened illness-focused attention appeared to be associated with lower self-esteem and a negative outlook. Future orientation was frequently pessimistic or uncertain (78%), and clinically relevant depressive symptoms were reported by 42%, often linked to illness burden (Fig. 5a).

A direct comparison of D2T to non-D2T RA revealed many overlapping psychological traits but also identified some differences. In the questionnaire-based comparison, the D2T group seemed to exhibit a greater tendency to magnify pain experiences and focus on somatic complaints (PCS-Enlarge). D2T RA patients also showed higher sensitivity to pain (PSQ-H, mild and overall score), suggesting a lower pain threshold and heightened sensitivity to painful stimuli. D2T patients tended to prefer emotion-focused strategies that provide quick relief when facing difficulties over problem-focused coping (WoC stress reduction) (Fig. 5b). Depressive symptoms could be observed for 13 D2T RA patients (42%), 4 non-D2T RA patients (22%), and 2 HCs (6%).

Rorschach comparisons further characterized the differences between D2T and non-D2T RA. D2T RA patients were characterized by emotional indistinction, less mature emotion regulation, and maladaptive coping strategies. In addition to internal emotional instability, lability, and increased suffering, a predominantly outward-directed, externalizing communication pattern was observed, in which extensive verbalization and dramatized expression of distress played a central role. D2T group showed a significantly altered personality organization compared to non-D2T RA, which is characterized by increased reliance on external help, externalization of responsibility, and psychological adjustment to the disease (Suppl.Mat.12).

Overall, these data together point toward a pattern of greater psychosocial burden, reduced emotional variability, and coping strategies that are rigid or maladaptive in D2T RA. This can properly be described by our newly proposed term “morbid adaptation”, characterized by a pathological attempt to adapt accompanied by hopelessness, motivational decline, and the lack of positive outlook (Fig. 5a). These findings shed light on the complex psychological challenges that patients with D2T RA experience, indicating that their treatment with pharmacotherapeutics only might be especially difficult.

### RA characteristic psychological changes are associated with a specific brain connectivity network pattern

Evaluating different parameters of the Rorschach test allows us to identify a characteristic psychological profile of a patient with features that usually are not revealed in self-report questionnaires. We utilized the Rorschach test, applying Mérei's notation, which includes various statistically derived scales and indices [51]. Our group previously developed a formula called the Rorschach Probability Index (RPI) that predicts the probability of the subject suffering from chronic pain solely on Rorschach test indices [52], indicating that chronic pain patients exhibit measurable psychological profiles (Suppl.Mat.13). The RPI is generally low among HCs, and as the RPI increases, more characteristic features of chronic pain patients are present. We tested the RPI in our cohort and found an accuracy rate of 82% in successfully identifying RA patients based on Rorschach results only. This means that RA patients also exhibit a characteristic personality profile, showing several similarities with the pathological psychological changes of a chronic pain patient; however, it does not fully overlap. RPI alone also determined a higher degree of psychological involvement in D2T patients. In the case of RPI, we measured 82.7% in the D2T group and 63.6% in the non-D2T group (Fig. 5c). The significant difference with an appropriate effect size indicates that D2T patients are more likely to suffer from mental-emotional and cognitive difficulties than non-D2T patients.

While the psychological assessment using the Rorschach test has been explored in many pathological conditions, there is growing interest in integrating somatic modalities, such as fMRI, with projective tests [18]. Therefore, we investigated how the relationship between RPI and functional brain connectivity changes across the whole study population at baseline and following acute pain. Multivariate pattern analysis identified the left SFG, which was used as a seed in the seed-based connectivity analysis, along with RPI as an independent variable of interest. This resulted in significant associations between the RPI and the functional connectivity between the seed and several brain regions (bilateral PCG/supramarginal gyrus (SMG), PCC, MPFC, AG, MTG, FP, TP, OFC, etc.). These data, together with a subsequent ROI-to-ROI analysis, showed both positive and inverse associations, e.g., higher RPI scores were associated with lower functional connectivity between the left SFG and bilateral PCG, but increased connectivity was noted with the PCC (Fig. 5d,e, Suppl.Mat.10).

After acute pain stimulus, we observed significant changes in this network (Fig. 5.f,g, Suppl.Mat.10). Notably, the previously reduced SFG-PCG connectivity in individuals with high RPI during the baseline (Fig. 5d, Suppl.Mat.10) became significantly stronger after acute pain (Fig. 5f, Suppl.Mat.10). Altogether, this identified network highlights the complex relationship between pain processing and psychological features in RA, further emphasizing the involvement of the DMN and somatosensory cortex in RA-related pathomechanisms.

### Peripheral transcriptomic alterations in D2T RA reveal nervous-system-related functional annotations and associations with brain connectivity

**Fig. 6:**
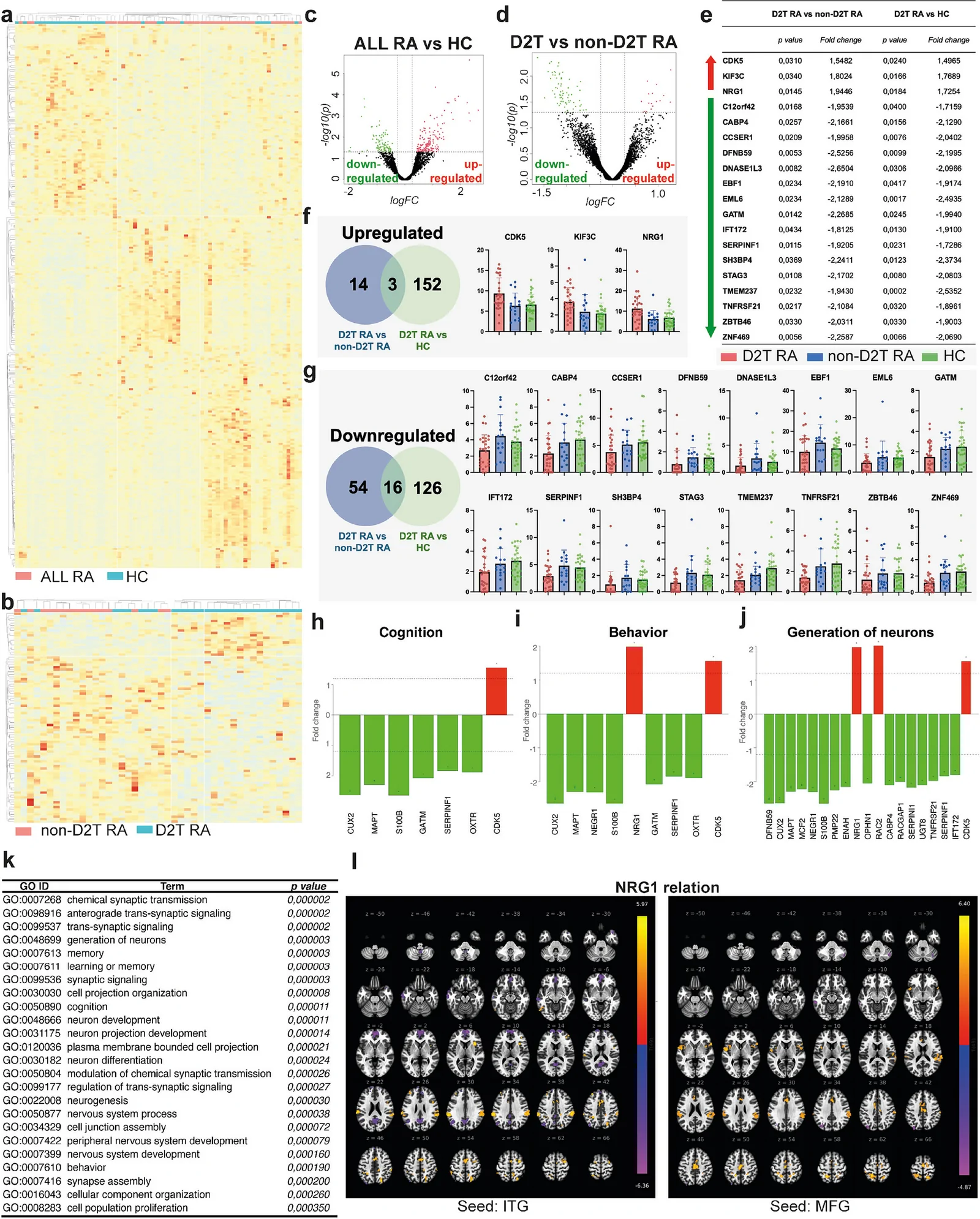
Characteristic transcriptomic alterations of RA patients highlighting the differences of the D2T-RA population. Heatmap of DE genes comparing all RA patients (n = 44) to HCs (n = 29) (**a**) Heatmap of DE genes comparing D2T RA (n = 28) to non-D2T (n = 16) (**b**) Volcano-plot of all RA vs. HC (**c**) and D2T RA vs. non-D2T RA (**d**) List, summary and expression levels (mean + SD) of shared upregulated and downregulated mRNAs in the D2T RA vs. non-D2T RA and the D2T vs. HC comparison (**e**–**g**) Examples of significantly enriched terms in GO Biological Process database: Cognition: p = 0.000011, Behaviour: p = 0.00019, Generation of neurons: p = 0.000003 (**h**-**j**) List of the top 20 significantly enriched terms in GO database categorized under Biological Process (**k**) Brain network related to NRG1 expression in all subjects. The seed is not marked, brain areas showing positive associations are red-yellow, and inverse associations are blue-purple. (**l**) Detailed statistical results of the fMRI measurements, including exact comparisons with *p* values, are provided in Suppl.Mat. 11. *D2T: Difficult-to-treat; GO: Gene Ontology; HC: Healthy Controls; ITG, inferior temporal gyrus; MFG, middle frontal gyrus; RA: Rheumatoid Arthritis*

Recent findings suggest that pathological processes in the brain may be mirrored in peripheral tissues [27–35]. To characterize peripheral transcriptional alterations associated with RA and the D2T disease state, bulk RNA-seq analysis was performed on PBMC samples of 28 D2T RA, 16 non-D2T RA and 29 HCs. When comparing RA to HCs, 269 differentially expressed (DE) mRNAs (174 upregulated and 95 downregulated in RA) were identified (Fig. 6.a,c). Functional enrichment analysis was performed using Gene Ontology (GO) and Reactome databases, which revealed the involvement of the DE genes in *immune processes, oxygen transport, cell signaling, and communication* (Suppl.Mat.14–15).

Next, we wanted to examine whether the transcriptomic profile of D2T patients could distinguish them from the non-D2T group. From the 87 DE mRNAs, 17 were upregulated, and 70 were downregulated (Fig. 6.b,d, Suppl.Mat.16). To identify transcriptomic alterations consistently associated with the D2T group, we compared the DE mRNAs identified in the D2T RA vs. non-D2T RA (Suppl.Mat.16) analysis with those identified in the D2T RA vs. HCs analysis (Suppl.Mat.17). The common DE transcripts include 16 downregulated mRNAs, such as EBF1, SERPINF1, or TNFRSF21, and three upregulated transcripts including NRG1, KIF3C, and CDK5 (Fig. 6.e–g). These transcripts and the related pathways potentially represent candidate peripheral molecular features associated with D2T RA; however, they require validation in independent cohorts.

Functional enrichment analysis identified GO terms categorized under “Biological Processes” such as *chemical synaptic transmission, generation of neurons, memory, cognition, or behavior* (Fig. 6.h–k). Additionally, enriched GO Molecular Function and Cellular Component terms included *transmembrane transporter binding* and *neuronal cell body* or *synapse* while Reactome analysis identified terms including *nuclear signaling by ERBB4* (Suppl.Mat.18).

Given the occurrence of functional annotations related to neuronal processes in the peripheral transcriptomic results, we next examined whether selected mRNAs identified in the D2T-related transcriptomic analyses were associated with functional connectivity measures. Peripheral NRG1 expression was associated with alterations in baseline resting-state functional connectivity across all participants. Multivariate pattern analysis identified the right MFG and inferior temporal gyri (ITG) as seed regions with high functional connectivity heterogeneity, explained by NRG1 expression (Fig. 6l, Suppl.Mat.11). Seed-based connectivity analysis showed that higher NRG1 expression was linked to increased MFG connectivity with bilateral PCG, insular and opercular cortices, a cluster encompassing both the ACC and PCC, and supplementary motor cortex, with an inverse association in the left superior LOC. Similarly, higher NRG1 expression enhanced functional connectivity between ITG and frontal, parietal and temporal regions while inversely associating with medial prefrontal and posterior midline areas. These analyses identified an association between peripheral NRG1 expression and functional connectivity patterns involving brain regions relevant to pain processing, sensorimotor integration and cognitive-affective functions [21–23, 26, 45, 47, 49, 50, 53].

### Peripheral transcriptomic profiles differ across pain and inflammation phenotypes in D2T RA

**Fig. 7:**
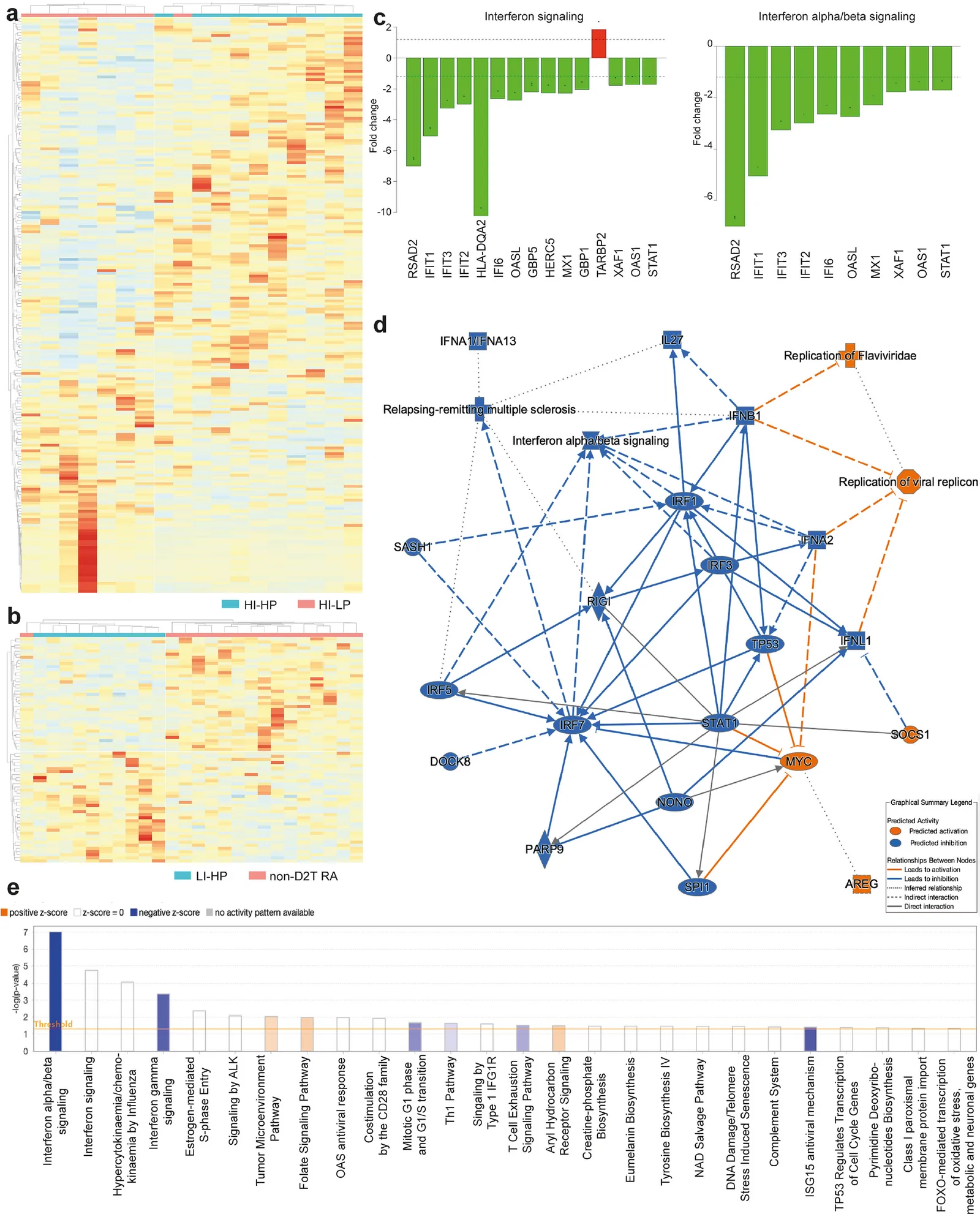
Characteristic transcriptomic alterations of different subgroups of D2T patients. Heatmap of DE genes comparing all D2T RA patients with high inflammation and high pain (HI-HP, n = 10) and high inflammation and low pain(HI-LP, n = 8) (**a**) Heatmap of DE genes comparing D2T RA patients with low inflammation, high pain (LI-HP, n = 10) and non-D2T RA (also referred as LI-LP, n = 16) (**b**) Based on enrichment analysis of DE genes using Reactome database Interferon signaling (p = 2.4E-06) was significantly altered, with Interferon alpha/beta signaling (p = 4.96E-08) yielding the highest significance. Each bar represents a DE gene associated with the given term, with red bars representing upregulated and green bars downregulated (**c**) Graphical summary generated by Ingenuity Pathway Analysis based on DE genes between HI-HP and HI-LP patients, integrating predicted upstream regulators, affected biological functions, and pathways. Arrows indicate directional relationships based on the curated IPA database. **d** Canonical pathway analysis of DE genes using IPA demonstrating the significance of interferon pathways. Bars represent –log_10_ (*p*-value); color indicates predicted activation (orange), inhibition (blue), or no prediction (grey) based on z-scores. **e** *D2T: Difficult-to-treat; HC: Healthy Controls; HI, high inflammation; HP, high pain; LI, low inflammation; LP, low pain; RA: Rheumatoid Arthritis*

Due to the heterogeneity of the D2T population, transcriptomic analysis was conducted among RA subgroups with distinct pain and inflammatory parameters to identify potential subgroup-specific molecular signatures. Comparing the D2T RA HI-HP and HI-LP subgroups revealed 208 DE mRNAs mainly associated with disease-specific, inflammatory, and pain-related processes. Altogether 129 mRNAs were upregulated (e.g. GPSM1, CR2, HLA-DRB1 or IGF1) while 79 downregulated (e.g. HLA-DQ2, SIGLEC1 or IFIT1,2,3) (Fig. 7a). Functional analysis based on GO and Reactome databases revealed that transcripts downregulated in HI-HP were significantly enriched for interferon (IFN)-related pathways compared to HI-LP (Fig. 7c, Suppl.Mat.19–20). Consistently, Ingenuity Pathway Analysis (IPA) identified *Interferon alpha/beta signaling* as the most significantly enriched pathway in the HI-HP-HI-LP comparison and predicted its inhibition in HI-HP compared with HI-LP (Fig. 7.d,e).

In the LI-HP vs. non-D2T RA comparison 35 upregulated and 38 downregulated DE mRNAs were identified. Functional enrichment analysis identified terms related to both immune function and nervous system-related processes including the *glutamate neurotransmitter release cycle, complement receptor-mediated signalling*, or *leukocyte migration*. (Fig. 7.b, Suppl.Mat.21–22).

## Discussion

**Fig. 8:**
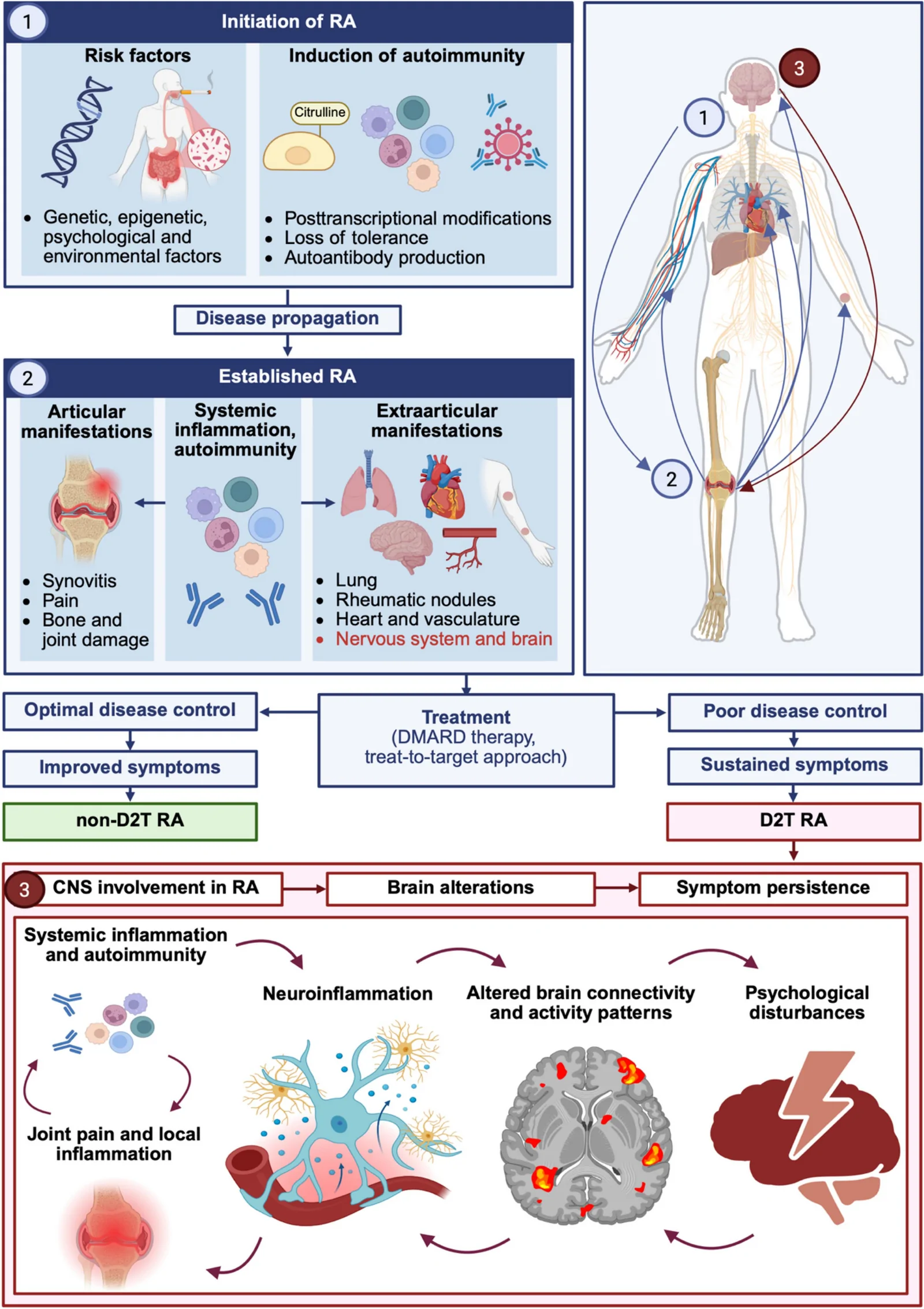
Potential mechanisms of CNS involvement in RA hypothetically contributing to the development of the D2T disease state. RA is a systemic autoimmune disease that affects not only the joints but also several organ systems, including the CNS. Persistent inflammation and autoantibody production may lead to sustained neuroinflammatory processes that influence the activity and connectivity of the brain. These changes could contribute to the psychological symptoms often experienced by RA patients, including emotional dysregulation and cognitive dysfunction. Moreover, these alterations may also affect pain perception and contribute to pain chronification, thereby leading to symptom persistence. Such changes may influence the modulatory effect of the CNS on peripheral structures and might further sustain inflammatory activity and/or pain at the level of joints. This hypothetical circulus vitiosus may be present in all RA patients. However, based on our findings, these mechanisms could be a major contributor to the D2T disease state. *CNS: central nervous system; D2T: difficult-to-treat; DMARD: disease-modifying antirheumatic drug; RA: rheumatoid arthritis*

To our knowledge, this is the first study providing a comprehensive, multimodal analysis of D2T RA integrating fMRI, clinical and psychological assessments, along with transcriptomic profiling. While RA primarily starts with synovial inflammation, it extends beyond the joints, impacting multiple organ systems, including the nervous system. These systemic manifestations contribute to the overall disease state, influencing symptom severity and treatment response [1, 6, 54]. Our findings support the presence of CNS-related alterations in RA, particularly in D2T patients, characterized by altered brain connectivity, changes in central pain processing, and cognitive-emotional disturbances, accompanied by peripheral molecular signatures. These alterations may contribute to symptom persistence and the complex clinical phenotype of the D2T disease state (Fig. 8).

Our results support and extend prior research demonstrating altered brain functioning in RA [20–26] and other chronic pain conditions [23, 42, 43, 46], as well as psychological disturbances [55–58]. We observed functional alterations in several brain areas, primarily the PCC, PCG, and SFG, alongside major brain networks (DMN, SMN, CEN, and SN). Previous studies in RA suggested that such alterations may be associated with a complex interplay between peripheral inflammation and central mechanisms, potentially influencing symptom severity, including pain and psychological dysfunction, as well as treatment response [20–26].

The PCC, as the central hub of the DMN, is active during rest and involved in cognitive processes, such as autobiographical memory retrieval and future planning, and regulates the shift from internal to external attention focus [53]. PCC disruptions in all RA patients, but especially observed in D2T RA, may contribute to enhanced self-referential thoughts and difficulties in switching the focus to task-related thoughts, which were also observed in the D2T population. Decreased PCC functional connectivity may also contribute to cognitive impairments in major depressive disorder (MDD) [56–58]. MDD has a higher prevalence among RA patients, which we can also observe in our cohort, especially in the D2T population and brain areas involved in MDD partially overlap with those affected in RA or in other chronic pain conditions, which may partially explain the higher prevalence of MDD and shared psychological characteristics [56, 58, 59].

We also observed significant functional connectivity disruptions involving the primary somatosensory cortex (S1) an important part of the “pain-matrix” located in the PCG [45, 47, 50]. Altered S1 function was reported in RA [21–23, 26, 49], and similar reductions in connectivity were linked to higher clinical pain severity in fibromyalgia (FM) and complex regional pain syndrome (CRPS) [43, 44, 48], suggesting that our findings of the reduced connectivity strength in RA may also be related to sustained chronic pain. Importantly, the prevalence of FM among RA patients is higher than in the normal population, which also could be observed in our cohort. FM often contributes to the D2T condition further complicating the interpretation of pain-related connectivity alterations [6]. However, this reduced functional connectivity between the bilateral somatosensory cortices changes dramatically after short-term painful stimulation. In contrast to the HC group, where the functional connectivity between the bilateral PCG is significantly reduced shortly after the painful event, the RA group exhibits a slight increase in connectivity strength. This difference alone may contribute to the emergence and the impact of central sensitization observed in RA [60]. In contrast, the enhanced hyperconnectivity with the thalamus, also observed in MDD, may be relevant to somatosensory dysregulation characteristic of both diseases [61].

Our observed fALFF reduction affecting many brain regions, particularly the LOC, LG, caudate, MFG, MPFC, precentral gyrus, and FP, potentially influences somatosensory processing [62, 63], cognitive functions [64, 65], attention [65, 66], learning [65, 67], memory [63, 65, 67] and motor control [68]. D2T patients exhibited a more pronounced reduction, potentially associated with a greater symptom severity and more complex alterations in pain modulation, behavior, and cognitive functions, aligning with their psychological profile.

Alterations of acute cerebral pain processing were previously demonstrated in RA and FM [21–23, 42, 43], and we found that D2T patients share some of these characteristic alterations after painful stimulation. Normally, an acute pain stimulus activates the SN with the involvement of the CEN in the regulatory process, in parallel with the deactivation of the DMN [69]. However, the enhanced connectivity strength in the AG and PCC in D2T patients suggests sustained DMN activity, which may contribute to enhanced pain-related self-referential processing and could be relevant to the observed pain catastrophizing [70]. In a study, painful stimulation of disease-affected joints in RA led to significantly reduced pain-related brain activation compared to HCs [22]. A similar pattern of reduced functional connectivity affecting the DMN and SMN could also be observed in the non-D2T group, potentially suggesting adaptation and compensatory mechanisms in pain perception and modulation. On the contrary, D2T patients exhibited a higher activation compared to HCs and non-D2T patients, indicating distinct alterations in how D2T patients may regulate and perceive pain. Inflammatory activity also seems to affect pain perception, as D2T subgroups with higher inflammation showed altered functional connectivity patterns in the insula/frontal opercular area and in the right OFC, which, being related to emotional processing, behavioral flexibility, and learning [71], might be relevant to pain modulatory mechanisms.

Research has demonstrated psychosocial disturbances in RA [72, 73], which we also observed. Our psychological assessment based on the interviews, questionnaires, and the Rorschach test revealed characteristic alterations, including mood instability, emotional dysregulation, cognitive difficulties, and impaired coping. While questionnaires are the most common tools for assessing psychological state in RA, they primarily capture current symptoms and are less informative about predisposing personality traits or coping mechanisms that might influence disease course [74]. Historical models of RA-specific personality patterns have been proposed [75], but systematic confirmation has been inconsistent, and it remains unclear whether such features could contribute to more persistent or refractory disease manifestations.

The Rorschach test may provide information beyond conscious defenses, revealing deeper facets of emotional functioning [18] not captured by questionnaires. Although there was a debate in the 1990s about standardized systems (e.g., Exner Comprehensive System [76] and the Mérei Hungarian system [51]), the recent introduction of the Rorschach Performance Assessment System, the R-PAS [77] has resulted in renewed interest and enabled the broader application of the test. In parallel, neuroimaging studies have also provided further support for its validity in psychological assessments, demonstrating that the test is associated with brain networks involved in vision, attention, emotion, and social cognition [18]. Beyond personality assessment, the Rorschach test has also been applied in somatic conditions. Studies in chronic inflammatory disease have shown that specific standardized variables and derived indices can predict subsequent disease activity and psychological distress [78], as well as quantify difficulties in identifying and describing emotions through the Rorschach Alexithymia Scale [79]. More recently, certain Rorschach variables have been linked to reduced perceived competence, lower self-esteem, and avoidant coping styles in autoimmune cohorts [80]. These findings highlight the value of projective methods as a complementary tool alongside other psychological assessments in clinical settings, supporting the idea that selected Rorschach variables can offer clinically relevant information on personality and psychosocial functioning that may assist in medical management.

Extending these observations, our study also examined the RPI, a composite measure of selected Rorschach parameters originally developed to estimate risk for chronic musculoskeletal pain in this clinical context [52]. Our study suggests that RA patients exhibit a characteristic personality profile that also shares common features with chronic pain patients, and that this profile is accompanied by functional connectivity pattern mainly involving the relations of SFG, PCC and PCG, which dynamically changes after painful stimulation. This altered connectivity may be associated with increased pain-related rumination that disrupts cognitive control over self-focused thoughts [81, 82], consistent with maladaptive psychological adaptation in D2T RA.

The transcriptomic analysis of peripheral blood-derived cellular components has been successfully used in neurological and neuropsychiatric conditions to identify CNS-related peripheral molecular signatures [27–35]. PBMCs represent an easily accessible immune compartment that is also transcriptionally active and cross-tissue analyses have also demonstrated considerable overlap between transcripts detectable in blood and brain tissues, suggesting that, in addition to providing information on peripheral inflammatory processes, blood-derived expression profiles may also inform about alterations related to CNS functions [34, 35]. This can be of particular importance in D2T RA, representing a more complex disease phenotype [54], with a potential contribution of the interplay of “bottom-up” inflammatory signaling acting on the brain and “top-down” mechanisms as a consequence of CNS alterations leading to symptom persistence [83]. Circulating cytokines and other inflammatory mediators may activate responsive endothelial cells in the blood–brain barrier, leading to increased permeability and immune cell trafficking [84]. In addition, the activation of afferent neural pathways and neuroendocrine mechanisms can also contribute to the consequential neuroimmune alterations [83, 85]. In contrast, CNS-derived factors can be detected in the peripheral circulation, including extracellular vesicles (EVs) carrying proteins and nucleic acids, providing a potential route by which molecular changes related to CNS processes may be accessible in blood [86]. Furthermore, emerging data in RA have reported elevated serum levels of CNS-associated proteins, including neurofilament light chain, glial fibrillary acidic protein, and tau, together with associations between these markers and peripheral inflammatory proteins, providing evidence of blood-detected neuroinflammatory burden [87]. Importantly, EVs can also be taken up by recipient cells at the periphery, facilitating a potential transfer of bioactive cargo, including proteins and nucleic acids, which may provide a potential mechanism by which CNS-related molecular signals might influence peripheral cellular responses [88].

In our cohort, bulk RNA-seq identified peripheral transcriptomic alterations associated with the D2T disease state and with subgroups characterized by different inflammatory and pain phenotypes. Functional enrichment analysis revealed several nervous-system-related annotations, and among altered transcripts, NRG1 was significantly upregulated compared with both non-D2T RA and HCs. Moreover, peripheral NRG1 expression was found to show an association across all participants with functional connectivity patterns involving regions implicated in pain processing, sensorimotor integration, and cognitive-affective function [21–23, 26, 45, 47, 49, 50, 53]. This makes NRG1 a biologically plausible candidate for further investigation, given the reported involvement of NRG1/ErbB signaling in neuronal regulation and inflammatory responses [89–92]. In addition, comparison of high inflammatory subgroups with different extents of pain revealed downregulation of IFN-related transcriptional pathways in patients with high pain. In addition to their established immunomodulatory functions, type I IFNs can exhibit both pro- and antinociceptive effects, depending on concentration and site of action, suggesting alterations in these patterns may be relevant underlying pain heterogeneity among patients with D2T RA [93–95].

However, the transcriptional profile of PBMCs cannot be regarded as a direct surrogate for brain-specific molecular alterations, especially in a disease characterized by systemic immune dysregulation; therefore, nervous system-related functional annotations identified in peripheral samples require cautious interpretation. This is particularly relevant for NRG1, a complex gene with multiple alternatively spliced isoforms and tissue- and cell-type-specific expression. Moreover, blood-based DNA methylation differences involving NRG1 have previously been reported in post-traumatic stress disorder [96], indicating that peripheral molecular findings involving NRG1 are more complex and may not be disease-specific. Accordingly, the association between peripheral NRG1 expression and functional connectivity identifies NRG1 as a candidate peripheral molecular correlate of the observed neuroimaging phenotype, rather than evidence of altered CNS NRG1/ErbB signaling. Therefore, transcriptomic findings should be interpreted as complementary to neuroimaging and psychological results and require validation in independent cohorts before disease-specific or mechanistic conclusions can be drawn.

While our study provides a detailed multimodal characterization of D2T patients, several limitations should be noted. Given the heterogeneity of this population, we aimed to integrate complementary clinical, psychological, neuroimaging and peripheral transcriptomic data to capture different aspects of the D2T disease state. The relatively small sample size reflects the high-content, resource-intensive design of this multimodal study, which required repeated visits, MRI acquisition, experimental pain stimulation, psychological assessment and biological sampling. MRI-related contraindications, claustrophobia and incomplete datasets further reduced the number of participants available for some analyses (Fig. 1), which is particularly relevant for subgroup comparisons. Although the main demographic characteristics were broadly comparable across groups and recruitment sites, the limited sample size precluded a detailed assessment of recruitment-site-related effects, and residual site-related confounding cannot be fully excluded. Similarly, although sex and age were considered where applicable (Suppl. Mat. 4, 23-27), the cohort was not sufficiently powered to examine independent sex- or age-related effects, or their interactions with clinical subgroup status. This is particularly relevant in autoimmune diseases such as RA, where demographic and biological factors may influence inflammatory responses, pain processing and peripheral immune signatures. The potential contribution of relevant comorbid conditions, including fibromyalgia and depressive symptoms, to the observed functional connectivity alterations also cannot be excluded. In addition, the transcriptomic findings require cautious interpretation. Bulk PBMC expression patterns may be influenced by peripheral immune activation and cellular composition, and cannot be regarded as direct surrogates of molecular alterations in the CNS. Moreover, pathways annotated as neuroplastic, synaptic or nervous-system-related in neuronal contexts may have distinct functions in circulating immune cells. As this was an observational study without an independent replication cohort, the transcriptomic and subgroup-level findings should be considered hypothesis-generating and require confirmation in larger, independent populations.

Nevertheless, the present data support the relevance of altered central pain processing, psychological burden and peripheral immune-related molecular signatures in the D2T RA disease state. These observations highlight the need for further studies investigating integrated management strategies that extend beyond immunosuppression alone, including non-pharmacological interventions such as cognitive-behavioral therapy, mindfulness-based stress reduction and physiotherapy in selected patients [54, 97].

Overall, our research supports the relevance of CNS-related alterations in RA, particularly among D2T patients, which is associated with altered functional brain connectivity patterns, and a distinct clinical and psychological profile. These findings provide a basis for future studies examining the underlying mechanisms of the D2T state, including further investigation the link between the peripheral immune processes, central sensitization and psychological burden. Furthermore, our results highlight the need and serve as a foundation for future research on pathomechanisms, biomarkers, novel therapeutic targets, as well as nonpharmacological management strategies.

## Supplementary Information

Supplementary Material 1.

## Data Availability

Processed data information and tables are included in the Supplementary Information. RNA transcriptomic data is deposited in a public repository (https://www.ebi.ac.uk/ena) under the following identifier: PRJEB88406. All other datasets generated and/or analyzed during the current study are available from the corresponding author upon reasonable request.

## Abbreviations

AAL: Automated Anatomical Labeling (atlas)
ACC: Anterior cingulate cortex
ACR: American College of Rheumatology
AG: Angular gyrus
b/tsDMARDs: Biologic/targeted synthetic disease-modifying antirheumatic drugs
BOLD: Blood-oxygen-level dependent
CEN: Central executive network
CHEPS: Contact Heat Evoked Potential Stimulator
CNS: Central nervous system
CONN: CONN functional connectivity toolbox
CRP: C-reactive protein
CRPS: Complex regional pain syndrome
D2T: Difficult-to-treat
DAN: Dorsal attention network
DAS28: Disease Activity Score in 28 joints
DE: Differentially expressed
DMN: Default mode network
EDTA: Ethylenediaminetetraacetic acid
ESR: Erythrocyte sedimentation rate
ETT-TUKEB: Hungarian National Ethics Committee
EULAR: European Alliance of Associations for Rheumatology
fALFF: Fractional amplitude of low-frequency fluctuations
FM: Fibromyalgia
fMRI: Functional magnetic resonance imaging
FP: Frontal pole
GO: Gene Ontology
HC(s): Healthy control(s)
HI-HP: High inflammation–high pain
HI-LP: High inflammation–low pain
ICC: Intrinsic connectivity
IHC: Interhemispheric correlation
IFN: Interferon
IPA: Ingenuity Pathway Analysis
ITG: Inferior temporal gyrus
LCOR: Local correlation
LG: Lingual gyrus
LI-HP: Low inflammation–high pain
LI-LP: Low inflammation–low pain
LOC: Lateral occipital cortex
MDD: Major depressive disorder
MFG: Middle frontal gyrus
MIST: Multi-resolution parcellation atlas
MPFC: Medial prefrontal cortex
mRNA: Messenger RNA
MS: Morning stiffness
MTG: Middle temporal gyrus
NRG1: Neuregulin 1
OFC: Orbitofrontal cortex
OP: Occipital pole
PBMC: Peripheral blood mononuclear cell(s)
PCC: Posterior cingulate cortex
PCG: Postcentral gyrus
PGA: Patient global assessment
PGH: Patient global health
PSQ: Pain Sensitivity Questionnaire
PTGA: Physician global assessment
RA: Rheumatoid arthritis
ROI: Region of interest
R-PAS: Rorschach Performance Assessment System
RPI: Rorschach Probability Index
rs-fMRI: Resting-state functional magnetic resonance imaging
SFG: Superior frontal gyrus
SMG: Supramarginal gyrus
SMN: Sensorimotor network
SN: Salience network
SPM: Statistical Parametric Mapping
TP: Temporal pole
VAS: Visual analogue scale
WoC: Ways of Coping
