# Supplementary material for: Characteristic central nervous system fingerprints and related molecular signatures in difficult-to-treat rheumatoid arthritis
Source: J Neuroinflammation. 2026 Aug 3;23:358. doi: 10.1186/s12974-026-03992-z (PMC13721227; doi:10.1186/s12974-026-03992-z)
Supplement: Supplementary file 1 [file 12974_2026_3992_MOESM1_ESM.pdf]

Deciphering characteristic central nervous system alterations and related molecular signatures in difficult-to-treat rheumatoid arthritis

Supplementary Materials

Table of contents

Supplementary Materials 1 – Study design protocol and detailed methodology. ... 2
Supplementary Materials 2 – Exploratory sex-related sensitivity analysis of psychological assessment measures ... 32
Supplementary Materials 3 – Post-hoc power sensitivity analysis of the transcriptomic comparisons ... 38
Supplementary Materials 4 – Sensitivity analysis assessing the influence of sex on transcriptomic findings ... 39
Supplementary Materials 5 – Clinical feature details (data for Fig.2) ... 40
Supplementary Materials 6 – Clinical feature details (stratified by study group and recruitment site) ... 41
Supplementary Materials 7 – Results of baseline fMRI alterations (data for Fig. 3 a-e, g-i, k) ... 44
Supplementary Materials 8 – Results of pairwise analysis for baseline fMRI measurements (data for Fig. 3f,j,l) ... 46
Supplementary Materials 9 – Results of fMRI measurements summarizing the effect of acute pain on brain connectivity (data for Fig. 4) ... 47
Supplementary Materials 10 – Results of fMRI measurements summarizing the relations of the Rorschach probability index (RPI) and brain connectivity at baseline (a) and after acute pain stimulation (b) (data for Fig.5.d-g) ... 48
Supplementary Materials 11 – Results of fMRI measurements summarizing the relation of NRG1 expression with baseline resting-state functional connectivity patterns (data for Fig. 6l) ... 49
Supplementary Materials 12 – Comparison based on Rorschach indicators between D2T RA and non-D2T RA patients ... 50
Supplementary Materials 13 – Description of the indicators that make up the Rorschach Probability Index (RPI) ... 53
Supplementary Materials 14 – List of differentially expressed (DE) genes ... 56
Supplementary Materials 15 – Summary of enrichment analysis of DE genes listing the top 25 processes or pathways based on different databases (Gene Ontology and Reactome) ... 74
Supplementary Materials 16 – List of differentially expressed (DE) genes ... 77
Supplementary Materials 17 – List of differentially expressed (DE) genes ... 82
Supplementary Materials 18 – Summary of enrichment analysis of DE genes listing the top 25 processes or pathways based on different databases (Gene Ontology and Reactome) ... 100
Supplementary Materials 19 – List of differentially expressed (DE) genes ... 104
Supplementary Materials 20 – Summary of enrichment analysis of DE genes listing the top 25 processes or pathways based on different databases (Gene Ontology and Reactome) ... 115
Supplementary Materials 21 – List of differentially expressed (DE) genes ... 118
Supplementary Materials 22 – Summary of enrichment analysis of DE genes listing the top 25 processes or pathways based on different databases (Gene Ontology and Reactome) ... 122
Supplementary Materials 23 – List of differentially expressed (DE) genes (sex-adjusted) ... 125
Supplementary Materials 24 – List of differentially expressed (DE) genes (sex-adjusted) ... 139
Supplementary Materials 25 – List of differentially expressed (DE) genes (sex-adjusted) ... 158
Supplementary Materials 26 – List of differentially expressed (DE) genes (sex-adjusted) ... 165
Supplementary Materials 27 – List of differentially expressed (DE) genes (sex-adjusted) ... 177

Supplementary Materials 1 – Study design protocol and detailed methodology

Study design

The study consisted of four major parts for all participants: (1) a clinical examination, (2) functional magnetic resonance imaging (fMRI) examinations, (3) a psychological evaluation and (4) peripheral blood mononuclear cell (PBMC) transcriptomics.

Participant selection

The electronic databases of the Department of Rheumatology and Immunology at the University of Pécs (Pécs center) and the Hospital of the Hospitaller Brothers in Budapest (Budapest center) were screened for rheumatoid arthritis (RA) patients. All patients identified as eligible during pre-screening were contacted by telephone, provided with detailed information, and those interested attended an in-person visit where consent and initial screening were obtained prior to any examinations. The study components (clinical examination, psychological assessment, blood collection, and fMRI) were scheduled on separate days within a 3-week period, adapted to patient needs. Healthy controls (HC) were recruited from the university and hospital community and were age- and sex-matched to the patient cohort. RA patients were categorized into difficult-to-treat (D2T) (n=31) and non-D2T (n=18) subgroups based on the EULAR definition for D2T RA[1]. Non-D2T patients received at least two different biological or targeted synthetic disease modifying antirheumatic drug (b/tsDMARDs) with a disease duration comparable to the D2T group, but were not classified as D2T based on their overall clinical status and well-being. D2T RA patients were further classified into subgroups based on their inflammatory and pain parameters, resulting in three subgroups: (1) high inflammation and high pain (n=10), (2) high inflammation and low pain (n=11), and (3) low inflammation and high pain (n=10). High inflammation was defined based on C-reactive protein (CRP) and erythrocyte sedimentation rate (ESR) levels, with CRP above 5 mg/L and/or ESR above 25 mm/hr considered as high. Pain was assessed by visual analog scale (VAS) based on the average pain over the last four weeks, with values above 50 mm considered high. A group of age- and sex-matched HCs (n=32) was included in the study to compare with the RA patients. All participants provided written informed consent. The study was conducted in compliance with the Declaration of Helsinki and local country regulations. The protocol was approved by the institutional review board and the relevant independent ethics committee, the Hungarian National Ethics Committee (ETT-TUKEB; approval number: BMEÜ/3788-1/2022/EKU).

References

[1] Nagy G, Roodenrijs NM, Welsing PM, et al EULAR definition of difficult-to-treat rheumatoid arthritis Annals of the Rheumatic Diseases 2021;80:31-35.

Procedures and data analysis

1. Clinical examinations

Participants underwent a comprehensive clinical evaluation, including a detailed medical interview, physical examination, and joint ultrasound when indicated. Pain characteristics were further investigated using the PainDetect Questionnaire [1] and 2016 ACR fibromyalgia criteria [2]. Laboratory tests included inflammatory markers, metabolic and organ function parameters. Detailed protocol of the obtained questions and examinations is listed in *Appendix 1.* The comparisons between two groups were conducted using a two-sample t-test and a non-parametric Mann-Whitney test, while comparisons involving more than two groups employed ANOVA or the Kruskal-Wallis test.

References

[1]. Freynhagen R, Baron R, Gockel U, Tölle TR. painDETECT: a new screening questionnaire to identify neuropathic components in patients with back pain. Curr Med Res Opin. 2006 Oct;22(10):1911-20.

[2]. Wolfe, F., et al. 2016 Revisions to the 2010/2011 fibromyalgia diagnostic criteria. Semin Arthritis Rheum 46, 319-329 (2016).

2. Functional magnetic resonance imaging experiments

Subjects were scanned using a 3T MRI scanner (MAGNETOM Prisma^{Fit}, Siemens-Healthineers, Erlangen, Germany) using a 64-ch head-neck coil. The MRI study protocol included the following sequences: 3D T1 magnetization-prepared rapid acquisition with gradient echo (MPRAGE), 2D FID-EPI resting-state functional MRI (rs-fMRI) with field mapping to reduce image distortions due to B0 inhomogeneities.

Evaluation was carried out by using CONN (21.a)[1, 2] and SPM (12.7771) [3].

Preprocessing: Functional and anatomical data were preprocessed using a flexible preprocessing pipeline[4] including creation of voxel-displacement maps, realignment with susceptibility distortion correction using fieldmaps, slice timing correction, outlier detection, indirect segmentation and MNI-space normalization, and smoothing. Functional data were realigned using SPM realign & unwarp procedure[5] integrating fieldmaps for susceptibility distortion correction, where all scans were coregistered to a reference image (first scan of the first session) using a least squares approach and a 6 parameter (rigid body) transformation, and resampled using b-spline interpolation[6] to simultaneously correct for motion, magnetic susceptibility geometric distortions, and their interaction. Temporal misalignment between different slices of the functional data (acquired in interleaved Siemens order) was corrected following SPM slice-timing correction (STC) procedure[7,8], using sinc temporal interpolation to resample each slice BOLD timeseries to a common mid-acquisition time. Potential outlier scans were identified using ART[9] as acquisitions with framewise displacement above 0.9 mm or global BOLD signal changes above 5 standard deviations[10,11], and a reference BOLD image was computed for each subject by averaging all scans excluding outliers. Functional and anatomical data were coregistered and normalized into standard MNI space, segmented into grey matter, white matter, and CSF tissue classes, and resampled to 2 mm isotropic voxels following an indirect normalization procedure[11,12] using SPM unified segmentation and normalization algorithm[13,14] with the default IXI-549 tissue probability map template. Last, functional data were smoothed using spatial convolution with a Gaussian kernel of 6 mm full width half maximum (FWHM).

Denoising: In addition, functional data were denoised using a standard denoising pipeline[15] including the regression of potential confounding effects characterized by white matter timeseries (5 CompCor noise components), CSF timeseries (5 CompCor noise components), motion linear and quadratic parameters and their first order derivatives (24 factors)[16], outlier scans (below 27 factors)[10], session and task effects and their first order derivatives (6 factors), and linear trends (2 factors) within each functional run, followed by bandpass frequency filtering of the BOLD timeseries[17] between 0.008 Hz and 0.09 Hz. CompCor[18,19] noise components within white matter and CSF were estimated by computing the average BOLD signal as well as the largest principal components orthogonal to the BOLD average, motion parameters, and outlier scans within each subject's eroded segmentation masks. From the number of noise terms included in this denoising strategy, the effective degrees of freedom of the BOLD signal after denoising were estimated to range from 190.4 to 204.7 (average 203.6) across all subjects[11].

First-level analyses

Seed-based connectivity maps (SBC) and ROI-to-ROI connectivity matrices (RRC) were estimated characterizing the patterns of functional connectivity between ROIs (RRC) or ROIs and target voxels (SBC). Functional connectivity strength was represented by Fisher-transformed bivariate correlation coefficients from a weighted general linear model (weighted-GLM[20]), defined separately for each pair of seed and target areas, modeling the association between their BOLD signal timeseries. Individual scans were weighted by a boxcar signal characterizing each individual task or experimental condition convolved with an SPM canonical hemodynamic response function and rectified.

Functional connectivity multivariate pattern analyses (fc-MVPA[21]) were performed to estimate the first 4 eigenpatterns characterizing the principal axes of heterogeneity in functional connectivity across subjects and conditions. From these eigenpatterns, 4 associated eigenpattern-score images were derived for each individual subject and condition characterizing their brain-wide functional connectome state. Eigenpatterns and eigenpattern-scores were computed separately for each individual seed voxel as the left- and right- singular vectors, respectively, from a singular value decomposition (group-level SVD) of the matrix of functional connectivity values between this seed voxel and the rest of the brain (a matrix with one row per target voxel, and one column per subject and condition). Individual functional connectivity values were computed from the matrices of bivariate correlation coefficients between the BOLD timeseries from each pair of voxels, estimated using a singular value decomposition of the z-score normalized BOLD signal (subject-level SVD) with 64 components separately for each subject and condition[1].

Intrinsic Connectivity maps (ICC) characterizing network centrality at each voxel were estimated as the root mean square (RMS) of all short- and long- range connections between a voxel and the rest of the brain[22]. Connections were computed from the matrix of bivariate correlation coefficients between the BOLD timeseries from each pair of voxels, estimated using a singular value decomposition of the z-score normalized BOLD signal (subject-level SVD) with 64 components separately for each subject and condition[1].

Local Correlation maps (LCOR) characterizing local coherence at each voxel were estimated as the weighted average of all short-range connections between a voxel and a 25 mm FWHM Gaussian neighborhood area[23]. Short-range connections were computed from the matrix of bivariate correlation coefficients between the BOLD timeseries from each pair of voxels, estimated using a singular value decomposition of the z-score normalized BOLD signal (subject-level SVD) with 64 components separately for each subject and condition[1].

Interhemispheric correlation maps (IHC) characterizing the strength of homotopic functional connectivity between the two hemispheres were estimated as the Fisher-transformed bivariate correlation coefficient between the BOLD signal at each voxel and at the same anatomical location in the contralateral hemisphere (voxels with the same y&z MNI coordinates and opposite-sign x MNI coordinates)[24,20].

Fractional amplitude of low frequency fluctuations (fALFF) maps characterizing low-frequency BOLD signal variability at each voxel were estimated as the ratio between the root mean square (RMS) of the BOLD signal after denoising and band-pass filtering between 0.008 Hz and 0.09 Hz, divided by the same measure computed before band-pass filtering[25].

Group-level analyses were performed using a General Linear Model (GLM[26]). For each individual voxel a separate GLM was estimated, with first-level connectivity measures at this voxel as dependent variables (one independent sample per subject and one measurement per task or experimental condition, if applicable), and groups or other subject-level identifiers as independent variables. Voxel-level hypotheses were evaluated using multivariate parametric statistics with random-effects across subjects and sample covariance estimation across multiple measurements. Inferences were performed at the level of individual clusters (groups of contiguous voxels). Cluster-level inferences were based on parametric statistics from Gaussian Random Field theory[27,28]. Results were thresholded using a combination of a cluster-forming $p < 0.001$ voxel-level threshold, and a familywise corrected p-FDR < 0.05 cluster-size threshold[29]. For RRC, inferences were performed at the level of individual clusters (groups of similar connections). Cluster-level inferences were based on parametric statistics within- and between- each pair of networks (Functional Network Connectivity[30]), with networks identified using a complete-linkage hierarchical clustering procedure[31] based on ROI-to-ROI anatomical proximity and functional similarity metrics[28]. Results were thresholded using a combination of a $p < 0.05$ connection-level threshold and a familywise corrected p-FDR < 0.05 cluster-level threshold[32].

References

[1] Whitfield-Gabrieli, S., & Nieto-Castanon, A. (2012). Conn: a functional connectivity toolbox for correlated and anticorrelated brain networks. Brain connectivity, 2(3), 125-141.

[2] Nieto-Castanon, A. & Whitfield-Gabrieli, S. (2022). CONN functional connectivity toolbox: RRID SCR_009550, release 22. doi:10.56441/hilbertpress.2246.5840.

[3] Penny, W. D., Friston, K. J., Ashburner, J. T., Kiebel, S. J., & Nichols, T. E. (Eds.). (2011). Statistical parametric mapping: the analysis of functional brain images. Elsevier.

[4] Nieto-Castanon, A. (2020). FMRI minimal preprocessing pipeline. In Handbook of functional connectivity Magnetic Resonance Imaging methods in CONN (pp. 3–16). Hilbert Press.

[5] Andersson, J. L., Hutton, C., Ashburner, J., Turner, R., & Friston, K. J. (2001). Modeling geometric deformations in EPI time series. Neuroimage, 13(5), 903-919.

[6] Friston, K. J., Ashburner, J., Frith, C. D., Poline, J. B., Heather, J. D., & Frackowiak, R. S. (1995). Spatial registration and normalization of images. Human brain mapping, 3(3), 165-189.

[7] Henson, R. N. A., Buechel, C., Josephs, O., & Friston, K. J. (1999). The slice-timing problem in event-related fMRI. NeuroImage, 9, 125.

[8] Sladky, R., Friston, K. J., Tröstl, J., Cunnington, R., Moser, E., & Windischberger, C. (2011). Slice-timing effects and their correction in functional MRI. Neuroimage, 58(2), 588-594.

[9] Whitfield-Gabrieli, S., Nieto-Castanon, A., & Ghosh, S. (2011). Artifact detection tools (ART). Cambridge, MA. Release Version, 7(19), 11.

[10] Power, J. D., Mitra, A., Laumann, T. O., Snyder, A. Z., Schlaggar, B. L., & Petersen, S. E. (2014). Methods to detect, characterize, and remove motion artifact in resting state fMRI. Neuroimage, 84, 320-341.

[11] Nieto-Castanon, A. (submitted). Preparing fMRI Data for Statistical Analysis. In M. Filippi (Ed.). fMRI techniques and protocols. Springer. doi:10.48550/arXiv.2210.13564

[12] Calhoun, V.D., Wager, T.D., Krishnan, A., Rosch, K.S., Seymour, K.E., Nebel, M.B., Mostofsky, S.H., Nyalakanai, P. and Kiehl, K. (2017). The impact of T1 versus EPI spatial normalization templates for fMRI data analyses (Vol. 38, No. 11, pp. 5331-5342).

[13] Ashburner, J., & Friston, K. J. (2005). Unified segmentation. Neuroimage, 26(3), 839-851.

[14] Ashburner, J. (2007). A fast diffeomorphic image registration algorithm. Neuroimage, 38(1), 95-113.

[15] Nieto-Castanon, A. (2020). FMRI denoising pipeline. In Handbook of functional connectivity Magnetic Resonance Imaging methods in CONN (pp. 17–25). Hilbert Press.

[16] Friston, K. J., Williams, S., Howard, R., Frackowiak, R. S., & Turner, R. (1996). Movement-related effects in fMRI time-series. Magnetic resonance in medicine, 35(3), 346-355.

[17] Hallquist, M. N., Hwang, K., & Luna, B. (2013). The nuisance of nuisance regression: spectral misspecification in a common approach to resting-state fMRI preprocessing reintroduces noise and obscures functional connectivity. Neuroimage, 82, 208-225.

[18] Behzadi, Y., Restom, K., Liau, J., & Liu, T. T. (2007). A component based noise correction method (CompCor) for BOLD and perfusion based fMRI. Neuroimage, 37(1), 90-101.

[19] Chai, X. J., Nieto-Castanon, A., Ongur, D., & Whitfield-Gabrieli, S. (2012). Anticorrelations in resting state networks without global signal regression. Neuroimage, 59(2), 1420-1428.

[20] Nieto-Castanon, A. (2020). Functional Connectivity measures. In Handbook of functional connectivity Magnetic Resonance Imaging methods in CONN (pp. 26–62). Hilbert Press.

[21] Nieto-Castanon, A. (2022). Brain-wide connectome inferences using functional connectivity MultiVariate Pattern Analyses (fc-MVPA). PLOS Computational Biology, 18(11), e1010634.

[22] Martuzzi, R., Ramani, R., Qiu, M., Shen, X., Papademetris, X., & Constable, R. T. (2011). A whole-brain voxel based measure of intrinsic connectivity contrast reveals local changes in tissue connectivity with anesthetic without a priori assumptions on thresholds or regions of interest. Neuroimage, 58(4), 1044-1050.

[23] Deshpande, G., LaConte, S., Peltier, S., & Hu, X. (2009). Integrated local correlation: a new measure of local coherence in fMRI data. Human brain mapping, 30(1), 13-23.

[24] Jin, X., Liang, X., & Gong, G. (2020). Functional integration between the two brain hemispheres: evidence from the homotopic functional connectivity under resting state. Frontiers in Neuroscience, 14.

[25] Zou, Q. H., Zhu, C. Z., Yang, Y., Zuo, X. N., Long, X. Y., Cao, Q. J., ... & Zang, Y. F. (2008). An improved approach to detection of amplitude of low-frequency fluctuation (ALFF) for resting-state fMRI: fractional ALFF. Journal of neuroscience methods, 172(1), 137-141.

[26] Nieto-Castanon, A. (2020). General Linear Model. In Handbook of functional connectivity Magnetic Resonance Imaging methods in CONN (pp. 63–82). Hilbert Press.

[27] Worsley, K. J., Marrett, S., Neelin, P., Vandal, A. C., Friston, K. J., & Evans, A. C. (1996). A unified statistical approach for determining significant signals in images of cerebral activation. Human brain mapping, 4(1), 58-73.

[28] Nieto-Castanon, A. (2020). Cluster-level inferences. In Handbook of functional connectivity Magnetic Resonance Imaging methods in CONN (pp. 83–104). Hilbert Press.

[29] Chumbley, J., Worsley, K., Flandin, G., & Friston, K. (2010). Topological FDR for neuroimaging. Neuroimage, 49(4), 3057-3064.

[30] Jafri, M. J., Pearlson, G. D., Stevens, M., & Calhoun, V. D. (2008). A method for functional network connectivity among spatially independent resting-state components in schizophrenia. Neuroimage, 39(4), 1666-1681.

[31] Sørensen, T. (1948). A method of establishing groups of equal amplitude in plant sociology based on similarity of species and its application to analyses of the vegetation on Danish commons. Biologiske Skrifter / Kongelige Danske Videnskabernes Selskab 5: 1-34.

[32] Benjamini, Y., & Hochberg, Y. (1995). Controlling the false discovery rate: a practical and powerful approach to multiple testing. Journal of the Royal statistical society: series B (Methodological), 57(1), 289-300.

3. Psychological examinations

The psychologist approached the patient/HC at the appointed time in a discreet, friendly room of the hospital suitable for psychological examination and, after introducing himself, said that he would first like to explain the privacy statement informed and then, subject to it, ask for written permission to record the conversation, which he would immediately encode and destroy after listening and transcribing. No third party is allowed to link what the person under investigation said with the person's identity.

The psychologist then asked a semi-structured set of questions, which included the following questions (for later comparability and content analysis):

- What do you expect from the research?

- What do you think about why, by what and what finally caused this symptom?

- Can you relate it to something in yourself?

- Specifically (in your life events) and psychologically-subjectively, what was happening at the time (and in the period) when your illness started (moving house, divorce, child leaving home, failures... etc.) How did you react to this? What was the patient concerned about at the time?

- If the pain could speak, and not just hurt, what could you say to him?

- What does this disturbance mean for his life? What has changed radically because of it?

- How do family members and colleagues relate to your illness?

- With whom have you shared your problem? Who knows about it? Has anyone helped you?

- What do you wish for, what would you most like to have? (By asking this question, we are trying to find out what your expectations are of your therapy: it is the treatment you imagine (expect, hope for) based on your own fantasies that promises the greatest effectiveness!)

- What is your fantasy of what your life will be like in 3-5-1 years?

- What do you wish you had 3 wishes?

This was followed by the Rorschach Projective Personality Test, the instructions for which were word for word:

"I would now like to conduct a psychological test. During the test, I will show you pictures and ask you to describe what you see. This test is not about performance; it is not timed, so there's no need to rush. I, the examiner, will hand you the charts and record what you say. The images may remind you of nothing, or they might remind you of several things. I'll let you know if you don't need to say anything more, but it's okay if you don't remember anything. Afterwards, I will read everything back to you, and we will discuss whether I recorded everything accurately and where you saw what you described."

It is important to note that we never say *"What is this?"* as we are asking the person to make an association. If they get stuck, after 60 seconds, we can encourage them by saying, *"What does it look like? What does it remind you of?"*. If he still fails to answer, i.e., fails in the German and Ferenc Mérei marking system, we give the subject another board, and only at the retest, which we call the post-test, do we give them another chance to answer. However, this is no longer included in the numerical evaluation of the test, but only in the qualitative analysis of the person's response after the tension in the test situation has been released. The tables can be rotated, the baseline position is marked with the letter, and the marking system is used to indicate whether a response is given sideways or backwards. At the start and end of the test, the time is recorded, and the number of seconds elapsed before the first tickable answer is given is measured as a table (in your head or by looking at a second hand). If the person hesitates for more than 30 seconds, a "confusion" mark is entered in column 5 when marking.

After the test examination, the psychologist handed over the psychological questionnaire package, which consisted of the following questionnaires (links and references for the tests are summarized in *Appendix 2*):

1. General data (age, sex, residence)
2. Questions concern the process of self-assessment of health status
3. Visual Analogue Scale for pain measurement (VAS)
4. Lifestyle Questions
5. Questions about the use of the healthcare system
6. Questions about negative life events (losses)
7. The issues that are prospective in nature are to be addressed
8. Holmes Rahe Social Readjustment Rating Scale. (SRSS)
9. Illness Intrusiveness Rating Scale (IIRS)
10. Generalized Anxiety Scale (GAD-7)
11. Beck Depression Scale (BDI)
12. WHO General Well-being (WHO-5)
13. Perceived Stress Scale (PSS-4)
14. Connor-Davidson Resilience Scale (CD-RISC)
15. Tampa Scale of Kinesiophobia (TSK)
16. Patient-Reported Outcomes Measurement Information System (PROMIS-PF-4A)
17. Patient Health Questionnaire-15 (PHQ-15)
18. Owestry Disability Index (ODI)
19. Somatic Absorption Scale (SAS)
20. MOS- Social Support Survey (MOS SSS-H)
21. Pain Catastrophizing Scale (PCS)
22. Positive and Negative Affect Scale (PANAS)
23. Cognitive Emotion Regulation Questionnaire (CERQ)
24. Psychological Inflexibility in Pain Scale (PIPS)
25. Pain Sensitivity Questionnaire (PSQ-H)
26. Current Opioid Misuse Measure (COMM)
27. Somatosensory Amplification Scale (SSAS)
28. Health-Specific Locus-of-Control Beliefs (HLC)
29. Anger Expression Scale (AES)
30. Ways of Coping Questionnaire (WOC)
31. Young Schema Questionnaire (SQ)
32. Bem Sex Role Inventory (BSRI)
33. Short Form of McGill Pain Questionnaire (SF-MPQ)
34. Chronic Disability Index of Waddell and Main for Patients with Low Back Pain (CDI)

This was to be given to the subject by the treating physician after completion. If this was not possible, a stamped reply envelope was given or the psychologist went back to the institute to collect the sealed envelope, which the patient left with the tests inside.

The tests did not contain the name of the patient/healthy person, but a code number created by the main investigator before the study. They also did not know whether a patient was difficult-to-treat or non-difficult-to-treat; however, some of the healthy volunteers could be identified due to previous acquaintances, since they were included from the medical centers involved in the study. The statistical processing was done by code only.

After typing the audio material of the interview and anamnesis, the psychologist coded the text (which he had received beforehand) and it was stored in this way. The audio material was deleted. The text material was coded for content analysis by the clinical psychologists who did not know the study participants, i.e. when forming professional opinions, the clinical psychologist only knew the male/female, education and age of the person. In the case of healthy persons, questions about illness were obviously without reason.

The coded, typed version of the Rorschach test answers was marked by 3 independent persons using the Hungarian marking system of Ferenc Mérei [1], and the calculation of the indicators was analysed and calculated by the Rorschach software developed by Péter Bánsági, which is also licensed in Hungary and the analysts were also "blind" to the person being analysed except for their age, gender and education status.

For quantitative evaluation, the comparison of the 2 groups (D2T vs. non-D2T or RA vs. HC), both a two-sample t-test and a non-parametric Mann-Whitney test were used to compare the groups. In the comparisons, not only the p-value for the tests (we choose to use a significance level of 0.05), but also the Cohen's d effect size is used as the effect size. For the effect size above an absolute value of 0.5, we can already speak of a medium effect size - and this is what we have taken as a basis [2]

References

[1] Mérei F. The Rorschach-test: Medicina; 2002.

[2] Vargha, A. Mathematical Statistics, (Pólya Kiadó, Budapest, 2015).

4. Trancriptomic examinations

Blood collection: Peripheral blood collection took place in the morning hours (before 11 am) after fasting. One portion of the blood was used for diagnostic purposes. Additional blood was drawn for further experiments detailed below. Samples were transported to the lab with appropriate cooling (4 °C), and processing steps started within one hour at the latest.

For the transcriptomic measurements, peripheral blood was collected in an EDTA (ethylenediaminetetraacetic acid) containing tube (Vacuette, Greiner Bio-One, Kremsmünster, Austria). PBMCs were isolated by density gradient centrifugation using Ficoll Histopaque-1077 (Sigma-Aldrich, Merck KGaA, Darmstadt, Germany). 7 ml of peripheral blood diluted with 3 ml physiological saline solution were mixed in a sterile centrifuge tube. 10 ml of diluted blood was layered on top of 5 ml Ficoll solution, and samples were centrifuged 400×g for 20 minutes at 21°C. with brake and acceleration at the lowest setting. After centrifugation, the PBMC layer was transferred into a new centrifuge tube, suspended with 15 mL Hanks' Balanced Salt Solution (HBSS, Gibco, Life Technologies Corporation, Grand Island, New York, US), and centrifuged for 10 min at 300×g, 21 °C. The supernatant was removed, and the pellet was resuspended in 10 mL HBSS solution, followed by centrifugation (10 min, 300×g, 21 °C). After the removal of the supernatant, cells were resuspended with 1 mL of TRI Reagent (Zymo Research, Irvine, CA, USA) and stored at − 80 °C in Eppendorf tubes until the mRNA expression investigations.

RNA isolation, sequencing and data analysis: The purification of total RNA was carried out as previously described according to the TRI Reagent manufacturer's protocol (Molecular Research Center, Inc., Cincinnati, OH, USA) until the acquisition of the aqueous phase [1]. In summary, blood samples were homogenized in 1 ml of TRI Reagent, followed by the addition of 200 µl of bromo-chloro-propane (Sigma-Aldrich, Saint Louis, USA). The purification of RNA from the aqueous phase was then undertaken using the Direct-zol RNA MiniPrep kit (Cat. No. R2052; Zymo Research, Irvine, CA, USA), in accordance with the manufacturer's protocol. Briefly, 400 µl of the aqueous phase was mixed with 400 µl of absolute ethanol. The mixture was then loaded onto the column, washed, and the RNA was eluted in 50 µl of RNase-free water. The quantity and purity of the extracted RNA were subsequently assessed using a Nanodrop ND-1000 Spectrophotometer V3.5 (Nano-Drop Technologies, Inc., Wilmington, DE, USA).

RNA libraries were generated using the QuantSeq 3' mRNA-Seq Library Prep Kit FWD for Illumina (Lexogen, Vienna, Austria). Briefly, 100 ng of total RNA was used as input for first strand cDNA generation using oligodT primer followed by RNA removing. Thereafter, the second strand synthesis is initiated by random priming and

the products were purified with magnetic beads. Finally, the libraries were amplified and barcoded using PCR. All libraries were assessed on the Agilent 4200 TapeStation (Agilent Technologies, Palo Alto, CA, USA) to examine if adapter dimers formed during PCR. Illumina sequencing was performed on the NovaSeq 6000 instrument (Illumina, San Diego, CA, USA) with 1 × 75 run configuration.

Binary Base Call (BCL) files generated by the sequencing instrument were base called, demultiplexed and translated into FASTQ files using bcl2fastq v2.20.0.422 software (Illumina Inc.). Reads were subsequently filtered for a minimum length of 40bp and quality-trimmed to Q30 using the Phred algorithm with BBDuk from the BBTools suite v38.86 [2]. The processed reads were aligned against the human reference genome (GRCh37 Ensembl release) with STAR v2.7.6a [3]. The number of reads aligned within each gene was counted using HTSeq Python library v0.11.1[4]. Gene count data were normalized using the trimmed mean of M values (TMM) normalization method of the edgeR R/Bioconductor package (v3.28, R v3.6.0, Bioconductor v3.9). Data were further log transformed using the voom approach for statistical evaluation in the limma package [5]. Fold change values between the compared groups and moderated t-test p-values were calculated by the limma package. Multiple hypothesis testing correction was applied during analysis, although not all results reached FDR significance. Given the exploratory nature of the study, findings with a fold change ≥1.2 and raw p-value < 0.05 were considered indicative of potential biological relevance and served as a basis for hypothesis generation, interpreted with appropriate caution. Normalized counts in exploratory data analysis and visualization were represented as transcripts per million (TPM) values. Functional analysis (annotations of genes) was prepared using the Gene Ontology (GO) and Reactome databases. Detection of functional enrichment was performed in the differentially expressed gene list (DE list enrichment: Fisher's exact test for GO, hypergeometric test for Reactome) applying the topGO v2.37.0, ReactomePA v1.30.0, gage v2.36.0 packages. Ingenuity Pathway Analysis (IPA) software (QIAGEN, Venlo, Netherlands) version 111725566 was used to run a core analysis on differentially expressed genes to discover implicated canonical pathways, regulatory networks, and upstream regulators.

References

[1] Aczél T, Körtési T, Kun J, et al. Identification of disease- and headache-specific mediators and pathways in migraine using blood transcriptomic and metabolomic analysis. J Headache Pain 2021; 22(1): 117.

[2] Bushnell, B., Rood, J. & Singer, E. BBMerge – Accurate paired shotgun read merging via overlap. PLOS ONE 12, e0185056 (2017).

[3] Dobin, A., et al. STAR: ultrafast universal RNA-seq aligner. Bioinformatics 29, 15-21 (2012).

[4] Anders, S., Pyl, P.T. & Huber, W. HTSeq—a Python framework to work with high-throughput sequencing data. Bioinformatics 31, 166-169 (2014).

[5] Ritchie, M.E., et al. limma powers differential expression analyses for RNA-sequencing and microarray studies. Nucleic Acids Research 43, e47-e47 (2015).

Appendix 1. Protocol of the clinical examinations

Country:
City:
Hospital:
Doctor:
Blood sample code:
Date of blood sampling:

1. Patient data

Insurance number: ………………………………

Name: ……………………………………………..

Date of birth: ……………………………………..

Contact number: …………………………………

Gender: Female / Male

Ethnicity/Race: White / Black / Indian / Asian / Other: …….

Date of questioning: ………………………………(year/month/day)

2. Details from the medical history

2.1 Lifestyle

Smoking: yes / no
if yes: amount (cigarettes/day): ………………………..
For how many years?…………………………
if not:
Did you smoke earlier? yes / no
if yes: amount (pcs/occasion): …………………………………
For how many years? ………………………………………
How long ago did you stop smoking? ……………………………….

Alcohol consumption: yes / no
if yes: frequency: occasionally/monthly/weekly/daily
amount (g/day):…………………………………
since when? (years):………………………….
Alcohol consumption in the last 2 weeks: …………………..
if not:
Did you drink alcohol earlier? yes / no
if yes: frequency: occasionally/monthly/weekly/daily
amount (g/occasion):…………………………………
For how many years?………………………………………
How long ago did you stop drinking alcohol?.........................

Guide for estimation of the amount:
1 dl beer (4.5 vol. %) = ~3.5 g alcohol
1 dl wine (12.5 vol. %) = ~10 g alcohol
1 dl hard drink (50 vol. %) = ~40 g alcohol

Drug abuse: yes / no *Prescribed medication should not be included here.*
if yes: type of drug: …………………………………….
amount: ………………………………………….
for how many years:……………………………

Diet: yes / no
if yes: diabetic / gluten-free / vegetarian / paleolit / lactose-free / other:………… (többször választható)

Physical activity: none / occasionally / regularly / intensely*

if ocassionally / regularly / intensely:

Types of exercise: house work / work in the garden / walking / running / jogging / swimming / aerobic / dancing / cycling / weight lifting / other:………… (többször választható)

** None: no exercise or exercise for < 3 hours/week for < 2 years*
Occasionally: < 3 hours/week for >= 2 years
Regularly: > 3 hours/week for >= 2 years
Intensely: > 7 hours/week for>= 2 years

Job: Does (Did, if pensioner) it involve physical activity?
No / Yes, walking. / Yes, weight lifting. / Yes, other hard work. (többször választható)

What is the highest grade or level of school you have completed or the highest degree you have received?

never attended / kindergarten only / 1st Grade / 2nd Grade / 3rd Grade / 4th Grade / 5th Grade / 6th Grade / 7th Grade / 8th Grade / 9th Grade / 10th Grade / 11th Grade / 12th Grade, no diploma / High school graduate / Some college, no degree / Ged or equivalent / Associate degree: occupational, technical or vocational program / Associate degree: academic program / Bachelor's Degree (example: Ba, Ab, Bs, Bba) / Master's Degree (example: Ma, Ms, Meng, Med, Mba) / Professional School Degree (example: Phd, Edd) / Refused to answer / don't know (többször választható)

2.2 Comorbidities

Allergies: yes / no

if yes: specify: inhalative / other nutrition / drug / eczema / other

Diabetes mellitus: yes/no

if yes: type I. / type II / type III. / MODY

date of diagnosis (date: year)……………………………….

Hypertension: yes / no

if yes: since when (year):…………………………………...

Cancerous disease: yes / no / no data

if yes: **type of tumor:** brain / thyroid / lung / breast / oral cavity / esophagus /stomach / colon / pancreas / liver / prostate / kidney / bladder / ovary / other

Thromboembolic events in history: yes / no

if yes: deep vein thrombosis: yes / no
if yes: date:…………….(year/month/day) (többször hozzáadható)
pulmonary embolism: yes / no
if yes: date:…………….(year/month/day) (többször hozzáadható)
repeated miscarriages or stillbirths: yes / no
if yes: date:…………….(year/month/day) (többször hozzáadható)
stroke: yes / no
if yes: date:…………….(year/month/day) (többször hozzáadható)
myocardial infarction: yes / no
if yes: date:…………….(year/month/day) (többször hozzáadható)

Autoimmunity in history: yes /no
if yes: systemic / organ specific
if systemic: Rheumatoid arthritis / Systemic lupus erythematosus / Scleroderma / Sjögren's Syndrome / Antiphospholipid syndrome / Idiopathic inflammatory myopathies / Vasculitis / Other
if Rheumatoid arthritis: date of diagnosis: ……..(year/month/day)

if Systemic lupus erythematosus:date of diagnosis:…..(year/month/day)
if Scleroderma: date of diagnosis: ……..(year/month/day)
if Sjögren's Syndrome: date of diagnosis: ……..(year/month/day)
if Antiphospholipid syndrome: date of diagnosis:....(year/month/day)
if Idiopathic inflammatory myopathies: date of diagnosis:......(year/month/day)
if Vasculitis: date of diagnosis: ……..(year/month/day)
if Other:please specify: …… date of diagnosis: ……..(year/month/day)

if organ specific: Hashimoto's thyroiditis / Graves' disease / Addison's disease / Autoimmune hepatitis / Primary biliary cholangitis / Primary sclerosing cholangitis / Multi-glandular diseases / Autoimmune pancreatitis / Coeliac disease / Autoimmune hemolytic anemia / Autoimmune thrombocytopenic purpura / Myasthenia gravis / Multiple sclerosis / Inflammatory neuropathies / Other

if Hashimoto's thyroiditis: date of diagnosis: ……..(year/month/day)
if Graves' disease: date of diagnosis: ……..(year/month/day)
if Addison's disease: date of diagnosis: ……..(year/month/day)
if Autoimmune hepatitis: date of diagnosis: ……..(year/month/day)
if Primary biliary cholangitis: date of diagnosis: ……..(year/month/day)
if Primary sclerosing cholangitis:date of diagnosis: ….(year/month/day)
if Multi-glandular diseases: date of diagnosis: ……..(year/month/day)
if Autoimmune pancreatitis: date of diagnosis: ……..(year/month/day)
if Coeliac disease: date of diagnosis: ……..(year/month/day)
if Autoimmune hemolytic anemia: date of diagnosis:...(year/month/day)
if Autoimmune thrombocytopenic purpura:date of diagnosis:....(year/month/day)
if Myasthenia gravis: date of diagnosis: ……..(year/month/day)
if Multiple sclerosis: date of diagnosis: ……..(year/month/day)
if Inflammatory neuropathies: date of diagnosis: ….(year/month/day)
if Other: please specify: ……………
date of diagnosis:.......(year/month/day)

Ischemic heart disease*: yes / no
if yes: date of diagnosis: ……………….(year/month/day)

** Definition: previews myocardial infarct or PTCA or CABG or positive CT-angiography or positive coronary angiography*

Congestive heart disease*: yes / no

** Definition: using of loop diuretics*

Malabsorption: yes / no

Malabsorption definition: decreased serum protein / nutritional supplement / long-term weight loss.

Hiperalimentation required: yes / no

Asthma: yes / no
if yes: date of start of treatment: …………….(year/month/day)

COPD: yes / no
if yes: date of start of treatment: …………….(year/month/day)

Interstitial lung disease: yes / no *
if yes: date of diagnosis: ……………….(year/month/day)

** Diagnosis is based on the combination of compatible clinical features, pulmonary function testing (eg, restrictive changes and a gas transfer abnormality), and high resolution computed tomography (HRCT) findings (eg, reticular, ground glass, or consolidative changes), and also exclusion of other processes, such as infection, drug-induced pulmonary toxicity and malignancy.*

Gout in history: yes / no
if yes: date of first attack: …………(year/month/day)

Spondyloarthritis in history: yes / no
if yes: axial spondyloarthritis / peripheral spondyloarthritis / reactive arthritis / psoriatic arthritis / enteropathic arthritis / endogenous uveitis (többszörösen választható)
date of diagnosis: …………(year/month/day)

Long lasting / recurrent fever of unknown origin: yes / no
if yes: date of start of the first episode: …………(year/month/day)
date of last episode: …………(year/month/day)

Other internal organ disease: yes / no
if yes: heart / lung / gastrointestinal / liver / renal / hematologic / thyroid
please specify: …………….

Osteoporosis (DEXA): yes / no

if yes: date of diagnosis: ………….(year/month/day)
any osteoporotic fracture: yes / no
if yes date of the first fracture:………….(year/month/day)
Number of the osteoporotic fractures:…………..

Gum sensitivity, pain or bleeding: yes / no

Other: yes / no
if yes: please specify:………………….

2.3 Family history

Thromboembolic events in family history
if yes: relationship to patient:*..........................
deep vein thrombosis / pulmonary embolism / stroke / myocardial infarction

Autoimmunity in family: yes /no
if yes: relationship to patient*:…………………………………….
systemic / organ specific
if systemic: Rheumatoid arthritis / Systemic lupus erythematosus / Scleroderma / Sjögren's Syndrome / The antiphospholipid syndrome / Idiopathic inflammatory myopathies / Vasculitis / Other
if Other: please specify: ……………
if organ specific: Hashimoto's thyroiditis / Graves' disease / Addison's disease / Autoimmune hepatitis / Primary biliary cholangitis / Primary sclerosing cholangitis / Multi-glandular diseases / Autoimmune pancreatitis / Coeliac disease / Autoimmune hemolytic anemia / Autoimmune thrombocytopenic purpura / Myasthenia gravis / Multiple sclerosis / inflammatory neuropathies / Other
if Other: please specify: ……………

Spondyloarthritis in family history: yes / no

if yes: relationship to patient*: …………………………………….

Diabetes mellitus in family history: yes/no

if yes: type I. / type II / type III. / MODY

relationship to patient*: …………………………………….

Hypertension in family history: yes / no

if yes: relationship to patient*: …………………………………….

Malignancy in family history: yes / no

if yes: please specify:…………………………

relationship to patient*: …………………………………….

(többször hozzáadható)

Asthma in family history: yes / no
if yes: relationship to patient*: …………………………………….

COPD in family history: yes / no
if yes: relationship to patient*:…………………………………….

Other: yes / no
if yes: please specify:………………….
relationship to patient*:…………………………………….

* **relationship:** father / mother / sibling / child / paternal grandfather / paternal grandmother / maternal grandmother / maternal grandfather / paternal cousin / maternal cousin / father sibling (uncle, aunt) / mother sibling (uncle, aunt) / siblings child (nephew, niece) / grandchild / paternal grandfathers sibling / paternal grandmothers sibling / maternal grandfathers sibling / maternal grandmothers sibling / other blood relation / spouse (husband, wife, other not blood relation) (többszörösen választható)

2.4 Medication in history

Prednisolone /methylprednisolone: yes / no

if yes: dose (mg/day): …………

start date: ………….. (year/month/day)

stop date: ………….. (year/month/day)

if no: previously used? yes / no

if yes: why was it stopped? the medication became unnecessary / unbearable side-effects / not effective enough / allergy / other

NSAID: yes / no

if yes: aspirin / ibuprofen / dexketoprofen / naproxen / indomethacin / diclofenac / aceclofenac / piroxicam / meloxicam / mefenamic acid / niflumic acid / aminophenazon / nimesulid / celecoxib /etoricoxib / other (többször választható)

if aspirin: occasionally / regularly
daily / weekly
if daily: dose/ day:……..mg
if weekly: dose/week:……..mg

if ibuprofen: occasionally / regularly
daily / weekly
if daily: dose/ day:……..mg
if weekly: dose/week:……..mg

if dexketoprofen: occasionally / regularly
daily / weekly
if daily: dose/ day:…….. mg
if weekly: dose/week:…….. mg

if naproxen: occasionally / regularly
daily / weekly
if daily: dose/ day:…….. mg
if weekly: dose/week:…….. mg

if indomethacin: occasionally / regularly
daily / weekly
if daily: dose/ day:…….. mg
if weekly: dose/week:…….. mg

if diclofenac: occasionally / regularly
daily / weekly
if daily: dose/ day:…….. mg
if weekly: dose/week:…….. mg

if aceclofenac: occasionally / regularly
daily / weekly
if daily: dose/ day:…….. mg
if weekly: dose/week:…….. mg

if piroxicam: occasionally / regularly
daily / weekly
if daily: dose/ day:…….. mg
if weekly: dose/week:…….. mg

if meloxicam: occasionally / regularly
daily / weekly
if daily: dose/ day:…….. mg
if weekly: dose/week:…….. mg

if mefenamic acid: occasionally / regularly
daily / weekly
if daily: dose/ day:…….. mg
if weekly: dose/week:…….. mg

if niflumic acid: occasionally / regularly
daily / weekly
if daily: dose/ day:…….. mg
if weekly: dose/week:…….. mg

if nimesulid: occasionally / regularly
daily / weekly

if daily: dose/ day:…….. mg
if weekly: dose/week:……..mg
if celecoxib: occasionally / regularly
daily / weekly
if daily: dose/ day:…….. mg
if weekly: dose/week:…….. mg
if etoricoxib: occasionally / regularly
daily / weekly
if daily: dose/ day:…….. mg
if weekly: dose/week:…….. mg
if other: please specify:……………….
daily / weekly
if daily: dose/ day:…….. mg
if weekly: dose/week:…….. mg (többször hozzáadható)
if no: previously used? yes / no
if yes: why was it stopped? the medication became unnecessary / unbearable side-effects / not effective enough / allergy / other

Paracetamol: yes / no
if yes: occasionally / regularly
daily: yes / no
weekly : yes / no
if no: previously used? yes / no
if yes: why was it stopped? the medication became unnecessary / unbearable side-effects / not effective enough / allergy /other

Tramadol: yes / no
if yes: occasionally / regularly
daily: yes / no
weekly : yes / no
if no: previously used? yes / no
if yes: why was it stopped? the medication became unnecessary / unbearable side-effects / not effective enough / allergy / other

Methotrexate: yes / no
if yes: dose (mg/week): …………
start date: ………….. (year/month/day)
stop date: ………….. (year/month/day)
route of administration: oral / subcutaneous / intravenous / intramuscular
if no: previously used? yes / no
if yes: why was it stopped? the medication became unnecessary / unbearable side-effects / not effective enough / allergy /other

Sulfasalazine: yes / no
if yes: dose (g/day): …………
start date: ………….. (year/month/day)
stop date: ………….. (year/month/day)
route of administration: oral / subcutaneous / intravenous / intramuscular
if no: previously used? yes / no
if yes: why was it stopped? the medication became unnecessary / unbearable side-effects / not effective enough / allergy / other

Leflunomide: yes / no
if yes: dose (g/day): …………
start date: ………….. (year/month/day)
stop date: ………….. (year/month/day)
route of administration: oral / subcutaneous / intravenous / intramuscular
if no: previously used? yes / no
if yes: why was it stopped? the medication became unnecessary / unbearable side-effects / not effective enough / allergy /other

Cyclophosphamide: yes / no
if yes: dose (mg/administration): …………
start date: ………….. (year/month/day)

stop date: ………….. (year/month/day)
route of administration: oral / subcutaneous / intravenous / intramuscular
if no: previously used? yes / no
if yes: why was it stopped? the medication became unnecessary / unbearable side-effects / not effective enough / allergy /other

Cyclosporin A: yes / no
if yes: dose (mg/day): ………….
start date: ………….. (year/month/day)
stop date: ………….. (year/month/day)
route of administration: oral / subcutaneous / intravenous / intramuscular
if no: previously used? yes / no
if yes: why was it stopped? the medication became unnecessary / unbearable side-effects / not effective enough / allergy /other

Azathioprine: yes / no
if yes: dose (mg/day): ………….
start date: ………….. (year/month/day)
stop date: ………….. (year/month/day)
route of administration: oral / subcutaneous / intravenous / intramuscular
if no: previously used? yes / no
if yes: why was it stopped? the medication became unnecessary / unbearable side-effects / not effective enough / allergy / other

Antimalarials (Chloroquine, hydroxychloroquine): yes / no
if yes: dose (mg/day): ………….
start date: ………….. (year/month/day)
stop date: ………….. (year/month/day)
if no: previously used? yes / no
if yes: why was it stopped? the medication became unnecessary / unbearable side-effects / not effective enough / allergy /other

TNF alpha antagonist: yes / no
if yes: adalimumab / golimumab / etanercept / certolizumab pegol / infliximab / other
if adalimumab: yes / no
if no: previously used? yes / no
if yes: why was it stopped? the medication became unnecessary / unbearable side-effects / not effective enough / allergy /other
if yes golimumab: yes / no
if no: previously used? yes / no
if yes: why was it stopped? the medication became unnecessary / unbearable side-effects / not effective enough / allergy /other
if etanercept: yes / no
if no: previously used? yes / no
if yes: why was it stopped? the medication became unnecessary / unbearable side-effects / not effective enough / allergy /other
if certolizumab pegol: yes / no
if no: previously used? yes / no
if yes: why was it stopped? the medication became unnecessary / unbearable side-effects / not effective enough / allergy /other
if infliximab: yes / no
if no: previously used? yes / no
if yes: why was it stopped? the medication became unnecessary / unbearable side-effects / not effective enough / allergy /other

Rituximab: yes / no
if no: previously used? yes / no
if yes: why was it stopped? the medication became unnecessary / unbearable side-effects / not effective enough / allergy /other

IL-6R inhibitor: yes / no
if yes: tocilizumab / sarilumab / other
if tocilizumab: yes / no

if no: previously used? yes / no
if yes: why was it stopped? the medication became unnecessary / unbearable side-effects / not effective enough / allergy /other
if sarilumab: yes / no
if no: previously used? yes / no
if yes: why was it stopped? the medication became unnecessary / unbearable side-effects / not effective enough / allergy /other

JAK inhibitor: yes / no
if yes: tofacitinib / baricitinib / upadacitinib /filgotinib
if Tofacitinib: yes / no
if no: previously used? yes / no
if yes: why was it stopped? the medication became unnecessary / unbearable side-effects / not effective enough / allergy /other
if Baricitinib: yes / no
if no: previously used? yes / no
if yes: why was it stopped? the medication became unnecessary / unbearable side-effects / not effective enough / allergy /other
if upadacitinib: yes / no
if no: previously used? yes / no
if yes: why was it stopped? the medication became unnecessary / unbearable side-effects / not effective enough / allergy /other
if filgotinib: yes / no
if no: previously used? yes / no
if yes: why was it stopped? the medication became unnecessary / unbearable side-effects / not effective enough / allergy /other

Abatacept: yes / no
if no: previously used? yes / no
if yes: why was it stopped? the medication became unnecessary / unbearable side-effects / not effective enough / allergy /other

Antidepressants: yes / no
if yes: SSRI / SNRI / tricyclic antidepressant / other
if SSRI: fluoxetine / paroxetine / sertraline / citalopram / escitalopram / fluvoxamine / other
if SNRI: venlafaxine / duloxetine / desvenlafaxine / other
if tricyclic antidepressants: nortriptyline / desipramine / amitriptyline / clomipramine / imipramine / other
if other: mirtazapine /trazodone / nefazodone /bupropion / vortioxetine / varenicline / other

Benzodiazepines: yes / no
if yes: alprazolam / lorazepam / diazepam / clonazepam / other

Benzodiazepine-like substances (Z-drugs): yes / no
if yes: zolpidem / zaleplon

Melatonin agonists: yes / no

Other sedative-hypnotic drugs: yes / no
if yes: please specify: …………………………

Anticonvulsants: yes /no
if yes: gabapentin / pregabalin / carbamazepine / other:……………….

Ca channel blockers: yes / no
Pentoxyphyllin: yes / no
ACE inhibitors: yes / no
Angiotensin II receptor blockers (ARBs): yes / no
Proton pump inhibitors: yes / no
H2 receptor blockers: yes / no
Prokinetics: yes / no
Diuretics: yes / no
Anti-platelet agent: yes / no
Anticoagulants: yes / no
Oxygen supply: yes / no
Vitamin-D: yes / no

Calcium: yes / no
Thyroid hormone substitution: yes /no
Bronchodilator treatment: yes / no
Antidiabetic treatment: yes / no
if yes: pleases specify: …………
Other: yes / no
if yes: please specify:………………….

<u>3. Status</u>

Body weight (kg): …..………………………

Body height (cm): ………………

BMI (automatikusan számolt)

Blood pressure (Hgmm): ……..… / ………

Heart rate: ……………………… /minute

Patient Global Assessment of Disease Activity: …… mm

The PGA or Patient Global disease Activity estimate represents the patient's self assessment of disease activity on a 0-10 scale where 10 means maximal activity (VAS)

(Data for SDAI and CDAI)

0 mm Worst Activity | 100 mm Possible best

Evaluator Global Assessment of Disease Activity: …..mm

The EGA or Evaluator Global disease Activity estimate represents the evaluator's assessment of disease activity on a 0-10 scale where 10 means maximal activity. (VAS)

(Data for SDAI and CDAI)

0 mm Worst Activity | 100 mm Possible best

Patient Global Health: …….. mm (0-100 mm)

Measured in mm on Visual Analog Scale

(Data for DAS-28 ESR and DAS-28 CRP)

0 mm No pain | 100 mm Worst pain

SDAI: …….
(automatikusan számolt: SDAI = Tender Joint Count + Swollen Joint Count + CRP(mg/dL) + Patient Global Activity (VAS 0-10 cm) + Evaluator Global Activity (VAS 0-10 cm))

SDAI	***Disease Severity***
≤3.3	*Remission*
>3.3-11.0	*Low*
>11.0-26.0	*Moderate*
>26.0	*High*

CDAI: ……..
(automatikusan számolt: Tender Joint Count + Swollen Joint Count + Patient Global Activity (VAS 0-10 cm) + Evaluator Global Activity (VAS 0-10 cm))

CDAI Score Range	***Disease Severity***
≤ 2.8	*Remission*
> 2.8 - 10.0	*Low*
> 10.0 - 22.0	*Moderate*
> 22.0	*High*

DAS28-CRP: ……..
(automatikusan számolt: DAS28-CRP= (0.56*√(tender joint count) + 0.28*√(swollen joint count) + 0.36*ln(CRP + 1) + 0.014*patient global (VAS 0-100 mm)+ 0.96)
http://www.4s-dawn.com/DAS28/

DAS28-ESR: ………
(automatikusan számolt: DAS28-ESR=0.56*√(Tender Joint Count)+0.28*√(Swollen Joint Count)+0.7*ln(ESR (mm/hr))+0.014*(global health (VAS 0-100 mm))
http://www.4s-dawn.com/DAS28/

DAS28 Score Range	***Disease Severity***
< 2.6	*Remission*
≥ 2.6 - < 3.2	*Low*
≥ 3.2 - ≤ 5.1	*Moderate*
> 5.1	*High*

4. Symptoms / Complaints

Duration of symptoms: Less than 6 weeks / More than 6 weeks
Tender joints yes / no

if yes:

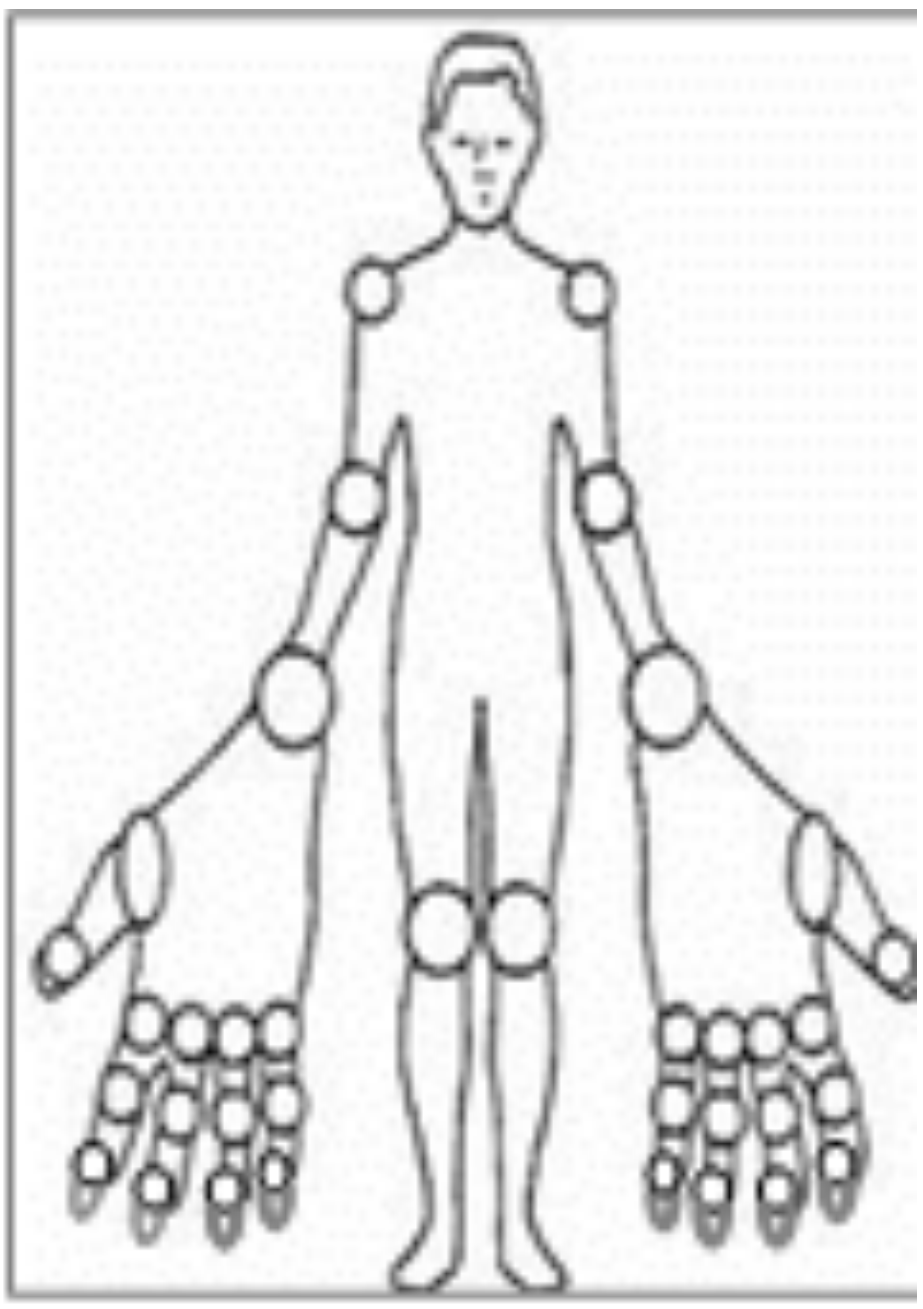

right PIP I:	yes / no
right PIP II:	yes / no
right PIP III:	yes / no
right PIP IV:	yes / no
right PIP V:	yes / no
right MCP I:	yes / no
right MCP II:	yes / no
right MCP III:	yes / no
right MCP IV:	yes / no
right MCP V:	yes / no
right Wrist:	yes / no
right Elbow:	yes / no
right Shoulder:	yes / no
right Knee:	yes / no
left PIP I:	yes / no
left PIP II:	yes / no
left PIP III:	yes / no
left PIP IV:	yes / no
left PIP V:	yes / no
left MCP I:	yes / no
left MCP II:	yes / no
left MCP III:	yes / no
left MCP IV:	yes / no
left MCP V:	yes / no
left Wrist:	yes / no
left Elbow:	yes / no
left Shoulder:	yes / no
left Knee:	yes / no

Number of tender joints: ……
(Data for SDAI, CDAI, DAS28-ESR and DAS28-CRP)

Swollen joints: yes / no

if yes:

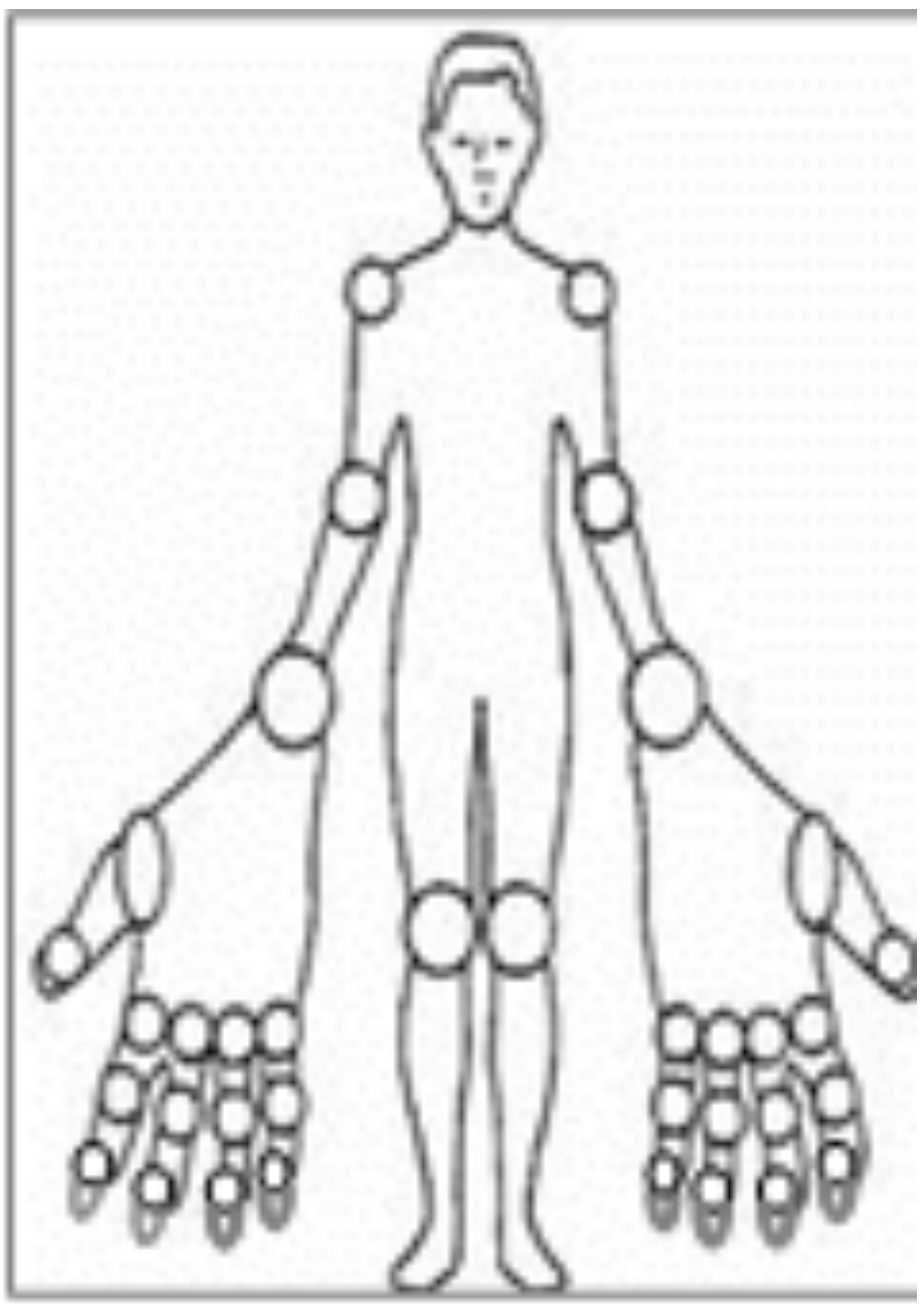

right PIP I:	yes / no
right PIP II:	yes / no
right PIP III:	yes / no
right PIP IV:	yes / no
right PIP V:	yes / no
right MCP I:	yes / no
right MCP II:	yes / no
right MCP III:	yes / no
right MCP IV:	yes / no
right MCP V:	yes / no
right Wrist:	yes / no
right Elbow:	yes / no
right Shoulder:	yes / no
right Knee:	yes / no
left PIPI:	yes / no
left PIP II:	yes / no
left PIP III:	yes / no
left PIP IV:	yes / no
left PIP V:	yes / no
left MCP I:	yes / no
left MCP II:	yes / no
left MCP III:	yes / no
left MCP IV:	yes / no
left MCP V:	yes / no
left Wrist:	yes / no
left Elbow:	yes / no

left Shoulder: yes / no
left Knee: yes / no

Number of swollen joints: ……
(Data for SDAI, CDAI, DAS28-ESR and DAS28-CRP)

Limitation of motion: yes / no
Joint deformities: yes / no
if yes: Number of affected joints: ……..
Morning stiffness: yes / no
if yes: Morning stiffness duration (min): …….
Tenosynovitis: yes / no

if yes: Please specify localization: ……………..

Rheumatic nodules: yes / no
if yes: Number of rheumatic nodules: ……
Skin lesions: **yes / no**
if yes: Digital ischaemia / Cutaneous ulcer / Nail fold infarcts / Purpuras / Palmar erythema / Other: …………
Sicca symptoms (dry eyes and/or dry mouth): yes / no
Scleritis: yes / no

Enlarged / palpable lymph nodes: yes / no

if yes: Pleases specify localization:………………

Presence of anasarca: yes / no
Headache: never / rarely / frequent
Dizziness: yes / no
Neck pain: yes / no
Lung involvement: yes / no

if yes: Dyspnea / Chest pain / Cough / Other: ……………….

Gastrointestinal involvement: yes / no

if yes: dysphagia / reflux / heartburn / nausea / vomiting / bloating / other: …….. (többszörösen választható)

Hepatomegaly: yes / no

Splenomegaly: yes / no

Stool: normal / diarrhea / constipation / fatty / putrid / undigested food/bloody/mucus (többszörösen választható)

How often: daily once/ daily twice/ more: ………./every second /third day/other…………. (egyszer választható)

Urinary disturbances: yes / no

if yes: painful urination / haematuria / nycturia / other: ………. (többször választható)

Subfebrility/fever: yes / no

if yes: since when:…………………………………

temperature: …………… ºC (axillary/rectal)

Appetite: good / retained / bad (egyszer választható)

Change in body weight: yes / no

if yes: direction of change: increase / decrease

how long did it take? (weeks):…………………………….

how much (kg):…………………………………………

Widespread Pain Index (WPI)

The WPI quantifies the extent of bodily pain on a 0-19 scale by asking patients if they have had pain or tenderness in 19 different body regions in the last 7 days.

Left upper region: yes / no
  if yes: Left jaw / Left shoulder girdle / Left upper arm / Left lower arm (többszörösen választható)
Right upper region: yes / no
  if yes: Right jaw / Right shoulder girdle / Right upper arm / Right lower arm (többszörösen választható)
Axial region: yes / no
  if yes: Neck / Upper back / Lower back / Chest / Abdomen (többszörösen választható)
Left lower region: yes / no
  if yes: Left hip (trochanter) / Left upper leg / Left lower leg (többszörösen választható)
Right lower region: yes / no
  if yes: Right hip (trochanter) / Right upper leg / Right lower leg (többszörösen választható)
Affected regions:………… (0-5)
WPI score:..........(0-19)

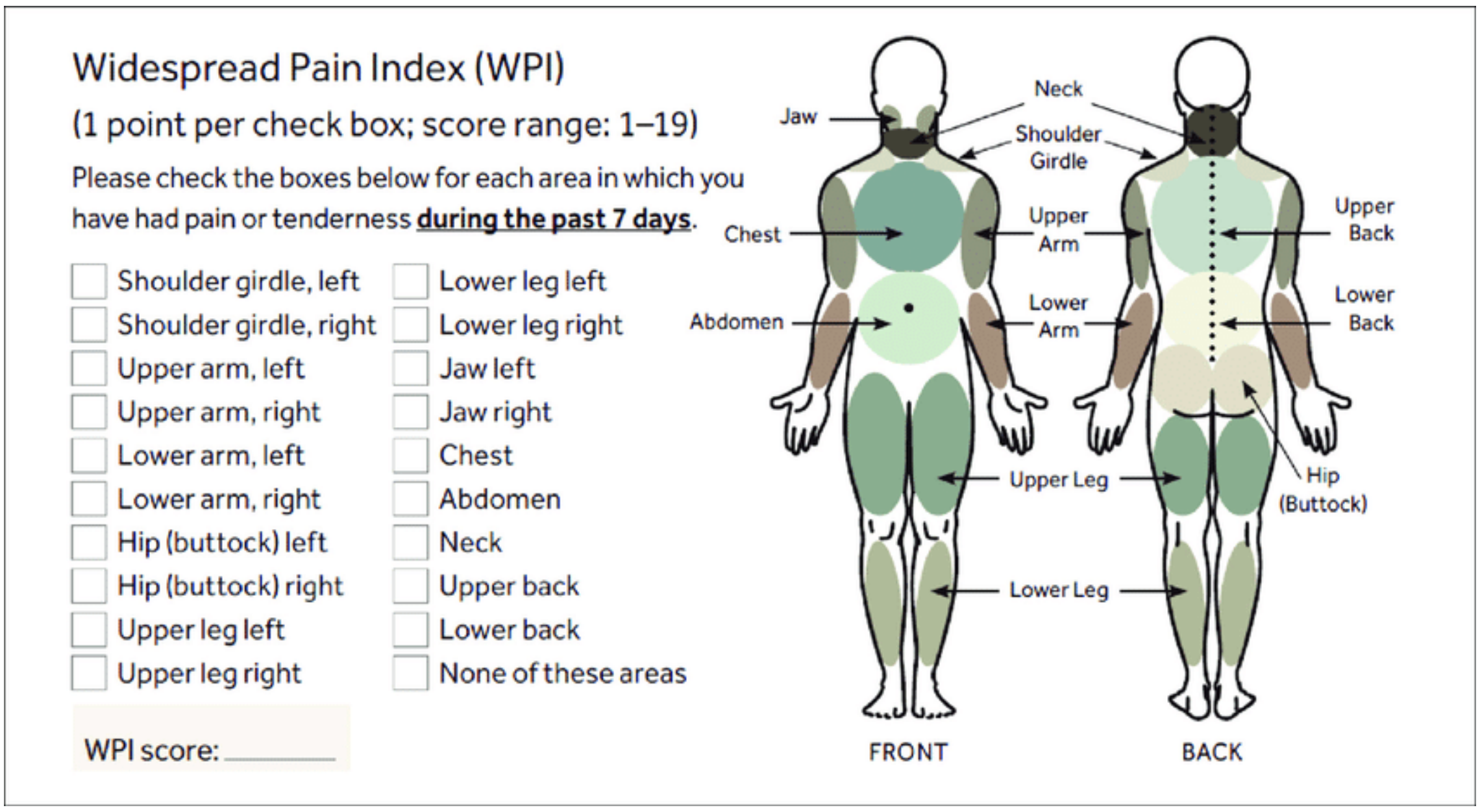

Symptoms severity score (SSS)

The SS scale quantifies symptom severity on a 0-12 scale in the past 7 days.

Fatigue: yes / no
  if yes: slight (1) / moderate (2) / severe (3)
Waking unrefreshed: yes / no
  if yes: slight (1) / moderate (2) / severe (3)
Cognitive symptoms: yes / no
  if yes: slight (1) / moderate (2) / severe (3)
Headaches: yes (1) / no
Pain and cramps in the lower abdomen: yes (1) / no
Depression: yes (1) / no

Total SSS:.........

/

/

Symptoms Severity Score (SSS)

For each of the following, for the past week, rate

	0=No problem	1=slight or mild problem, often mild or intermittent	2=moderate, considerable problem, often present	3=severe, pervasive, continuous, life-disturbing
Fatigue	☐	☐	☐	☐
Waking unrefreshed	☐	☐	☐	☐
Cognitive symptoms	☐	☐	☐	☐

In the past week, have you been bothered by any of the following?

	0=No problem	1=Problem
Headaches	☐	☐
Pain or cramps in lower abdomen	☐	☐
Depression	☐	☐

Total SSS: _______(0-12)

If *WPI ≥ 7 and SSS ≥ 5 OR WPI 4-6 and SSS ≥ 9*

AND Generalized pain: pain appears in 4/5 regions

AND symptoms present ≥ 3 months

suggest the diagnosis of fibromyalgia (if no other conditions contributes

painDETECT

How would you assess your pain now, at this moment?

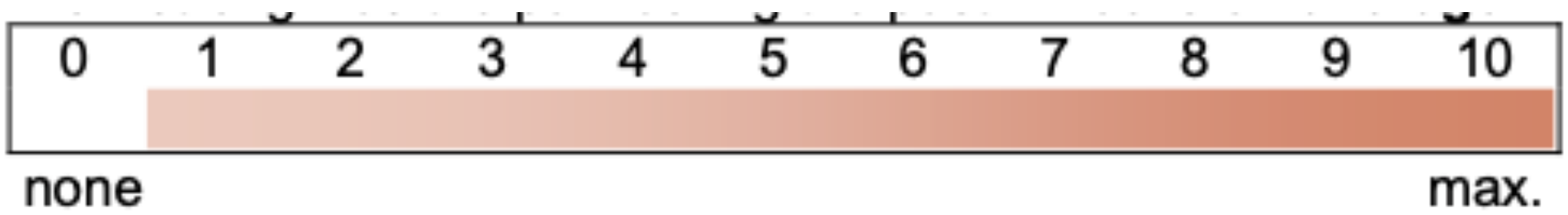

How was the strongest pain during the past 4 weaks?

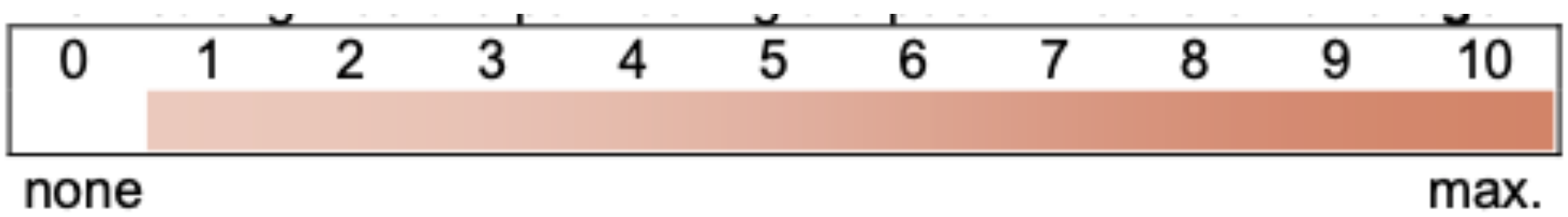

How strong was the pain during the past 4 weeks on average?

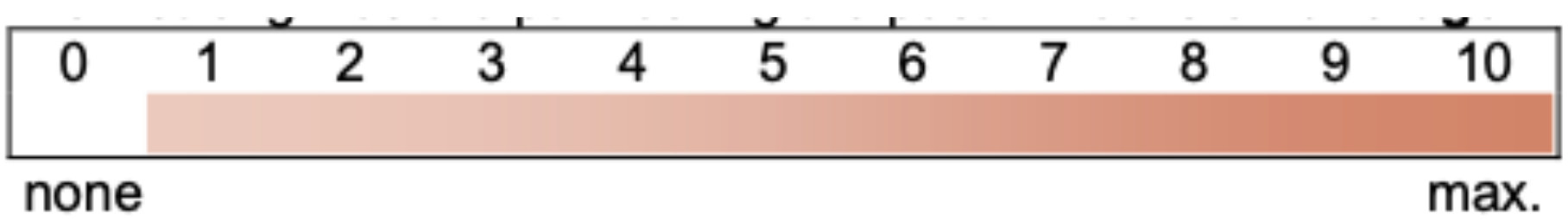

Do you suffer from a burning sensation (e.g., stinging nettles) in the marked areas?

never/ hardly noticed / slightly / moderately / strongly / very strongly

(Data for painDETECT Score)

Do you have a tingling or prickling sensation in the area of your pain (like crawling ants or electrical tingling)?

never/ hardly noticed / slightly / moderately / strongly / very strongly

(Data for painDETECT Score)

Is light touching (clothing, a blanket) in this area painful?

never/ hardly noticed / slightly / moderately / strongly / very strongly
(Data for painDETECT Score)

Do you have sudden pain attacks in the area of your pain, like electric shocks?

never/ hardly noticed / slightly / moderately / strongly / very strongly

(Data for painDETECT Score)

Is cold or heat (bath water) in this area occasionally painful?

never/ hardly noticed / slightly / moderately / strongly / very strongly

(Data for painDETECT Score)

Do you suffer from a sensation of numbness in the areas that you marked?

never/ hardly noticed / slightly / moderately / strongly / very strongl

(Data for painDETECT Score)

Does slight pressure in this area, e.g., with a finger, trigger pain?

never/ hardly noticed / slightly / moderately / strongly / very strongly

(Data for painDETECT Score)

automatikusan számolt
Total score = (number of never)*0 + (number of hardly noticed)*1 + (number of slightly)*2 + (number of moderately)*3 + (number of strongly)*4 + (number of very strongly) *5

How would you describe the course of your pain?

persistent pain with slight fluctuations / persistent pain with pain attacks / pain attacks without pain between them / pain attacks with pain between them

(Data for painDETECT Score)

automatikusan számolt

if marked:

persistent pain with slight fluctuations	Total score + 0
persistent pain with pain attacks	Total score – 1
pain attacks without pain between them	Total score + 1
pain attacks with pain between them	Total score + 1

Does your pain radiate to other regions of your body? yes/ no

if yes: Pleases specify direction:………………

(Data for painDETECT Score)

automatikusan számolt

if marked yes: Total Score + 2

painDETECT Final Score ....

(automatikusan számolt a korábbi instrukciók alapján)

if painDETECT Final Score is between 0-12

→ *neuropathic pain in unlikely (<15 %)*

if painDETECT Final Score is between 13-18

→ *Result is ambiguous, however a neuropathic pain component can be present*

if painDETECT Final Score is between 19-38

→ *A neuropathic pain component is likely (> 90%)*

5. Examinations

5.1 Laboratory / immunserology*

Date of laboratory examination: …………. (year/month/day)

**erythrocyte sedimentation rate (mm/h)**	
**CRP (mg/l)**	
**Blood: yes/no**	
**WBC count (G/l)**	
**RBC count (T/l)**	

**Haemoglobin (g/l)**	
**Haematocrit (%)**	
**MCV**	
**Platelet count (G/l)**	
**Ions: yes/no**	
**Sodium (mmol/l)**	
**Potassium (mmol/l)**	
**Calcium (mmol/l)**	
**Magnesium (mmol/l)**	
**Phosphate (mmol/l)**	
**Chloride (mmol/l)**	
**Iron (umol/l)**	
**Pancreas: yes/no**	
**Glucose (mmol/l)**	
**Amylase (U/l)**	
**Lipase (U/l)**	
**Renal functions: yes/no**	
**Urea nitrogen (Karbamid) (mmol/l)**	
**Creatinine (umol/l)**	
**eGFR**	
**Liver functions: yes/no**	
**Total bilirubin (umol/l)**	
**Direct/conjugated bilirubin (umol/l)**	
**Indirect bilirubin (umol/l)**	
**ASAT/GOT (U/l)**	
**ALAT/GPT (U/l)**	
**Gamma GT (U/l)**	
**Alkaline phosphatase (U/l)**	
**Lactate dehydrogenase LDH (U/l)**	
**Protrombin (%)**	
**Protrombin INR**	
**Metabolism: yes/no**	
**Cholesterol (mmol/l)**	

**HDL cholesterol (mmol/l)**	
**Triglycerides (mmol/l)**	
**Uric acid (umol/l)**	
**TSH (mU/l)l**	
**HgbA1C (%)**	
**Proteins: yes/no**	
**Total protein (g/l)**	
**Albumin (g/l)**	
**Fibrinogen (g/l)**	
**C3 (g/l)**	
**C4 (g/l)**	
**Other: yes/no**	

Appendix 2. Questionnaires used in the psychological assessment

General data (age, sex, residence)
Originally in Hungarian: Császár, N. (1995). *Állapotfelmérő füzet a Gerincgyógyászati betegek számára. [Assessment Booklet for Patients with Spinal Disorders.] Budapest* (Custom-developed questionnaire). Used with the author's prior permission.

Questions concern the process of self-assessment of health status
Originally in Hungarian: Császár, N. (1995). *Állapotfelmérő füzet a Gerincgyógyászati betegek számára. [Assessment Booklet for Patients with Spinal Disorders.] Budapest* (Custom-developed questionnaire). Used with the author's prior permission.

Visual Analogue Scale for pain measurement (VAS)
Bijur, P. E., Silver, W., & Gallagher, E. J. (2001). Reliability of the visual analog scale for measurement of acute pain. *Academic Emergency Medicine, 8*(12), 1153.
https://doi.org/10.1111/j.1553-2712.2001.tb01132.x
Originally in Hungarian: Császár, N. (1995). *Állapotfelmérő füzet a Gerincgyógyászati betegek számára. [Assessment Booklet for Patients with Spinal Disorders.] Budapest* (Custom-developed questionnaire). Used with the author's prior permission.

Lifestyle Questions
Originally in Hungarian: Császár, N. (1995). *Állapotfelmérő füzet a Gerincgyógyászati betegek számára. [Assessment Booklet for Patients with Spinal Disorders.] Budapest* (Custom-developed questionnaire). Used with the author's prior permission.

Questions about the use of the healthcare system
Originally in Hungarian: Császár, N. (1995). *Állapotfelmérő füzet a Gerincgyógyászati betegek számára. [Assessment Booklet for Patients with Spinal Disorders.] Budapest* (Custom-developed questionnaire). Used with the author's prior permission.

Questions about negative life events (losses)
Originally in Hungarian: Császár, N. (1995). *Állapotfelmérő füzet a Gerincgyógyászati betegek számára. [Assessment Booklet for Patients with Spinal Disorders.] Budapest* (Custom-developed questionnaire). Used with the author's prior permission.

The issues that are prospective in nature are to be addressed

Originally in Hungarian: Császár, N. (1995). *Állapotfelmérő füzet a Gerincgyógyászati betegek számára. [Assessment Booklet for Patients with Spinal Disorders.] Budapest* (Custom-developed questionnaire). Used with the author's prior permission.

Holmes Rahe Social Readjustment Rating Scale. (SRSS)
Holmes, T. H., Rahe, R. H. (1967). The social readjustment rating scale. Journal of Psychosomatic Research, 11(2), 213-218.
DOI: 10.1016/0022-3999(67)90010-4
Originally in Hungarian: Dávid, I. (2014). A stressz és kezelése. In M. Fülöp, N. Pataky, & J. Rudas (Eds.), *Stressz, megküzdés, versengés, konfliktusok* (pp. 11–67). Magyar Tehetségsegítő Szervezetek Szövetsége. ISSN 2062-5936.

Illness Intrusiveness Rating Scale (IIRS)
Devins, G. M., Binik, Y. M., Hutchinson, T. A., Hollomby, D. J., Barré, P. E., & Guttmann, R. D. (1983). Illness Intrusiveness Rating Scale (IIRS). https://doi.org/10.1037/t32545-000
The Hungarian version: Novak, M., Mah, K., Molnar, M. Z., Ambrus, C., Csepanyi, G., Kovacs, A., Vamos, E., Zambo, M., Zoller, R., Mucsi, I., & Devins, G. M. (2005). Factor structure and reliability of the Hungarian version of the Illness Intrusiveness Scale: Invariance across North American and Hungarian dialysis patients. *Journal of Psychosomatic Research, 58*(1), 103–110. https://doi.org/10.1016/j.jpsychores.2004.05.008
and
Perczel-Forintos, D., Kiss, Zs., & Ajtay, G. (2018). Illness Intrusiveness Rating Scale. In D. Perczel-Forintos, Zs. Kiss, & G. Ajtay (Eds.), Questionnaires, rating scales in clinical psychology (pp. 41-42). Semmelweis Kiadó. ISBN: 978-963-331-442-5

Generalized Anxiety Scale (GAD-7)
Spitzer, R. L., Kroenke, K., Williams, J. B., & Löwe, B. (2006). A brief measure for assessing generalized anxiety disorder: The GAD-7. *Archives of Internal Medicine, 166*(10), 1092–1097. https://doi.org/10.1001/archinte.166.10.1092
GAD-7:https://www.phqscreeners.com/select-screener; (it needs no permission for use)

Beck Depression Scale (BDI)
Beck, A. T., Ward, C. H., Mendelsohn, M., Mock, J., & Erbaugh, J. (1961). An inventory for measuring depression. *Archives of General Psychiatry, 4*, 561–571.
https://doi.org/10.1001/archpsyc.1961.01710120031004
The Hungarian Version: Perczel-Forintos, D., Kiss, Zs., & Ajtay, G. (2018). Beck Depression Scale. In D. Perczel-Forintos, Zs. Kiss, & G. Ajtay (Eds.), Questionnaires, rating scales in clinical psychology (pp. 53-55). Semmelweis Kiadó. ISBN: 978-963-331-442-5

WHO General Well-being (WHO-5)
Bech, P. (2004). Measuring the dimensions of psychological general well-being by the WHO-5. *QoL Newsletter, 32*, 15–16. https://eprovide.mapi-trust.org/instruments/who-five-well-being-index. © Psychiatric Research Unit, WHO Collaborating Center for Mental Health, Frederiksborg General Hospital, DK-3400 Hillerød. https://cdn.who.int/media/docs/default-source/mental-health/five-well-being-index-(who-5)/who-5_hungarian.pdf?sfvrsn=357833bd_3
Originally in Hungarian: Susánszky, É., Konkoly Thege, B., Stauder, A., & Kopp, M. (2006). A WHO jól-lét kérdőív rövidített (WBI-5) magyar változatának validálása a Hungarostudy 2002 országos lakossági egészségfelmérés alapján. *Mentálhigiéné és Pszichoszomatika, 7*(3), 247–255. https://doi.org/10.1556/mental.7.2006.3.8

Perceived Stress Scale (PSS-4)
Cohen, S., Kamarck, T., & Mermelstein, R. (1983). A global measure of perceived stress. *Journal of Health and Social Behavior, 24*, 385–396. https://doi.org/10.2307/2136404
Originally in Hungarian: Stauder, A., & Konkolÿ Thege, B. (2006). Az észlelt stressz kérdőív (PSS) magyar verziójának jellemzői. *Mentálhigiéné és Pszichoszomatika, 7*(3), 203–216. https://doi.org/10.1556/mental.7.2006.3.4

Connor-Davidson Resilience Scale (CD-RISC)
Connor, K. M., & Davidson, J. R. T. (2003). Development of a new resilience scale: The Connor-Davidson Resilience Scale (CD-RISC). *Depression and Anxiety, 18*(2), 76–82. https://doi.org/10.1002/da.10113
The Hungarian version: Kiss, E. C., Vajda, D., Káplár, M., Csókási, K., Hargitai, R., & Nagy, L. (2015). The Hungarian adaptation of the 25-item Connor-Davidson Resilience Scale. *Mentálhigiéné és Pszichoszomatika, 16*(1), 93–113. https://doi.org/10.1556/mental.16.2015.1.4

Tampa Scale of Kinesiophobia (TSK)
Miller, R. P., Kori, S. H., & Todd, D. D. (1991). The Tampa Scale: A measure of kinesiophobia. *The Clinical Journal of Pain, 7*(1), 51.
https://journals.lww.com/clinicalpain/citation/1991/03000/the_tampa_scale__a_measure_of_kinisophobia.53.aspx
Dupuis, F., Cherif, A., Batcho, C., Massé-Alarie, H., & Roy, J.-S. (2023). The Tampa Scale of Kinesiophobia: A systematic review of its psychometric properties in people with musculoskeletal pain. *The Clinical Journal of Pain, 39*(5), 236–247.
https://doi.org/10.1097/AJP.0000000000001104
Originally in Hungarian: Simoncsics, E. (2023). *Attitűdök és a kognitív megközelítés szerepe a krónikus deréktáji fájdalomban* [Doctoral dissertation, Semmelweis University]. Semmelweis University Repository. https://doi.org/10.14753/SE.2023.2869

Patient-Reported Outcomes Measurement Information System (PROMIS-PF-4A)
Cella, D., Yount, S., Rothrock, N., Gershon, R., Cook, K., & Reeve, B. (2007). The Patient-Reported Outcomes Measurement Information System (PROMIS): Progress of an NIH Roadmap Cooperative Group during its first two years. *Medical Care, 45*(5 Suppl 1), S3–S11. https://doi.org/10.1097/01.mlr.0000258615.42478.55
Jenei, B., Bató, A., Mitev, A. Z., Brodszky, V., & Rencz, F. (2023). Hungarian PROMIS-29+2: Psychometric properties and population reference values. *Quality of Life Research, 32*(8), 2179–2194. https://doi.org/10.1007/s11136-023-03364-7

Patient Health Questionnaire-15 (PHQ-15)

Kroenke, K., Spitzer, R. L., & Williams, J. B. W. (2002). The PHQ-15: Validity of a new measure for evaluating the severity of somatic symptoms. *Psychosomatic Medicine, 64*(2), 258–266. https://doi.org/10.1097/00006842-200203000-00008

The Hungarian version: Stauder, A., Witthöft, M., & Köteles, F. (2021). Validation of the Hungarian PHQ-15: A latent variable approach. *Ideggyógyászati Szemle, 74*(5-6), 183–190.

https://doi.org/10.18071/isz.74.0183

Owestry Disability Index (ODI)
Fairbank, J. C., Pynsent, P. B. (2000) The Oswestry disability index. Spine. Nov 15;25(22):2940-53. DOI:10.1093/occmed/kqw051
The Hungarian version: Valasek, T., Varga, P. P., Szövérfi, Z., Kümin, M., Fairbank, J., & Lazary, A. (2013). Reliability and validity study on the Hungarian versions of the Oswestry Disability Index and the Quebec Back Pain Disability Scale. *European Spine Journal, 22*(5), 1010–1018. https://doi.org/10.1007/s00586-012-2645-9

Somatic Absorption Scale (SAS)
Köteles, F., Simor, P., & Tolnai, N. (2012). Psychometric evaluation of the Hungarian version of the Somatic Absorption Scale. *Mentálhigiéné és Pszichoszomatika, 13*(4), 375–395.
https://doi.org/10.1556/mental.13.2012.4.2

MOS- Social Support Survey (MOS SSS-H)
Sherbourne, D. C., & Stewart, A. L. (1991). The MOS Social Support Survey. *Social Science & Medicine, 32*, 705–714.
https://doi.org/10.1016/0277-9536(91)90150-B
Originally in Hungarian: Szentiványi-Makó, H., Bernáth, L., Szentiványi-Makó, N., Veszprémi, B., Vajda, D., & Kiss, E. C. (2016). A MOS SSS–társas támasz mérésére szolgáló kérdőív magyar változatának pszichometriai jellemzői. *Alkalmazott Pszichológia, 16*(3), 145–162. https://doi.org/10.17627/ALKPSZICH.2016.3.145

Pain Catastrophizing Scale (PCS)

Sullivan, M. J. L., Bishop, S., & Pivik, J. (1995). The Pain Catastrophizing Scale: Development and validation. *Psychological Assessment, 7*, 524–532. https://doi.org/10.1037/1040-3590.7.4.524

The Hungarian version: Galambos, A., Stoll, D. P., Bolczár, S., Lazáry, Á., Urbán, R., & Kökönyei, G. (2021). A bifactor structural model of the Hungarian Pain Catastrophizing Scale and latent classes of a clinical sample. *Heliyon, 7*(9), e08026. https://doi.org/10.1016/j.heliyon.2021.e08026

Positive and Negative Affect Scale (PANAS)

Watson, D., Clark, L. A., & Tellegen, A. (1988). Development and validation of brief measures of positive and negative affect: The PANAS scales. *Journal of Personality and Social Psychology, 54*(6), 1063–1070. https://doi.org/10.1037/0022-3514.54.6.1063

Originally in Hungarian: Rózsa, S. (2009). *A mindennapos testi tünetek pszichológiája és mérésének módszertana*. Eötvös Loránd Tudományegyetem Pszichológia Doktori Iskola. https://docplayer.hu/23744135-A-mindennapos-testi-tunetek-pszichologiaja-es-meresenek-modszertana.html

and

Gyollai, A., Simor, P., Köteles, F., & Demetrovics, Z. (2011). Psychometric properties of the Hungarian version of the original and the short form of the Positive and Negative Affect Schedule (PANAS). *Neuropsychopharmacologia Hungarica, 13*(2), 73–79. https://www.researchgate.net/publication/51223075_Psychometric_properties_of_the_Hungarian_version_of_the_original_and_the_short_form_of_the_Positive_and_Negative_Affect_Schedule_PAN

Cognitive Emotion Regulation Questionnaire (CERQ)

Garnefski, N; Kraaij, V; Spinhoven, Ph. (2002): Manual for the use of the cognitive emotion regulation questionnaire. Leiderdorp, DATEC
DOI:10.1027/1015-5759.23.3.141

The Hungarian version: Miklós, M., Martos, T., Kocsis-Bogár, K., & Perczel-Forintos, D. (2011). Psychometric properties of the Hungarian version of the Cognitive Emotion Regulation Questionnaire. *Psychiatria Hungarica, 26*(2), 102–111.
https://www.researchgate.net/publication/51202142_Psychometric_properties_of_the_Hungarian_version_of_the_Cognitive_Emotion_Regulation_Questionnaire
and Perczel-Forintos, D., Kiss, Zs., & Ajtay, G. (2018). Cognitive Emotion Regulation Questionnaire. In D. Perczel-Forintos, Zs. Kiss, & G. Ajtay (Eds.), Questionnaires, rating scales in clinical psychology (pp. 174-176). Semmelweis Kiadó. ISBN: 978-963-331-442-5

Psychological Inflexibility in Pain Scale (PIPS)

Wicksell, R. K., Renöfält, J., Olsson, G. L., Bond, F. W., & Melin, L. (2008). Avoidance and cognitive fusion—Central components in pain-related disability? Development and preliminary validation of the Psychological Inflexibility in Pain Scale (PIPS). *European Journal of Pain, 12*(4), 491–500.https://doi.org/10.1016/j.ejpain.2007.08.003

Pain Sensitivity Questionnaire (PSQ-H)

Ruscheweyh, R., Marziniak, M., Stumpenhorst, F., Reinholz, J., & Knecht, S. (2009). Pain sensitivity can be assessed by self-rating: Development and validation of the Pain Sensitivity Questionnaire. *Pain, 146*(1-2), 65–74. https://doi.org/10.1016/j.pain.2009.06.020

Originally in Hungarian: Gács, B. (2019). *A fájdalomadaptáció többszempontú pszichológiai vizsgálata: A téri- és az időtényezők szerepe* [Doctoral dissertation, Pécsi Tudományegyetem Általános Orvostudományi Kar]. https://docplayer.hu/149549149-A-fajdalomadaptacio-tobbszempontu-pszichologiai-vizsgalata-a-teri-es-az-idotenyezok-szerepe-doktori-phd-ertekezes.html

Current Opioid Misuse Measure (COMM)

Butler, S. F., Budman, S. H., Fernandez, K. C., Houle, B., Benoit, C., Katz, N., & Jamison, R. N. (2007). Development and validation of the Current Opioid Misuse Measure. *Pain, 130*(1), 144–156. https://doi.org/10.1016/j.pain.2007.01.014

Somatosensory Amplification Scale (SSAS)

Barsky, A. J., Wyshak, G., & Klerman, G. L. (1990). The Somatosensory Amplification Scale and its relationship to hypochondriasis. *Journal of Psychiatric Research, 24*(4), 323–334. https://doi.org/10.1016/0022-3956(90)90004-A

The Hungarian version: Köteles, F., Gémes, H., Papp, G., Túróczi, P., Pásztor, A., Freyler, A., Szemerszky, R., & Bárdos, G. (2009). Validation of the Hungarian version of the Somatosensory Amplification Scale (SSAS). *Mentálhigiéné és Pszichoszomatika, 10*(4), 321–335. https://doi.org/10.1556/mental.10.2009.4.3

Health-Specific Locus-of-Control Beliefs (HLC)

Lau, R. R., & Ware, J. F. (1981). Refinements in the measurement of health-specific locus-of-control beliefs. *Medical Care, 19*(11), 1147–1158.
https://doi.org/10.1097/00005650-198111000-00007

Anger Expression Scale (AES)

Spielberger, C. D., Johnson, E. H., Russell, S. F., Crane, R. J., Jacobs, G. A., & Worden, T. J. (1985). The experience and expression of anger: Construction and validation of an anger expression scale. In M. A. Chesney & R. H. Rosenman (Eds.), *Anger and hostility in cardiovascular and behavioral disorders* (pp. 5–30). Hemisphere/McGraw-Hill. ISBN: 089116393X, 9780891163930
The Hungarian Version: Perczel-Forintos, D., Kiss, Zs., & Ajtay, G. (2018). *Anger Expression Scale*. In D. Perczel-Forintos, Zs. Kiss, & G. Ajtay (Eds.), *Questionnaires, rating scales in clinical psychology* (pp. 158–159). Semmelweis Kiadó. ISBN: 978-963-331-442-5

Ways of Coping Questionnaire (WOC)

Folkman, S., & Lazarus, R. (1988). *Manual for the Ways of Coping questionnaire*. Consulting Psychologists.
https://doi.org/10.1037/t06501-000
Originally in Hungarian: Rózsa, S., Purebl, G., Susánszky, É., Kő, N., Szádóczky, E., Réthelyi, J., Danis, I., Skrabski, Á., & Kopp, M. (2008). A megküzdés dimenziói: A konfliktusmegoldó kérdőív hazai változata. *Mentálhigiéné és Pszichoszomatika, 9*(3), 217–241. https://doi.org/10.1556/Mentál.9.2008.3.3
and
Perczel-Forintos, D., Kiss, Zs., & Ajtay, G. (2018). Ways of Coping Questionnaire. In D. Perczel-Forintos, Zs. Kiss, & G. Ajtay (Eds.), *Questionnaires, rating scales in clinical psychology* (pp. 172–173). Semmelweis Kiadó. ISBN: 978-963-331-442-5

Young Schema Questionnaire (SQ)

Schmidt, N. B., Joiner, T. E., Young, J. E., & Telch, M. J. (1995). The Schema Questionnaire: Investigation of psychometric properties and the hierarchical structure of a measure of maladaptive schemas. *Cognitive Therapy and Research, 19*(3), 295–321.
https://doi.org/10.1007/BF02230402
Originally in Hungarian: Unoka, Zs., Rózsa, S., Fábián, Á., Mervó, B., & Simon, L. (2004). A Young-féle Séma Kérdőív: A korai maladaptív sémák jelenlétét mérő eszköz pszichometriai jellemzőinek vizsgálata. *Psychiatria Hungarica, 19*(3), 235–243.
https://www.doki.net/tarsasag/pszichiatria/upload/pszichiatria/magazine/0403/04030102.html
and
Perczel-Forintos, D., Kiss, Zs., & Ajtay, G. (2018). Young Schema Questionnaire. In D. Perczel-Forintos, Zs. Kiss, & G. Ajtay (Eds.), *Questionnaires, rating scales in clinical psychology* (pp.207–219). Semmelweis Kiadó. ISBN: 978-963-331-442-5

Bem Sex Role Inventory (BSRI)

Bem, S. L. (1974). *Bem Sex Role Inventory*.
https://doi.org/10.1037/t00748-000
Originally in Hungarian: Meskó, N., Láng, A., Ferenczhalmy, R., Miklós, K., & Glöckner, B. (2013). Szülői bánásmód összefüggése a konfliktuskezeléssel: Nemi különbségek és nemi szerepek. *Mentálhigiéné és Pszichoszomatika, 14*(1), 45–66.
https://doi.org/10.1556/Mental.14.2013.1.3

Short Form of McGill Pain Questionnaire (SF-MPQ)

Melzack, R. (1975). The McGill Pain Questionnaire: Major properties and scoring methods. *Pain, 1*, 277–299.
https://doi.org/10.1016/0304-3959(75)90044-5
Strand, L. I., Ljunggren, A. E., Bogen, B., Ask, T., & Johnsen, T. B. (2008). The Short-Form McGill Pain Questionnaire as an outcome measure: Test-retest reliability and responsiveness to change. *European Journal of Pain, 12*(7), 917–925.
https://doi.org/10.1016/j.ejpain.2007.12.013

Originally in Hungarian: Bende, J., Ménesi, R., Ure, B., Troidl, H., Baltás, B., Lencz, L., & Szegesdi, I. (1993). A McGill-féle fájdalom-kérdőív magyar nyelvű adaptálása. *Aneszteziológia és Intenzív Terápia, 23*(2), 99–103. SSN 0133-5405.
http://mob.gyemszi.hu/detailsperm.jsp?PERMID=56898

Chronic Disability Index of Waddell and Main for Patients with Low Back Pain (CDI)

Waddell, G., & Main, C. J. (1984). Assessment of severity in low-back disorders. *Spine, 9*, 204–208. https://doi.org/10.1097/00007632-198403000-00012(Oswestry Disability Questionnaire or Roland Morris Disability Questionnaire (R.M.D.Q) has more validation data nowadays)

Fibromyalgia Impact Questionnaire (FIQR)

Bennett, R. M., Friend, R., Jones, K. D., Ward, R., Han, B. K., & Ross, R. L. (2009). The Revised Fibromyalgia Impact Questionnaire (FIQR): Validation and psychometric properties. *Arthritis Research & Therapy, 11*(4), R120.
https://doi.org/10.1186/ar2783

Rheumatoid Arthritis Self Report Questionnaire (SRQ)

Wolfe, F., Pincus, T., Thompson, A. K., & Doyle, J. (2003). The assessment of rheumatoid arthritis and the acceptability of self-report questionnaires in clinical practice. *Arthritis & Rheumatism, 49*(1), 59–63. https://doi.org/10.1002/art.10904
Pincus, T., Callahan, L. F., Brooks, R. H., Fuchs, H. A., Olsen, N. J., & Kaye, J. J. (1989). Self-report questionnaire scores in rheumatoid arthritis compared with traditional physical, radiographic, and laboratory measures. *Annals of Internal Medicine, 110*(4), 259–266. https://doi.org/10.7326/0003-4819-110-4-259

Spielberger- State-Trait Anxiety Inventory (STAI)

Skapinakis, P. (2014). Spielberger State-Trait Anxiety Inventory. In *Encyclopedia of Quality of Life and Well-Being Research* (pp. 6261–6264). https://doi.org/10.1007/978-94-007-0753-5_2825
The Hungarian version: Sipos, K., & Sipos, M. (1978). The development and validation of the Hungarian form of the STAI. *Cross-Cultural Anxiety*, 51-61. https://www.researchgate.net/publication/285022988
and
Perczel-Forintos, D., Kiss, Zs., & Ajtay, G. (2018). Spielberger- State-Trait Anxiety Inventory. In D. Perczel-Forintos, Zs. Kiss, & G. Ajtay (Eds.), Questionnaires, rating scales in clinical psychology (pp. 30-32). Semmelweis Kiadó. ISBN: 978-963-331-442-5

The Neck Disability Index (NDI)

Vernon, H., & Mior, S. (1991). The Neck Disability Index: A study of reliability and validity. *Journal of Manipulative and Physiological Therapeutics, 14*, 409–415.
https://doi.org/10.1007/978-94-007-0753-5_1915

Supplementary Material 2 – Exploratory sex-related sensitivity analysis of psychological assessment measures

Additional exploratory analyses were performed to assess the potential influence of sex on the psychological findings. These analyses used two-way ANOVA models including diagnostic group, sex, and the group × sex interaction term. No relevant interaction effect was observed for the Rorschach-based results and most of the questionnaire-based findings (data not shown).

For the following questionnaire-based baseline assessment measures, significant group x sex interaction effects were detected:

1. Illness Intrusiveness Rating Scale (IIRS) questionnaire

Relationship and personal development subscale

Descriptives plots

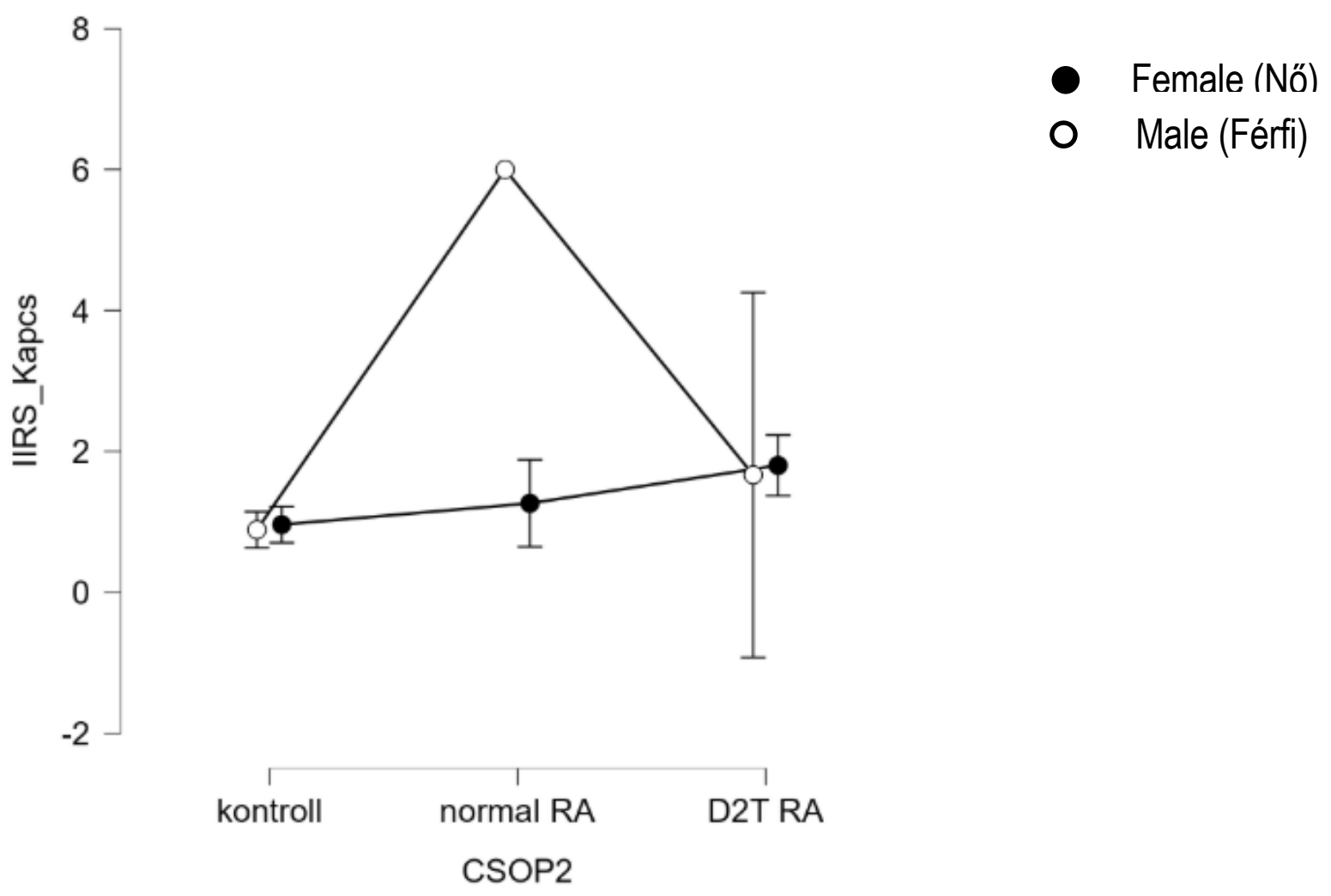

ANOVA - IIRS_Kapcs

Cases	Sum of Squares	df	Mean Square	F	p	η²
CSOP2	26.12	2	13.06	13.85	< .001	0.201
Kromoszomalis_nem	13.64	1	13.64	14.46	< .001	0.105
CSOP2 ✻ Kromoszomalis_nem	20.12	2	10.06	10.67	< .001	0.155
Residuals	69.78	74	0.943			

Note. Type III Sum of Squares

Descriptives

Descriptives - IIRS_Kapcs

CSOP2	Kromoszomalis_nem	N	Mean	SD	SE	Coefficient of variation
kontroll	férfi	9	0.889	0.333	0.111	0.375
	nő	22	0.962	0.579	0.124	0.602
normal RA	férfi	1	6.000			
	nő	17	1.265	1.200	0.291	0.949
D2T RA	férfi	4	1.667	1.627	0.814	0.976
	nő	27	1.802	1.089	0.210	0.604

kontroll= healthy controls, normal RA=non-D2T RA, Kromoszomalis_nem=Sex, CSOP2=Group, férfi=male, nő=female

Intimity subscale

Descriptives plots ▼

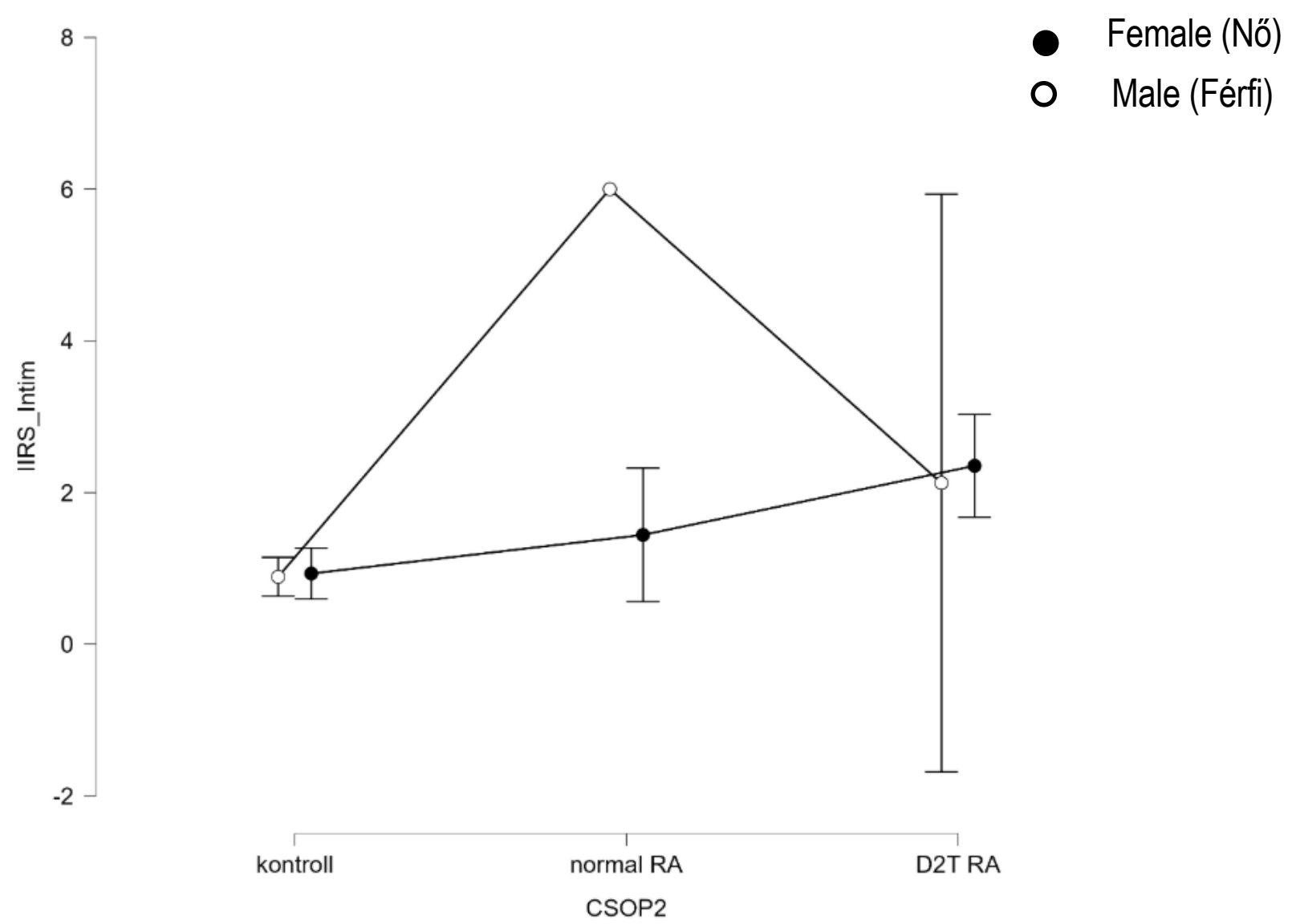

ANOVA - IIRS_Intim

Cases	Sum of Squares	df	Mean Square	F	p	η²
CSOP2	34.81	2	17.40	8.386	< .001	0.159
Kromoszomalis_nem	12.24	1	12.24	5.900	.018	0.056
CSOP2 ✻ Kromoszomalis_nem	18.85	2	9.424	4.541	.014	0.086
Residuals	153.6	74	2.075			

Note. Type III Sum of Squares

Descriptives

Descriptives - IIRS_Intim

CSOP2	Kromoszomalis_nem	N	Mean	SD	SE	Coefficient of variation
kontroll	férfi	9	0.889	0.333	0.111	0.375
	nő	22	0.932	0.761	0.162	0.816
normal RA	férfi	1	6.000			
	nő	17	1.441	1.713	0.415	1.189
D2T RA	férfi	4	2.125	2.394	1.197	1.126
	nő	27	2.352	1.714	0.330	0.729

kontroll= healthy controls, normal RA=non-D2T RA, Kromoszomalis_nem=Sex, CSOP2=Group, férfi=male, nő=female

Sum

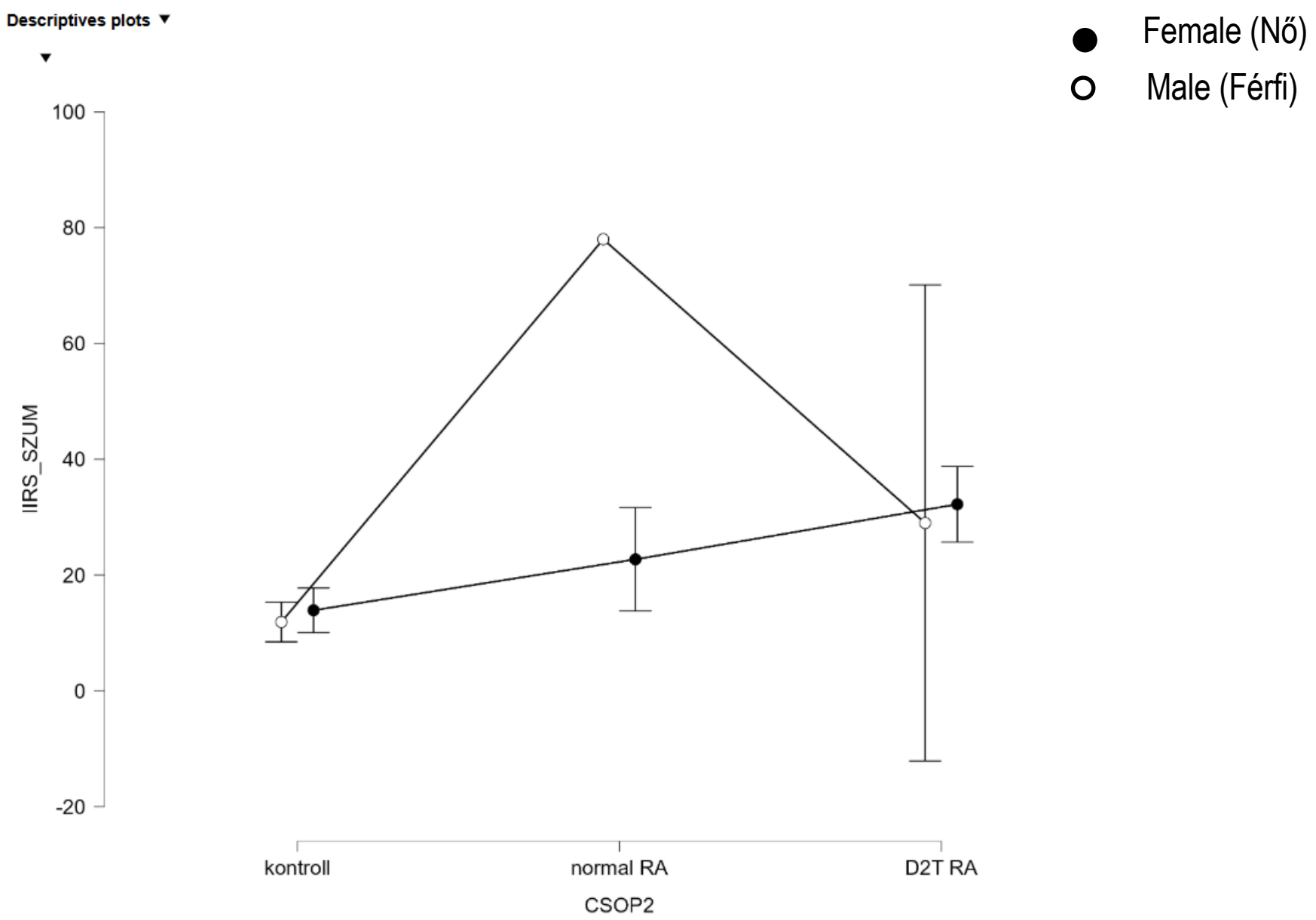

ANOVA - IIRS_SZUM

Cases	Sum of Squares	df	Mean Square	F	p	η²
CSOP2	6186	2	3093	14.62	< .001	0.234
Kromoszomalis_nem	1667	1	1667	7.882	.006	0.063
CSOP2 ✻ Kromoszomalis_nem	2877	2	1438	6.799	.002	0.109
Residuals	15650	74	211.6			

Note. Type III Sum of Squares

Descriptives

Descriptives - IIRS_SZUM

CSOP2	Kromoszomalis_nem	N	Mean	SD	SE	Coefficient of variation
kontroll	férfi	9	11.89	4.485	1.495	0.377
	nő	22	13.91	8.706	1.856	0.626
normal RA	férfi	1	78.00			
	nő	17	22.71	17.37	4.214	0.765
D2T RA	férfi	4	29.00	25.85	12.92	0.891
	nő	27	32.22	16.49	3.173	0.512

kontroll= healthy controls, normal RA=non-D2T RA, Kromoszomalis_nem=Sex, CSOP2=Group, férfi=male, nő=female

2. Owestry Disability Index (ODI) questionnaire

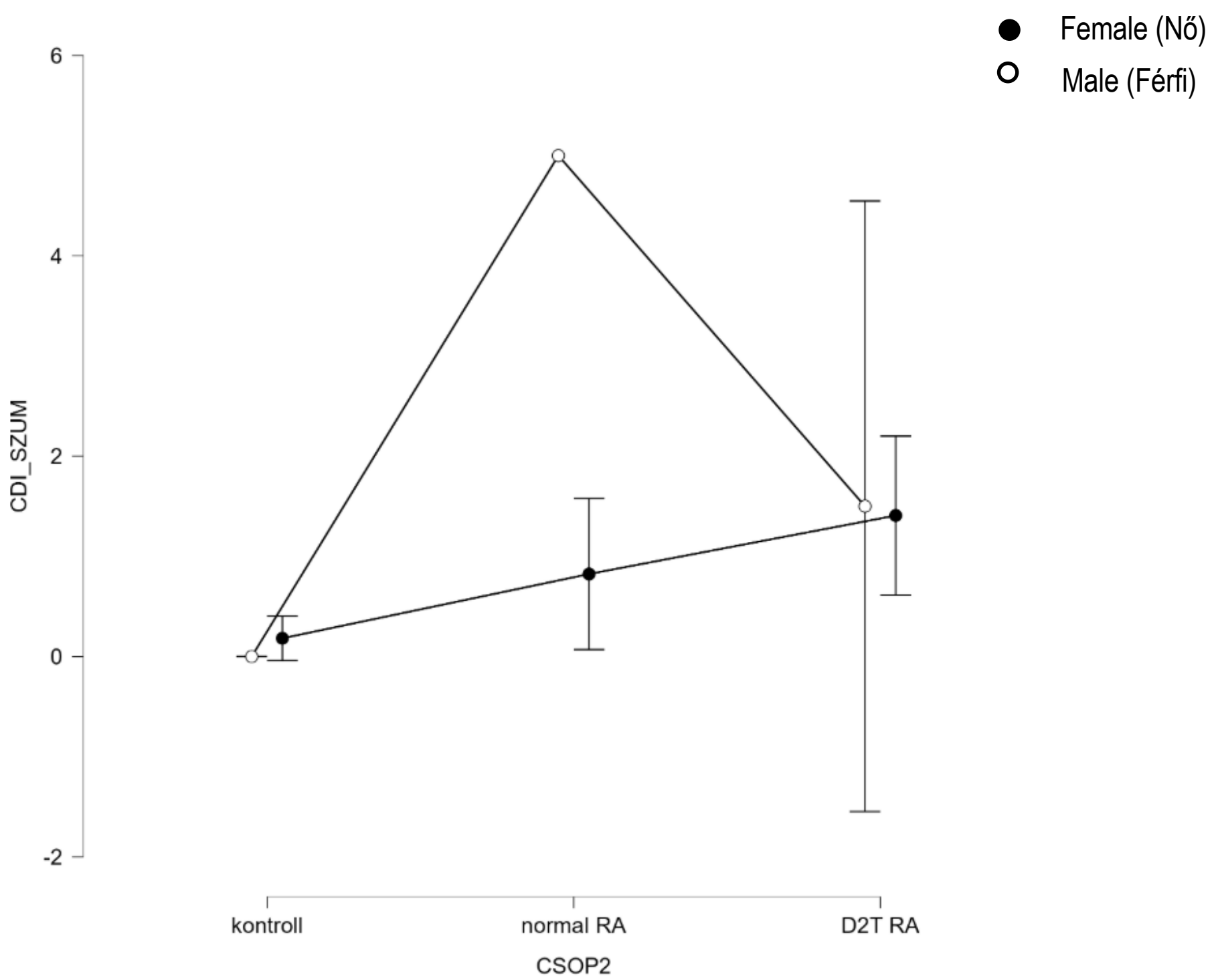

ANOVA - CDI_SZUM

Cases	Sum of Squares	df	Mean Square	F	p	η²
CSOP2	35.62	2	17.81	8.489	< .001	0.164
Kromoszomalis_nem	11.12	1	11.12	5.300	.024	0.051
CSOP2 ∗ Kromoszomalis_nem	15.82	2	7.912	3.771	.028	0.073
Residuals	155.3	74	2.098			

Note. Type III Sum of Squares

Descriptives

Descriptives - CDI_SZUM

CSOP2	Kromoszomalis_nem	N	Mean	SD	SE	Coefficient of variation
kontroll	férfi	9	0.000	0.000	0.000	NaN
	nő	22	0.182	0.501	0.107	2.756
normal RA	férfi	1	5.000			
	nő	17	0.824	1.468	0.356	1.782
D2T RA	férfi	4	1.500	1.915	0.957	1.277
	nő	27	1.407	2.005	0.386	1.425

kontroll= healthy controls, normal RA=non-D2T RA, Kromoszomalis_nem=Sex, CSOP2=Group, férfi=male, nő=female

3. The Neck Disability Index (NDI) questionnaire

Sum

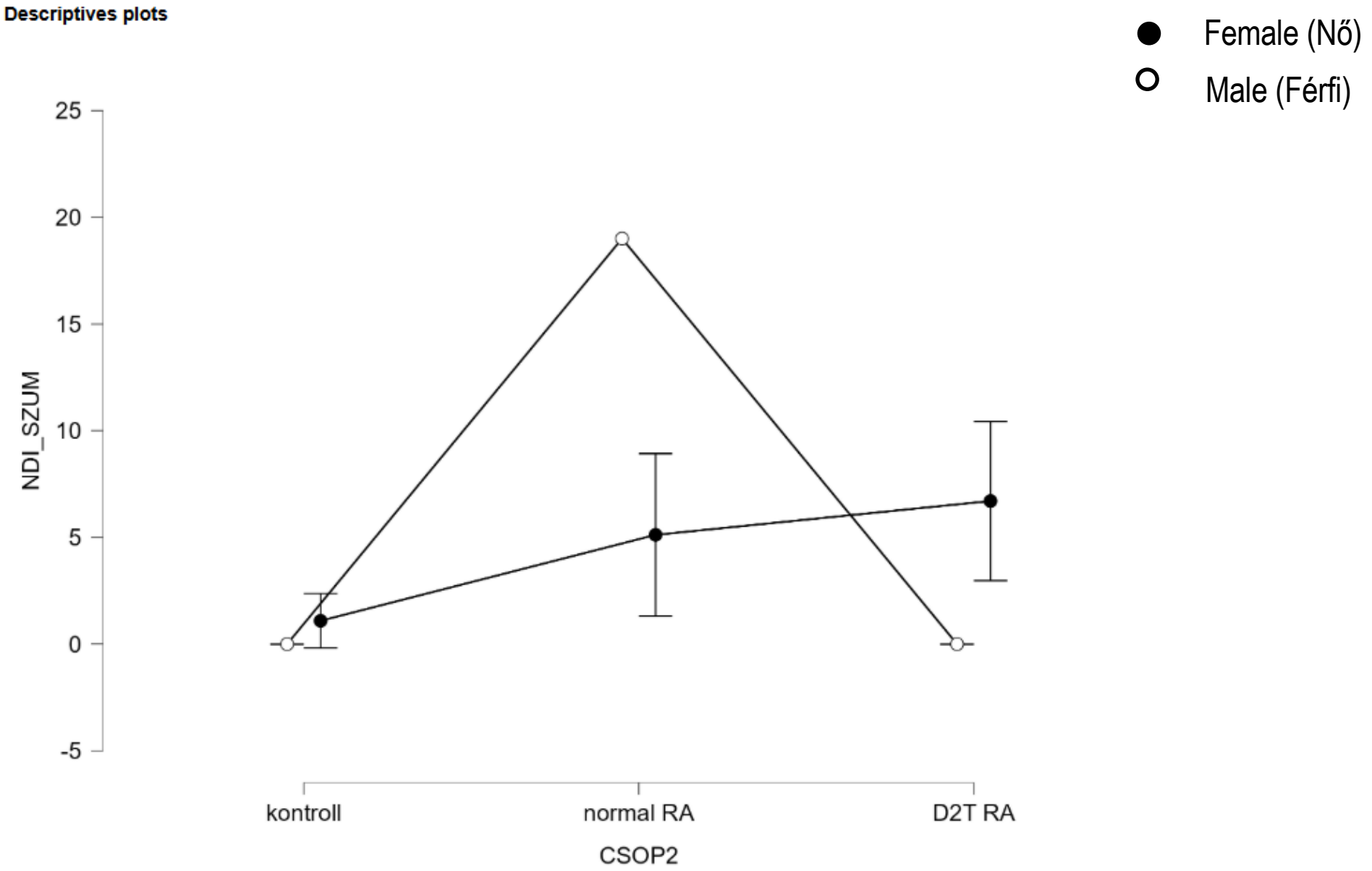

ANOVA - NDI_SZUM

Cases	Sum of Squares	df	Mean Square	F	p	η²
CSOP2	452.8	2	226.4	4.984	.009	0.109
Kromoszomalis_nem	24.67	1	24.67	0.543	.463	0.006
CSOP2 ✻ Kromoszomalis_nem	318.8	2	159.4	3.509	.035	0.077
Residuals	3361	74	45.42			

Note. Type III Sum of Squares

Descriptives ▼

Descriptives - NDI_SZUM

CSOP2	Kromoszomalis_nem	N	Mean	SD	SE	Coefficient of variation
kontroll	férfi	9	0.000	0.000	0.000	NaN
	nő	22	1.091	2.877	0.613	2.637
normal RA	férfi	1	19.00			
	nő	17	5.118	7.407	1.796	1.447
D2T RA	férfi	4	0.000	0.000	0.000	NaN
	nő	27	6.704	9.425	1.814	1.406

kontroll= healthy controls, normal RA=non-D2T RA, Kromoszomalis_nem=Sex, CSOP2=Group, férfi=male, nő=female

Percent (%)

Descriptives plots

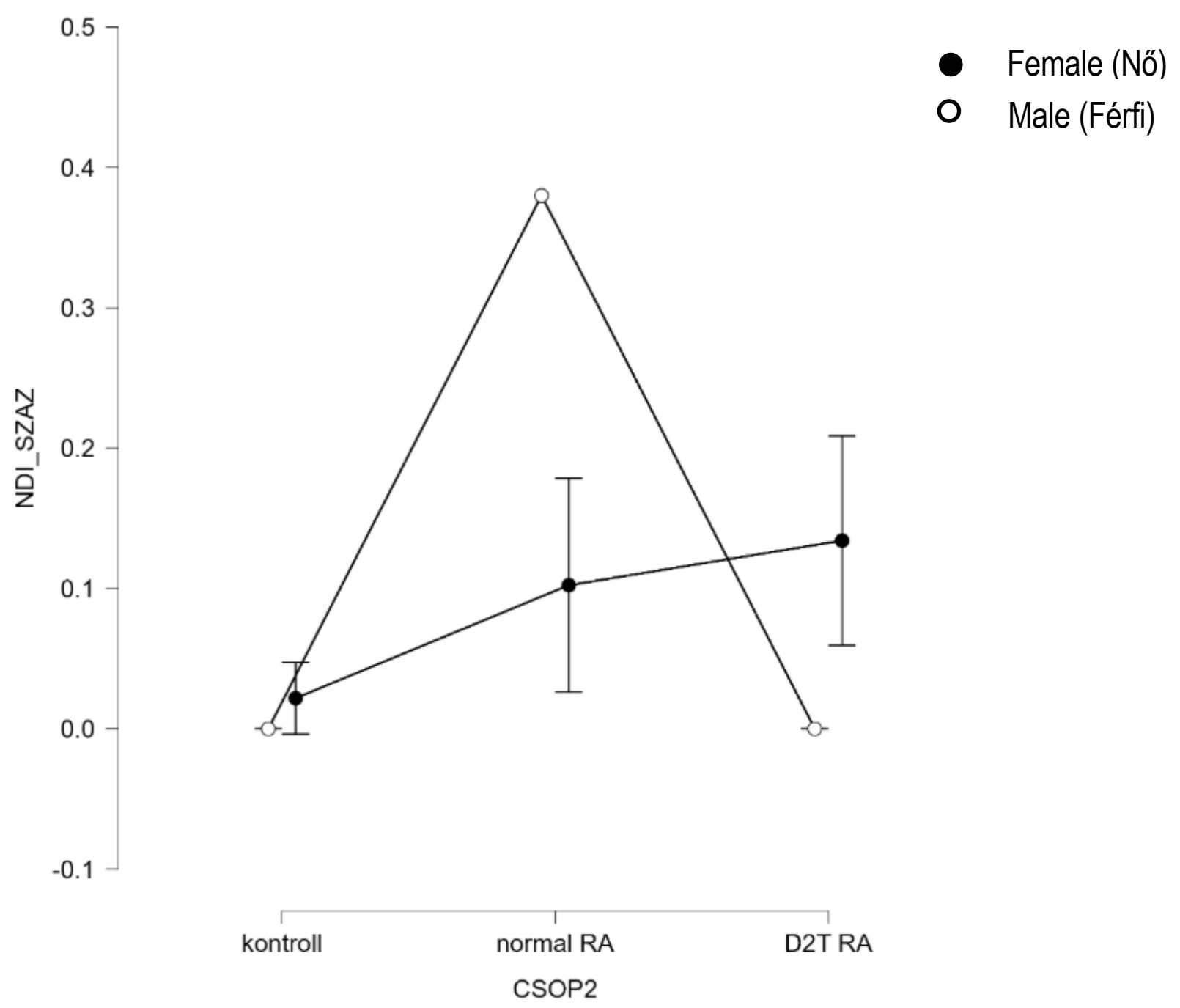

ANOVA - NDI_SZAZ

Cases	Sum of Squares	df	Mean Square	F	p	η²
CSOP2	0.181	2	0.091	4.984	.009	0.109
Kromoszomalis_nem	0.010	1	0.010	0.543	.463	0.006
CSOP2 ✻ Kromoszomalis_nem	0.128	2	0.064	3.509	.035	0.077
Residuals	1.344	74	0.018			

Note. Type III Sum of Squares

Descriptives ▼

Descriptives - NDI_SZAZ

CSOP2	Kromoszomalis_nem	N	Mean	SD	SE	Coefficient of variation
kontroll	férfi	9	0.000	0.000	0.000	NaN
	nő	22	0.022	0.058	0.012	2.637
normal RA	férfi	1	0.380			
	nő	17	0.102	0.148	0.036	1.447
D2T RA	férfi	4	0.000	0.000	0.000	NaN
	nő	27	0.134	0.189	0.036	1.406

Summary of findings:

Post-hoc inspection showed that these were mainly driven by sparse subgroup cells, particularly by one male participant in the non-D2T RA group with an outlying value. Therefore, these statistically significant interaction effects were considered unstable and not clinically interpretable, and they did not alter the interpretation of the psychological findings reported in the manuscript.

kontroll= healthy controls, normal RA=non-D2T RA, Kromoszomalis_nem=Sex, CSOP2=Group, férfi=male, nő=female

Supplementary Material 3 – Post-hoc power sensitivity analysis of the transcriptomic comparisons

We performed a post-hoc sensitivity analysis of statistical power using the RNASeqPower package, applying the biological variability (coefficient of variation) estimated directly from our voom-normalised expression data. This approach uses the observed dispersion structure of our specific dataset rather than generic assumptions, making it more realistic than a standard formula-based calculation.

The two primary comparisons - all RA vs healthy controls and D2T vs non-D2T - were well-powered (0.86 and 0.89 respectively), exceeding the conventional 0.80 threshold. The LI-HP vs non-D2T subgroup comparison reached adequate power (0.80), while the HI-HP vs HI-LP comparison was moderately underpowered (0.64), consistent with its very small subgroup sizes. We used the minimum absolute fold change actually observed (range: 1.34–1.57) among detected DEGs instead of the value of 1.2 used by the treat function in the limma package to test whether the true differential expression of a gene significantly exceeds this pre-defined threshold. We also note that low power primarily increases the risk of false negatives - real differences that went undetected - rather than false positives. Significant findings detected under lower power conditions can be considered conservative in this respect, as they survived the significance threshold despite less favourable statistical conditions.

**comparison**	**power**
all_RA_vs_healthy_controls	0.85830085
d2t_ra_vs_non_d2t_ra	0.8919171
hihp_vs_hilp	0.64101875
lihp_vs_non_d2t	0.80267737

**comparison**	**fold_change_threshold_tested**	**actual_minimal_fold_change**
all_RA_vs_healthy_controls	1.2	1.34
D2T_vs_non_D2T	1.2	1.49
HIHP_vs_HILP	1.2	1.55
LIHP_vs_non_D2T	1.2	1.57

Supplementary Material 4 – Sensitivity analysis assessing the influence of sex on transcriptomic findings

To directly address the concern about combining males and females, we performed a sex-adjusted sensitivity analysis by adding sex as a covariate to the limma design matrix for all comparisons (~ group + sex), and compared the resulting DEG lists to the original models. Concordance between original and sex-adjusted models was high across all comparisons:

Comparison	Original DEGs	Sex-adjusted DEGs	Shared	Concordance
All RA vs HC	269	288	213	79.2%
D2T vs non-D2T	87	79	74	85.1%
HI-HP vs HI-LP	208	164	148	71.2%
LI-HP vs non-D2T	73	73	64	87.7%

The majority of DEGs were shared between models in all comparisons, with concordance ranging from 71.2% to 87.7%. These findings confirm that the main results are not materially driven by sex differences between groups. We acknowledge that the HI-HP vs HI-LP comparison showed the lowest concordance (71.2%), which we attribute primarily to the reduced degrees of freedom when adding sex to a model with n=10 vs n=8 - an expected consequence of sex adjustment in very small subgroups, rather than evidence of sex confounding in the original analysis. The complete lists of the DEG are listed in the Supplementary Material

Supplementary Material 5 – Clinical feature details (data for Fig.2)

	Healthy controls	Rheumatoid arthritis patients				*p-value*
	**n=32**	**n=49**				
		**non-D2T RA**	**D2T RA**			
		n=18	n=31			
			High inflammation, high pain	High inflammation, low pain	Low inflammation, high pain	
			n=10	n=11	n=10	
**Disease duration**	-	18.50 (9.00, 21.25)	15.00 (3.75, 20.00)	17.00 (14.00, 20.50)	15.50 (10.00, 22.75)	*0.6976*
**DAS28-CRP**	-	3.26 (2.85, 4.52)	5.36 (5.23, 5.92)	4.10 (3.80, 4.86)	4.70 (3.86, 4.96)	***0.0002***
**DAS28-ESR**	-	2.64 (2.26, 4.09)	5.42 (4.89, 5.96)	4.03 (3.83, 4.78)	4.07 (3.03, 4.66)	***0.0002***
**CRP (mg/l)**	0.90 (0.57, 1.80)	1.80 (0.51, 3.58)	11.32 (1.75, 28.11)	11.20 (8.00, 22.00)	0.66 (0.30, 1.18)	***<0.0001***
**ESR (mm/hr)**	3.50 (2.00, 8.00)	11.00 (3.00, 21.00)	31.00 (23.50, 53.50)	30.00 (17.75, 54.00)	8.50 (4.25, 19.50)	***<0.0001***
**TJC (n)**	0.00 (0.00, 0.00)	2.50 (1.00, 7.50)	15.50 (8.25, 20.75)	3.50 (2.00, 5.00)	12.00 (5.25, 15.25)	***<0.0001***
**SJC (n)**	0.00 (0.00, 0.00)	0.50 (0.00, 15.25)	5.00 (2.75, 16.25)	4.00 (1.00, 14.00)	5.50 (1.50, 9.25)	***<0.0001***
**PainVAS - avg. 4 weeks (mm)**	5.00 (0.00, 15.00)	40.00 (23.75, 50.00)	70.00 (60.00, 81.25)	45.00 (30.00, 50.00)	62.50 (60.00, 70.00)	***<0.0001***
**PainVAS - current (mm)**	0.00 (0.00, 1.50)	20.00 (0.00, 45.00)	60.00 (60.00, 80.00)	30.00 (10.00, 60.00)	60.00 (50.00, 80.00)	***<0.0001***
**PGA (mm)**	-	30.00 (20.00, 45.00)	55.00 (47.50, 66.25)	40.00 (30.00, 60.00)	55.00 (30.00, 62.50)	***0.0026***
**PTGA (mm)**	-	30.00 (17.50, 42.50)	65.00 (57.50, 80.00)	50.00 (50.00, 70.00)	70.00 (47.50, 80.00)	***0.0008***
**PTGH (mm)**	20.00 (10.00, 25.00)	45.00 (20.00, 60.00)	65.00 (50.00, 72.50)	35.00 (20.00, 50.00)	65.00 (60.00, 80.00)	***<0.0001***
**MS (min)**	-	5.00 (1.13, 33.75)	15.00 (8.75, 60.00)	10.00 (0.00, 15.00)	5.00 (0.00, 21.25)	***<0.0001***

Compared populations:
ALL RA patients including subgroups and HCs
Analysis type:
Data are presented as median (interquartile range, IQR; 25th–75th percentile). Group differences were assessed using the Kruskal–Wallis test, with p-values < 0.05 considered statistically significant.
Abbreviations:
D2T RA, difficult-to-treat rheumatoid arthritis; DAS28-CRP: Disease Activity Score based on 28 joints and C-reactive protein; DAS28-ESR: Disease Activity Score based on 28 joints and erythrocyte sedimentation rate; CRP: C-reactive protein (mg/l); ESR: erythrocyte sedimentation rate (mm/hr); TJC: tender joint count (n); SJC: swollen joint count (n); PainVAS avg. 4 weeks: average pain intensity over the past 4 weeks assessed on a visual analogue scale (VAS, mm); PainVAS current: current pain intensity on VAS (mm); PGA: patient's global assessment of disease activity (mm); PTGA: patient's global assessment of arthritis (mm); PTGH: patient's global assessment of general health (mm); MS: morning stiffness duration (minutes).

Supplementary Material 6 – Clinical feature details (stratified by study group and recruitment site)

	Rheumatoid arthritis patients				Healthy controls		p-value
	D2T RA		Non-D2T RA				
	Pécs	Budapest	Pécs	Budapest	Pécs	Budapest	
Age (mean+SD)	56.40+8.73	53.95+14.54	62.67+3.79	57.33+13.82	51.00+9.87	50.85+15.46	0.5139
Gender (female, n)	8	19	3	13	8	14	0.4281
Current smoker	3	4	1	3	0	4	0.4015
Former smoker	3	7	1	6	2	4	0.7517
Allergies	6	9	3	6	5	12	0.3787
Diabetes	1	3	0	1	1	0	0.5781
Hypertension	4	11	2	6	2	4	0.1672
COPD	1	2	0	2	0	0	0.5004
Asthma	0	1	0	1	0	2	0.8789
Fibromyalgia	3	1	0	1	0	0	0.0493
Neuropathic pain - possible	2	8	0	5	0	0	0.0033
Neuropathic pain - likely	1	4	0	0	0	0	0.1018
DAS28-CRP (median+IQR)	5.32 (4.70, 5.60)	4.55 (3.82, 5.15)	4.74 (4.16, 5.18)	3.18 (2.68, 4.28)	-	-	<0.0001
DAS28-ESR(median+IQR)	5.42 (4.42, 6.14)	4.03 (3.76, 6.27)	4.035 (3.79, 4.28)	2.53 (2.18, 4.96)	-	-	<0.0001
CRP (mg/l) (median+IQR)	7.70 (1.68, 26.08)	8.07 (0.66, 13.16)	1.80 (0.60, 2.50)	1.80 (0.41, 4.34)	1.80 (0.90, 3.40)	0.85 (0.54, 1.35)	0.0188
ESR (mm/hr) (median+IQR)	33.50 (13.75, 59.50)	22.00 (10.25, 30.00)	8.50 (6.00, 11.00)	13.00 (3.00, 21.50)	8.00 (2.00, 12.00)	3.00 (2.00, 6.00)	<0.0001
TJC (n) (median+IQR)	12.50 (7.50, 16.00)	8.00 (4.00, 16.00)	12.00 (2.00, 23.00)	2.00 (1.00, 5.00)	0.00 (0.00, 0.00)	0.00 (0.00, 1.75)	<0.0001
SJC (n) (median+IQR)	13.00 (5.75, 16.25)	3.00 (0.50, 8.00)	19.00 (14.00, 20.00)	0.00 (0.00, 5.00)	0.00 (0.00, 0.00)	0.00 (0.00, 0.00)	<0.0001
PainVAS - avg. 4 weeks (mm) (median+IQR)	60.00 (45.00, 61.25)	60.00 (50.00, 70.00)	50.00 (50.00, 50.00)	40.00 (20.00, 50.00)	2.50 (0.00, 20.00)	7.50 (0.00, 10.00)	<0.0001
PainVAS - current (mm) (median+IQR)	60.00 (40.00, 80.00)	60.00 (30.00, 75.00)	60.00 (30.00, 60.00)	10.00 (0.00, 40.00)	0.00 (0.00, 0.00)	0.00 (0.00, 10.00)	<0.0001
MS (min) (median+IQR)	7.50 (0.00, 42.50)	10.00 (5.00, 17.50)	5.00 (1.50, 60.00)	5.00 (0.00, 30.00)	0.00 (0.00, 0.00)	0.00 (0.00, 0.00)	<0.0001
PGA (mm) (median+IQR)	50.00 (40.00, 60.00)	60.00 (32.50, 67.50)	30.00 (30.00, 30.00)	30.00 (20.00, 50.00)	-	-	0.0031
PTGA (mm) (median+IQR)	70.00 (57.50, 80.00)	60.00 (45.00, 77.50)	70.00 (30.00, 75.00)	25.00 (10.00, 40.00)	-	-	0.0001
PGH (mm) (median+IQR)	55.00 (20.00, 82.50)	60.00 (45.00, 70.00)	30.00 (10.00, 50.00)	50.00 (20.00, 60.00)	-	-	0.2635

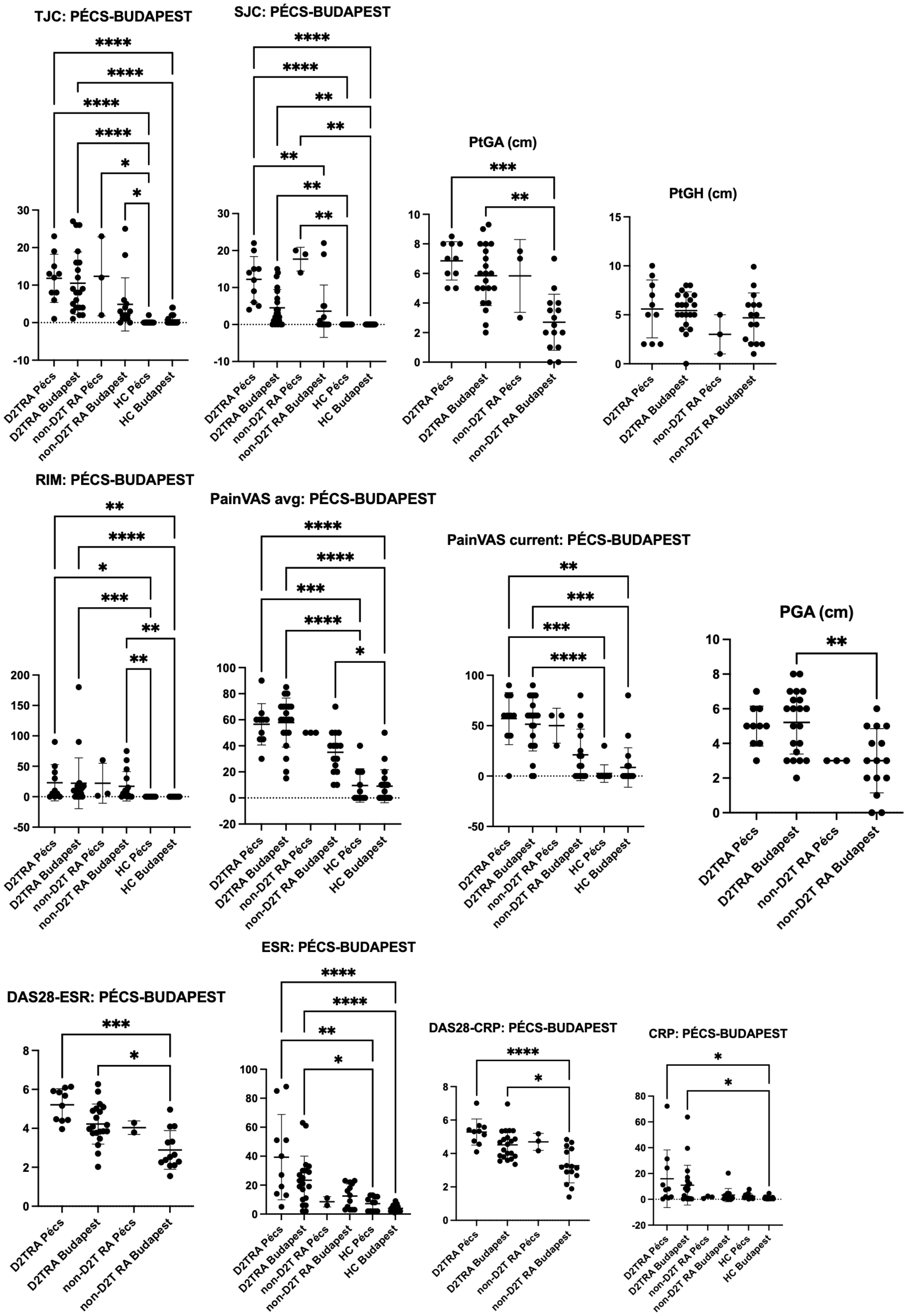

Compared populations:

ALL RA patients subgrouped based on study groups and inclusion sites

Analysis type:

Group differences were assessed using Fisher's exact test for categorical variables and ANOVA or the Kruskal–Wallis test for continuous variables, depending on data distribution.

Abbreviations:

D2T RA, difficult-to-treat rheumatoid arthritis; DAS28-CRP: Disease Activity Score based on 28 joints and C-reactive protein; DAS28-ESR: Disease Activity Score based on 28 joints and erythrocyte sedimentation rate; CRP: C-reactive protein (mg/l); ESR: erythrocyte sedimentation rate (mm/hr); TJC: tender joint count (n); SJC: swollen joint count (n); PainVAS avg. 4 weeks: average pain intensity over the past 4 weeks assessed on a visual analogue scale (VAS, mm); PainVAS current: current pain intensity on VAS (mm); PGA: patient's global assessment of disease activity (mm); PTGA: patient's global assessment of arthritis (mm); PTGH: patient's global assessment of general health (mm); MS/RIM: morning stiffness duration (minutes).

Supplementary Material 7 – Results of baseline fMRI alterations (data for Fig. 3 a-e, g-i, k)

Brain Area / Functional connections	Analysis Type	Voxel coordinates	Size (in voxels)	p value (FDR	Result
Posterior Cingulate Cortex (PCC)	ICC	-4 -28 32	285	0.000004	reduced in all RA patients compared to HCs
Bilateral Sensory Cortices	IHC	±24 -28 72	109	0.000188	reduced in all RA patients compared to HCs
Bilateral PCC	IHC	±6 -30 40	81	0.000989	reduced in all RA patients compared to HCs
Bilateral Insula/Parietal Operculum/Planum Polare	IHC	-52 -6 0	36	0.029	reduced in all RA patients compared to HCs
Gyri	IHC	±22 -50 -4	48	0.009371	reduced in all RA patients compared to HCs
Bilateral Middle Temporal Gyri (MTG)	IHC	±52 10 -26	61	0.003149	reduced in all RA patients compared to HCs
Left Poscentral Gyrus (PCG)-Right Thalamus	SBC	12 -4 4	146	0.00112	increased in all RA patients comapred to HCs
PCG – Left Angular Gyrus (AG)	SBC	-50 -50 42	180	0.000364	increased in all RA patients comapred to HCs
PCG – Contralateral Somatosensory Cortex	SBC	18 -40 70	686	<0.000001	reduced in all RA patients compared to HCs
PCG – Right Temporal Pole (TP)	SBC	32 12 -30	131	0.001713	reduced in all RA patients compared to HCs
PCG – Left TP	SBC	-28 14 -36	69	0.03114	reduced in all RA patients compared to HCs
PCG – Bilateral Hippocampal Formation (CA1, HATA, Subiculum)	SBC	-24 -10 -20 / 16 -8 -18	70 / 57	0.03114 / 0.04902	reduced in all RA patients compared to HCs
PCC	ICC	-4 -26 34	111	0.003892	reduced in non-D2T RA and D2T RA patients compared to HCs
Lateral Occipital Cortex (LOC)	fALFF	44 -80 -2	215	<0.000001	reduced in non-D2T RA and D2T RA patients compared to HCs
Lingual Gyrus	fALFF	-2 -88 -10	72	0.000358	reduced in non-D2T RA and D2T RA patients compared to HCs
Caudate	fALFF	-10 0 16	43	0.006876	reduced in non-D2T RA and D2T RA patients compared to HCs
Middle Frontal Gyrus (MFG)	fALFF	34 24 54	48	0.004163	reduced in non-D2T RA and D2T RA patients compared to HCs
Medial Prefrontal Cortex (MPFC)	fALFF	6 46 -22	24	0.04767	reduced in non-D2T RA and D2T RA patients compared to HCs
Postcentral Gyrus (PCG)	fALFF	-12 -40 80	63	0.000814	reduced in non-D2T RA and D2T RA patients compared to HCs
Frontal Pole (FP)	fALFF	8 10 56	35	0.017836	reduced in non-D2T RA and D2T RA patients compared to HCs
Frontal Operculum	fALFF	44 24 10	26	0.0003	reduced in D2T RA comapred to non-D2T RA patients and to HCs
Left MFG	fALFF	-54 10 38	23	0.0444	reduced in D2T RA comapred to non-D2T RA patients and to HCs
Right MFG	fALFF	44 4 50	27	0.0014	reduced in D2T RA comapred to non-D2T RA patients and to HCs
Superior Frontal Gyri (SFG)	fALFF	8 34 58	35	0.0001	reduced in D2T RA comapred to non-D2T RA patients and to HCs
LOC (Left)	fALFF	-22 -90 18	174	0.0033	reduced in D2T RA comapred to non-D2T RA patients and to HCs
LOC (Right)	fALFF	46 -78 -6	215	0.0472	reduced in D2T RA comapred to non-D2T RA patients and to HCs
Left Occipital Pole (OP)	fALFF	-20 -98 18	163	0.043	reduced in D2T RA comapred to non-D2T RA patients and to HCs
Right OP	fALFF	24 -96 8	491	0.0185	reduced in D2T RA comapred to non-D2T RA patients and to HCs
Left PCG	fALFF	-14 -40 78	63	0.036	reduced in D2T RA comapred to non-D2T RA patients and to HCs
Right PCG	fALFF	12 -42 76	28	<0.0001	reduced in D2T RA comapred to non-D2T RA patients and to HCs
bilateral PCG – several temporal, parietal frontal regions	ROI-to-ROI	N.A.	N.A.	0.005035	significantly different FCS was observed between the bilateral PCG and several temporal, parietal, and frontal regions in subgroup analysis (ANOVA) LI-LP vs. HI-LP vs. LI-HP vs. HI-HP
PCG – MPFC	SBC	-8 36 -10	64	0.008115	significantly different FCS was observed between the left PCG and MPFC in subgroup analysis (ANOVA) LI-LP vs. HI-LP vs. LI-HP vs. HI-HP
PCG – bilateral SFG	SBC	-22 34 40 / 26 22 56	138 / 70	0.00023 / 0.007943	significantly different FCS was observed between the left PCG and SFG in subgroup analysis (ANOVA) LI-LP vs. HI-LP vs. LI-HP vs. HI-HP
PCG – bilateral MTG	SBC	-66 -24 -10 / 66 -26 -8	63 / 129	0.008115 / 0.00023	significantly different FCS was observed between the left PCG and bilateral MTG in subgroup analysis (ANOVA) LI-LP vs. HI-LP vs. LI-HP vs. HI-HP

Compared populations: Indicated in the Result column in each comparison. Analysis type: Indicated in the Analysis type column in each comparison. Abbreviations: ACC, Anterior Cingulate Cortex; AG, Angular Gyrus; D2T, difficult-to-treat; fALFF, fractional amplitude of low-frequency fluctuations; FP, Frontal Pole; FOP, Frontal Opercular Area; HC, healthy controls; HI, high inflammation; HP, high pain; ICC, intrinsic connectivity; IHC, interhemispheric connectivity; LI, low inflammation; LOC, Lateral Occipital Cortex; LP, low pain; MFG, Middle Frontal Gyrus; MPFC, Medial Prefrontal Cortex; MTG, Middle Temporal Gyrus; MVPA, multivariate pattern analysis; OFC, Orbitofrontal Cortex; OP, Occipital Pole; PCC, Posterior Cingulate Cortex; PCG, Postcentral

Gyrus; RA, rheumatoid arthritis; ROI, region of interest; SBC, seed based connectivity; SFG, Superior Frontal Gyrus; SI-SII, Primary and Secondary Somatosensory Cortex; TP, Temporal Pole

Supplementary Material 8 - Results of pairwise analysis for baseline fMRI measurements (data for Fig. 3 f,j,l)

Brain Area / Functional connections	Analysis type	Voxel coordinates	Size (in voxels)	p value (FDR corrected)	Result
PCC	ICC	-4 -26 34	111	0.0518	reduced in non-D2T RA patients compared to HCs
PCC	ICC	-4 -26 34	111	<0.0001	reduced in D2T RA patients compared to HCs
bilateral PCG – several temporal, parietal frontal regions	SBC	N.A.	N.A.	0.002	non-D2T RA subgroup showed reduced FCS as compared to the HI-LP subgroup of D2TRA
PCG – MPFC	SBC	-8 36 -10	64	0.05	non-D2T RA (LI-LP) subgroup showed reduced FCS as compared to the HI-HP subgroup of D2TRA
PCG – bilateral SFG	SBC	-22 34 40 / 26 22 56	138 / 70	0.0001-0.006	Low inflammation subgroups (LI-LP and LI-HP) showed reduced functional connectivities between right PCG and bilateral SFG as compared to high inflammation subgroups (HI-LP, HI-HP). Significant differences are depicted on the corresponding bar graph.
PCG – bilateral MTG	SBC	-66 -24 -10 / 66 -26 -8	63 / 129	0.0001-0.0018	HI-LP subgroup showed the highest functional connectivities between right PCG and bilateral MTG among all subgroups. Significant differences are depicted on the corresponding bar graph.

Compared populations: Indicated in the Result column in each comparison. Analysis type: Indicated in the Analysis type column in each comparison. Abbreviations: D2T, difficult-to-treat; HC, healthy controls; HI, high inflammation; HP, high pain; ICC, intrinsic connectivity; LI, low inflammation; LP, low pain; MPFC, Medial Prefrontal Cortex; MTG, Middle Temporal Gyrus; OFC, Orbitofrontal Cortex; OP, Occipital Pole; PCC, Posterior Cingulate Cortex; PCG, Postcentral Gyrus; RA, rheumatoid arthritis; SBC, seed-based connectivity; SFG, Superior Frontal Gyrus

Supplementary Material 9 - Results of fMRI measurements summarizing the effect of acute pain on brain connectivity (data for Fig. 4)

Brain Area / Functional connections	Analysis Type	Voxel coordinates	Size (in voxels)	p value (FDR corrected)	Result
PCC	ICC	4 -28 30	168	0.008991	increased in all RA patients compared to HCs
MPFC	ICC	10 50 -6	132	0.003453	increased in all RA patients compared to HCs
Left AG	ICC	-52 -52 16	198	0.012653	increased in all RA patients compared to HCs
Left Intracalcarine Cortex	ICC	-20 -78 12	53	0.0315432	increased in all RA patients compared to HCs
Left and Right Sensory Areas	IHC	±18 -34 70	215	0.000007	increased in all RA patients compared to HCs
PCC	IHC	±6 -54 36	68	0.043734	increased in all RA patients compared to HCs
MTG	IHC	±56 -13 5	41	0.0397545	increased in all RA patients compared to HCs
PCG I – Bilateral SI-SII Areas (mostly right)	SBC	-16 -40 68 / 16 36 66	301 / 1053	<0.0001	increased in all RA patients compared to HCs
PCC, FP, AG, Cerebellum	ICC	N.A.	N.A.	0.000304-0.044322	reduced or slightly changed in non-D2T RA patients and HCs, increased in D2T RA patients
Left Sensorimotor Area	LCOR	-18 -30 72	98	0.03181	reduced in non-D2T RA patients and HCs, increased in D2T RA patients
LI/FOP	ICC	-42 16 6	43	0.039831	high inflammation subgroups (HI-LP, HI-HP) show significant increase as compared to the subgroups with low inflammation
LI/FOP – Left Insula	SBC	-36 -2 6	83	0.007435	high inflammation subgroups (HI-LP, HI-HP) show significant increase as compared to the subgroups with low inflammation
LI/FOP – Right Caudate	SBC	8 12 10	63	0.017668	high inflammation subgroups (HI-LP, HI-HP) show significant increase as compared to the subgroups with low inflammation
right OFC – PCC	SBC	4 -18 42	46	0.035573	high inflammation subgroups (HI-LP, HI-HP) show significant increase as compared to the subgroups with low inflammation
right OFC – MPFC/Paracingulate Cortex	SBC	-4 38 28	59	0.042036	high inflammation subgroups (HI-LP, HI-HP) show significant increase as compared to the subgroups with low inflammation
right OFC – SFG	SBC	0 16 56	492	<0.000001	high inflammation subgroups (HI-LP, HI-HP) show significant increase as compared to the subgroups with low inflammation
right OFC – MTG	SBC	66 -24 -8	210	0.000002	high inflammation subgroups (HI-LP, HI-HP) show significant increase as compared to the subgroups with low inflammation

Compared populations: Indicated in the Result column in each comparison. Analysis type: Indicated in the Analysis type column in each comparison. Abbreviations: AG, Angular Gyrus; D2T, difficult-to-treat;; HC, healthy controls; HI, high inflammation; HP, high pain; ICC, intrinsic connectivity; IHC, interhemispheric connectivity; ITG, Inferior Temporal Gyrus; LI, low inflammation; LI/FOP, Left Insula/Frontal Opercular Area LP, low pain; MFG, Middle Frontal Gyrus; MPFC, Medial Prefrontal Cortex; MTG, Middle Temporal Gyrus; MVPA, multivariate pattern analysis; OFC, Orbitofrontal Cortex; OP, Occipital Pole; PCC, Posterior Cingulate Cortex; PCG, Postcentral Gyrus; RA, rheumatoid arthritis; SBC, seed - based connectivity; SFG, Superior Frontal Gyrus

Supplementary Material 10 - Results of fMRI measurements summarizing the relations of the Rorschach probability index (RPI) and brain connectivity at baseline (a) and after acute pain stimulation (b) (data for Fig.5 d-g)

a) Baseline resting-state connectivity pattern is associated with the Rorschach Probability Index (RPI)

Brain Area / Functional connections	Analysis Type	Voxel coordinates	Size (in voxels)	p value (FDR corrected)	Result
Left SFG	MVPA	-26 20 60	221	<0.000001	MVPA analysis identified the left SFG as seed
Left SFG – Left Bilateral PCG / SMG	SBC	-50 -40 44	789	<0.000001	Connections of the seed region show a characteristic profile associated with the value of the RPI
Left SFG – Right Bilateral PCG / SMG	SBC	50 -34 44	715	<0.000001	
Left SFG – PCC	SBC	-4 -52 34	141	0.000663	
Left SFG – MPFC	SBC	-4 58 20	828	<0.000001	
Left SFG – Left AG	SBC	-52 -56 22	543	<0.000001	
Left SFG – Right AG	SBC	62 -58 26	92	0.005499	
Left SFG – Left MTG	SBC	-64 -18 -12	238	0.000037	
Left SFG – Right MTG	SBC	64 -4 -28	166	0.000319	
Left SFG – FP	SBC	-10 56 38	257	0.000023	
Left SFG – Left TP	SBC	-42 4 -38	91	0.005499	
Left SFG – Right TP	SBC	48 18 -40	193	0.000154	
Left SFG – Left OFC	SBC	-34 28 -16	221	0.00006	
Left SFG – Right OFC	SBC	38 32 -14	151	0.000459	

b) Resting-state connectivity pattern after acute pain stimulation is associated with the RPI

Brain Area / Functional connections	Analysis Type	Voxel coordinates	Size (in voxels)	p value (FDR corrected)	Result
Left SFG – Left PCG	SBC	-50 -24 38	54	0.039461	Acute painful stimulus significantly changes the connection of the seed region showing a characteristic profile associated with the value of the RPI
Left SFG – Right PCG	SBC	54 -30 52	78	0.012869	
Left SFG – MTG	SBC	54 -2 -26	74	0.01287	

Compared populations: Indicated in the Result column in each comparison. Analysis type: Indicated in the Analysis type column in each comparison. Abbreviations: AG, Angular Gyrus; FP, Frontal Pole; LOC, Lateral Occipital Cortex; LP, low pain; MPFC, Medial Prefrontal Cortex; MTG, Middle Temporal Gyrus; MVPA, multivariate pattern analysis; OFC, Orbitofrontal Cortex; OP, Occipital Pole; PCC, Posterior Cingulate Cortex; PCG, Postcentral Gyrus; RA, rheumatoid arthritis; RPI, Rorschach probability index; SBC, seed-based connectivity; SFG, Superior Frontal Gyrus; SMG, supramarginal gyrus; TP, Temporal Pole

Supplementary Material 11 - Results of fMRI measurements summarizing the relation of NRG1 expression with baseline resting-state functional connectivity patterns (data for Fig.6 l)

NRG1 expression is associated with a characteristic baseline resting-state connectivity pattern

Brain Area / Functional connections	Analysis Type	Voxel coordinates	Size (in voxels)	p value (FDR corrected)	Result
Right MFG	MVPA	54 22 34	30	0.003829	MVPA analysis identified the right MFG as seed
Right ITG	MVPA	56 -12 -42	76	0.000008	MVPA analysis identified the ITG as seed
Right MFG – Left PCG	SBC	-22 -52 60	457	<0.000001	Higher NRG1 expression was associated with enhanced functional connectivity
Right MFG – Right PCG	SBC	14 -50 54	230	<0.000001	
Right MFG – Left Insular/Opercular Cortices	SBC	-50 0 0	479	<0.000001	
Right MFG – Right Insular/Opercular Cortices	SBC	46 -36 20	1172	<0.000001	
Right MFG – Large cluster encompassing both the ACC and PCC	SBC	-4 -16 52	805	<0.000001	
Right MFG – Supplementary Motor Cortex	SBC	2 -18 74	315	0.000002	
Right MFG – Left Superior LOC	SBC	-32 -86 42	165	0.000381	Higher NRG1 expression was associated with reduced functional connectivity
Right ITG – Left PCG / SMG	SBC	-62 -34 34	979	<0.000001	Higher NRG1 expression was associated with enhanced functional connectivity
Right ITG – Right PCG / SMG	SBC	56 -34 38	956	<0.000001	
Right ITG – ACC	SBC	-4 4 52	630	<0.000001	
Right ITG – Right SFG	SBC	32 -2 60	389	<0.000001	
Right ITG – Right Superior LOC	SBC	22 -70 44	692	<0.000001	
Right ITG – FP/MPFC	SBC	-2 52 0	1813	<0.000001	Higher NRG1 expression was associated with reduced functional connectivity
Right ITG – PCC/Precuneus	SBC	-4 -54 14	1004	<0.000001	
Right ITG – Left Superior LOC/AG	SBC	-42 -76 42	816	<0.000001	
Right ITG – Left MTG	SBC	-60 -16 -2	345	<0.000001	

Compared populations: Indicated in the Result column in each comparison. Analysis type: Indicated in the Analysis type column in each comparison. Abbreviations: ACC, Anterior Cingulate Cortex; FP, Frontal Pole; ITG, Inferior Temporal Gyrus; LOC, Lateral Occipital Cortex; MFG, Middle Frontal Gyrus; MPFC, Medial Prefrontal Cortex; MTG, Middle Temporal Gyrus; MVPA, multivariate pattern analysis; PCC, Posterior Cingulate Cortex; PCG, Postcentral Gyrus; SBC, seed-based connectivity; SFG, Superior Frontal Gyrus

Supplementary Material 12 - Comparison based on Rorschach indicators between D2T RA and NON D2T RA patients (Detailed explanation for Fig.5.b)

Variables	RA *mean*	RA *SD*	D2T *mean*	D2T *SD*	Effect size	p-value
Number of responses	23,67	9,665	19	8,753	0,511	0,027
Number of responses to Plates I–VII	15,33	6,055	12,53	5,629	0,482	0,037
Number of Gkomb responses	0,424	0,561	0,0638	0,247	0,886	< 0,001
G values in FT	11,21	4,328	8,234	3,073	0,817	0,001
Number of F+ responses	7,242	3,491	5,213	3,127	0,618	0,008
Number of B+ responses	1,788	1,431	1,149	0,978	0,539	0,020
Number of FFb+ responses	1,758	1,921	0,809	1,173	0,622	0,008
Number of FbF responses	2,303	1,403	1,489	1,14	0,648	0,005
Value of the right side of the ÉT	3,97	2,905	2,521	1,658	0,642	0,006
Sociability-index	5,03	3,423	2,617	2,419	0,839	< 0,001
Regression-index	161,03	38,74	137,3	30,33	0,697	0,003
Signs of suicidal risk – baseline marker	0,121	0,331	0	0	0,569	0,014
Number of [Obj] contents	1,485	1,302	0,83	1,049	0,565	0,015
Number of [Tűz] contents	0,394	0,659	0,128	0,337	0,537	0,020
Number of [Szcéna] responses	0,182	0,528	0	0	0,537	0,020
Number of [Füst] contents	0,152	0,442	0	0	0,534	0,021
Number of [Víz] contents	1,182	1,236	0,468	0,804	0,71	0,002
Number of [Ornamentika] contents	0,424	0,663	0,106	0,312	0,651	0,005
Number of [Szex] contents	0,606	0,827	0,234	0,598	0,53	0,022
Total content score	35,58	13,7	26,55	12,13	0,705	0,003
Number of content circles	14,09	4,34	10,7	3,764	0,845	< 0,001
Right side of M:Md	11,65	20,15	3,153	11,76	0,538	0,020

Number of V2 responses	2,303	1,51	1,413	1,343	0,629	0,007
Number of V3 responses	3,97	2,229	2,766	1,925	0,586	0,012
Value of V2–V3 index	6,273	2,992	4,149	2,579	0,77	0,001
Number of special reaction: Értékelés ('Evaluation')	2,333	2,582	1,085	1,38	0,635	0,006
Number of special reaction: EOa	0,121	0,331	0	0	0,569	0,014
Number of special reaction: Kettőzés ('Doubling')	0,212	0,485	0,0426	0,204	0,487	0,035
Number of special reaction: Perspektíva ('Perspective')	0,303	0,684	0,0638	0,247	0,5	0,030
Number of special reaction: Színhívás ('Color call')	0,212	0,545	0,0213	0,146	0,519	0,025
Number of special reaction: Színdicséret ('Color praise')	0,485	1,121	0,0638	0,247	0,565	0,015
Hegedűs–Schizophrenia + signs	2,606	3,427	4,106	2,352	-0,527	0,023
Hegedűs–Schizophrenia - signs	3,121	2,497	5	2,941	-0,679	0,004
T percentage	38,94	20,99	50,27	18,86	-0,573	0,014
Number of special reaction: Jelentéstudat ('Meaning awareness')	0,0303	0,174	0,574	0,927	-0,757	0,001
Number of special reaction: Zavar ('Confusion')	0,727	1,18	1,489	1,412	-0,577	0,013

- The non-D2T group produced a higher total number of responses, indicating a broader and richer range of interests and greater motivation to perform well during the test.
- The non-D2T group provided more responses to the first seven plates, indicating greater activity on emotionally neutral plates, suggesting that they are more motivated to respond even in emotion-free or mildly anxiety-provoking situations.
- The non-D2T group provided more G-combined responses, indicating a higher capacity for abstraction and a better ability to organize life beyond immediate concerns.
- The non-D2T group produced more G responses in FT, indicating a tendency toward more comprehensive, integrative perception and abstract thinking.
- The non-D2T group provided more F+ responses, indicating a higher level of mental regulation and a stronger use of cognitive control in their behavior.
- The non-D2T group produced more B+ responses, showing a stronger internal inhibitory system against impulses, allowing more conventional participation in society.
- The non-D2T group gave more FFb+ responses, reflecting more mature emotional regulation.
- The non-D2T group produced more FbF responses, suggesting that emotions and subjectivity still strongly influence their behavior, but are accompanied by conscious cognitive control.
- The non-D2T group scored higher on the right side of the ÉT, indicating more extraversion and a stronger orientation toward external stimuli.
- The non-D2T group had a higher sociability index, suggesting stronger personality traits that make them good social partners.

- The non-D2T group showed a higher number of suicidal base signs, indicating a greater risk of self-destructive behavior.
- The non-D2T group gave more Obj content responses, reflecting higher ambition.
- The non-D2T group produced more [Tűz] (‘Fire’) content responses, indicating stronger passion in action and greater interest in their social environment.
- The non-D2T group gave more [Szcéna] (‘Scene’) responses, suggesting more vivid imaginative activity and a richer fantasy life.
- The non-D2T group produced more [Füst] (‘Smoke’) content responses, indicating stronger struggle against experiencing anxiety.
- The non-D2T group gave more [Víz] (‘Water’) content responses, reflecting impulsivity and lack of inhibition.
- The non-D2T group gave more [Ornamentika] content responses, suggesting greater attention to external judgment and stronger drive to conform.
- The non-D2T group gave more [Szex] content responses, which normally average 1–2 in healthy individuals. Since both groups scored below this level, the D2T group can be described as much less interested and less motivated in this domain.
- The non-D2T group produced a greater total number of content responses, indicating greater creativity, ambition, and elaboration capacity during testing.
- The non-D2T group gave more content circles, reflecting a more varied imaginative flow and less reliance on stereotypical associations.
- In the non-D2T group, the proportion of Md (human detail) responses within the M:Md ratio was higher, suggesting a more inhibited disposition and greater difficulty in opening up.
- The non-D2T group produced more V2–V3 responses, indicating stronger conformity based on internalized rules.
- The non-D2T group gave more [Értékelés] special responses, suggesting a stronger need to express emotions.
- The non-D2T group produced more [EQa] special responses, showing a more colorful self-image, with greater abstraction and synthesis capacity.
- The non-D2T group gave more [Kettőzés] special responses, indicating a risk of behavioral regression.
- The non-D2T group produced more [Perspektíva] special responses, reflecting stronger compensatory ability and a generally more favorable prognosis.
- The non-D2T group gave more [Színhívás] special responses, suggesting stronger attempts to compensate for mood lability with emotional expression.
- The non-D2T group produced more [Színdicséret] special responses, again reflecting higher compensatory effort against mood instability.
- The D2T group showed more positive and negative signs on the Hegedűs schizophrenia scale, suggesting a more schizoid personality tendency.
- The D2T group had a higher T%, indicating excessive conformity and rigidity, in contrast to the non-D2T group, which showed average levels of conventionality.
- The D2T group gave more [Jelentéstudat] special responses, indicating hesitation before intellectual tasks and mental narrowing.
- The D2T group produced more [Zavar] special responses, suggesting stronger fear of failure or unsuccessfulness during the test.

Supplementary Material 13- Description of the indicators that make up the Rorschach Probability Index (RPI)

The Rorschach notation and interpretation system we use has been in development in Hungary since the 1940s and is based on a large body of statistical and clinical research. Its basic textbook is Ferenc Mérei (2002) Rorschach Test . The Hungarian notation system is primarily related to the CS notation convention of Exner et al. (2022) used in the USA, but its elements are also easily understandable for those familiar with the modern RPAS system.

The formula that makes up the RPI includes the following indicators:

$$RPI = 1 - \frac{2{,}72^{0{,}517Gzw+0{,}082F++0{,}178BF++0{,}3FFb++0{,}22FHd++0{,}583HdF+0{,}333Td+0{,}299V2-5{,}107}}{1 + 2{,}72^{0{,}517Gzw+0{,}082F++0{,}178BF++0{,}3FFb++0{,}22FHd++0{,}583HdF+0{,}333Td+0{,}299V2-5{,}107}}$$

1. Gzw

A whole response (G) in which both the stain and the white background (sign zw) have meaning for the respondent.

Criteria:

The approach is whole controlled (the content shows that the test person has grasped both the blot and the white details that went into the response).

The white details have a meaning value (expressed spontaneously by the subject or in response to a question in the post-test control questionnaire).

Interpretation : Combining the stain and the background is an elaboration solution. An adaptation that reconciles two simultaneous stimuli. Those who can do this are used to difficult adaptations. The person responding to Gzw is usually in severe conflict with his environment, in a state of defiance, but can adapt to other situations and tasks.

In a high-quality protocol (with F+ and B occurrences), a lot of Gzw indicates overview, effective thinking and independence.

Poor Gzw responses in a low-quality protocol indicate inappropriate, poorly administered aggression, seen in skizoform symptomatology.

What it also means: carrying a conflict of conscience, the unresolved nature of the conflict, which maintains tension.

If there are many, then a self-punishing conscience. If there are few, then you have a weak conscience function.

2. F+

Responses where the meaning is determined by the shape of the spot alone are given this mark.

The only **criterion** is that the examinee could have given this report even if the area marked in the field had only a contour - neither colour nor shading plays a role in the answer.

Criteria for the degree of advection (+, +- or -)

1. To what extent does the stain match or resemble the objective sample?
2. If the semantic and semantic form gives the examiner a sense of similarity evidence, it is automatically given an + adverbial signal,
3. The frequency of responses also plays a role (if we are uncertain about similarity, statistical frequency is in favor of addition).

Interpretation: realistic perception of reality, what you see is valid, not subjectively altered. Mental break, discipline, because from these F+s we calculate mental control F1%.

High F%, i.e. many F+ is rigid mental control, low F% (few F+) is weak mental control, reality is impaired by subjective factors in thinking

3. BF+

If the respondent gives an animal response in which he/she expresses that the animal is moving (jumping, climbing, flying, swimming, running, playing ball, etc.), then give this signal.

A rare exception is the anthropomorphic movement of some animals walking on two legs in response B (human movement response signal).

The degree of adaptation is also indicated here according to the criteria above.

Interpretation: very good sign, motivation ready to be implemented, intention easily turns into implementation.

BF (realistic animal moving) is the "antechamber" to the (big) B (human movement) in the picture.

Lots of BF+ ready to go implementation tension in the personality (towards something), few BF reflect immature will forces, no internal readiness, not enough motivation to act.

4. FFb+

Meaning is determined by form, but color is also a guide to perception.

The contours of the area captured are not enough to formulate a response - color is needed to give meaning, but its role is secondary.
The **degree of adequacy** applies only to the formal element of the response.
Interpretation: anger with the need for mental brake (control), mental control works well. Mental control overrides subjectivity in emotion regulation, means mature emotion regulation. It is optimal to have a lot of this because subjectivity does not drive the individual away (then FbF would be a lot). High FFb+ number is a sign of mature emotion regulation, little FFb+ or if none, emotions are dominating in a way that determines thinking and judgement.

5. FHd+
The area is outlined and structured according to the meaning - undoubtedly a form control.
Here, shading also plays a role in the meaning, because it makes the answer more precise and adds a further element (usually an additional element) to the answer.
Interpretation: superego fear: conscience control. **Anxiety is dominated by reality control, a good conscience function.** If there are many, it indicates a severe conscience.
High FHd+ number very strongly reality-driven tension and anxiety management, ability to deal with realistic fear (correctly assess dangerous situations). If little or none, then fear is in control, phobic tendency, or tends toward elementary anxiety management.

6. HdF:
Shading driven shape response. Light, dark tones and other shape elements can be substituted.
It usually occurs from the first to the seventh board.
The emotional element predominates over the rational. The examinee is in a state of constant anxious readiness, and this intangible tension is always objectified and occasionally rationalized. A frequent response to a healthy protocol shows occasional, object-found anxiety. Indicates neurosis in more pathological protocol.
Interpretation: fear of ego: anxiety dominates the intellectual brake. The emotional element predominates over the rational. The examinee attaches all anxiety tension to something, does not let it float freely, and always attaches this intangible tension to an object, rationalizing it occasionally. Frequent response is a healthy protocol and indicates a transfer of occasional anxiety to the situation. If there is a lot of it, specifically there is a phobic symptom of the person is very afraid of something. It also indicates phobia, anxiety found on the object. It is involved in the development of phobia if there is a lot of it. If there is little or none of it, then the transference of the anxiety to something, object, thing does not work.

7. Td
This is used to indicate responses in which animal body parts occur.
The same restrictions apply as for the Md (human body part) responses (animal internal organs or bones are placed in the anatomical responses, genital responses in the sexual group).
Mark when the whole animal (T) has not yet been pronounced by the subject.
After T (whole animal answer) the list of animal body parts is not marked in the content column.
Interpretation: an element belonging to behavioral restraint, because from this and the T responses together we calculate the T%, which indicates behavioral control. But weaker control content, "weaker restraint", less strict control trait, which is why we say that psychopaths have more Td than T. Let there be more T than Td, this is the criterion. Less Td indicates more stable behavioral control.

8. V2
This is used to denote those responses that occur with a frequency of 16-24% in the Hungarian adult population surveyed in the standardization, i.e. one in five or six protocols. Each country has its own frequency standard, and statistical control of the responses to this standard requires regular review.

Table	Response with localisation code	Freq or Relative Frequency (%)
I.	G – Butterfly	23
I.	DGkonf - Beetle	20
I.	1, D - Beetle	18
I.	Gzw - Mask, mask	15
III.	1, D - Obj (basket, parcel, etc.)	23
III.	2, D - Clothes (e.g. bows)	17
VII.	DGkonf - Human (like)	24
VIII.	4, 5, D - Rock, mountain	18

IX.	1, D - Animal	19
X.	3, D - Animal	19

Vulger 2 (V2) responses in the Rorschach test, with their prevalence (Gyak (%)*)* (*113*)

Interpretation: condensation of Vulger responses: conventional, conformist behaviour
V1 is the most conformist perception, following the others, seeing what the majority sees**.** The less conventional but still realistically conformist perception is V2. Let's have about the same number of V1 as V2+V3. That's what we work with. Separately for V2 we have no other interpretation.
The values V1, V2 and V3 are to be aggregated as several items according to the Mérei convention. This simply means the number of V1, V2 or V3 answers given by the respondent, this indicator is not interpreted as such, only **in the indicator V 1: (V2 + V3)** - the indicator V% is only a proportion of the total number of answers and the total number of Vulger answers.
The percentage of vulgar responses (V%) in normal populations is between 40-50%.

Supplementary Material 14 – List of differentially expressed (DE) genes

ID	mean_RA	mean_Control	sd_RA	sd_Control	FC	logFC	p value	—LOG10(p)	avgRank	Description	geneName	EntrezID
ENSG00000244734	3113.03179	1155.37854	3134.30291	2229.79337	4.94615893	2.3063086	2.25E-06	5.64774789	1	hemoglobin, beta [Source:HGNC Symbol;Acc:4827]	HBB	3043
ENSG00000172159	27.6964057	15.0683043	14.1607613	9.83920651	2.13401548	1.09357064	4.36772E-05	4.35974529	20	FERM domain containing 3 [Source:HGNC Symbol;Acc:24125]	FRMD3	257019
ENSG00000206172	200.528791	84.7344559	213.959683	131.290964	3.76725539	1.91351384	0.000118478	3.92636126	2	hemoglobin, alpha 1 [Source:HGNC Symbol;Acc:4823]	HBA1	3040
ENSG00000072195	0.84592934	1.77279893	1.02687854	1.08506981	-2.7507419	-1.4598208	0.000154745	3.81038215	8	SPEG complex locus [Source:HGNC Symbol;Acc:16901]	SPEG	100996693
ENSG00000188536	725.398478	370.226899	709.489742	623.36819	3.46413643	1.79249575	0.000156921	3.80431767	6	hemoglobin, alpha 2 [Source:HGNC Symbol;Acc:4824]	HBA2	3040
ENSG00000133169	1.70674775	0.35488561	1.63098609	0.55120452	3.48580041	1.80148996	0.000190111	3.72099339	5	brain expressed, X-linked 1 [Source:HGNC Symbol;Acc:1036]	BEX1	55859
ENSG00000198829	5.21510252	1.93308799	5.01707692	1.67530504	2.8737082	1.52291358	0.000235605	3.62781513	11	succinate receptor 1 [Source:HGNC Symbol;Acc:4542]	SUCNR1	56670
ENSG00000169877	3.29442262	0.38280226	4.92505856	0.63392344	4.6843509	2.22784915	0.000235838	3.62738625	4	alpha hemoglobin stabilizing protein [Source:HGNC Symbol;Acc:18075]	AHSP	51327
ENSG00000047597	5.49765327	2.17170901	5.305473	2.47853265	2.97485431	1.57281902	0.000238843	3.62188688	10	X-linked Kx blood group (McLeod syndrome) [Source:HGNC Symbol;Acc:12811]	XK	7504
ENSG00000227184	1.3560413	3.26868176	1.78329894	2.37719746	-2.724522	-1.4460032	0.000376205	3.42457566	13	epiplakin 1 [Source:HGNC Symbol;Acc:15577]	EPPK1	83481
ENSG00000158578	8.45383713	1.39422151	10.7508797	2.76471307	6.17007992	2.62528918	0.000536203	3.270671	3	aminolevulinate, delta-, synthase 2 [Source:HGNC Symbol;Acc:397]	ALAS2	212
ENSG00000148498	6.00170575	2.81407373	4.02885418	2.22724482	2.67014094	1.41691589	0.000550084	3.25957064	15	par-3 family cell polarity regulator [Source:HGNC Symbol;Acc:16051]	PARD3	56288
ENSG00000134668	3.49283645	1.16141706	3.05440923	1.17629249	3.17332999	1.66599755	0.0007052	3.15168789	12	SPOC domain containing 1 [Source:HGNC Symbol;Acc:26338]	SPOCD1	90853
ENSG00000206177	6.5250651	1.23144829	9.0767652	1.80420905	4.90589658	2.29451682	0.000722048	3.14143366	7	hemoglobin, mu [Source:HGNC Symbol;Acc:4826]	HBM	3042
ENSG00000140022	7.54117584	4.23916814	3.79637432	2.28796943	1.87583806	0.90753529	0.000941327	3.02625939	49	stonin 2 [Source:HGNC Symbol;Acc:30652]	STON2	85439
ENSG00000072422	12.542174	6.64826572	8.25325374	5.6561545	2.50355841	1.32398012	0.000943914	3.02506755	18	Rho-related BTB domain containing 1 [Source:HGNC Symbol;Acc:18738]	RHOBTB1	9886

ENSG00000144290	5.12471531	12.6637233	6.65849265	16.339091	-2.9306426	-1.551217	0.001055405	2.97658103	16	solute carrier family 4, sodium bicarbonate transporter, member 10 [Source:HGNC Symbol;Acc:13811]	SLC4A10	57282
ENSG00000147650	6.02068516	3.18953792	3.39136417	2.220231	2.21525993	1.14747599	0.001157733	2.93639165	21	low density lipoprotein receptor-related protein 12 [Source:HGNC Symbol;Acc:31708]	LRP12	29967
ENSG00000136231	13.4577281	6.84541599	8.60037442	4.56307794	2.07451934	1.05277711	0.001332835	2.87522377	28	insulin-like growth factor 2 mRNA binding protein 3 [Source:HGNC Symbol;Acc:28868]	IGF2BP3	10643
ENSG00000143416	2.19441546	0.61392877	2.57841094	1.04208788	3.30552809	1.72488078	0.001441532	2.84117563	14	selenium binding protein 1 [Source:HGNC Symbol;Acc:10719]	SELENBP1	8991
ENSG00000223609	7.67352761	1.07968881	11.5423204	1.77616381	5.03994561	2.33340817	0.001714398	2.76588823	9	hemoglobin, delta [Source:HGNC Symbol;Acc:4829]	HBD	3045
ENSG00000142173	6.76108091	13.8371288	4.93226688	8.73062204	-1.8773283	-0.908681	0.00172191	2.76398951	51	collagen, type VI, alpha 2 [Source:HGNC Symbol;Acc:2212]	COL6A2	1292
ENSG00000091106	78.6265559	52.0815209	33.7977166	23.0992511	1.62683683	0.70206956	0.001865524	2.72919905	142	NLR family, CARD domain containing 4 [Source:HGNC Symbol;Acc:16412]	NLRC4	58484
ENSG00000132970	3.78442297	1.80640219	3.61353032	2.01607679	2.64842505	1.40513468	0.002177091	2.66212346	17	WAS protein family, member 3 [Source:HGNC Symbol;Acc:12734]	WASF3	10810
ENSG00000153162	3.46438972	1.47847307	2.78040228	1.27450465	2.55137348	1.3512741	0.00232701	2.63320181	19	bone morphogenetic protein 6 [Source:HGNC Symbol;Acc:1073]	BMP6	654
ENSG00000109674	4.48030835	2.01492106	2.98626945	1.22693646	2.24167287	1.16457576	0.00243442	2.61360453	25	nei endonuclease VIII-like 3 (E. coli) [Source:HGNC Symbol;Acc:24573]	NEIL3	55247
ENSG00000204165	3.32720556	5.88153269	2.16747916	2.93570524	-1.8894493	-0.9179658	0.002697631	2.5690174	50	chromosome X open reading frame 65 [Source:HGNC Symbol;Acc:33713]	CXorf65	158830
ENSG00000088053	9.30664507	4.95428782	7.0952495	4.32648309	2.26618099	1.18026309	0.002919568	2.53468146	24	glycoprotein VI (platelet) [Source:HGNC Symbol;Acc:14388]	GP6	51206
ENSG00000155755	1.82198222	3.16327296	1.41273653	1.8717985	-2.0106236	-1.0076431	0.003104258	2.5080422	34	transmembrane protein 237 [Source:HGNC Symbol;Acc:14432]	TMEM237	65062
ENSG00000062524	1.47769246	3.66819787	1.57525055	3.35933968	-2.2602128	-1.1764586	0.003185511	2.49682086	27	leukocyte receptor tyrosine kinase [Source:HGNC Symbol;Acc:6721]	LTK	4058
ENSG00000144645	13.0867554	19.7749679	10.6864099	8.15709664	-2.2912811	-1.1961545	0.003312539	2.47983899	22	oxysterol binding protein-like 10 [Source:HGNC Symbol;Acc:16395]	OSBPL10	114884
ENSG00000113368	79.9037564	51.7079558	36.8973184	20.3330311	1.60468732	0.68229221	0.003745571	2.42648201	155	lamin B1 [Source:HGNC Symbol;Acc:6637]	LMNB1	4001

ENSG00000119862	21.8185771	11.9427089	13.5060562	5.58457664	1.84618196	0.88454475	0.003757952	2.42504873	56	lectin, galactoside-binding-like [Source:HGNC Symbol;Acc:25012]	LGALSL	29094
ENSG00000145685	32.2219176	19.8079182	17.5066573	8.26688737	1.64753683	0.72031072	0.003922913	2.40639128	133	lipoma HMGIC fusion partner-like 2 [Source:HGNC Symbol;Acc:6588]	LHFPL2	10184
ENSG00000114405	2.25362687	1.29647683	1.72704475	1.56660677	2.44311443	1.28872144	0.003944663	2.40399009	23	chromosome 3 open reading frame 14 [Source:HGNC Symbol;Acc:25024]	C3orf14	57415
ENSG00000111817	67.0005141	47.8762958	19.4122555	11.9441669	1.46431299	0.55022396	0.004569113	2.34016814	285	dermatan sulfate epimerase [Source:HGNC Symbol;Acc:21144]	DSE	29940
ENSG00000255103	4.48360448	7.52308772	2.76182285	3.3770128	-1.5940323	-0.6726808	0.004672623	2.33043929	169	KIAA0754 [Source:HGNC Symbol;Acc:29111]	KIAA0754	643314
ENSG00000150681	177.124698	121.791	75.1291885	69.7256025	1.60822336	0.68546779	0.004777201	2.3208265	157	regulator of G-protein signaling 18 [Source:HGNC Symbol;Acc:14261]	RGS18	64407
ENSG00000171241	3.94329055	2.12866434	2.25859385	1.80038289	2.09185218	1.06478091	0.004853805	2.31391763	32	SHC SH2-domain binding protein 1 [Source:HGNC Symbol;Acc:29547]	SHCBP1	79801
ENSG00000123405	86.8080975	56.2310342	41.3155318	28.0611892	1.63862012	0.71248143	0.004999956	2.30103379	139	nuclear factor, erythroid 2 [Source:HGNC Symbol;Acc:7780]	NFE2	4778
ENSG00000177324	13.4411641	6.870657	10.6404179	5.37797698	2.27028045	1.18287053	0.005484678	2.26084884	29	BEN domain containing 2 [Source:HGNC Symbol;Acc:28509]	BEND2	139105
ENSG00000136869	103.326637	68.4058767	45.1106063	22.1048365	1.5445877	0.62722179	0.005562312	2.25474464	207	toll-like receptor 4 [Source:HGNC Symbol;Acc:11850]	TLR4	7099
ENSG00000065534	28.5674708	17.702701	16.9221661	13.4831022	1.84184734	0.88115349	0.005664057	2.24687236	63	myosin light chain kinase [Source:HGNC Symbol;Acc:7590]	MYLK	4638
ENSG00000102362	10.2153395	6.03921723	6.97525721	4.45481336	2.02426697	1.01739958	0.005786879	2.23755562	40	synaptotagmin-like 4 [Source:HGNC Symbol;Acc:15588]	SYTL4	94121
ENSG00000100336	1.73380036	0.45537549	1.98760385	0.58452344	2.56140766	1.35693688	0.005997922	2.22199922	26	apolipoprotein L, 4 [Source:HGNC Symbol;Acc:14867]	APOL4	80832
ENSG00000110104	4.55496269	7.54762303	3.05387595	3.41557414	-1.7017277	-0.7670002	0.00627337	2.20249909	113	coiled-coil domain containing 86 [Source:HGNC Symbol;Acc:28359]	CCDC86	79080
ENSG00000174080	3.93665687	6.63958476	2.09919283	2.8632222	-1.6330972	-0.7076106	0.006277535	2.20221084	148	cathepsin F [Source:HGNC Symbol;Acc:2531]	CTSF	8722
ENSG00000187808	9.14115213	6.64283071	3.570676	4.74125179	1.67477429	0.74396668	0.006411653	2.19302997	124	sosondowah ankyrin repeat domain family member D [Source:HGNC Symbol;Acc:32960]	SOWAHD	347454
ENSG00000003436	5.87620917	3.03616628	4.95545679	3.55208305	2.35765661	1.2373536	0.00723764	2.14040303	30	tissue factor pathway inhibitor (lipoprotein-associated coagulation inhibitor)	TFPI	7035

										[Source:HGNC Symbol;Acc:11760]		
ENSG00000049246	4.53108676	7.42787982	2.69258067	3.26961252	-1.6310914	-0.7058377	0.007454447	2.12758458	151	period circadian clock 3 [Source:HGNC Symbol;Acc:8847]	PER3	8863
ENSG00000034053	24.5564809	36.516268	11.844073	12.0799498	-1.5456911	-0.6282521	0.007468662	2.1267572	211	amyloid beta (A4) precursor protein-binding, family A, member 2 [Source:HGNC Symbol;Acc:579]	APBA2	321
ENSG00000141736	4.79392602	8.53961103	3.245201	5.12298671	-1.7656585	-0.8202063	0.007617837	2.11816832	88	v-erb-b2 avian erythroblastic leukemia viral oncogene homolog 2 [Source:HGNC Symbol;Acc:3430]	ERBB2	2064
ENSG00000196466	5.37841193	3.5979144	3.41130267	2.9440097	2.01201358	1.00864004	0.007691913	2.11396565	48	zinc finger protein 799 [Source:HGNC Symbol;Acc:28071]	ZNF799	90576
ENSG00000145335	21.8966721	9.44969852	18.8822267	6.0576883	2.08725228	1.06160498	0.007932622	2.10058324	39	synuclein, alpha (non A4 component of amyloid precursor) [Source:HGNC Symbol;Acc:11138]	SNCA	6622
ENSG00000198934	4.95449136	7.71111476	3.08435207	3.71575058	-1.7773187	-0.8297024	0.00802669	2.09546349	86	melanoma antigen family E, 1 [Source:HGNC Symbol;Acc:24934]	MAGEE1	57692
ENSG00000167633	2.27170079	4.13324238	2.8038383	3.02006715	-2.1901808	-1.13105	0.008068233	2.09322155	37	killer cell immunoglobulin-like receptor, three domains, long cytoplasmic tail, 1 [Source:HGNC Symbol;Acc:6338]	KIR3DL1	3812
ENSG00000249992	19.0075731	9.70998216	13.682446	5.98482611	1.87647557	0.90802551	0.008306183	2.08059851	60	transmembrane protein 158 (gene/pseudogene) [Source:HGNC Symbol;Acc:30293]	TMEM158	25907
ENSG00000107317	8.31341339	16.4776172	7.24971888	11.4549206	-1.8757334	-0.9074548	0.008525805	2.06926463	62	prostaglandin D2 synthase 21kDa (brain) [Source:HGNC Symbol;Acc:9592]	PTGDS	5730
ENSG00000213782	2.09934836	4.03196829	1.85896841	3.52441715	-2.0410961	-1.0293441	0.008526141	2.06924749	45	DEAD (Asp-Glu-Ala-Asp) box polypeptide 47 [Source:HGNC Symbol;Acc:18682]	DDX47	51202
ENSG00000130830	67.3870492	48.0623207	22.0626338	13.2745165	1.46690762	0.55277802	0.008668571	2.0620525	289	membrane protein, palmitoylated 1, 55kDa [Source:HGNC Symbol;Acc:7219]	MPP1	4354
ENSG00000204475	4.62137566	8.535486	3.25727996	5.39701742	-1.7045384	-0.7693811	0.008901005	2.05056093	116	natural cytotoxicity triggering receptor 3 [Source:HGNC Symbol;Acc:19077]	NCR3	259197
ENSG00000267673	0.90498094	1.73376418	1.08117289	1.42638188	-2.1660731	-1.1150819	0.008989436	2.04626754	41	ferredoxin 1-like [Source:HGNC Symbol;Acc:30546]	FDX1L	112812
ENSG00000180190	2.04677454	1.25558605	1.82823075	1.36125879	2.1524479	1.10597831	0.009034905	2.04407643	44	testis development related protein [Source:HGNC Symbol;Acc:26951]	TDRP	157695

ENSG00000184792	3.38993579	1.06349579	3.75204388	1.15122179	2.6010701	1.37910528	0.009658127	2.01510711	31	oxysterol binding protein 2 [Source:HGNC Symbol;Acc:8504]	OSBP2	23762
ENSG00000184113	9.53744034	4.83878876	8.05890437	4.37219209	2.16804428	1.11639422	0.009845348	2.00676894	43	claudin 5 [Source:HGNC Symbol;Acc:2047]	CLDN5	7122
ENSG00000103740	1.79544184	0.68805835	1.69642377	0.79268068	2.36542295	1.24209817	0.010388676	1.9834398	33	acyl-CoA synthetase bubblegum family member 1 [Source:HGNC Symbol;Acc:29567]	ACSBG1	23205
ENSG00000181631	170.286503	116.808552	71.8028608	53.8554781	1.54664465	0.62914177	0.010542794	1.97704427	218	purinergic receptor P2Y, G-protein coupled, 13 [Source:HGNC Symbol;Acc:4537]	P2RY13	53829
ENSG00000080493	4.06929689	7.18019559	2.48186855	4.06565712	-1.7993851	-0.847504	0.010610482	1.97426488	82	solute carrier family 4 (sodium bicarbonate cotransporter), member 4 [Source:HGNC Symbol;Acc:11030]	SLC4A4	8671
ENSG00000162367	37.9275677	22.1503069	25.7673792	15.6393445	1.86736647	0.90100508	0.011091481	1.95501047	66	T-cell acute lymphocytic leukemia 1 [Source:HGNC Symbol;Acc:11556]	TAL1	6886
ENSG00000055732	2.49339573	1.07271642	2.10745067	0.93371837	2.22134332	1.15143239	0.011126035	1.95365957	42	mucolipin 3 [Source:HGNC Symbol;Acc:13358]	MCOLN3	55283
ENSG00000205639	2.82323527	1.32652024	2.69970325	1.2783593	2.27165053	1.18374091	0.011227028	1.94973519	38	major facilitator superfamily domain containing 2B [Source:HGNC Symbol;Acc:37207]	MFSD2B	388931
ENSG00000101162	348.777478	206.5292	230.012529	148.52903	1.77229	0.82561469	0.011303928	1.94677061	100	tubulin, beta 1 class VI [Source:HGNC Symbol;Acc:16257]	TUBB1	81027
ENSG00000175471	98.3543208	71.8188992	28.7899545	21.408754	1.44912265	0.53517971	0.01140597	1.94286776	317	multiple C2 domains, transmembrane 1 [Source:HGNC Symbol;Acc:26183]	MCTP1	79772
ENSG00000112299	15.3544865	10.6677606	8.89489694	9.09304534	1.80553325	0.85242499	0.011443685	1.9414341	83	vanin 1 [Source:HGNC Symbol;Acc:12705]	VNN1	8876
ENSG00000269636	7.13421318	3.21788126	7.27489887	3.20646761	2.47369548	1.30666791	0.011507994	1.93900039	36		AC010441.1	
ENSG00000088726	12.7541262	6.91799916	9.97532679	4.79798429	1.86014659	0.89541632	0.01152625	1.93831195	71	transmembrane protein 40 [Source:HGNC Symbol;Acc:25620]	TMEM40	55287
ENSG00000138449	61.3562862	44.0599345	21.7636362	13.0653849	1.45764207	0.5436365	0.012030854	1.91970356	314	solute carrier family 40 (iron-regulated transporter), member 1 [Source:HGNC Symbol;Acc:10909]	SLC40A1	30061
ENSG00000115289	4.66354987	3.1066065	2.38458603	1.52081505	1.62962393	0.70453907	0.012224302	1.91277593	170	polycomb group ring finger 1 [Source:HGNC Symbol;Acc:17615]	PCGF1	84759
ENSG00000091129	0.92231087	2.28095957	1.1779693	2.41489514	-2.2199929	-1.1505551	0.012465573	1.90428777	46	neuronal cell adhesion molecule [Source:HGNC Symbol;Acc:7994]	NRCAM	4897

ENSG00000137098	2.10047441	3.55431904	1.94892974	2.430343	-2.0036243	-1.002612	0.012469009	1.90416807	54	sperm associated antigen 8 [Source:HGNC Symbol;Acc:14105]	SPAG8	26206
ENSG00000105289	1.4655656	2.51117387	1.57326287	1.67107638	-2.0163345	-1.011735	0.012536424	1.90182632	52	tight junction protein 3 [Source:HGNC Symbol;Acc:11829]	TJP3	27134
ENSG00000102010	3.52485584	1.73630081	3.48124582	2.29650182	2.62341449	1.39144577	0.012578267	1.90037918	35	BMX non-receptor tyrosine kinase [Source:HGNC Symbol;Acc:1079]	BMX	660
ENSG00000070371	2.7712781	1.68352126	1.46535048	1.33144927	2.01301806	1.00936011	0.01286242	1.89067733	53	clathrin, heavy chain-like 1 [Source:HGNC Symbol;Acc:2093]	CLTCL1	8218
ENSG00000169398	14.8912295	9.62799727	7.49289857	4.19751931	1.58821812	0.66740906	0.012984754	1.88656628	191	protein tyrosine kinase 2 [Source:HGNC Symbol;Acc:9611]	PTK2	5747
ENSG00000126861	3.51453347	1.93137106	2.63668556	2.53111423	2.26672918	1.18061203	0.012997694	1.8861337	47	oligodendrocyte myelin glycoprotein [Source:HGNC Symbol;Acc:8135]	OMG	101927057
ENSG00000133069	5.24665369	2.89221658	4.25800069	1.75931677	1.88027658	0.91094489	0.013434356	1.87178314	68	transmembrane and coiled-coil domain family 2 [Source:HGNC Symbol;Acc:24239]	TMCC2	9911
ENSG00000167747	2.30563802	3.24346137	2.66713527	1.81735058	-1.9431264	-0.9583797	0.013520801	1.86899757	64	chromosome 19 open reading frame 48 [Source:HGNC Symbol;Acc:29667]	C19orf48	692204
ENSG00000129595	2.81675112	5.12339874	2.21796496	3.73956482	-1.9421187	-0.9576314	0.013678917	1.86394829	65	erythrocyte membrane protein band 4.1 like 4A [Source:HGNC Symbol;Acc:13278]	EPB41L4A	64097
ENSG00000137124	2.30350179	3.99484397	1.55594756	2.29573476	-1.7658409	-0.8203553	0.013681846	1.86385529	111	aldehyde dehydrogenase 1 family, member B1 [Source:HGNC Symbol;Acc:407]	ALDH1B1	219
ENSG00000166428	2.41293318	4.40417469	2.1968438	2.91887033	-1.8613329	-0.8963361	0.01462686	1.83484889	74	phospholipase D family, member 4 [Source:HGNC Symbol;Acc:23792]	PLD4	122618
ENSG00000005961	46.1219609	26.7943422	37.2700314	17.2981723	1.83212852	0.87352071	0.014884187	1.82727488	81	integrin, alpha 2b (platelet glycoprotein IIb of IIb/IIIa complex, antigen CD41) [Source:HGNC Symbol;Acc:6138]	ITGA2B	3674
ENSG00000112290	3.09990552	1.76431087	2.49525756	1.45704018	2.01058194	1.00761314	0.015200354	1.81814631	57	WAS protein family, member 1 [Source:HGNC Symbol;Acc:12732]	WASF1	8936
ENSG00000127920	124.65307	71.3485208	94.077821	54.0391419	1.80299087	0.85039209	0.015289714	1.81560063	95	guanine nucleotide binding protein (G protein), gamma 11 [Source:HGNC Symbol;Acc:4403]	GNG11	2791
ENSG00000166313	8.98916117	14.0663714	5.06718211	7.53593437	-1.5196806	-0.6037681	0.015975897	1.79653474	257	amyloid beta (A4) precursor protein-binding, family B, member 1 (Fe65) [Source:HGNC Symbol;Acc:581]	APBB1	322

ENSG00000204301	1.39103304	2.30680649	1.36872833	1.36271882	-1.8783303	-0.9094508	0.016012256	1.79554748	73	notch 4 [Source:HGNC Symbol;Acc:7884]	NOTCH4	4855
ENSG00000137807	2.16494209	1.47873518	1.27621955	1.25016221	1.89028683	0.91860516	0.016091322	1.79340826	72	kinesin family member 23 [Source:HGNC Symbol;Acc:6392]	KIF23	9493
ENSG00000185245	15.2365901	9.39822736	10.5834256	6.86176146	1.82009458	0.86401342	0.016746368	1.77607937	91	glycoprotein Ib (platelet), alpha polypeptide [Source:HGNC Symbol;Acc:4439]	GP1BA	2811
ENSG00000243452	1.59284382	2.72615528	1.84422755	1.75231532	-1.9080625	-0.9321084	0.017215907	1.76407009	70	neuroblastoma breakpoint family, member 15 [Source:HGNC Symbol;Acc:28791]	NBPF15	284565
ENSG00000204420	68.534284	39.0413615	53.9487416	27.0505283	1.80501446	0.85201039	0.017663755	1.75291698	101	chromosome 6 open reading frame 25 [Source:HGNC Symbol;Acc:13937]	C6orf25	80739
ENSG00000135378	44.7052617	27.8635055	25.198965	15.1915541	1.61365621	0.69033324	0.017704828	1.7519083	185	proline rich Gla (G-carboxyglutamic acid) 4 (transmembrane) [Source:HGNC Symbol;Acc:30799]	PRRG4	79056
ENSG00000173210	10.4231144	5.83777273	8.81008413	4.43850597	1.87510471	0.90697116	0.017779353	1.75008404	75	actin binding LIM protein family, member 3 [Source:HGNC Symbol;Acc:29132]	ABLIM3	22885
ENSG00000120896	3.90736698	5.93872342	2.53834314	2.15100887	-1.622289	-0.6980309	0.018081253	1.74277147	180	sorbin and SH3 domain containing 3 [Source:HGNC Symbol;Acc:30907]	SORBS3	10174
ENSG00000176945	3.9972019	7.09247189	3.28767838	4.80353315	-2.047314	-1.0337324	0.018619305	1.73003654	55	mucin 20, cell surface associated [Source:HGNC Symbol;Acc:23282]	MUC20	200958
ENSG00000183762	12.4210595	6.25956642	12.0967908	6.73891384	2.03209716	1.02296938	0.019198544	1.7167317	59	kringle containing transmembrane protein 1 [Source:HGNC Symbol;Acc:17550]	KREMEN1	83999
ENSG00000162591	2.91857402	5.24050842	2.26723653	3.77015034	-1.7424869	-0.8011478	0.019358605	1.71312594	123	multiple EGF-like-domains 6 [Source:HGNC Symbol;Acc:3232]	MEGF6	1953
ENSG00000134827	9.28189995	4.86521236	8.35599563	3.7353379	1.85609304	0.89226903	0.019861512	1.70198769	84	transcobalamin I (vitamin B12 binding protein, R binder family) [Source:HGNC Symbol;Acc:11652]	TCN1	6947
ENSG00000126351	30.4777874	43.7095674	7.91067258	10.748305	-1.3681665	-0.4522438	0.019870706	1.7017867	501	thyroid hormone receptor, alpha [Source:HGNC Symbol;Acc:11796]	THRA	7067
ENSG00000197903	183.305657	108.264069	112.09653	62.7785852	1.74684142	0.80474864	0.020043207	1.69803278	126	histone cluster 1, H2bk [Source:HGNC Symbol;Acc:13954]	HIST1H2BK	85236
ENSG00000184702	52.1610449	29.9867935	39.5902926	19.0353034	1.79161026	0.84125683	0.020232202	1.69395685	112	septin 5 [Source:HGNC Symbol;Acc:9164]	SEPT5	5413
ENSG00000125459	4.07092475	6.68767784	2.3296545	3.33375433	-1.664912	-0.735446	0.021234028	1.67296761	163	misato 1, mitochondrial distribution and morphology	MSTO1	55154

										regulator [Source:HGNC Symbol;Acc:29678]		
ENSG00000115318	4.38626195	2.63980129	2.81637566	2.07528107	1.88145819	0.91185123	0.021265087	1.67233283	76	lysyl oxidase-like 3 [Source:HGNC Symbol;Acc:13869]	LOXL3	84695
ENSG00000061918	20.3137181	11.6646619	14.6119295	7.59280475	1.71265563	0.77623509	0.021289531	1.67183391	140	guanylate cyclase 1, soluble, beta 3 [Source:HGNC Symbol;Acc:4687]	GUCY1B3	2983
ENSG00000038945	12.7644894	6.59514692	9.37202769	3.50485783	1.81375204	0.85897724	0.021384814	1.66989452	103	macrophage scavenger receptor 1 [Source:HGNC Symbol;Acc:7376]	MSR1	4481
ENSG00000114541	53.1636492	39.6570703	18.3979769	12.0360893	1.39664566	0.48196604	0.021664665	1.66424801	427	FERM domain containing 4B [Source:HGNC Symbol;Acc:24886]	FRMD4B	23150
ENSG00000214595	4.34853398	5.52231297	4.31797993	2.45002887	-1.857736	-0.8935455	0.021727992	1.66298042	90	echinoderm microtubule associated protein like 6 [Source:HGNC Symbol;Acc:35412]	EML6	400954
ENSG00000064763	13.7487403	9.37944599	6.6534825	4.566373	1.56183049	0.64323789	0.021939767	1.65876798	230	fatty acyl CoA reductase 2 [Source:HGNC Symbol;Acc:25531]	FAR2	55711
ENSG00000198478	31.4930351	19.4708849	22.8922357	15.0971906	1.73382921	0.7939618	0.02194025	1.65875843	135	SH3 domain binding glutamic acid-rich protein like 2 [Source:HGNC Symbol;Acc:15567]	SH3BGRL2	83699
ENSG00000082196	1.42617631	2.2556547	1.18354675	1.42744948	-1.8733855	-0.9056478	0.021964951	1.65826976	85	C1q and tumor necrosis factor related protein 3 [Source:HGNC Symbol;Acc:14326]	C1QTNF3	114899
ENSG00000085733	24.6639946	15.3132271	18.2441503	14.7622085	1.82596913	0.86866238	0.022126598	1.65508535	102	cortactin [Source:HGNC Symbol;Acc:3338]	CTTN	2017
ENSG00000143878	43.3131398	30.769088	25.4154114	24.0912416	1.67527583	0.74439865	0.022144677	1.65473064	161	ras homolog family member B [Source:HGNC Symbol;Acc:668]	RHOB	388
ENSG00000109684	2.0484579	3.11702537	1.50278053	1.77189658	-1.7243289	-0.786035	0.022243997	1.65278716	143	cytokine-dependent hematopoietic cell linker [Source:HGNC Symbol;Acc:17438]	CLNK	116449
ENSG00000259207	20.162006	12.1093108	16.6351514	8.89944599	1.80210375	0.84968207	0.022388986	1.64996557	115	integrin, beta 3 (platelet glycoprotein IIIa, antigen CD61) [Source:HGNC Symbol;Acc:6156]	ITGB3	3690
ENSG00000172543	32.7523923	53.6822672	17.1920231	26.4235791	-1.5264823	-0.6102108	0.022494285	1.64792781	265	cathepsin W [Source:HGNC Symbol;Acc:2546]	CTSW	1521
ENSG00000113532	120.921058	92.0453894	35.6861458	24.6559863	1.38423364	0.46908747	0.022654532	1.6448449	460	ST8 alpha-N-acetyl-neuraminide alpha-2,8-sialyltransferase 4 [Source:HGNC Symbol;Acc:10871]	ST8SIA4	7903
ENSG00000099256	2.00847286	0.99433086	1.91142435	1.21145427	2.2149391	1.14726703	0.023018905	1.63791534	61	phosphoribosyl transferase domain containing 1 [Source:HGNC Symbol;Acc:23333]	PRTFDC1	56952

ENSG00000137757	22.2165142	10.6902111	19.4789426	7.22443386	1.81045971	0.85635607	0.02302305	1.63783714	110	caspase 5, apoptosis-related cysteine peptidase [Source:HGNC Symbol;Acc:1506]	CASP5	838
ENSG00000051128	34.796217	25.0527018	15.7811836	9.29282427	1.46835774	0.5542035	0.023104717	1.63629935	324	homer homolog 3 (Drosophila) [Source:HGNC Symbol;Acc:17514]	HOMER3	9454
ENSG00000198948	24.3278202	14.680201	17.6702591	10.6539595	1.75396775	0.81062222	0.023152051	1.63541052	136	microfibrillar-associated protein 3-like [Source:HGNC Symbol;Acc:29083]	MFAP3L	101928198
ENSG00000005249	198.083923	123.714277	146.954991	98.4831642	1.72653129	0.78787648	0.023176369	1.63495461	145	protein kinase, cAMP-dependent, regulatory, type II, beta [Source:HGNC Symbol;Acc:9392]	PRKAR2B	5577
ENSG00000146243	3.75690281	5.6872077	2.70819657	2.95343413	-1.6696223	-0.7395218	0.023228737	1.6339744	172	interleukin-1 receptor-associated kinase 1 binding protein 1 [Source:HGNC Symbol;Acc:17368]	IRAK1BP1	134728
ENSG00000011105	7.20930812	4.42094112	5.04075145	3.07021745	1.72537816	0.7869126	0.023327526	1.63213132	146	tetraspanin 9 [Source:HGNC Symbol;Acc:21640]	TSPAN9	10867
ENSG00000187116	101.805731	70.2966498	43.1295122	26.5717511	1.48805458	0.57342744	0.023400423	1.6307763	295	leukocyte immunoglobulin-like receptor, subfamily A (with TM domain), member 5 [Source:HGNC Symbol;Acc:16309]	LILRA5	353514
ENSG00000169704	12.8002121	7.88153354	11.0478094	6.48622412	1.83783614	0.87800815	0.023420115	1.63041098	108	glycoprotein IX (platelet) [Source:HGNC Symbol;Acc:4444]	GP9	2815
ENSG00000170677	10.5115374	7.31150662	4.47074543	4.46257931	1.53691479	0.62003718	0.023465112	1.62957738	261	suppressor of cytokine signaling 6 [Source:HGNC Symbol;Acc:16833]	SOCS6	9306
ENSG00000124145	2.52989012	1.79684796	1.53432259	1.41016507	1.8084642	0.85476504	0.023575466	1.62753971	117	syndecan 4 [Source:HGNC Symbol;Acc:10661]	SDC4	6385
ENSG00000170545	3.76658736	5.87356621	3.07702817	3.34710782	-1.6065024	-0.6839231	0.02414266	1.61721489	203	small cell adhesion glycoprotein [Source:HGNC Symbol;Acc:26918]	SMAGP	57228
ENSG00000187951	4.22195295	3.04741886	2.05482831	1.80930687	1.68787013	0.7552039	0.024440952	1.61188187	168	Rho GTPase activating protein 11B [Source:HGNC Symbol;Acc:15782]	ARHGAP11B	100288637
ENSG00000197959	22.0879462	14.4567579	14.0995119	12.6141327	1.72877273	0.78974822	0.024824458	1.60512023	147	dynamin 3 [Source:HGNC Symbol;Acc:29125]	DNM3	26052
ENSG00000123095	2.55320124	3.30409383	2.87403413	2.56240833	-1.959604	-0.9705621	0.024876101	1.60421769	78	basic helix-loop-helix family, member e41 [Source:HGNC Symbol;Acc:16617]	BHLHE41	79365
ENSG00000019991	11.7427257	7.72231835	6.31113644	2.7994412	1.51092129	0.59542851	0.024973316	1.60252378	279	hepatocyte growth factor (hepapoietin A; scatter factor) [Source:HGNC Symbol;Acc:4893]	HGF	3082
ENSG00000151458	22.5710527	15.5370154	10.509025	6.24703838	1.48854242	0.57390034	0.025014439	1.60180923	298	ankyrin repeat domain 50 [Source:HGNC Symbol;Acc:29223]	ANKRD50	57182

ENSG00000180914	3.12480512	1.64032312	2.70478959	1.43282697	1.979046	0.98480515	0.025319522	1.59654449	77	oxytocin receptor [Source:HGNC Symbol;Acc:8529]	OXTR	5021
ENSG00000197774	5.1142838	7.79487868	3.41351514	3.17346794	-1.670337	-0.7401392	0.025958793	1.58571551	175	essential meiotic structure-specific endonuclease subunit 2 [Source:HGNC Symbol;Acc:27289]	EME2	197342
ENSG00000128512	12.7026794	6.93932878	9.0419921	5.53941973	1.79369826	0.84293722	0.026849552	1.57106296	129	dedicator of cytokinesis 4 [Source:HGNC Symbol;Acc:19192]	DOCK4	9732
ENSG00000137198	6.29983093	2.72927674	7.5684277	2.51460744	2.12281398	1.08597796	0.026969566	1.56912604	69	guanosine monophosphate reductase [Source:HGNC Symbol;Acc:4376]	GMPR	2766
ENSG00000047648	18.2053386	10.2657465	12.2437012	5.62173098	1.75451474	0.81107207	0.027018145	1.56834447	144	Rho GTPase activating protein 6 [Source:HGNC Symbol;Acc:676]	ARHGAP6	395
ENSG00000005020	266.364356	207.003457	67.0153534	50.8801664	1.3689145	0.45303234	0.027392648	1.56236598	513	src kinase associated phosphoprotein 2 [Source:HGNC Symbol;Acc:15687]	SKAP2	8935
ENSG00000157240	4.7656308	2.98505998	3.11808431	2.15685841	1.71520069	0.77837739	0.02801158	1.55266239	156	frizzled family receptor 1 [Source:HGNC Symbol;Acc:4038]	FZD1	8321
ENSG00000073536	1.91190807	3.38633143	1.48944538	2.05356711	-1.7262687	-0.7876571	0.028271045	1.54865813	154	notchless homolog 1 (Drosophila) [Source:HGNC Symbol;Acc:19889]	NLE1	54475
ENSG00000088826	11.6133026	7.17583734	7.6766763	4.24363536	1.71334785	0.77681808	0.028275928	1.54858313	159	spermine oxidase [Source:HGNC Symbol;Acc:15862]	SMOX	54498
ENSG00000008300	1.3153614	2.03611599	1.51291905	1.52084379	-1.8170791	-0.8616212	0.028359844	1.54729617	122	cadherin, EGF LAG seven-pass G-type receptor 3 [Source:HGNC Symbol;Acc:3230]	CELSR3	1951
ENSG00000168209	18.7353585	32.0306424	12.8861359	20.6406497	-1.5706759	-0.6513856	0.028583502	1.54388456	241	DNA-damage-inducible transcript 4 [Source:HGNC Symbol;Acc:24944]	DDIT4	54541
ENSG00000133067	2.50119972	4.69243921	1.99222341	3.93719324	-1.8073192	-0.8538513	0.028855151	1.53977664	128	leucine-rich repeat containing G protein-coupled receptor 6 [Source:HGNC Symbol;Acc:19719]	LGR6	59352
ENSG00000160856	27.1409775	41.9224853	11.6657429	17.6989475	-1.4702803	-0.5560912	0.028935711	1.53856585	332	Fc receptor-like 3 [Source:HGNC Symbol;Acc:18506]	FCRL3	115352
ENSG00000163430	7.85090187	4.72758536	9.85284107	8.03721761	2.27611108	1.18657097	0.029075056	1.53647945	67	follistatin-like 1 [Source:HGNC Symbol;Acc:3972]	FSTL1	11167
ENSG00000163126	1.12978214	1.60811377	1.60560645	1.27054025	-1.9474731	-0.9616034	0.029099325	1.53611709	92	ankyrin repeat domain 23 [Source:HGNC Symbol;Acc:24470]	ANKRD23	200539
ENSG00000162722	18.8219972	11.4903071	12.1348306	7.00622502	1.66624206	0.736598	0.030040283	1.52229598	182	tripartite motif containing 58 [Source:HGNC Symbol;Acc:24150]	TRIM58	25893

ENSG00000120885	151.269517	91.1534901	115.215332	63.1658447	1.67505839	0.74421138	0.030146142	1.52076826	178	clusterin [Source:HGNC Symbol;Acc:2095]	CLU	1191
ENSG00000159128	81.8960177	61.5111961	33.0010528	29.7920466	1.46649307	0.55237026	0.030436968	1.51659861	344	interferon gamma receptor 2 (interferon gamma transducer 1) [Source:HGNC Symbol;Acc:5440]	IFNGR2	3460
ENSG00000174125	98.9730912	70.744484	42.8825098	30.2838379	1.46766538	0.55352308	0.030907299	1.50993895	340	toll-like receptor 1 [Source:HGNC Symbol;Acc:11847]	TLR1	7096
ENSG00000090920	2.96575181	5.25649658	2.60641236	3.97445754	-1.8394808	-0.8792986	0.030915284	1.50982677	120	Fc fragment of IgG binding protein [Source:HGNC Symbol;Acc:13572]	FCGBP	8857
ENSG00000114374	7.92760418	38.6139991	23.708317	63.3033714	-3.8580099	-1.9478569	0.031165136	1.50633097	58	ubiquitin specific peptidase 9, Y-linked [Source:HGNC Symbol;Acc:12633]	USP9Y	8287
ENSG00000143995	7.1430144	3.94510835	5.86138497	2.98238189	1.91309243	0.93590658	0.031165789	1.50632188	104	Meis homeobox 1 [Source:HGNC Symbol;Acc:7000]	MEIS1	4211
ENSG00000118690	3.42139839	5.21987277	2.06823635	2.4564275	-1.5391333	-0.6221182	0.031294204	1.50453609	269	armadillo repeat containing 2 [Source:HGNC Symbol;Acc:23045]	ARMC2	84071
ENSG00000152213	38.5874664	28.2686115	14.3472818	11.6841605	1.44485789	0.53092761	0.031517931	1.5014423	367	ADP-ribosylation factor-like 11 [Source:HGNC Symbol;Acc:24046]	ARL11	115761
ENSG00000174428	3.88570352	5.91223102	2.12432138	2.48358362	-1.5773371	-0.657491	0.031582562	1.50055264	242	GTF2I repeat domain containing 2B [Source:HGNC Symbol;Acc:33125]	GTF2IRD2B	389524
ENSG00000163736	368.014749	220.317114	301.766363	162.380027	1.68814678	0.75544035	0.031728043	1.49855672	177	pro-platelet basic protein (chemokine (C-X-C motif) ligand 7) [Source:HGNC Symbol;Acc:9240]	PPBP	5473
ENSG00000198590	2.12409877	3.18322806	1.58473311	1.39450997	-1.679236	-0.747805	0.031812426	1.49740321	181	chromosome 3 open reading frame 35 [Source:HGNC Symbol;Acc:24082]	C3orf35	339883
ENSG00000149557	1.93027741	3.49213671	1.85268925	2.93286223	-1.8963824	-0.9232499	0.032052374	1.49413979	109	fasciculation and elongation protein zeta 1 (zygin I) [Source:HGNC Symbol;Acc:3659]	FEZ1	9638
ENSG00000136425	1.0251374	1.82072473	1.36378952	1.70073381	-1.9575597	-0.9690563	0.03206763	1.49393314	96	calcium and integrin binding family member 2 [Source:HGNC Symbol;Acc:24579]	CIB2	10518
ENSG00000136014	3.8598658	5.98095337	2.824637	3.53016596	-1.6251241	-0.7005499	0.032302729	1.49076078	214	ubiquitin specific peptidase 44 [Source:HGNC Symbol;Acc:20064]	USP44	84101
ENSG00000162490	3.02416415	4.35669108	4.33794885	3.49974896	-2.0211048	-1.0151441	0.032514705	1.48792019	89	dorsal inhibitory axon guidance protein [Source:HGNC Symbol;Acc:25054]	DRAXIN	374946
ENSG00000183621	31.1311782	23.4537921	9.28685505	7.02398134	1.4032861	0.48880918	0.032633104	1.48634162	448	zinc finger protein 438 [Source:HGNC Symbol;Acc:21029]	ZNF438	220929

ENSG00000054793	11.47593	8.76252461	5.66291125	6.33146835	1.59983658	0.67792455	0.032802786	1.48408927	227	ATPase, class II, type 9A [Source:HGNC Symbol;Acc:13540]	ATP9A	10079
ENSG00000171793	6.53143922	10.2623812	2.84368095	3.70120324	-1.4612982	-0.5472507	0.032808881	1.48400859	354	CTP synthase 1 [Source:HGNC Symbol;Acc:2519]	CTPS1	1503
ENSG00000166262	3.25977691	5.18822629	1.89523597	2.63501583	-1.5416239	-0.6244508	0.032830547	1.48372188	273	family with sequence similarity 227, member B [Source:HGNC Symbol;Acc:26543]	FAM227B	196951
ENSG00000100678	1.938896	0.58174805	2.4655947	0.71856766	2.15792765	1.10964649	0.032997179	1.48152319	80	solute carrier family 8 (sodium/calcium exchanger), member 3 [Source:HGNC Symbol;Acc:11070]	SLC8A3	6547
ENSG00000178623	1.45531369	3.02324093	1.38874612	3.2305915	-1.855101	-0.8914977	0.033068757	1.48058213	125	G protein-coupled receptor 35 [Source:HGNC Symbol;Acc:4492]	GPR35	2859
ENSG00000177432	2.19902256	3.59829381	1.41509916	2.25645779	-1.648364	-0.7210348	0.033239929	1.47833991	200	nucleosome assembly protein 1-like 5 [Source:HGNC Symbol;Acc:19968]	NAP1L5	266812
ENSG00000007264	14.2970099	22.6186035	8.00885724	11.1835492	-1.5005719	-0.5855125	0.033697181	1.47240643	310	megakaryocyte-associated tyrosine kinase [Source:HGNC Symbol;Acc:6906]	MATK	4145
ENSG00000174482	0.94616509	1.9537929	1.16319907	1.88185893	-1.955739	-0.9677139	0.033950404	1.46915505	106	leucine rich repeat and Ig domain containing 2 [Source:HGNC Symbol;Acc:21207]	LINGO2	158038
ENSG00000148346	18.5314242	11.7859283	19.0122925	12.6163618	1.76331111	0.81828704	0.034436914	1.46297577	158	lipocalin 2 [Source:HGNC Symbol;Acc:6526]	LCN2	3934
ENSG00000249437	8.63853825	5.69755881	5.29566537	3.62125501	1.70134869	0.76667885	0.034474198	1.46250583	179	NLR family, apoptosis inhibitory protein [Source:HGNC Symbol;Acc:7634]	NAIP	4671
ENSG00000213934	2.18944235	0.82902823	3.04725417	1.315702	2.25636159	1.17399828	0.034654889	1.46023548	79	hemoglobin, gamma A [Source:HGNC Symbol;Acc:4831]	HBG1	3047
ENSG00000167775	7.56919545	11.3367892	3.48120736	4.5583269	-1.4448953	-0.5309649	0.034710765	1.45953581	377	CD320 molecule [Source:HGNC Symbol;Acc:16692]	CD320	51293
ENSG00000170425	1.55948078	0.70650048	1.65938024	0.80625752	1.9841184	0.98849812	0.034714802	1.45948531	105	adenosine A2b receptor [Source:HGNC Symbol;Acc:264]	ADORA2B	136
ENSG00000168461	278.126346	213.635119	86.2217377	53.1618344	1.36216556	0.44590206	0.034851756	1.45777533	556	RAB31, member RAS oncogene family [Source:HGNC Symbol;Acc:9771]	RAB31	11031
ENSG00000177119	76.8599975	58.6674736	24.8669929	16.7662796	1.37742286	0.46197153	0.034865736	1.45760116	516	anoctamin 6 [Source:HGNC Symbol;Acc:25240]	ANO6	196527
ENSG00000163735	44.9678185	25.3401503	42.6083257	30.5237176	1.9142715	0.93679546	0.034886406	1.45734377	118	chemokine (C-X-C motif) ligand 5 [Source:HGNC Symbol;Acc:10642]	CXCL5	6374
ENSG00000179855	1.99553559	1.37590769	1.72151019	2.10601285	2.03551332	1.02539266	0.03495427	1.45649976	97	GIPC PDZ domain containing family, member 3 [Source:HGNC Symbol;Acc:18183]	GIPC3	126326

ENSG00000134461	3.09720617	4.22893691	2.4411078	2.00241162	-1.681279	-0.7495592	0.034968787	1.45631943	192	ankyrin repeat domain 16 [Source:HGNC Symbol;Acc:23471]	ANKRD16	54522
ENSG00000156273	118.790395	92.3442717	31.4161017	21.9105284	1.35602221	0.4393808	0.035060108	1.45518675	584	BTB and CNC homology 1, basic leucine zipper transcription factor 1 [Source:HGNC Symbol;Acc:935]	BACH1	100379661
ENSG00000144677	15.5467445	9.50296577	10.8083682	5.34316801	1.63993139	0.71363546	0.035236233	1.45301053	212	CTD (carboxy-terminal domain, RNA polymerase II, polypeptide A) small phosphatase-like [Source:HGNC Symbol;Acc:16890]	CTDSPL	10217
ENSG00000162882	1.82494298	1.63925161	1.38850284	2.19176912	1.93260202	0.95054457	0.035769222	1.44649051	119	3-hydroxyanthranilate 3,4-dioxygenase [Source:HGNC Symbol;Acc:4796]	HAAO	23498
ENSG00000105374	96.3270566	144.136073	67.5246134	64.4294049	-1.4983499	-0.5833745	0.035779707	1.44636322	322	natural killer cell group 7 sequence [Source:HGNC Symbol;Acc:7830]	NKG7	4818
ENSG00000138293	319.064846	246.981773	95.5809865	69.7043269	1.36568104	0.44962058	0.035998564	1.44371482	550	nuclear receptor coactivator 4 [Source:HGNC Symbol;Acc:7671]	NCOA4	8031
ENSG00000130150	45.8355264	33.9205793	20.4081806	14.7678971	1.44968034	0.53573482	0.036122541	1.4422217	375	motile sperm domain containing 2 [Source:HGNC Symbol;Acc:28381]	MOSPD2	158747
ENSG00000161911	19.239284	11.9007854	16.0802954	8.59598265	1.6863407	0.75389604	0.036674338	1.43563771	193	triggering receptor expressed on myeloid cells-like 1 [Source:HGNC Symbol;Acc:20434]	TREML1	340205
ENSG00000167100	2.39172688	1.1839314	2.14453597	1.14933428	1.9720123	0.97966855	0.036737954	1.43488503	114	sterile alpha motif domain containing 14 [Source:HGNC Symbol;Acc:27312]	SAMD14	201191
ENSG00000115423	1.41502566	2.20643355	1.62439743	1.84157281	-1.9192969	-0.9405779	0.036800984	1.43414057	121	dynein, axonemal, heavy chain 6 [Source:HGNC Symbol;Acc:2951]	DNAH6	1768
ENSG00000154814	55.6546697	82.4977946	20.239112	31.9319033	-1.4172459	-0.5030901	0.036843023	1.43364474	433	oxidoreductase NAD-binding domain containing 1 [Source:HGNC Symbol;Acc:25128]	OXNAD1	92106
ENSG00000118496	17.8784553	13.6867162	6.40732472	6.32023753	1.41938052	0.50526142	0.036853964	1.43351579	428	F-box protein 30 [Source:HGNC Symbol;Acc:15600]	FBXO30	84085
ENSG00000270672	3.69343931	2.139425	2.73538919	1.70019596	1.80418896	0.85135044	0.037491484	1.42606736	153	MT-RNR2-like 6 [Source:HGNC Symbol;Acc:37163]	MTRNR2L6	100463482
ENSG00000137720	2.70452476	4.19330607	1.89764631	2.43668156	-1.6648707	-0.7354101	0.037502636	1.4259382	205	chromosome 11 open reading frame 1 [Source:HGNC Symbol;Acc:1163]	C11orf1	64776
ENSG00000165682	9.95841978	6.02781016	7.76883326	5.44825587	1.65976119	0.73097568	0.037684025	1.42384272	208	C-type lectin domain family 1, member B [Source:HGNC Symbol;Acc:24356]	CLEC1B	51266
ENSG00000149131	14.3435282	6.05473362	24.3459207	7.9749508	2.19064758	1.13135741	0.037690041	1.42377339	98	serpin peptidase inhibitor, clade G (C1 inhibitor), member 1	SERPING1	710

										[Source:HGNC Symbol;Acc:1228]		
ENSG00000215883	1.69423838	2.62281763	1.56781475	1.76691776	-1.7819003	-0.8334166	0.038335418	1.4163998	166	cytochrome b5 reductase-like [Source:HGNC Symbol;Acc:32220]	CYB5RL	606495
ENSG00000181619	2.94332766	1.93645766	1.86360621	1.98898922	1.78213804	0.83360909	0.038527557	1.41422853	167	G protein-coupled receptor 135 [Source:HGNC Symbol;Acc:19991]	GPR135	64582
ENSG00000163462	1.13435489	1.82346664	1.31872845	1.44255459	-1.8805642	-0.9111656	0.038610949	1.41328953	131	tripartite motif containing 46 [Source:HGNC Symbol;Acc:19019]	TRIM46	80128
ENSG00000162545	3.8285004	5.79843816	2.93048019	2.9371641	-1.6821758	-0.7503285	0.038660383	1.41273385	197	calcium/calmodulin-dependent protein kinase II inhibitor 1 [Source:HGNC Symbol;Acc:24190]	CAMK2N1	55450
ENSG00000111644	25.6133742	16.1184096	18.4494747	9.38933992	1.60241263	0.6802457	0.039029379	1.40860836	244	acrosin binding protein [Source:HGNC Symbol;Acc:17195]	ACRBP	84519
ENSG00000184205	29.0884518	46.264976	13.9654319	24.5374875	-1.485172	-0.57063	0.039178771	1.4069492	343	TSPY-like 2 [Source:HGNC Symbol;Acc:24358]	TSPYL2	64061
ENSG00000120217	7.04091246	4.49810326	5.93460918	3.96454798	1.83109864	0.87270951	0.039354658	1.40500386	149	CD274 molecule [Source:HGNC Symbol;Acc:17635]	CD274	29126
ENSG00000135838	28.389345	20.1231182	13.9797527	8.78572603	1.47696014	0.56263089	0.039524063	1.40313842	353	N-acetylneuraminate pyruvate lyase (dihydrodipicolinate synthase) [Source:HGNC Symbol;Acc:16781]	NPL	80896
ENSG00000118113	4.07582966	1.01699309	7.40511204	1.18748809	2.28767394	1.19388144	0.039836851	1.399715	94	matrix metallopeptidase 8 (neutrophil collagenase) [Source:HGNC Symbol;Acc:7175]	MMP8	4317
ENSG00000121858	243.311264	161.414871	165.531639	64.8554337	1.47937316	0.56498601	0.040058388	1.39730653	350	tumor necrosis factor (ligand) superfamily, member 10 [Source:HGNC Symbol;Acc:11925]	TNFSF10	8743
ENSG00000074416	23.467017	15.9637691	13.0711784	8.22523019	1.53314301	0.61649227	0.040297132	1.39472586	296	monoglyceride lipase [Source:HGNC Symbol;Acc:17038]	MGLL	11343
ENSG00000100385	29.4760085	42.4996243	13.8190774	15.372572	-1.4298516	-0.5158654	0.04078721	1.389476	420	interleukin 2 receptor, beta [Source:HGNC Symbol;Acc:6009]	IL2RB	3560
ENSG00000115828	42.0007181	28.0858301	26.4082393	15.8250381	1.55277311	0.63484704	0.041011758	1.38709161	281	glutaminyl-peptide cyclotransferase [Source:HGNC Symbol;Acc:9753]	QPCT	25797
ENSG00000143207	42.1832398	32.2943489	12.856976	11.1404386	1.39005	0.47513677	0.041150301	1.38562698	503	ring finger and WD repeat domain 2, E3 ubiquitin protein ligase [Source:HGNC Symbol;Acc:17440]	RFWD2	64326
ENSG00000159496	9.14953405	5.95374691	5.72174444	3.82238577	1.65420875	0.7261413	0.041384963	1.38315743	219	ral guanine nucleotide dissociation stimulator-like 4 [Source:HGNC Symbol;Acc:31911]	RGL4	266747

ENSG00000166963	11.4063962	7.36349542	8.2942045	3.99083442	1.59303998	0.67178247	0.041517226	1.38177167	254	microtubule-associated protein 1A [Source:HGNC Symbol;Acc:6835]	MAP1A	4130
ENSG00000165914	5.37495403	3.42129761	3.85889004	2.81670422	1.80849788	0.85479191	0.041564016	1.38128249	162	tetratricopeptide repeat domain 7B [Source:HGNC Symbol;Acc:19858]	TTC7B	145567
ENSG00000183837	7.00644018	11.0875087	4.798039	7.30194199	-1.6110189	-0.6879734	0.04197076	1.37705317	245	paraneoplastic Ma antigen 3 [Source:HGNC Symbol;Acc:18742]	PNMA3	29944
ENSG00000150672	1.68977019	3.02610937	1.79086851	2.29957587	-1.8080001	-0.8543948	0.042487052	1.37174341	164	discs, large homolog 2 (Drosophila) [Source:HGNC Symbol;Acc:2901]	DLG2	1740
ENSG00000049323	20.8629741	12.6343519	15.4620363	8.49034878	1.67278811	0.74225471	0.042517884	1.37142835	215	latent transforming growth factor beta binding protein 1 [Source:HGNC Symbol;Acc:6714]	LTBP1	4052
ENSG00000168497	412.940092	255.882638	325.579158	216.133699	1.70001638	0.76554864	0.042579384	1.37080063	199	serum deprivation response [Source:HGNC Symbol;Acc:10690]	SDPR	8436
ENSG00000067646	1.68228527	5.7157485	4.76002522	8.94712292	-2.5454884	-1.3479425	0.042630987	1.37027461	99	zinc finger protein, Y-linked [Source:HGNC Symbol;Acc:12870]	ZFY	7544
ENSG00000198003	2.1464293	1.42052818	1.87514328	1.6009123	1.94413497	0.95912838	0.042646989	1.37011163	132	coiled-coil domain containing 151 [Source:HGNC Symbol;Acc:28303]	CCDC151	115948
ENSG00000079308	2.66064559	1.5296473	2.28969297	1.65857867	1.95101881	0.96422769	0.042695262	1.36962032	130	tensin 1 [Source:HGNC Symbol;Acc:11973]	TNS1	7145
ENSG00000188001	3.65403322	5.46507775	2.71439324	3.50941655	-1.6556888	-0.7274315	0.042953841	1.366998	223	tumor protein p63 regulated 1 [Source:HGNC Symbol;Acc:24759]	TPRG1	285386
ENSG00000143297	21.4176085	29.4039465	15.4684646	21.9547394	-1.9441875	-0.9591674	0.043622162	1.36029281	134	Fc receptor-like 5 [Source:HGNC Symbol;Acc:18508]	FCRL5	83416
ENSG00000255823	57.3819533	44.0819585	93.4589247	108.4633	1.86915983	0.90238994	0.043904202	1.35749391	150	MT-RNR2-like 8 [Source:HGNC Symbol;Acc:37165]	MTRNR2L8	100463486
ENSG00000161381	6.27085662	9.34290966	3.93235385	4.13661285	-1.4954331	-0.5805634	0.044022959	1.35632077	341	plexin domain containing 1 [Source:HGNC Symbol;Acc:20945]	PLXDC1	57125
ENSG00000129824	20.1689904	60.0115818	62.1744622	96.0463771	-3.9937209	-1.9977335	0.044670058	1.34998349	87	ribosomal protein S4, Y-linked 1 [Source:HGNC Symbol;Acc:10425]	RPS4Y1	6192
ENSG00000143409	28.3514956	21.5028115	11.7614119	8.65610593	1.43297852	0.51901698	0.044926435	1.34749804	422	family with sequence similarity 63, member A [Source:HGNC Symbol;Acc:25648]	FAM63A	55793
ENSG00000171914	1.54118276	0.99822059	1.08898731	1.15297148	1.77112777	0.82466829	0.044933795	1.3474269	183	talin 2 [Source:HGNC Symbol;Acc:15447]	TLN2	83660
ENSG00000110400	5.14681701	3.5821801	3.0150085	2.50801004	1.62261604	0.69832166	0.045021975	1.34657546	246	poliovirus receptor-related 1 (herpesvirus entry mediator C) [Source:HGNC Symbol;Acc:9706]	PVRL1	5818

ENSG00000067048	20.6191254	57.1336056	62.15539	87.666415	-3.9016488	-1.9640839	0.04504765	1.34632786	93	DEAD (Asp-Glu-Ala-Asp) box helicase 3, Y-linked [Source:HGNC Symbol;Acc:2699]	DDX3Y	8653
ENSG00000013392	1.92267273	2.70566595	1.41293212	1.54256644	-1.6671272	-0.7373642	0.045110545	1.34572192	222	RWD domain containing 2A [Source:HGNC Symbol;Acc:21385]	RWDD2A	112611
ENSG00000101247	5.14370079	7.59215363	2.1911688	3.02222996	-1.425941	-0.5119143	0.0451192	1.34563861	439	NADH dehydrogenase (ubiquinone) complex I, assembly factor 5 [Source:HGNC Symbol;Acc:15899]	NDUFAF5	79133
ENSG00000072952	42.1135831	28.5054928	25.4622288	19.6615466	1.53537362	0.61858976	0.04526455	1.3442418	313	murine retrovirus integration site 1 homolog [Source:HGNC Symbol;Acc:7237]	MRVI1	10335
ENSG00000174175	10.5566094	6.42623381	7.15870829	4.48693897	1.66617097	0.73653645	0.04546826	1.34229166	224	selectin P (granule membrane protein 140kDa, antigen CD62) [Source:HGNC Symbol;Acc:10721]	SELP	6403
ENSG00000121807	68.5116169	44.1023307	41.4273028	19.142979	1.53589063	0.61907549	0.045581387	1.34121246	311	chemokine (C-C motif) receptor 2 [Source:HGNC Symbol;Acc:1603]	CCR2	729230
ENSG00000181274	178.978428	126.445078	103.503837	78.2684763	1.49561606	0.58073987	0.0462211	1.33515973	346	frequently rearranged in advanced T-cell lymphomas 2 [Source:HGNC Symbol;Acc:16048]	FRAT2	23401
ENSG00000163297	86.1395594	66.1549591	27.1128522	21.448008	1.38206328	0.46682367	0.046310661	1.33431902	534	anthrax toxin receptor 2 [Source:HGNC Symbol;Acc:21732]	ANTXR2	118429
ENSG00000114450	25.3203598	19.6217719	7.64399738	5.7181932	1.35965384	0.4432394	0.046429186	1.33320893	599	guanine nucleotide binding protein (G protein), beta polypeptide 4 [Source:HGNC Symbol;Acc:20731]	GNB4	59345
ENSG00000178935	9.31067949	6.43223652	4.79373539	3.58501904	1.53718459	0.62029042	0.046518267	1.33237647	312	zinc finger protein 552 [Source:HGNC Symbol;Acc:26135]	ZNF552	79818
ENSG00000151208	3.08050942	4.99446598	2.01893643	2.86299312	-1.5797995	-0.6597414	0.046627374	1.33135904	274	discs, large homolog 5 (Drosophila) [Source:HGNC Symbol;Acc:2904]	DLG5	9231
ENSG00000188906	412.146635	295.448295	187.695805	122.100478	1.44705634	0.53312109	0.046784361	1.3298993	401	leucine-rich repeat kinase 2 [Source:HGNC Symbol;Acc:18618]	LRRK2	120892
ENSG00000183878	4.61183803	12.7720217	13.0950677	20.4908987	-2.8792903	-1.5257132	0.046981	1.32807775	107	ubiquitously transcribed tetratricopeptide repeat containing, Y-linked [Source:HGNC Symbol;Acc:12638]	UTY	7404
ENSG00000188921	73.4964276	58.5515133	18.2911119	16.5284438	1.34097968	0.42328737	0.047149586	1.32652211	671	protein tyrosine phosphatase-like A domain containing 2 [Source:HGNC Symbol;Acc:20920]	PTPLAD2	401494

ENSG00000186407	104.417914	76.9553083	50.2408341	25.7858179	1.40461015	0.49016976	0.047174221	1.32629527	486	CD300e molecule [Source:HGNC Symbol;Acc:28874]	CD300E	342510
ENSG00000139610	1.0737529	1.5851988	1.32401851	1.10482822	-1.7280388	-0.7891356	0.047217144	1.32590028	201	chymotrypsin-like elastase family, member 1 [Source:HGNC Symbol;Acc:3308]	CELA1	1990
ENSG00000070190	161.995889	125.007246	58.7496495	52.6640281	1.4166463	0.5024796	0.047271187	1.3254035	462	dual adaptor of phosphotyrosine and 3-phosphoinositides [Source:HGNC Symbol;Acc:16500]	DAPP1	27071
ENSG00000183092	1.86720458	1.23992314	1.91836775	1.73514014	2.01149936	1.00827128	0.047288571	1.32524381	137	brain-enriched guanylate kinase-associated [Source:HGNC Symbol;Acc:24163]	BEGAIN	57596
ENSG00000137801	40.3319449	21.7980339	39.5315469	14.733531	1.69614685	0.76226108	0.047334513	1.32482209	217	thrombospondin 1 [Source:HGNC Symbol;Acc:11785]	THBS1	7057
ENSG00000122862	1462.71015	1026.62452	777.467256	450.43342	1.45154662	0.53759091	0.047493514	1.3233657	403	serglycin [Source:HGNC Symbol;Acc:9361]	SRGN	5552
ENSG00000139187	59.0234362	87.6401303	32.9837046	40.8127921	-1.4601348	-0.5461016	0.047611205	1.32229083	393	killer cell lectin-like receptor subfamily G, member 1 [Source:HGNC Symbol;Acc:6380]	KLRG1	10219
ENSG00000066735	2.89041159	1.25292321	4.21004685	2.20139769	2.19802964	1.13621084	0.048044113	1.31835982	127	kinesin family member 26A [Source:HGNC Symbol;Acc:20226]	KIF26A	26153
ENSG00000117115	59.3688808	41.0268596	38.2164856	28.8705671	1.57375753	0.65421328	0.048211435	1.31684994	283	peptidyl arginine deiminase, type II [Source:HGNC Symbol;Acc:18341]	PADI2	11240
ENSG00000123610	17.2357605	10.4075998	16.2506595	11.8554049	1.79602212	0.84480512	0.048332675	1.31575917	184	tumor necrosis factor, alpha-induced protein 6 [Source:HGNC Symbol;Acc:11898]	TNFAIP6	7130
ENSG00000189233	3.72595968	5.62769726	3.25226197	2.96472923	-1.5893959	-0.6684786	0.049000873	1.30979618	275	nuclear GTPase, germinal center associated [Source:HGNC Symbol;Acc:33550]	NUGGC	389643
ENSG00000156475	12.3279537	18.4529804	6.47625886	8.18511856	-1.4219047	-0.5078248	0.049020878	1.30961892	455	protein phosphatase 2, regulatory subunit B, beta [Source:HGNC Symbol;Acc:9305]	PPP2R2B	5521
ENSG00000149548	5.68862286	8.51782883	2.41102022	3.65821263	-1.429941	-0.5159556	0.049158598	1.30840051	443	coiled-coil domain containing 15 [Source:HGNC Symbol;Acc:25798]	CCDC15	80071
ENSG00000221909	9.64949323	6.50426762	5.54133456	3.0566423	1.51732728	0.6015323	0.049195196	1.30807731	339	family with sequence similarity 200, member A [Source:HGNC Symbol;Acc:25401]	FAM200A	221786
ENSG00000129270	1.14280295	2.03909697	1.37159483	1.73213698	-1.8839539	-0.9137637	0.049382629	1.30642579	160	matrix metallopeptidase 28 [Source:HGNC Symbol;Acc:14366]	MMP28	79148
ENSG00000205038	2.38999728	0.91922543	2.80970445	1.14063021	2.03899726	1.02785984	0.049405971	1.30622056	138	polycystic kidney and hepatic disease 1 (autosomal recessive)-	PKHD1L1	93035

										like 1 [Source:HGNC Symbol;Acc:20313]		
ENSG00000145912	20.7636545	29.5808269	7.61416088	9.47081291	-1.36039	-0.4440203	0.049998116	1.30104636	615	NHP2 ribonucleoprotein [Source:HGNC Symbol;Acc:14377]	NHP2	55651

Compared populations:

ALL RA vs HC

Fold change describes the size of the difference in gene expression (given in mean and standar deviation, sd) between the compared groups. The values are always in relation to the group used as a base level group (reference). P-value describes the reliability of the change in expression value between the compared groups. Results were considered significant with a fold change of 1.2 and a p-value cutoff of 0.05.

Supplementary Material 15 – Summary of enrichement analysis of DE genes listing the top 25 processes or pathways based different databases (Gene Ontology, Reactome)

Gene Ontology Biological Process					
**GO.ID**	**Term**	**Annotated**	**Significant**	**Expected**	**p value**
GO:0032501	multicellular organismal process	4001	134	92.1	0.000000023
GO:0042060	wound healing	265	22	6.1	0.00000018
GO:0015671	oxygen transport	8	5	0.18	0.00000033
GO:0007599	hemostasis	156	16	3.59	0.00000057
GO:0034330	cell junction organization	381	26	8.77	0.00000066
GO:0015669	gas transport	9	5	0.21	0.00000072
GO:0009611	response to wounding	337	24	7.76	0.00000085
GO:0042744	hydrogen peroxide catabolic process	16	6	0.37	0.00000092
GO:0015670	carbon dioxide transport	5	4	0.12	0.0000013
GO:0007596	blood coagulation	150	15	3.45	0.0000018
GO:0050817	coagulation	151	15	3.48	0.0000019
GO:0006954	inflammatory response	538	31	12.38	0.0000021
GO:0019755	one-carbon compound transport	28	7	0.64	0.0000025
GO:0050729	positive regulation of inflammatory resp...	99	12	2.28	0.0000026
GO:0050896	response to stimulus	5091	152	117.19	0.0000034
GO:0002376	immune system process	1719	67	39.57	0.0000047
GO:0034329	cell junction assembly	211	17	4.86	0.0000072
GO:0007166	cell surface receptor signaling pathway	1645	64	37.87	0.0000089
GO:0050878	regulation of body fluid levels	220	17	5.06	0.000013
GO:0010572	positive regulation of platelet activati...	9	4	0.21	0.000032
GO:0050808	synapse organization	237	17	5.46	0.000033
GO:0023052	signaling	3561	112	81.97	0.000034
GO:0007154	cell communication	3619	113	83.31	0.000043
GO:0007165	signal transduction	3339	106	76.86	0.000045

Reactome				
Reactome.ID	Term	GeneRatio	BgRatio	p value
R-HSA-109582	Hemostasis	36/160	418/6988	1.90389E-12
R-HSA-76002	Platelet activation, signaling and aggregation	22/160	191/6988	2.76778E-10
R-HSA-114608	Platelet degranulation	12/160	89/6988	7.10628E-07
R-HSA-76005	Response to elevated platelet cytosolic Ca2+	12/160	93/6988	1.15224E-06
R-HSA-76009	Platelet Aggregation (Plug Formation)	5/160	22/6988	0.000113554
R-HSA-500792	GPCR ligand binding	12/160	152/6988	0.00017531
R-HSA-140877	Formation of Fibrin Clot (Clotting Cascade)	4/160	18/6988	0.00063037
R-HSA-983231	Factors involved in megakaryocyte development and platelet production	9/160	108/6988	0.000778916
R-HSA-373076	Class A/1 (Rhodopsin-like receptors)	9/160	114/6988	0.001148667
R-HSA-372708	p130Cas linkage to MAPK signaling for integrins	3/160	10/6988	0.001255916
R-HSA-9651496	Defects of contact activation system (CAS) and kallikrein/kinin system (KKS)	3/160	10/6988	0.001255916
R-HSA-9671793	Diseases of hemostasis	3/160	10/6988	0.001255916
R-HSA-140837	Intrinsic Pathway of Fibrin Clot Formation	3/160	11/6988	0.001697986
R-HSA-354194	GRB2:SOS provides linkage to MAPK signaling for Integrins	3/160	11/6988	0.001697986
R-HSA-75892	Platelet Adhesion to exposed collagen	3/160	12/6988	0.002226136
R-HSA-6803157	Antimicrobial peptides	4/160	25/6988	0.002300257
R-HSA-6794361	Neurexins and neuroligins	4/160	26/6988	0.002670595
R-HSA-372790	Signaling by GPCR	16/160	323/6988	0.00284371
R-HSA-9660821	ADORA2B mediated anti-inflammatory cytokines production	4/160	27/6988	0.003079858
R-HSA-3000170	Syndecan interactions	3/160	15/6988	0.004377485
R-HSA-8936459	RUNX1 regulates genes involved in megakaryocyte differentiation and platelet function	5/160	48/6988	0.004573948
R-HSA-114604	GPVI-mediated activation cascade	4/160	31/6988	0.005143807
R-HSA-418594	G alpha (i) signalling events	9/160	147/6988	0.006433534
R-HSA-1566948	Elastic fibre formation	3/160	18/6988	0.007465524
R-HSA-163359	Glucagon signaling in metabolic regulation	3/160	19/6988	0.008718248

Compared populations:

ALL RA vs HC

Enrichment analysis for the differentially expressed filtered gene lists tests whether the genes within a certain GO term or Reactome pathway are statistically over-represented in a given comparison. Annotated: number of genes associated with the given term. R: Reactome database. Significant: number of statistically significant associated with the given term. Expected: expected number of genes associated with the given term in the DE gene list. Gene ratio: ratio of number of genes in the DE list that overlap with genes associated with the given term, and the number of genes in the DE list which overlap with genes associated with all terms in the Reactome database. Bg ratio: ratio of the number of genes associated with the given term, and the total number of genes associated with Reactome terms

Supplementary Material 16 - List of differentially expressed (DE) genes

ID	mean_D2T_RA	mean_Normal_RA	sd_D2T_RA	sd_Normal_RA	FC	logFC	p value	avgRank	Description	geneName	EntrezID
ENSG00000142621	0.702934903	2.408501637	1.043732195	3.121225377	-3.109639209	-1.636747204	0.0043969211	1	forkhead-associated (FHA) phosphopeptide binding domain 1 [Source:HGNC Symbol;Acc:29408]	FHAD1	114827
ENSG00000204311	0.806085252	1.472189113	1.273419159	0.94976692	-2.525580031	-1.336614759	0.0052767606	2	deafness, autosomal recessive 59 [Source:HGNC Symbol;Acc:29502]	DFNB59	494513
ENSG00000225614	1.1797002	2.393571336	1.066925539	1.619299961	-2.258709221	-1.175498555	0.0055505960	12	zinc finger protein 469 [Source:HGNC Symbol;Acc:23216]	ZNF469	84627
ENSG00000117479	1.050936032	1.980663864	1.295259816	1.691601916	-2.589223474	-1.372519489	0.0056374386	3	solute carrier family 19 (thiamine transporter), member 2 [Source:HGNC Symbol;Acc:10938]	SLC19A2	10560
ENSG00000101977	2.133275693	3.583877331	1.779468102	1.543028783	-2.283897048	-1.191497619	0.0063927269	10	MCF.2 cell line derived transforming sequence [Source:HGNC Symbol;Acc:6940]	MCF2	4168
ENSG00000100908	2.244960426	0.924770744	1.516314941	0.773104651	2.41177403	1.270094741	0.0075832124	5	ER membrane protein complex subunit 9 [Source:HGNC Symbol;Acc:20273]	EMC9	51016
ENSG00000163687	1.227264034	2.567766891	1.459575425	2.71375072	-2.650388671	-1.406203942	0.0082173657	4	deoxyribonuclease I-like 3 [Source:HGNC Symbol;Acc:2959]	DNASE1L3	1776
ENSG00000133424	1.6939056	2.79382841	1.442779568	1.640204111	-2.41005025	-1.269063227	0.0082594885	7	like-glycosyltransferase [Source:HGNC Symbol;Acc:6511]	LARGE	9215
ENSG00000213213	1.289366031	2.47991502	0.969855622	1.371463597	-2.125192134	-1.087593278	0.0084291344	25	coiled-coil domain containing 183 [Source:HGNC Symbol;Acc:28236]	CCDC183	84960
ENSG00000186868	1.293032852	2.154222293	1.504736308	1.476667076	-2.366961744	-1.243036389	0.0084711395	11	microtubule-associated protein tau [Source:HGNC Symbol;Acc:6893]	MAPT	4137
ENSG00000111249	0.934659729	1.767507256	1.136448024	1.076567126	-2.490559379	-1.316469807	0.0085410581	6	cut-like homeobox 2 [Source:HGNC Symbol;Acc:19347]	CUX2	23316
ENSG00000166025	1.426047548	2.4816983	1.202328081	1.620841752	-2.228931236	-1.156352109	0.0088556650	15	angiomotin like 1 [Source:HGNC Symbol;Acc:17811]	AMOTL1	154810
ENSG00000149554	2.365424483	3.645360399	1.61319704	2.093309362	-2.232847322	-1.158884605	0.0093843944	16	checkpoint kinase 1 [Source:HGNC Symbol;Acc:1925]	CHEK1	1111
ENSG00000197705	2.366940112	3.740814244	2.299262835	2.40748672	-2.467044974	-1.302784016	0.0094942007	8	kelch-like family member 14 [Source:HGNC Symbol;Acc:29266]	KLHL14	57565
ENSG00000013573	7.660918736	11.42628958	6.974245649	5.87479096	-2.386513514	-1.254904506	0.0095905831	13	DEAD/H (Asp-Glu-Ala-Asp/His) box helicase 11 [Source:HGNC Symbol;Acc:2736]	DDX11	1663
ENSG00000066923	1.118090169	2.331424208	0.945193439	2.075096473	-2.170167739	-1.117806557	0.0108461335	23	stromal antigen 3 [Source:HGNC Symbol;Acc:11356]	STAG3	10734
ENSG00000163806	3.021340354	4.299232453	3.194132439	2.395924145	-2.331414075	-1.22120526	0.0109613741	14	speedy/RINGO cell cycle regulator family member A [Source:HGNC Symbol;Acc:30613]	SPDYA	245711
ENSG00000187990	1.452946942	2.432235243	2.275192935	2.01117235	-2.553407224	-1.352423641	0.0111425970	9	histone cluster 1, H2bg [Source:HGNC Symbol;Acc:4746]	HIST1H2BG	8347
ENSG00000137269	1.835439663	3.117967189	1.57581546	1.54884589	-2.168756716	-1.116868225	0.0111658709	24	leucine rich repeat containing 1 [Source:HGNC Symbol;Acc:14307]	LRRC1	55227
ENSG00000132386	2.896035418	4.798947288	1.946223485	2.887498137	-1.920534338	-0.941507759	0.0115444619	47	serpin peptidase inhibitor, clade F (alpha-2 antiplasmin, pigment epithelium	SERPINF1	5176

									derived factor), member 1 [Source:HGNC Symbol;Acc:8824]		
ENSG00000102796	12.78900365	7.256880134	6.771923403	3.058256536	1.693284082	0.759824034	0.0125442556	99	dehydrogenase/reductase (SDR family) member 12 [Source:HGNC Symbol;Acc:25832]	DHRS12	79758
ENSG00000188215	5.187472702	7.536654048	2.582101256	2.143428346	-1.625312849	-0.700717442	0.0127876785	135	DCN1, defective in cullin neddylation 1, domain containing 3 [Source:HGNC Symbol;Acc:28734]	DCUN1D3	123879
ENSG00000171766	1.484595844	2.290647481	1.366630063	1.10659889	-2.268514536	-1.181747904	0.0141597221	19	glycine amidinotransferase (L-arginine:glycine amidinotransferase) [Source:HGNC Symbol;Acc:4175]	GATM	2628
ENSG00000079482	1.772735807	2.749228808	1.579112131	1.420225344	-2.172409614	-1.119296153	0.0142287939	27	oligophrenin 1 [Source:HGNC Symbol;Acc:8148]	OPHN1	4983
ENSG00000157168	11.25918978	6.169427544	6.102259219	4.098046105	1.944611807	0.959482186	0.0145100352	45	neuregulin 1 [Source:HGNC Symbol;Acc:7997]	NRG1	3084
ENSG00000163814	1.263825874	2.229873519	1.519859567	1.435591977	-2.18508027	-1.127686279	0.0155058356	26	CUB domain containing protein 1 [Source:HGNC Symbol;Acc:24357]	CDCP1	64866
ENSG00000050438	4.708419049	6.657112625	2.873754216	2.5190116	-1.702427966	-0.767593755	0.0156842409	97	solute carrier family 4, sodium bicarbonate cotransporter, member 8 [Source:HGNC Symbol;Acc:11034]	SLC4A8	9498
ENSG00000179088	2.711870751	4.459958839	1.897029364	2.623270172	-1.953888579	-0.9663482	0.0167533848	46	chromosome 12 open reading frame 42 [Source:HGNC Symbol;Acc:24729]	C12orf42	374470
ENSG00000172260	2.114373555	3.602949638	1.773080462	2.077354564	-2.361004479	-1.239400778	0.0167861952	18	neuronal growth regulator 1 [Source:HGNC Symbol;Acc:17302]	NEGR1	257194
ENSG00000154655	3.905343689	5.218415958	3.57701953	2.580216093	-1.953400069	-0.965987453	0.0170992922	49	l(3)mbt-like 4 (Drosophila) [Source:HGNC Symbol;Acc:26677]	L3MBTL4	91133
ENSG00000074590	1.702491601	3.551979564	1.667736482	2.44938326	-2.303824795	-1.204031005	0.0172174539	20	NUAK family, SNF1-like kinase, 1 [Source:HGNC Symbol;Acc:14311]	NUAK1	9891
ENSG00000221838	4.720381299	7.061195168	2.688233091	1.976611077	-1.637089346	-0.711133061	0.0188602339	129	adaptor-related protein complex 4, mu 1 subunit [Source:HGNC Symbol;Acc:574]	AP4M1	9179
ENSG00000171385	0.904203788	2.081325278	1.074557553	1.696246339	-2.477519619	-1.308896482	0.0192634427	17	potassium voltage-gated channel, Shal-related subfamily, member 3 [Source:HGNC Symbol;Acc:6239]	KCND3	3752
ENSG00000166762	1.855513838	3.532780249	1.485068194	2.001812432	-2.127577274	-1.089211532	0.0193821940	34	cation channel, sperm associated 2 [Source:HGNC Symbol;Acc:18810]	CATSPER2	101930343
ENSG00000135747	1.250488189	2.395879856	1.044261426	1.606668651	-2.191068105	-1.131634328	0.0201129774	29	zinc finger protein 670 [Source:HGNC Symbol;Acc:28167]	ZNF670	93474
ENSG00000184305	3.747313153	5.14923793	3.139176613	2.625539678	-1.995786023	-0.996957051	0.0209321013	44	coiled-coil serine-rich protein 1 [Source:HGNC Symbol;Acc:29349]	CCSER1	401145
ENSG00000135482	4.326969696	2.583866436	2.313972921	1.621839764	1.830227456	0.872022954	0.0215848225	72	zinc finger CCCH-type containing 10 [Source:HGNC Symbol;Acc:25893]	ZC3H10	84872
ENSG00000146072	1.397215899	2.504497219	1.136726981	1.713127798	-2.108427556	-1.076167453	0.0217331576	37	tumor necrosis factor receptor superfamily, member 21 [Source:HGNC Symbol;Acc:13469]	TNFRSF21	27242
ENSG00000129696	5.830277151	3.392517626	3.159062679	2.482311604	1.727498979	0.788684858	0.0220640957	95	TELO2 interacting protein 2 [Source:HGNC Symbol;Acc:26262]	TTI2	80185
ENSG00000196169	1.959085268	3.711561366	2.558737736	4.017138195	-2.402349996	-1.264446351	0.0222320695	21	kinesin family member 19 [Source:HGNC Symbol;Acc:26735]	KIF19	124602
ENSG00000116678	8.413872631	12.04503455	7.127593315	5.926255797	-1.855016313	-0.891431874	0.0226793339	65	leptin receptor [Source:HGNC Symbol;Acc:6554]	LEPR	54741

ENSG00000177483	2.279453603	3.345668294	1.567630442	1.027980085	-1.860320563	-0.895551242	0.0227505484	63	RNA binding motif protein 44 [Source:HGNC Symbol;Acc:24756]	RBM44	375316
ENSG00000117016	0.923230465	1.667477314	1.071192307	1.129501812	-2.221832946	-1.151750348	0.0230678607	30	regulating synaptic membrane exocytosis 3 [Source:HGNC Symbol;Acc:21292]	RIMS3	9783
ENSG00000155755	1.426309883	2.069714262	1.260707525	1.078207813	-1.94296492	-0.958259853	0.0232468893	56	transmembrane protein 237 [Source:HGNC Symbol;Acc:14432]	TMEM237	65062
ENSG00000164330	9.989565032	14.41268213	7.990533423	8.818920202	-2.190975836	-1.131573573	0.0233983884	32	early B-cell factor 1 [Source:HGNC Symbol;Acc:3126]	EBF1	1879
ENSG00000214595	3.448240407	5.510900335	3.303667871	5.879562089	-2.128942802	-1.09013719	0.0234433071	39	echinoderm microtubule associated protein like 6 [Source:HGNC Symbol;Acc:35412]	EML6	400954
ENSG00000108515	3.027475843	1.46108291	2.453766106	1.458687555	2.170094731	1.117758022	0.0236815370	36	enolase 3 (beta, muscle) [Source:HGNC Symbol;Acc:3354]	ENO3	2027
ENSG00000163536	3.912905158	4.750771047	4.17368047	2.076385015	-2.382601476	-1.25253766	0.0255299155	28	serpin peptidase inhibitor, clade I (neuroserpin), member 1 [Source:HGNC Symbol;Acc:8943]	SERPINI1	5274
ENSG00000175544	2.310576397	3.655934354	2.203807748	2.355691796	-2.166076429	-1.115084149	0.0257372011	38	calcium binding protein 4 [Source:HGNC Symbol;Acc:1386]	CABP4	57010
ENSG00000167550	2.197607355	3.970336143	1.527346394	1.745184269	-1.966244134	-0.975442462	0.0260214709	54	Ras homolog enriched in brain like 1 [Source:HGNC Symbol;Acc:21166]	RHEBL1	121268
ENSG00000134489	0.946742344	1.96968495	0.928074325	1.740525626	-2.207915481	-1.142684947	0.0262160317	33	histamine receptor H4 [Source:HGNC Symbol;Acc:17383]	HRH4	59340
ENSG00000160307	2.432283249	6.047477156	3.076478631	6.456476901	-2.793774698	-1.482215681	0.0263633869	22	S100 calcium binding protein B [Source:HGNC Symbol;Acc:10500]	S100B	6285
ENSG00000172264	6.528075953	8.450630943	8.972405398	4.84792728	-2.12728644	-1.089014306	0.0274376438	43	MACRO domain containing 2 [Source:HGNC Symbol;Acc:16126]	MACROD2	140733
ENSG00000166483	5.806894994	8.79287439	3.837873682	5.0918863	-1.646635029	-0.719520822	0.0297528024	133	WEE1 G2 checkpoint kinase [Source:HGNC Symbol;Acc:12761]	WEE1	7465
ENSG00000161800	1.910270606	2.907622086	1.669375536	1.41406088	-1.944257074	-0.959218988	0.0299746461	61	Rac GTPase activating protein 1 [Source:HGNC Symbol;Acc:9804]	RACGAP1	29127
ENSG00000136059	4.744142646	2.812602185	3.662369037	3.126570534	2.052274676	1.037223834	0.0299917080	51	villin-like [Source:HGNC Symbol;Acc:30906]	VILL	50853
ENSG00000168152	1.514620021	2.580490968	1.047252946	1.412875066	-1.893505079	-0.921059291	0.0300774079	66	THAP domain containing 9 [Source:HGNC Symbol;Acc:23192]	THAP9	79725
ENSG00000168913	1.756309891	2.277632503	2.148210992	1.646478416	-2.177375747	-1.122590393	0.0309654368	40	energy homeostasis associated [Source:HGNC Symbol;Acc:24838]	ENHO	375704
ENSG00000164885	9.323095424	6.332336263	3.830961717	3.123561779	1.548168207	0.630562228	0.0310331694	205	cyclin-dependent kinase 5 [Source:HGNC Symbol;Acc:1774]	CDK5	1020
ENSG00000128340	2.102840986	1.173619782	1.259246197	1.048246605	1.940573574	0.956483131	0.0310522662	64	ras-related C3 botulinum toxin substrate 2 (rho family, small GTP binding protein Rac2) [Source:HGNC Symbol;Acc:9802]	RAC2	5880
ENSG00000154380	1.038828748	2.099774432	1.050815118	2.099530581	-2.114489581	-1.080309452	0.0311407528	48	enabled homolog (Drosophila) [Source:HGNC Symbol;Acc:18271]	ENAH	55740
ENSG00000185614	1.760668796	0.805273344	1.852346851	1.178934776	2.395829498	1.260525241	0.0319305537	31	family with sequence similarity 212, member A [Source:HGNC Symbol;Acc:32480]	FAM212A	389119
ENSG00000140025	2.463756889	3.063917561	2.25033368	1.074759565	-1.929690109	-0.948369182	0.0319604456	67	EF-hand calcium binding domain 11 [Source:HGNC Symbol;Acc:20357]	EFCAB11	90141
ENSG00000106537	5.283431444	6.987994784	3.938705542	3.541808702	-1.79915511	-0.84731957	0.0325743303	85	tetraspanin 13 [Source:HGNC Symbol;Acc:21643]	TSPAN13	27075

ENSG00000177025	1.16489047	1.798528672	1.166201731	1.25177568	-2.036206684	-1.025884008	0.0329535837	53	chromosome 19 open reading frame 18 [Source:HGNC Symbol;Acc:28642]	C19orf18	147685
ENSG00000130584	1.229848454	1.852804063	1.605359633	1.526513169	-2.031087769	-1.022252584	0.0330131899	55	zinc finger and BTB domain containing 46 [Source:HGNC Symbol;Acc:16094]	ZBTB46	140685
ENSG00000163221	257.0458107	137.4381711	150.7725372	74.62142933	1.728723958	0.789707518	0.0334396153	113	S100 calcium binding protein A12 [Source:HGNC Symbol;Acc:10489]	S100A12	6283
ENSG00000109099	0.841871667	1.6024155	1.02739907	1.491626201	-2.304377072	-1.204376808	0.0338120494	35	peripheral myelin protein 22 [Source:HGNC Symbol;Acc:9118]	PMP22	5376
ENSG00000084731	3.624935862	2.41199268	1.739885832	2.106336383	1.802385656	0.849907737	0.0339581207	87	kinesin family member 3C [Source:HGNC Symbol;Acc:6321]	KIF3C	3797
ENSG00000114204	0.988438335	1.66162669	1.190520869	1.299436869	-2.198907311	-1.136786792	0.0342458678	42	serpin peptidase inhibitor, clade I (pancpin), member 2 [Source:HGNC Symbol;Acc:8945]	SERPINI2	5276
ENSG00000092067	5.654889915	7.388416715	5.689029362	5.065031772	-2.000635669	-1.000458465	0.0348082308	60	CCAAT/enhancer binding protein (C/EBP), epsilon [Source:HGNC Symbol;Acc:1836]	CEBPE	1053
ENSG00000139182	9.806615895	13.95151977	6.476125287	4.219256458	-1.607605968	-0.684913838	0.0358671500	164	calsyntenin 3 [Source:HGNC Symbol;Acc:18371]	CLSTN3	9746
ENSG00000130147	0.914888854	1.736692229	1.57165595	1.61962761	-2.241101858	-1.164208221	0.0368535641	41	SH3-domain binding protein 4 [Source:HGNC Symbol;Acc:10826]	SH3BP4	23677
ENSG00000090674	6.961816869	9.76984423	3.861870058	3.401174462	-1.634336948	-0.708705452	0.0379413986	149	mucolipin 1 [Source:HGNC Symbol;Acc:13356]	MCOLN1	57192
ENSG00000104863	5.281141259	3.03215142	3.110092681	2.478575181	1.836372782	0.876858955	0.0381289683	80	lin-7 homolog B (C. elegans) [Source:HGNC Symbol;Acc:17788]	LIN7B	64130
ENSG00000100084	3.206734883	4.643137775	2.268331824	1.922180657	-1.748711114	-0.806291977	0.0385361089	107	histone cell cycle regulator [Source:HGNC Symbol;Acc:4916]	HIRA	7290
ENSG00000164171	1.639195231	3.102408138	1.528632387	2.94918455	-2.06075139	-1.043170468	0.0397212837	58	integrin, alpha 2 (CD49B, alpha 2 subunit of VLA-2 receptor) [Source:HGNC Symbol;Acc:6137]	ITGA2	3673
ENSG00000138002	1.923557533	2.763594601	1.584597029	1.480910817	-1.812505306	-0.857985218	0.0433868778	89	intraflagellar transport 172 homolog (Chlamydomonas) [Source:HGNC Symbol;Acc:30391]	IFT172	26160
ENSG00000226742	4.838113232	2.639044236	2.889346686	1.950465713	1.793886408	0.84308854	0.0434891458	94	heat shock factor binding protein 1-like 1 [Source:HGNC Symbol;Acc:37243]	HSBP1L1	440498
ENSG00000136319	10.31352279	14.68379999	4.054058372	4.362364961	-1.492644151	-0.577870265	0.0448089812	262	tetratricopeptide repeat domain 5 [Source:HGNC Symbol;Acc:19274]	TTC5	91875
ENSG00000137441	54.68569226	84.68540439	29.07168169	40.64669105	-1.553197122	-0.635240939	0.0453309482	211	fibroblast growth factor binding protein 2 [Source:HGNC Symbol;Acc:29451]	FGFBP2	83888
ENSG00000174944	8.06327201	13.0454726	4.419292553	7.824690124	-1.634966887	-0.709261418	0.0459898133	151	purinergic receptor P2Y, G-protein coupled, 14 [Source:HGNC Symbol;Acc:16442]	P2RY14	9934
ENSG00000214787	1.79291907	2.467291506	2.016463187	1.544658334	-1.981348931	-0.986482973	0.0463742811	71	membrane-spanning 4-domains, subfamily A, member 4E [Source:HGNC Symbol;Acc:14284]	MS4A4E	643680
ENSG00000113749	33.29108338	19.31815168	19.73091762	9.888267636	1.627723105	0.702855301	0.0480735985	157	histamine receptor H2 [Source:HGNC Symbol;Acc:5183]	HRH2	3274
ENSG00000140950	5.281728199	3.979519171	2.888891476	2.473302951	1.67220253	0.741749591	0.0483717717	136	TBC/LysM-associated domain containing 1 [Source:HGNC Symbol;Acc:29325]	TLDC1	57707
ENSG00000131037	1.029476135	1.460079472	1.335531883	1.174031855	-2.039749615	-1.028392068	0.0495115441	62	EPS8-like 1 [Source:HGNC Symbol;Acc:21295]	EPS8L1	54869

ENSG00000126461	8.783069957	5.453279781	4.097419763	2.052041911	1.587887799	0.667108974	0.0497829035	182	SR-related CTD-associated factor 1 [Source:HGNC Symbol;Acc:30403]	SCAF1	58506

Compared populations:

D2T vs non-D2T RA
Fold change describes the size of the difference in gene expression (given in mean and standard deviation, sd) between the compared groups. The values are always in relation to the group used as a base level group (reference). P-value describes the reliability of the change in expression value between the compared groups. Results were considered significant with a fold change of 1.2 and a p-value cutoff of 0.05.

Supplementary Material 17 - List of differentially expressed (DE) genes

ID	mean_D2T_RA	mean_Control	sd_D2T_RA	sd_Control	FC	logFC	P.Value	avgRank	Description	geneName	EntrezID
ENSG00000244734	4635.925549	1960.828234	4802.401242	4045.268049	4.489971166	2.16670618	7.8676E-05	1	hemoglobin, beta [Source:HGNC Symbol;Acc:4827]	HBB	3043
ENSG00000169877	3.713535262	0.370373351	6.584778669	0.613041637	4.950945903	2.307704185	0.000379157	2	alpha hemoglobin stabilizing protein [Source:HGNC Symbol;Acc:18075]	AHSP	51327
ENSG00000158578	9.380133501	1.529281564	11.94511723	3.300750518	6.452503749	2.689859075	0.001201866	3	aminolevulinate, delta-, synthase 2 [Source:HGNC Symbol;Acc:397]	ALAS2	212
ENSG00000134668	3.462992445	1.111725705	3.005511494	1.210112533	3.522075919	1.816426007	0.000466761	4	SPOC domain containing 1 [Source:HGNC Symbol;Acc:26338]	SPOCD1	90853
ENSG00000206172	195.3124377	90.08131135	195.6855017	138.9281615	3.534261561	1.821408814	0.000868838	5	hemoglobin, alpha 1 [Source:HGNC Symbol;Acc:4823]	HBA1	3040
ENSG00000144290	5.676768457	12.40855762	6.884257477	12.93533602	-3.42462142	-1.775944512	0.001088098	6	solute carrier family 4, sodium bicarbonate transporter, member 10 [Source:HGNC Symbol;Acc:13811]	SLC4A10	57282
ENSG00000088053	10.81736705	5.000609849	7.76730598	4.396086602	2.718406953	1.442761448	0.000322653	7	glycoprotein VI (platelet) [Source:HGNC Symbol;Acc:14388]	GP6	51206
ENSG00000132970	3.847507837	1.741405148	3.449171514	1.857416043	2.903518823	1.537802387	0.000858543	8	WAS protein family, member 3 [Source:HGNC Symbol;Acc:12734]	WASF3	10810
ENSG00000133169	1.584172441	0.342489479	1.642315091	0.528297179	3.178643033	1.668411009	0.001199996	9	brain expressed, X-linked 1 [Source:HGNC Symbol;Acc:1036]	BEX1	55859
ENSG00000206177	6.721994372	1.288112629	10.87677262	1.938452406	4.502695397	2.170788884	0.003028816	10	hemoglobin, mu [Source:HGNC Symbol;Acc:4826]	HBM	3042
ENSG00000155755	1.426309883	2.908152067	1.260707525	1.727239228	-2.53518538	-1.342091245	0.000187796	11	transmembrane protein 237 [Source:HGNC Symbol;Acc:14432]	TMEM237	65062
ENSG00000227184	1.532035208	3.18685128	2.119150588	2.313309574	-2.734969081	-1.451524524	0.001657982	12	epiplakin 1 [Source:HGNC Symbol;Acc:15577]	EPPK1	83481
ENSG00000062524	1.277605772	3.485122284	1.729620465	3.243051464	-2.708381118	-1.437430766	0.001701903	13	leukocyte receptor tyrosine kinase [Source:HGNC Symbol;Acc:6721]	LTK	4058
ENSG00000188536	1048.623005	510.5262346	1150.870831	740.9780011	3.085461719	1.625486395	0.002559457	14	hemoglobin, alpha 2 [Source:HGNC Symbol;Acc:4824]	HBA2	3040
ENSG00000144645	12.84246157	20.9225319	10.7452592	8.57107277	-2.701707646	-1.433871568	0.002320561	15	oxysterol binding protein-like 10 [Source:HGNC Symbol;Acc:16395]	OSBPL10	114884
ENSG00000148498	5.746202751	2.660428965	4.150806283	2.189581355	2.566682562	1.35990488	0.002100479	16	par-3 family cell polarity regulator [Source:HGNC Symbol;Acc:16051]	PARD3	56288
ENSG00000214595	3.448240407	5.319770887	3.303667871	2.294348899	-2.493534049	-1.318191903	0.001700807	17	echinoderm microtubule associated protein like 6 [Source:HGNC Symbol;Acc:35412]	EML6	400954
ENSG00000055732	2.76822987	0.986968116	2.149648078	0.925879363	2.640418464	1.400766592	0.003251791	18	mucolipin 3 [Source:HGNC Symbol;Acc:13358]	MCOLN3	55283

ENSG00000198829	4.626714079	2.085422601	3.475016925	1.628101471	2.412091925	1.270284889	0.002421332	19	succinate receptor 1 [Source:HGNC Symbol;Acc:4542]	SUCNR1	56670
ENSG00000072422	12.49850034	7.054499433	7.792766764	5.979288429	2.406528528	1.266953526	0.002810984	20	Rho-related BTB domain containing 1 [Source:HGNC Symbol;Acc:18738]	RHOBTB1	9886
ENSG00000129595	2.375505504	4.88145414	2.312087425	3.763854221	-2.438581006	-1.286041897	0.003253211	21	erythrocyte membrane protein band 4.1 like 4A [Source:HGNC Symbol;Acc:13278]	EPB41L4A	64097
ENSG00000047597	4.906810632	2.281276076	5.014084091	2.453576959	2.45022147	1.292912157	0.004075327	22	X-linked Kx blood group (McLeod syndrome) [Source:HGNC Symbol;Acc:12811]	XK	7504
ENSG00000003436	5.992901488	3.08180999	4.845158219	3.32956529	2.50220022	1.323197235	0.005491335	23	tissue factor pathway inhibitor (lipoprotein-associated coagulation inhibitor) [Source:HGNC Symbol;Acc:11760]	TFPI	7035
ENSG00000080493	3.385031692	6.887879069	2.258549343	4.077455267	-2.224283528	-1.153340699	0.001441668	24	solute carrier family 4 (sodium bicarbonate cotransporter), member 4 [Source:HGNC Symbol;Acc:11030]	SLC4A4	8671
ENSG00000204301	1.211923383	2.130260515	1.416109517	1.194495467	-2.301839417	-1.20278719	0.003007005	25	notch 4 [Source:HGNC Symbol;Acc:7884]	NOTCH4	4855
ENSG00000109674	4.623284335	1.907060428	3.443048783	1.213780153	2.329929945	1.220286578	0.003246443	26	nei endonuclease VIII-like 3 (E. coli) [Source:HGNC Symbol;Acc:24573]	NEIL3	55247
ENSG00000213782	2.011277543	3.608710757	1.573523653	1.863180526	-2.267711931	-1.181237385	0.00419678	27	DEAD (Asp-Glu-Ala-Asp) box polypeptide 47 [Source:HGNC Symbol;Acc:18682]	DDX47	51202
ENSG00000102010	4.031618173	1.797404611	4.036497444	2.414566388	2.873392786	1.522755219	0.011076352	28	BMX non-receptor tyrosine kinase [Source:HGNC Symbol;Acc:1079]	BMX	660
ENSG00000223609	7.428861593	1.15224078	13.905609	2.007679048	4.128617735	2.045658847	0.013157969	29	hemoglobin, delta [Source:HGNC Symbol;Acc:4829]	HBD	3045
ENSG00000136231	13.56333408	6.985711904	8.212609394	4.334444209	2.075993726	1.053802084	0.002159855	30	insulin-like growth factor 2 mRNA binding protein 3 [Source:HGNC Symbol;Acc:28868]	IGF2BP3	10643
ENSG00000136425	0.685855354	1.73834526	0.847208947	1.79313767	-2.448715691	-1.292025279	0.01029601	31	calcium and integrin binding family member 2 [Source:HGNC Symbol;Acc:24579]	CIB2	10518
ENSG00000167747	1.838781145	3.003465568	2.191799293	1.679098496	-2.261670949	-1.177389047	0.007622248	32	chromosome 19 open reading frame 48 [Source:HGNC Symbol;Acc:29667]	C19orf48	692204
ENSG00000172159	25.73955772	15.05128321	11.76907038	9.182772504	1.995384017	0.996666424	0.000288008	33	FERM domain containing 3 [Source:HGNC Symbol;Acc:24125]	FRMD3	257019
ENSG00000167633	2.186268793	4.07125112	3.035313619	3.028251964	-2.374800178	-1.247806127	0.010369541	34	killer cell immunoglobulin-like receptor, three domains, long cytoplasmic tail, 1 [Source:HGNC Symbol;Acc:6338]	KIR3DL1	3812
ENSG00000215883	1.281380803	2.507361126	1.29902482	1.739110282	-2.254685313	-1.17292609	0.007942092	35	cytochrome b5 reductase-like [Source:HGNC Symbol;Acc:32220]	CYB5RL	606495

ENSG00000130147	0.914888854	1.476606789	1.57165595	1.079134302	-2.373430323	-1.246973697	0.012324044	36	SH3-domain binding protein 4 [Source:HGNC Symbol;Acc:10826]	SH3BP4	23677
ENSG00000137098	1.811159114	3.188956563	1.55125024	1.962007683	-2.200666104	-1.137940269	0.009091642	37	sperm associated antigen 8 [Source:HGNC Symbol;Acc:14105]	SPAG8	26206
ENSG00000123095	2.272448126	3.237312653	2.735510851	2.682165177	-2.293802825	-1.197741383	0.011869156	38	basic helix-loop-helix family, member e41 [Source:HGNC Symbol;Acc:16617]	BHLHE41	79365
ENSG00000204311	0.806085252	1.431714962	1.273419159	1.345727971	-2.199467494	-1.13715428	0.009934205	39	deafness, autosomal recessive 59 [Source:HGNC Symbol;Acc:29502]	DFNB59	494513
ENSG00000153162	3.032628836	1.40344136	2.601885134	1.21737635	2.273151448	1.184693806	0.012386722	40	bone morphogenetic protein 6 [Source:HGNC Symbol;Acc:1073]	BMP6	654
ENSG00000148346	23.7377637	11.3143912	30.07009985	10.7481985	2.162114626	1.112443011	0.009880678	41	lipocalin 2 [Source:HGNC Symbol;Acc:6526]	LCN2	3934
ENSG00000091129	0.85556892	2.201277689	1.113803191	2.336674714	-2.401122499	-1.263709008	0.014184794	42	neuronal cell adhesion molecule [Source:HGNC Symbol;Acc:7994]	NRCAM	4897
ENSG00000225614	1.1797002	2.547583777	1.066925539	1.972483828	-2.06902648	-1.048952109	0.006613937	43	zinc finger protein 469 [Source:HGNC Symbol;Acc:23216]	ZNF469	84627
ENSG00000066923	1.118090169	2.091083308	0.945193439	1.285340213	-2.080268604	-1.056769821	0.007988204	44	stromal antigen 3 [Source:HGNC Symbol;Acc:11356]	STAG3	10734
ENSG00000147650	5.349330375	3.216511415	2.686651345	2.114899702	2.014392423	1.010344762	0.004497817	45	low density lipoprotein receptor-related protein 12 [Source:HGNC Symbol;Acc:31708]	LRP12	29967
ENSG00000143416	2.015910241	0.597872787	2.855675124	1.021500529	2.605575321	1.38160196	0.016394347	46	selenium binding protein 1 [Source:HGNC Symbol;Acc:10719]	SELENBP1	8991
ENSG00000134827	11.23115274	4.499569381	12.41858672	3.351896796	2.079241178	1.056057111	0.010013012	47	transcobalamin I (vitamin B12 binding protein, R binder family) [Source:HGNC Symbol;Acc:11652]	TCN1	6947
ENSG00000204165	3.231838513	5.592305289	2.211660667	2.83564971	-1.974730777	-0.981655978	0.003699685	48	chromosome X open reading frame 65 [Source:HGNC Symbol;Acc:33713]	CXorf65	158830
ENSG00000184305	3.747313153	5.556954117	3.139176613	3.017616091	-2.040239404	-1.028738449	0.007623696	49	coiled-coil serine-rich protein 1 [Source:HGNC Symbol;Acc:29349]	CCSER1	401145
ENSG00000114405	2.009352141	1.206492789	1.567628386	1.316485723	2.210402556	1.144309135	0.013301876	50	chromosome 3 open reading frame 14 [Source:HGNC Symbol;Acc:25024]	C3orf14	57415
ENSG00000198934	4.381023916	7.439029732	2.514551733	3.656885025	-1.96853371	-0.977121418	0.004472567	51	melanoma antigen family E, 1 [Source:HGNC Symbol;Acc:24934]	MAGEE1	57692
ENSG00000141736	4.430864515	8.283932345	3.495682622	5.036235979	-1.961419936	-0.971898447	0.004863819	52	v-erb-b2 avian erythroblastic leukemia viral oncogene homolog 2 [Source:HGNC Symbol;Acc:3430]	ERBB2	2064
ENSG00000132704	7.029303767	10.14048117	5.796707404	4.543305045	-2.195645198	-1.134644943	0.014388263	53	Fc receptor-like 2 [Source:HGNC Symbol;Acc:14875]	FCRL2	79368

ENSG00000107317	8.083966581	16.56912134	7.577860066	11.11082479	-2.036465953	-1.026067694	0.010307593	54	prostaglandin D2 synthase 21kDa (brain) [Source:HGNC Symbol;Acc:9592]	PTGDS	5730
ENSG00000125459	3.412862019	6.336984983	2.261877477	3.043931969	-1.931992549	-0.950089531	0.004542401	55	misato 1, mitochondrial distribution and morphology regulator [Source:HGNC Symbol;Acc:29678]	MSTO1	55154
ENSG00000183762	13.61113366	6.126659596	13.93662312	7.161621549	2.228989695	1.156389947	0.01625565	56	kringle containing transmembrane protein 1 [Source:HGNC Symbol;Acc:17550]	KREMEN1	83999
ENSG00000134461	2.526511983	3.953361435	2.114509312	1.891792274	-1.982098922	-0.987028966	0.009664815	57	ankyrin repeat domain 16 [Source:HGNC Symbol;Acc:23471]	ANKRD16	54522
ENSG00000189127	3.810561768	1.859650488	2.703359648	1.777696758	2.248063675	1.1686829	0.016574934	58	ankyrin repeat domain 34B [Source:HGNC Symbol;Acc:33736]	ANKRD34B	340120
ENSG00000174482	0.814443457	1.843529768	1.169428573	1.76815522	-2.294124974	-1.197943985	0.017725532	59	leucine rich repeat and Ig domain containing 2 [Source:HGNC Symbol;Acc:21207]	LINGO2	158038
ENSG00000110104	4.1313815	7.068796463	3.143293634	3.024158188	-1.900814806	-0.926617979	0.003079069	60	coiled-coil domain containing 86 [Source:HGNC Symbol;Acc:28359]	CCDC86	79080
ENSG00000183092	2.068391111	1.127006434	2.013630567	1.515494087	2.355666411	1.236135252	0.019836803	61	brain-enriched guanylate kinase-associated [Source:HGNC Symbol;Acc:24163]	BEGAIN	57596
ENSG00000175544	2.310576397	4.008870613	2.203807748	2.346306365	-2.129003423	-1.090178269	0.015618451	62	calcium binding protein 4 [Source:HGNC Symbol;Acc:1386]	CABP4	57010
ENSG00000196092	26.10169365	34.6376584	19.10317749	13.54193022	-2.377534238	-1.249466117	0.021466917	63	paired box 5 [Source:HGNC Symbol;Acc:8619]	PAX5	5079
ENSG00000213077	2.91386422	1.689494513	2.739177392	3.450423371	2.610122781	1.384117673	0.024651016	64	family with sequence similarity 106, member A [Source:HGNC Symbol;Acc:25682]	FAM106A	
ENSG00000149131	17.3154892	6.323972879	36.36520058	7.064779135	2.335683498	1.223844792	0.020774347	65	serpin peptidase inhibitor, clade G (C1 inhibitor), member 1 [Source:HGNC Symbol;Acc:1228]	SERPING1	710
ENSG00000103740	1.751846707	0.659288734	1.603819219	0.744052139	2.298156868	1.200477277	0.020427491	66	acyl-CoA synthetase bubblegum family member 1 [Source:HGNC Symbol;Acc:29567]	ACSBG1	23205
ENSG00000187808	9.39270328	6.225060207	2.896982015	4.311649416	1.848365348	0.886249948	0.000498723	67	sosondowah ankyrin repeat domain family member D [Source:HGNC Symbol;Acc:32960]	SOWAHD	347454
ENSG00000119782	3.283528783	2.003335711	2.327759778	1.598524549	1.991610722	0.993935687	0.013096556	68	FK506 binding protein 1B, 12.6 kDa [Source:HGNC Symbol;Acc:3712]	FKBP1B	2281
ENSG00000163462	0.995989366	1.704502165	1.313326943	1.390866237	-2.205929226	-1.141386505	0.019685567	69	tripartite motif containing 46 [Source:HGNC Symbol;Acc:19019]	TRIM46	80128
ENSG00000082196	1.181405025	2.033608599	0.988592285	1.218019159	-2.070852771	-1.050224988	0.015708353	70	C1q and tumor necrosis factor related protein 3 [Source:HGNC Symbol;Acc:14326]	C1QTNF3	114899

ENSG00000159496	9.348656914	5.717610363	5.190844356	3.633880368	1.907657575	0.93180223	0.007867352	71	ral guanine nucleotide dissociation stimulator-like 4 [Source:HGNC Symbol;Acc:31911]	RGL4	266747
ENSG00000165626	4.099427195	2.307072389	3.616599817	2.157578067	2.170075165	1.117745014	0.019589922	72	BEN domain containing 7 [Source:HGNC Symbol;Acc:23514]	BEND7	222389
ENSG00000109684	1.625330432	2.917866442	1.191671287	1.753847875	-1.950667292	-0.963967732	0.011503313	73	cytokine-dependent hematopoietic cell linker [Source:HGNC Symbol;Acc:17438]	CLNK	116449
ENSG00000169704	13.89780065	7.728397775	11.64675536	6.099334152	2.021757471	1.015609943	0.015075463	74	glycoprotein IX (platelet) [Source:HGNC Symbol;Acc:4444]	GP9	2815
ENSG00000137124	1.959610395	3.74100496	1.329675868	2.344128604	-1.913640416	-0.936319765	0.011045121	75	aldehyde dehydrogenase 1 family, member B1 [Source:HGNC Symbol;Acc:407]	ALDH1B1	219
ENSG00000128512	12.93459367	7.106042195	8.04102327	5.578029597	1.952582491	0.9653835	0.01317343	76	dedicator of cytokinesis 4 [Source:HGNC Symbol;Acc:19192]	DOCK4	9732
ENSG00000140022	6.917157194	4.01219886	3.177107476	2.209200823	1.839038806	0.878951923	0.003255787	77	stonin 2 [Source:HGNC Symbol;Acc:30652]	STON2	85439
ENSG00000166428	2.178995968	4.115025859	1.87988304	2.87388279	-1.977199615	-0.98345853	0.015540318	78	phospholipase D family, member 4 [Source:HGNC Symbol;Acc:23792]	PLD4	122618
ENSG00000173210	11.19022983	5.849426182	10.14559871	4.415626888	1.971397343	0.979218587	0.01552222	79	actin binding LIM protein family, member 3 [Source:HGNC Symbol;Acc:29132]	ABLIM3	22885
ENSG00000137757	23.36665299	10.65575188	18.77829098	7.413421448	1.955139182	0.967271313	0.0150373	80	caspase 5, apoptosis-related cysteine peptidase [Source:HGNC Symbol;Acc:1506]	CASP5	838
ENSG00000156738	241.4870655	287.8482272	165.0155405	85.96041999	-2.64657303	-1.404125465	0.034418953	81	membrane-spanning 4-domains, subfamily A, member 1 [Source:HGNC Symbol;Acc:7315]	MS4A1	931
ENSG00000138002	1.923557533	3.041534112	1.584597029	1.459984054	-1.909973127	-0.93355234	0.012970245	82	intraflagellar transport 172 homolog (Chlamydomonas) [Source:HGNC Symbol;Acc:30391]	IFT172	26160
ENSG00000243452	1.603726393	2.633982867	2.094318126	1.687245967	-2.024043031	-1.017239962	0.018721784	83	neuroblastoma breakpoint family, member 15 [Source:HGNC Symbol;Acc:28791]	NBPF15	284565
ENSG00000198590	1.728372365	2.974637161	1.362829743	1.384763232	-1.917734363	-0.939402898	0.013361893	84	chromosome 3 open reading frame 35 [Source:HGNC Symbol;Acc:24082]	C3orf35	339883
ENSG00000112299	16.11235355	10.72492815	8.84053553	8.919620734	1.879555334	0.910391389	0.010152666	85	vanin 1 [Source:HGNC Symbol;Acc:12705]	VNN1	8876
ENSG00000088726	13.08999555	6.677679135	10.7920425	4.577670004	1.912591148	0.935528504	0.013597218	86	transmembrane protein 40 [Source:HGNC Symbol;Acc:25620]	TMEM40	55287
ENSG00000128536	1.366717495	2.391469008	1.443938183	1.592286781	-2.125143958	-1.087560573	0.024660572	87	cadherin-related family member 3 [Source:HGNC Symbol;Acc:26308]	CDHR3	222256
ENSG00000120217	7.80960311	4.467352296	6.568825439	4.500361297	2.066175347	1.046962694	0.022135233	88	CD274 molecule [Source:HGNC Symbol;Acc:17635]	CD274	29126

ENSG00000180190	2.018975802	1.180589549	1.99016865	1.307068723	2.04299738	1.030687354	0.023921753	89	testis development related protein [Source:HGNC Symbol;Acc:26951]	TDRP	157695
ENSG00000005961	50.33563095	27.68898346	42.10437721	17.67942065	1.901494635	0.927133868	0.015121221	90	integrin, alpha 2b (platelet glycoprotein IIb of IIb/IIIa complex, antigen CD41) [Source:HGNC Symbol;Acc:6138]	ITGA2B	3674
ENSG00000267673	0.908540104	1.676821776	1.021565787	1.365100784	-2.07326343	-1.051903438	0.025253525	91	ferredoxin 1-like [Source:HGNC Symbol;Acc:30546]	FDX1L	112812
ENSG00000177324	12.74323872	7.248469412	9.667275882	5.516799863	2.050423122	1.035921652	0.024866856	92	BEN domain containing 2 [Source:HGNC Symbol;Acc:28509]	BEND2	139105
ENSG00000070371	2.610453365	1.573802469	1.364191347	1.239618979	1.984810095	0.989000978	0.021666146	93	clathrin, heavy chain-like 1 [Source:HGNC Symbol;Acc:2093]	CLTCL1	8218
ENSG00000243772	1.446197913	2.440036888	1.989192998	2.178394656	-2.09781512	-1.068887539	0.030124274	94	killer cell immunoglobulin-like receptor, two domains, long cytoplasmic tail, 3 [Source:HGNC Symbol;Acc:6331]	KIR2DL3	100653050
ENSG00000259207	22.02304554	12.46417787	18.08191931	9.137257676	1.916022304	0.938114356	0.016694453	95	integrin, beta 3 (platelet glycoprotein IIIa, antigen CD61) [Source:HGNC Symbol;Acc:6156]	ITGB3	3690
ENSG00000184792	2.927487273	1.031943816	2.716354587	1.123161504	2.333375158	1.222418281	0.035956853	96	oxysterol binding protein 2 [Source:HGNC Symbol;Acc:8504]	OSBP2	23762
ENSG00000163687	1.227264034	1.923755956	1.459575425	1.663075575	-2.096618375	-1.068064287	0.030626091	97	deoxyribonuclease I-like 3 [Source:HGNC Symbol;Acc:2959]	DNASE1L3	1776
ENSG00000100336	1.396711628	0.526456228	1.496998024	0.617037256	2.176407994	1.121949032	0.032181433	98	apolipoprotein L, 4 [Source:HGNC Symbol;Acc:14867]	APOL4	80832
ENSG00000143297	22.12763771	30.51023866	17.25200693	23.58509291	-2.206850166	-1.141988681	0.034389018	99	Fc receptor-like 5 [Source:HGNC Symbol;Acc:18508]	FCRL5	83416
ENSG00000105559	0.838050832	1.584179955	0.931845393	1.222601833	-2.074197412	-1.052553209	0.029916897	100	pleckstrin homology domain containing, family A (phosphoinositide binding specific) member 4 [Source:HGNC Symbol;Acc:14339]	PLEKHA4	57664
ENSG00000171766	1.484595844	2.473756281	1.366630063	1.587892001	-1.994002201	-0.995667002	0.024498291	101	glycine amidinotransferase (L-arginine:glycine amidinotransferase) [Source:HGNC Symbol;Acc:4175]	GATM	2628
ENSG00000112796	2.330696643	3.421812319	2.189585157	2.596159077	-1.999122947	-0.999367201	0.025349623	102	ectonucleotide pyrophosphatase/phosphodiesterase 5 (putative) [Source:HGNC Symbol;Acc:13717]	ENPP5	59084
ENSG00000133328	1.153633793	2.153588135	2.467947542	3.297670883	-2.337033453	-1.224678385	0.039227472	103	HRAS-like suppressor 2 [Source:HGNC Symbol;Acc:17824]	HRASLS2	54979
ENSG00000181027	3.05502236	4.919319218	1.854152141	2.524282036	-1.818386927	-0.862659218	0.012799205	104	fukutin related protein [Source:HGNC Symbol;Acc:17997]	FKRP	79147
ENSG00000142178	2.762648418	5.557453687	2.436595414	3.00779115	-2.089035702	-1.062837148	0.034725717	105	salt-inducible kinase 1 [Source:HGNC Symbol;Acc:11142]	SIK1	150094

ENSG00000086506	1.765327409	0.705592905	1.617904114	0.887468804	2.14478145	1.100830647	0.0367775	106	hemoglobin, theta 1 [Source:HGNC Symbol;Acc:4833]	HBQ1	3049
ENSG00000198003	2.368767859	1.382211289	1.947613386	1.610625071	2.073817101	1.052288662	0.036072537	107	coiled-coil domain containing 151 [Source:HGNC Symbol;Acc:28303]	CCDC151	115948
ENSG00000114374	6.606615684	36.98956722	19.46245562	59.98413076	-4.14965483	-2.052991337	0.047468348	108	ubiquitin specific peptidase 9, Y-linked [Source:HGNC Symbol;Acc:12633]	USP9Y	8287
ENSG00000121807	73.00684363	45.5065344	35.16300381	20.41282613	1.746024315	0.80407365	0.006398412	109	chemokine (C-C motif) receptor 2 [Source:HGNC Symbol;Acc:1603]	CCR2	729230
ENSG00000049246	3.961725571	7.212720345	2.105037079	3.151196614	-1.74390078	-0.80231796	0.005884936	110	period circadian clock 3 [Source:HGNC Symbol;Acc:8847]	PER3	8863
ENSG00000170456	6.306366599	8.739362796	4.946734931	3.980998762	-1.946106229	-0.960590463	0.029596588	111	DENN/MADD domain containing 5B [Source:HGNC Symbol;Acc:28338]	DENND5B	160518
ENSG00000007402	2.337380564	3.961398173	1.983706006	2.337978111	-1.863961133	-0.898371777	0.021626949	112	calcium channel, voltage-dependent, alpha 2/delta subunit 2 [Source:HGNC Symbol;Acc:1400]	CACNA2D2	9254
ENSG00000183960	0.841357013	1.941362846	1.132561264	2.063190063	-2.177218461	-1.122486174	0.041377672	113	potassium voltage-gated channel, subfamily H (eag-related), member 8 [Source:HGNC Symbol;Acc:18864]	KCNH8	131096
ENSG00000137807	1.975779446	1.363350542	1.004333607	1.141296304	1.878126104	0.909293934	0.023653803	114	kinesin family member 23 [Source:HGNC Symbol;Acc:6392]	KIF23	9493
ENSG00000088826	12.06287679	7.028925619	7.332543851	4.15511349	1.828404061	0.870584928	0.019024462	115	spermine oxidase [Source:HGNC Symbol;Acc:15862]	SMOX	54498
ENSG00000118113	4.736710684	1.164129837	8.107316241	1.353842133	2.392989757	1.258814221	0.045700749	116	matrix metallopeptidase 8 (neutrophil collagenase) [Source:HGNC Symbol;Acc:7175]	MMP8	4317
ENSG00000239961	7.296396007	11.61771434	5.157566155	4.664279164	-1.807858308	-0.85428161	0.017271039	117	leukocyte immunoglobulin-like receptor, subfamily A (with TM domain), member 4 [Source:HGNC Symbol;Acc:15503]	LILRA4	23547
ENSG00000133069	5.220706637	2.70994894	4.780159022	1.799786889	1.899780674	0.925832872	0.026433202	118	transmembrane and coiled-coil domain family 2 [Source:HGNC Symbol;Acc:24239]	TMCC2	9911
ENSG00000122420	2.933362749	1.20293339	3.380805928	1.919039597	2.271167688	1.183434229	0.044199168	119	prostaglandin F receptor (FP) [Source:HGNC Symbol;Acc:9600]	PTGFR	5737
ENSG00000080007	1.150387682	1.898972022	1.3731291	1.642303475	-2.031957306	-1.02287009	0.038248446	120	DEAD (Asp-Glu-Ala-Asp) box polypeptide 43 [Source:HGNC Symbol;Acc:18677]	DDX43	55510
ENSG00000167100	2.443910573	1.076311567	2.154848549	1.039152404	2.03993134	1.028520595	0.038973824	121	sterile alpha motif domain containing 14 [Source:HGNC Symbol;Acc:27312]	SAMD14	201191
ENSG00000126861	3.033068707	1.80764256	2.426037963	2.317240001	2.065480662	1.046477553	0.040167506	122	oligodendrocyte myelin glycoprotein [Source:HGNC Symbol;Acc:8135]	OMG	101927057
ENSG00000204420	72.78674772	39.57062656	61.53006727	25.21896394	1.816689632	0.861311967	0.01985255	123	chromosome 6 open reading frame 25 [Source:HGNC Symbol;Acc:13937]	C6orf25	80739

ENSG00000221909	9.944825771	6.441426169	4.316619651	3.063548934	1.714936659	0.778155292	0.005090491	124	family with sequence similarity 200, member A [Source:HGNC Symbol;Acc:25401]	FAM200A	221786
ENSG00000099256	1.996258938	1.063996967	1.565354831	1.283277082	2.102800602	1.072312053	0.042420511	126	phosphoribosyl transferase domain containing 1 [Source:HGNC Symbol;Acc:23333]	PRTFDC1	56952
ENSG00000113368	85.26330759	52.84436454	37.77045969	21.63507286	1.687155821	0.754593223	0.00215048	127	lamin B1 [Source:HGNC Symbol;Acc:6637]	LMNB1	4001
ENSG00000157985	5.033506213	9.033469608	3.137929912	5.509705576	-1.747088939	-0.804953053	0.013049695	128	ArfGAP with GTPase domain, ankyrin repeat and PH domain 1 [Source:HGNC Symbol;Acc:16922]	AGAP1	116987
ENSG00000146072	1.397215899	2.774929202	1.136726981	1.997050779	-1.896053032	-0.922999316	0.031982847	129	tumor necrosis factor receptor superfamily, member 21 [Source:HGNC Symbol;Acc:13469]	TNFRSF21	27242
ENSG00000150672	1.562190561	2.781733027	1.563364466	1.985228575	-1.925917833	-0.945546154	0.035389045	130	discs, large homolog 2 (Drosophila) [Source:HGNC Symbol;Acc:2901]	DLG2	1740
ENSG00000137198	6.144352171	2.394450542	8.352222499	2.178450334	2.095062821	1.066993504	0.043240544	131	guanosine monophosphate reductase [Source:HGNC Symbol;Acc:4376]	GMPR	2766
ENSG00000130584	1.229848454	1.866692648	1.605359633	1.327342588	-1.900280711	-0.92621255	0.032975429	132	zinc finger and BTB domain containing 46 [Source:HGNC Symbol;Acc:16094]	ZBTB46	140685
ENSG00000184557	26.91281886	16.76509484	19.42719283	17.11431175	1.886825272	0.91596083	0.031252226	133	suppressor of cytokine signaling 3 [Source:HGNC Symbol;Acc:19391]	SOCS3	9021
ENSG00000142173	6.875890191	13.43753212	4.908507498	8.092027487	-1.767091514	-0.821376756	0.015689997	134	collagen, type VI, alpha 2 [Source:HGNC Symbol;Acc:2212]	COL6A2	1292
ENSG00000084731	3.624935862	2.185717018	1.739885832	1.289802468	1.76894831	0.822891892	0.016629226	135	kinesin family member 3C [Source:HGNC Symbol;Acc:6321]	KIF3C	3797
ENSG00000205639	2.858321285	1.277127308	3.015274465	1.258781544	2.046491312	1.033152542	0.042104495	136	major facilitator superfamily domain containing 2B [Source:HGNC Symbol;Acc:37207]	MFSD2B	388931
ENSG00000174080	3.655585808	6.182629222	2.214316661	2.691723182	-1.70444619	-0.769303054	0.006695447	137	cathepsin F [Source:HGNC Symbol;Acc:2531]	CTSF	8722
ENSG00000134755	36.30950108	18.86528313	24.8216779	10.20383944	1.773069341	0.826248958	0.017868601	138	desmocollin 2 [Source:HGNC Symbol;Acc:3036]	DSC2	1824
ENSG00000089692	2.872978661	3.83653893	4.150271548	2.652064677	-1.95745763	-0.96898108	0.040394854	139	lymphocyte-activation gene 3 [Source:HGNC Symbol;Acc:6476]	LAG3	3902
ENSG00000102362	9.116400215	6.011329281	5.74217037	4.445579481	1.834752975	0.875585837	0.028638579	140	synaptotagmin-like 4 [Source:HGNC Symbol;Acc:15588]	SYTL4	94121
ENSG00000135636	51.34301447	30.30422339	42.84671449	23.02453027	1.780459913	0.832249954	0.021803103	141	dysferlin [Source:HGNC Symbol;Acc:3097]	DYSF	8291
ENSG00000162882	1.769496418	1.503863601	1.417284527	2.059714696	1.969847914	0.978084248	0.041493118	142	3-hydroxyanthranilate 3,4-dioxygenase [Source:HGNC Symbol;Acc:4796]	HAAO	23498

ENSG00000143878	44.2316963	30.69638004	23.67800985	23.0296258	1.754410429	0.810986294	0.0173292	143	ras homolog family member B [Source:HGNC Symbol;Acc:668]	RHOB	388
ENSG00000205784	4.724773935	7.490946218	3.474240915	2.898334759	-1.698394724	-0.764171794	0.009599215	144	arrestin domain containing 5 [Source:HGNC Symbol;Acc:31407]	ARRDC5	645432
ENSG00000147536	1.437761368	2.218866385	1.436842308	1.780184165	-1.942808325	-0.958143573	0.040635921	145	GINS complex subunit 4 (Sld5 homolog) [Source:HGNC Symbol;Acc:28226]	GINS4	84296
ENSG00000174469	2.85616464	4.04045338	3.698489914	4.669061486	-1.9618087	-0.972184368	0.042035319	146	contactin associated protein-like 2 [Source:HGNC Symbol;Acc:13830]	CNTNAP2	26047
ENSG00000147180	3.959723058	6.04596997	2.723850466	3.106056261	-1.734308179	-0.794360282	0.016385081	147	zinc finger protein 711 [Source:HGNC Symbol;Acc:13128]	ZNF711	7552
ENSG00000258839	2.206206963	3.06013124	2.631381485	2.163417864	-1.978767684	-0.984602244	0.043798037	148	melanocortin 1 receptor (alpha melanocyte stimulating hormone receptor) [Source:HGNC Symbol;Acc:6929]	MC1R	4157
ENSG00000145685	33.33868339	19.71024031	19.38327526	8.298731281	1.674257566	0.743521488	0.004850616	150	lipoma HMGIC fusion partner-like 2 [Source:HGNC Symbol;Acc:6588]	LHFPL2	10184
ENSG00000123405	91.57913214	56.93311906	45.50373791	30.06845679	1.681010244	0.749328516	0.00672042	151	nuclear factor, erythroid 2 [Source:HGNC Symbol;Acc:7780]	NFE2	4778
ENSG00000105048	5.268346785	3.170743092	4.997059576	3.016895299	1.850370786	0.887814394	0.035922135	152	troponin T type 1 (skeletal, slow) [Source:HGNC Symbol;Acc:11948]	TNNT1	7138
ENSG00000161911	20.95434859	12.06729495	17.34356732	8.478081952	1.78710682	0.837625871	0.027893528	153	triggering receptor expressed on myeloid cells-like 1 [Source:HGNC Symbol;Acc:20434]	TREML1	340205
ENSG00000164330	9.989565032	11.60812928	7.990533423	4.992795058	-1.917400381	-0.939151624	0.041670141	154	early B-cell factor 1 [Source:HGNC Symbol;Acc:3126]	EBF1	1879
ENSG00000156050	1.868657024	3.075647908	1.642744971	2.029489224	-1.85793262	-0.893698182	0.037584639	156	family with sequence similarity 161, member B [Source:HGNC Symbol;Acc:19854]	FAM161B	145483
ENSG00000127586	2.53684073	3.687294557	2.501111856	2.224571768	-1.803602323	-0.850881273	0.03283011	157	CTF18, chromosome transmission fidelity factor 18 homolog (S. cerevisiae) [Source:HGNC Symbol;Acc:18435]	CHTF18	63922
ENSG00000157168	11.25918978	6.759400543	6.102259219	3.83404127	1.725364359	0.786901059	0.018377865	159	neuregulin 1 [Source:HGNC Symbol;Acc:7997]	NRG1	3084
ENSG00000120896	3.647568683	5.747372752	2.732972973	2.173006016	-1.718153243	-0.780858717	0.017160432	160	sorbin and SH3 domain containing 3 [Source:HGNC Symbol;Acc:30907]	SORBS3	10174
ENSG00000123700	27.47846875	14.81130016	22.93635488	13.23279363	1.993304509	0.995162121	0.048376247	161	potassium inwardly-rectifying channel, subfamily J, member 2 [Source:HGNC Symbol;Acc:6263]	KCNJ2	3759
ENSG00000119862	21.24786801	11.59486805	15.57875846	5.610945014	1.727872756	0.788996978	0.020473796	163	lectin, galactoside-binding-like [Source:HGNC Symbol;Acc:25012]	LGALSL	29094

ENSG00000185245	16.11410719	9.727905444	12.49666276	7.281721439	1.782630873	0.834007997	0.033988488	164	glycoprotein Ib (platelet), alpha polypeptide [Source:HGNC Symbol;Acc:4439]	GP1BA	2811
ENSG00000184702	53.43376304	30.50340969	41.53192547	19.72139663	1.775994225	0.82862689	0.032018165	165	septin 5 [Source:HGNC Symbol;Acc:9164]	SEPT5	5413
ENSG00000184113	8.973000424	4.969282815	7.989213256	4.415423004	1.910611012	0.934034085	0.044436628	166	claudin 5 [Source:HGNC Symbol;Acc:2047]	CLDN5	7122
ENSG00000132386	2.896035418	4.459977098	1.946223485	2.210205442	-1.728605081	-0.789608307	0.023057396	167	serpin peptidase inhibitor, clade F (alpha-2 antiplasmin, pigment epithelium derived factor), member 1 [Source:HGNC Symbol;Acc:8824]	SERPINF1	5176
ENSG00000128218	6.531941933	8.184369874	5.353939821	3.888698133	-1.869359413	-0.902543976	0.044415854	170	pre-B lymphocyte 3 [Source:HGNC Symbol;Acc:12710]	VPREB3	29802
ENSG00000136869	111.2392865	69.59020966	49.90945131	22.70806614	1.615412049	0.691902205	0.00304267	171	toll-like receptor 4 [Source:HGNC Symbol;Acc:11850]	TLR4	7099
ENSG00000090920	2.848739275	5.016932463	2.784034745	4.025576578	-1.895796158	-0.922803849	0.047814306	172	Fc fragment of IgG binding protein [Source:HGNC Symbol;Acc:13572]	FCGBP	8857
ENSG00000145335	20.16570524	9.393305742	17.81879056	6.24955912	1.846153482	0.884522498	0.043323264	173	synuclein, alpha (non A4 component of amyloid precursor) [Source:HGNC Symbol;Acc:11138]	SNCA	6622
ENSG00000065534	26.13209965	17.70494861	13.17010933	13.4922254	1.719981051	0.782392671	0.025696375	174	myosin light chain kinase [Source:HGNC Symbol;Acc:7590]	MYLK	4638
ENSG00000118690	2.914819535	4.839438469	1.803600413	2.334421125	-1.668932451	-0.738925563	0.014891048	175	armadillo repeat containing 2 [Source:HGNC Symbol;Acc:23045]	ARMC2	84071
ENSG00000181631	176.9534575	116.6223911	71.47436247	54.87044308	1.620226838	0.69619581	0.006638441	176	purinergic receptor P2Y, G-protein coupled, 13 [Source:HGNC Symbol;Acc:4537]	P2RY13	53829
ENSG00000006432	1.632153721	2.519618065	1.546132735	1.739270202	-1.754089	-0.81072195	0.036473534	178	mitogen-activated protein kinase kinase kinase 9 [Source:HGNC Symbol;Acc:6861]	MAP3K9	4293
ENSG00000115289	4.523482439	3.045885498	1.972641168	1.39898421	1.641598267	0.715101113	0.012898046	179	polycomb group ring finger 1 [Source:HGNC Symbol;Acc:17615]	PCGF1	84759
ENSG00000183019	28.8862251	17.91220438	16.65177093	9.10302642	1.632299243	0.706905565	0.010732014	181	chromosome 19 open reading frame 59 [Source:HGNC Symbol;Acc:27291]	C19orf59	199675
ENSG00000165914	5.376300521	3.339214011	3.611122271	2.725783133	1.837653733	0.877864947	0.046239187	182	tetratricopeptide repeat domain 7B [Source:HGNC Symbol;Acc:19858]	TTC7B	145567
ENSG00000162591	2.852533028	5.054363143	2.281405388	3.598879168	-1.740708505	-0.799674632	0.03570404	184	multiple EGF-like-domains 6 [Source:HGNC Symbol;Acc:3232]	MEGF6	1953
ENSG00000064763	13.86288708	9.15212367	6.150491521	4.360344416	1.633283688	0.707775397	0.012843761	185	fatty acyl CoA reductase 2 [Source:HGNC Symbol;Acc:25531]	FAR2	55711

ENSG00000183837	6.512841925	10.80892708	4.3847128	7.084062633	-1.726592084	-0.787927279	0.034247122	186	paraneoplastic Ma antigen 3 [Source:HGNC Symbol;Acc:18742]	PNMA3	29944
ENSG00000091106	79.4127229	53.94051197	31.49767278	23.14761628	1.601442437	0.679371943	0.004519592	188	NLR family, CARD domain containing 4 [Source:HGNC Symbol;Acc:16412]	NLRC4	58484
ENSG00000174428	3.497063699	5.651778025	1.831972827	2.540251713	-1.683563448	-0.751518092	0.024288675	190	GTF2I repeat domain containing 2B [Source:HGNC Symbol;Acc:33125]	GTF2IRD2B	389524
ENSG00000107796	4.91072947	2.897335623	3.655193152	2.009172679	1.775184076	0.827968631	0.042706014	193	actin, alpha 2, smooth muscle, aorta [Source:HGNC Symbol;Acc:130]	ACTA2	59
ENSG00000063241	3.90605029	5.953901559	2.924856655	3.769377602	-1.73147521	-0.792001732	0.037732848	194	isochorismatase domain containing 2 [Source:HGNC Symbol;Acc:26278]	ISOC2	79763
ENSG00000174175	11.16097542	6.2173897	7.48981612	4.279211071	1.743596216	0.802065978	0.03950821	195	selectin P (granule membrane protein 140kDa, antigen CD62) [Source:HGNC Symbol;Acc:10721]	SELP	6403
ENSG00000189233	3.380482244	5.473586795	3.348923122	2.970115955	-1.725678677	-0.787163858	0.037553499	196	nuclear GTPase, germinal center associated [Source:HGNC Symbol;Acc:33550]	NUGGC	389643
ENSG00000174177	1.776698472	3.266714395	1.324631754	1.915208573	-1.743341149	-0.801854913	0.040901603	199	cytosolic thiouridylase subunit 2 homolog (S. pombe) [Source:HGNC Symbol;Acc:28005]	CTU2	348180
ENSG00000255103	4.324170877	7.319624212	3.019823905	3.316789246	-1.603766436	-0.681464051	0.01000806	200	KIAA0754 [Source:HGNC Symbol;Acc:29111]	KIAA0754	643314
ENSG00000134504	1.071358288	1.63554709	1.095473558	1.073115693	-1.753268882	-0.810047265	0.041736443	201	potassium channel tetramerization domain containing 1 [Source:HGNC Symbol;Acc:18249]	KCTD1	284252
ENSG00000135838	29.56031376	19.34282612	14.9441584	8.728716297	1.608504677	0.685720131	0.012526215	202	N-acetylneuraminate pyruvate lyase (dihydrodipicolinate synthase) [Source:HGNC Symbol;Acc:16781]	NPL	80896
ENSG00000007264	13.27442764	22.34544777	7.917276739	11.38805474	-1.616420431	-0.692802492	0.014082063	206	megakaryocyte-associated tyrosine kinase [Source:HGNC Symbol;Acc:6906]	MATK	4145
ENSG00000101162	328.4746602	198.978885	230.6247865	140.0663961	1.689457559	0.756560109	0.03301209	208	tubulin, beta 1 class VI [Source:HGNC Symbol;Acc:16257]	TUBB1	81027
ENSG00000105374	90.72728198	142.7410209	67.61126131	61.08542622	-1.607202038	-0.684551299	0.013106919	209	natural killer cell group 7 sequence [Source:HGNC Symbol;Acc:7830]	NKG7	4818
ENSG00000120885	165.8043038	98.5616073	130.6044996	66.21830457	1.690176797	0.757174164	0.035537299	211	clusterin [Source:HGNC Symbol;Acc:2095]	CLU	1191
ENSG00000179088	2.711870751	3.77247056	1.897029364	1.86407455	-1.715924532	-0.778986103	0.040031545	212	chromosome 12 open reading frame 42 [Source:HGNC Symbol;Acc:24729]	C12orf42	374470

ENSG00000117115	58.49828642	38.13518514	38.89920795	26.95268645	1.669364393	0.739298904	0.029350726	213	peptidyl arginine deiminase, type II [Source:HGNC Symbol;Acc:18341]	PADI2	11240
ENSG00000168209	16.9326304	31.84991366	10.48190035	19.90777361	-1.656853182	-0.728445768	0.027204839	215	DNA-damage-inducible transcript 4 [Source:HGNC Symbol;Acc:24944]	DDIT4	54541
ENSG00000167483	35.77298193	49.32162239	22.5825207	22.33556567	-1.720294319	-0.782655412	0.042410859	216	family with sequence similarity 129, member C [Source:HGNC Symbol;Acc:24130]	FAM129C	199786
ENSG00000185453	4.815788802	8.136451095	2.188252625	3.622596435	-1.618107382	-0.694307353	0.019043732	217	chromosome 19 open reading frame 68 [Source:HGNC Symbol;Acc:34495]	C19orf68	374920
ENSG00000204475	4.539341297	8.087732326	3.039745066	4.912094176	-1.650654759	-0.723038406	0.026284102	218	natural cytotoxicity triggering receptor 3 [Source:HGNC Symbol;Acc:19077]	NCR3	259197
ENSG00000103056	7.812212895	14.32378288	4.81731904	10.14012177	-1.662675634	-0.733506745	0.03083407	220	sphingomyelin phosphodiesterase 3, neutral membrane (neutral sphingomyelinase II) [Source:HGNC Symbol;Acc:14240]	SMPD3	55512
ENSG00000185864	3.353878048	6.09772586	2.581827178	4.403193096	-1.75147186	-0.80856781	0.048053724	221	nuclear pore complex interacting protein family, member B4 [Source:HGNC Symbol;Acc:41985]	NPIPB4	101929910
ENSG00000139187	53.1538433	83.12541712	35.00534776	37.04825718	-1.59429105	-0.672915028	0.014064054	222	killer cell lectin-like receptor subfamily G, member 1 [Source:HGNC Symbol;Acc:6380]	KLRG1	10219
ENSG00000198478	33.23958949	20.31881071	25.93384931	16.06000195	1.702600077	0.767739601	0.040958664	223	SH3 domain binding glutamic acid-rich protein like 2 [Source:HGNC Symbol;Acc:15567]	SH3BGRL2	83699
ENSG00000121858	258.0539065	160.9133809	188.3562427	55.79471712	1.567090596	0.648088586	0.008537051	226	tumor necrosis factor (ligand) superfamily, member 10 [Source:HGNC Symbol;Acc:11925]	TNFSF10	8743
ENSG00000197774	4.772392551	7.481871412	3.167032565	3.022879062	-1.696458507	-0.762526144	0.041177548	227	essential meiotic structure-specific endonuclease subunit 2 [Source:HGNC Symbol;Acc:27289]	EME2	197342
ENSG00000166262	2.739286196	4.790244135	1.587883103	2.490010091	-1.630037992	-0.70490559	0.025169575	228	family with sequence similarity 227, member B [Source:HGNC Symbol;Acc:26543]	FAM227B	196951
ENSG00000163596	3.554969647	5.61373527	2.466572507	2.376312352	-1.617811811	-0.694043798	0.022752114	229	islet cell autoantigen 1,69kDa-like [Source:HGNC Symbol;Acc:14442]	ICA1L	130026
ENSG00000206561	2.500851006	4.14237547	1.729055325	2.38821027	-1.723734153	-0.785537288	0.046566278	230	collagen-like tail subunit (single strand of homotrimer) of asymmetric acetylcholinesterase [Source:HGNC Symbol;Acc:2226]	COLQ	8292
ENSG00000131876	19.84771063	31.21688628	10.525697	15.03125166	-1.603297707	-0.681042336	0.019177824	232	small nuclear ribonucleoprotein polypeptide A' [Source:HGNC Symbol;Acc:11152]	SNRPA1	6627

ENSG00000091490	55.84628021	84.51247301	27.95246805	23.97198245	-1.55485663	-0.636781559	0.005643102	234	sel-1 suppressor of lin-12-like 3 (C. elegans) [Source:HGNC Symbol;Acc:29108]	SEL1L3	23231
ENSG00000127920	111.1745946	64.76501225	87.36528379	48.72344675	1.70160996	0.766900383	0.044226869	238	guanine nucleotide binding protein (G protein), gamma 11 [Source:HGNC Symbol;Acc:4403]	GNG11	2791
ENSG00000162367	34.41908151	21.1753089	24.7645512	15.16212901	1.705350585	0.770068358	0.046524466	240	T-cell acute lymphocytic leukemia 1 [Source:HGNC Symbol;Acc:11556]	TAL1	6886
ENSG00000163737	423.6930851	242.8815225	357.5787345	185.9056466	1.699725559	0.765301825	0.044633707	241	platelet factor 4 [Source:HGNC Symbol;Acc:8861]	PF4	5196
ENSG00000161835	5.462716879	10.77385401	2.919057535	10.14217826	-1.664879382	-0.73541766	0.03841883	242	GRP1 (general receptor for phosphoinositides 1)-associated scaffold protein [Source:HGNC Symbol;Acc:18707]	GRASP	160622
ENSG00000249992	18.15357751	10.00211895	14.17372645	6.558585289	1.695365526	0.761596356	0.046284779	244	transmembrane protein 158 (gene/pseudogene) [Source:HGNC Symbol;Acc:30293]	TMEM158	25907
ENSG00000185697	110.3247289	169.9025534	54.19334097	62.62780426	-1.567045204	-0.648046798	0.014290404	245	v-myb avian myeloblastosis viral oncogene homolog-like 1 [Source:HGNC Symbol;Acc:7547]	MYBL1	4603
ENSG00000157240	4.697100012	2.877816118	3.06489502	2.010634181	1.69892743	0.764624229	0.048748152	248	frizzled family receptor 1 [Source:HGNC Symbol;Acc:4038]	FZD1	8321
ENSG00000173511	6.129340218	9.235870786	3.213974191	3.235072002	-1.607330253	-0.684666385	0.029393616	251	vascular endothelial growth factor B [Source:HGNC Symbol;Acc:12681]	VEGFB	7423
ENSG00000121316	140.866915	96.94933264	55.36471934	37.60631491	1.539934287	0.622868788	0.005620494	252	phospholipase B domain containing 1 [Source:HGNC Symbol;Acc:26215]	PLBD1	79887
ENSG00000150681	174.3369348	122.4378507	77.65100399	71.31162584	1.566582556	0.647620799	0.01552633	253	regulator of G-protein signaling 18 [Source:HGNC Symbol;Acc:14261]	RGS18	64407
ENSG00000019991	11.99350354	7.330997462	6.828773519	2.708398675	1.574359137	0.654764681	0.017447845	254	hepatocyte growth factor (hepapoietin A; scatter factor) [Source:HGNC Symbol;Acc:4893]	HGF	3082
ENSG00000061918	21.09506705	11.69933823	15.86548469	7.610948101	1.668867325	0.738869265	0.042857609	257	guanylate cyclase 1, soluble, beta 3 [Source:HGNC Symbol;Acc:4687]	GUCY1B3	2983
ENSG00000166963	11.75632303	7.225341629	9.368204497	3.909385358	1.638712207	0.712562508	0.039357077	258	microtubule-associated protein 1A [Source:HGNC Symbol;Acc:6835]	MAP1A	4130
ENSG00000110987	16.8882232	23.19912121	10.71710349	8.068995566	-1.640939776	-0.714522291	0.039776828	259	B-cell CLL/lymphoma 7A [Source:HGNC Symbol;Acc:1004]	BCL7A	605
ENSG00000110002	7.069062428	4.958438138	4.532987075	2.451963671	1.606716979	0.684115823	0.030662534	260	von Willebrand factor A domain containing 5A [Source:HGNC Symbol;Acc:6658]	VWA5A	4013
ENSG00000103043	4.238733817	6.768277395	2.287087161	2.897013653	-1.576954305	-0.657140856	0.019943613	261	Vac14 homolog (S. cerevisiae) [Source:HGNC Symbol;Acc:25507]	VAC14	55697
ENSG00000150337	13.4274813	7.346525884	11.33759255	3.22485893	1.637827214	0.711783165	0.040258628	262	Fc fragment of IgG, high affinity Ia, receptor (CD64) [Source:HGNC Symbol;Acc:3613]	FCGR1A	2210

ENSG00000159128	85.64765915	61.75473903	31.20940724	27.9135291	1.540758802	0.623641033	0.011661894	267	interferon gamma receptor 2 (interferon gamma transducer 1) [Source:HGNC Symbol;Acc:5440]	IFNGR2	3460
ENSG00000144218	24.81187899	38.29422093	12.67810519	15.45649587	-1.59512414	-0.673668706	0.032435616	271	AF4/FMR2 family, member 3 [Source:HGNC Symbol;Acc:6473]	AFF3	3899
ENSG00000115523	273.9247854	486.6932318	154.9593066	353.7151552	-1.583393905	-0.663020203	0.027879784	272	granulysin [Source:HGNC Symbol;Acc:4414]	GNLY	10578
ENSG00000271043	93.09527083	66.96356775	33.39712512	30.40879076	1.542049042	0.624848648	0.014749508	281	MT-RNR2-like 2 [Source:HGNC Symbol;Acc:37156]	MTRNR2L2	100462981
ENSG00000134278	6.694075021	4.487204456	3.52526822	2.602856959	1.610911926	0.68787762	0.039359207	283	spire-type actin nucleation factor 1 [Source:HGNC Symbol;Acc:30622]	SPIRE1	56907
ENSG00000161920	10.92939224	7.680291279	4.883234991	3.182253688	1.540180872	0.623099784	0.014485007	284	mediator complex subunit 11 [Source:HGNC Symbol;Acc:32687]	MED11	400569
ENSG00000115828	42.05696786	27.166234	28.40926916	15.45161452	1.597760001	0.676050718	0.037841324	288	glutaminyl-peptide cyclotransferase [Source:HGNC Symbol;Acc:9753]	QPCT	25797
ENSG00000176473	6.119015581	4.268305644	3.215614656	2.342796886	1.6125477	0.689341836	0.04409594	298	WD repeat domain 25 [Source:HGNC Symbol;Acc:21064]	WDR25	79446
ENSG00000163220	5666.814737	3512.591224	3485.557356	1603.818859	1.565867021	0.646961699	0.031588687	302	S100 calcium binding protein A9 [Source:HGNC Symbol;Acc:10499]	S100A9	6280
ENSG00000111644	26.75027537	16.105678	20.16604496	9.34577934	1.626723383	0.701968948	0.048650445	303	acrosin binding protein [Source:HGNC Symbol;Acc:17195]	ACRBP	84519
ENSG00000160408	3.694325224	5.700262133	2.015372355	2.282942354	-1.576100807	-0.656359812	0.03569431	305	ST6 (alpha-N-acetyl-neuraminyl-2,3-beta-galactosyl-1,3)-N-acetylgalactosaminide alpha-2,6-sialyltransferase 6 [Source:HGNC Symbol;Acc:23364]	ST6GALNAC6	30815
ENSG00000165804	3.180181469	4.793031374	1.938313449	2.071574201	-1.567258178	-0.648242858	0.034323823	308	zinc finger protein 219 [Source:HGNC Symbol;Acc:13011]	ZNF219	51222
ENSG00000171793	5.925948117	9.505775135	2.925769011	3.393424787	-1.539482587	-0.62244555	0.020054219	309	CTP synthase 1 [Source:HGNC Symbol;Acc:2519]	CTPS1	1503
ENSG00000244482	48.1579115	31.84444459	20.86447834	13.04973	1.540699626	0.623585622	0.021879123	312	leukocyte immunoglobulin-like receptor, subfamily A (with TM domain), member 6 [Source:HGNC Symbol;Acc:15495]	LILRA6	
ENSG00000156475	11.33483707	18.16568296	6.573267847	8.37189699	-1.53477408	-0.618026305	0.019667708	315	protein phosphatase 2, regulatory subunit B, beta [Source:HGNC Symbol;Acc:9305]	PPP2R2B	5521
ENSG00000172543	33.00780241	54.01056394	18.24336494	28.09153507	-1.545205553	-0.627798768	0.026093057	316	cathepsin W [Source:HGNC Symbol;Acc:2546]	CTSW	1521
ENSG00000072952	43.4274048	28.47200947	25.91872347	21.74079208	1.601138839	0.679098413	0.044619245	317	murine retrovirus integration site 1 homolog [Source:HGNC Symbol;Acc:7237]	MRVI1	10335
ENSG00000187554	30.58700789	20.41186558	12.58617977	6.069820887	1.504781617	0.58955413	0.008748618	318	toll-like receptor 5 [Source:HGNC Symbol;Acc:11851]	TLR5	7100

ENSG00000174125	110.5989686	74.438468	56.30889154	35.57865716	1.537337999	0.620434391	0.024858712	322	toll-like receptor 1 [Source:HGNC Symbol;Acc:11847]	TLR1	7096
ENSG00000051128	36.60924639	25.190717	17.70040006	9.516803043	1.517364037	0.60156725	0.016614249	325	homer homolog 3 (Drosophila) [Source:HGNC Symbol;Acc:17514]	HOMER3	9454
ENSG00000054793	10.92687059	8.435043882	5.409681683	6.106558515	1.597339308	0.675670804	0.049888158	328	ATPase, class II, type 9A [Source:HGNC Symbol;Acc:13540]	ATP9A	10079
ENSG00000204252	17.55739892	26.05946797	8.74753859	9.196634738	-1.531974833	-0.615392597	0.025344163	333	major histocompatibility complex, class II, DO alpha [Source:HGNC Symbol;Acc:4936]	HLA-DOA	3111
ENSG00000155906	2.324998811	3.273544628	1.644634697	1.255888795	-1.544429285	-0.627073815	0.03690621	338	required for meiotic nuclear division 1 homolog (S. cerevisiae) [Source:HGNC Symbol;Acc:21176]	RMND1	55005
ENSG00000130150	46.37834855	32.88539247	21.03426744	14.54415403	1.512046411	0.596502423	0.021264066	343	motile sperm domain containing 2 [Source:HGNC Symbol;Acc:28381]	MOSPD2	158747
ENSG00000034053	24.49679162	36.30231515	11.01389478	11.10671005	-1.512745932	-0.597169705	0.021846294	346	amyloid beta (A4) precursor protein-binding, family A, member 2 [Source:HGNC Symbol;Acc:579]	APBA2	321
ENSG00000185513	2.831358363	4.278245195	1.817476527	1.90018971	-1.546129634	-0.628661286	0.039990106	347	l(3)mbt-like 1 (Drosophila) [Source:HGNC Symbol;Acc:15905]	L3MBTL1	26013
ENSG00000165795	16.25531004	25.4942507	8.150042465	12.21880551	-1.532536164	-0.615921119	0.033667623	348	NDRG family member 2 [Source:HGNC Symbol;Acc:14460]	NDRG2	57447
ENSG00000111817	67.64228398	47.1870614	22.23544359	12.32053952	1.47497511	0.56069061	0.007241331	351	dermatan sulfate epimerase [Source:HGNC Symbol;Acc:21144]	DSE	29940
ENSG00000117877	4.708109363	6.822495684	3.138822247	3.021892384	-1.565581835	-0.646698922	0.047737392	355	CD3e molecule, epsilon associated protein [Source:HGNC Symbol;Acc:24219]	CD3EAP	10849
ENSG00000172716	43.37926096	31.48664668	14.21525223	11.79895477	1.477520037	0.563177696	0.01085435	358	schlafen family member 11 [Source:HGNC Symbol;Acc:26633]	SLFN11	91607
ENSG00000187116	105.4338587	71.30938369	44.71117316	27.95919488	1.508694364	0.59330057	0.028963165	364	leukocyte immunoglobulin-like receptor, subfamily A (with TM domain), member 5 [Source:HGNC Symbol;Acc:16309]	LILRA5	353514
ENSG00000166313	9.01006604	13.50492553	5.525724571	6.935036685	-1.519403799	-0.603505334	0.034849793	369	amyloid beta (A4) precursor protein-binding, family B, member 1 (Fe65) [Source:HGNC Symbol;Acc:581]	APBB1	322
ENSG00000205336	38.25527737	61.32075322	23.40844172	31.74922692	-1.534489983	-0.617759228	0.041776525	374	G protein-coupled receptor 56 [Source:HGNC Symbol;Acc:4512]	GPR56	9289
ENSG00000100385	27.5879452	41.5141861	13.42825183	14.92904987	-1.499759926	-0.58473158	0.025001279	375	interleukin 2 receptor, beta [Source:HGNC Symbol;Acc:6009]	IL2RB	3560
ENSG00000007968	7.724905114	5.492674906	4.217174894	3.054324367	1.532250763	0.615652423	0.041227158	376	E2F transcription factor 2 [Source:HGNC Symbol;Acc:3114]	E2F2	1870

ENSG00000164885	9.323095424	6.611806711	3.830961717	2.505784794	1.496481731	0.581574667	0.023981641	377	cyclin-dependent kinase 5 [Source:HGNC Symbol;Acc:1774]	CDK5	1020
ENSG00000105738	8.762335822	13.37602492	4.550821714	7.020176976	-1.51763202	-0.601822023	0.036703698	383	signal-induced proliferation-associated 1 like 3 [Source:HGNC Symbol;Acc:23801]	SIPA1L3	23094
ENSG00000184205	27.83776142	46.57749616	12.40982331	25.97261914	-1.517014312	-0.601234697	0.037260742	385	TSPY-like 2 [Source:HGNC Symbol;Acc:24358]	TSPYL2	64061
ENSG00000132357	26.10889685	19.31809023	9.338453709	6.948613858	1.464798366	0.550702087	0.015371512	398	caspase recruitment domain family, member 6 [Source:HGNC Symbol;Acc:16394]	CARD6	84674
ENSG00000110318	9.864719463	15.26037288	4.668727274	5.703889363	-1.489644914	-0.574968477	0.034028062	405	KIAA1377 [Source:HGNC Symbol;Acc:29264]	KIAA1377	57562
ENSG00000181274	179.4529929	123.9319522	106.7305695	79.93464894	1.524620052	0.608449756	0.049056377	411	frequently rearranged in advanced T-cell lymphomas 2 [Source:HGNC Symbol;Acc:16048]	FRAT2	23401
ENSG00000151458	22.04189907	15.29349948	10.1998834	6.313836509	1.484736545	0.570206958	0.037478291	420	ankyrin repeat domain 50 [Source:HGNC Symbol;Acc:29223]	ANKRD50	57182
ENSG00000130830	66.79397165	47.54847467	24.27296932	13.56177362	1.448693022	0.53475192	0.019012097	428	membrane protein, palmitoylated 1, 55kDa [Source:HGNC Symbol;Acc:7219]	MPP1	4354
ENSG00000110921	7.119681945	10.07412418	3.607174291	3.473097761	-1.483652803	-0.569153519	0.043339715	435	mevalonate kinase [Source:HGNC Symbol;Acc:7530]	MVK	4598
ENSG00000186407	109.9135729	75.546896	60.72589884	21.15824076	1.449709286	0.535763622	0.024172056	437	CD300e molecule [Source:HGNC Symbol;Acc:28874]	CD300E	342510
ENSG00000156026	11.076805	8.306778641	4.016438732	3.755202429	1.457158985	0.543158293	0.038940504	458	mitochondrial calcium uniporter [Source:HGNC Symbol;Acc:23526]	MCU	90550
ENSG00000149548	5.193267723	7.92033107	2.305865625	3.429019148	-1.471048995	-0.556845298	0.045340465	463	coiled-coil domain containing 15 [Source:HGNC Symbol;Acc:25798]	CCDC15	80071
ENSG00000175471	99.49359132	72.31816689	33.55969782	22.37792498	1.435645898	0.521699952	0.024303968	464	multiple C2 domains, transmembrane 1 [Source:HGNC Symbol;Acc:26183]	MCTP1	79772
ENSG00000184083	6.510506665	9.94641757	3.279813448	4.099623878	-1.458526637	-0.544511735	0.04094021	465	family with sequence similarity 120C [Source:HGNC Symbol;Acc:16949]	FAM120C	54954
ENSG00000152213	38.79126964	27.71861812	16.25478591	12.13898575	1.457818128	0.543810745	0.041288818	468	ADP-ribosylation factor-like 11 [Source:HGNC Symbol;Acc:24046]	ARL11	115761
ENSG00000168461	290.3889325	210.3870255	100.1260858	52.59996364	1.421372448	0.50728464	0.014776332	469	RAB31, member RAS oncogene family [Source:HGNC Symbol;Acc:9771]	RAB31	11031
ENSG00000053372	14.59229467	20.91064661	8.009818306	5.746418832	-1.457429725	-0.54342632	0.043237573	477	mRNA turnover 4 homolog (S. cerevisiae) [Source:HGNC Symbol;Acc:18477]	MRTO4	51154
ENSG00000020633	233.4324919	347.0388739	113.2433183	131.4178547	-1.438100495	-0.524164495	0.033822417	481	runt-related transcription factor 3 [Source:HGNC Symbol;Acc:10473]	RUNX3	864

ENSG00000163694	76.97364233	55.40825304	32.1295607	23.68803849	1.453081144	0.53911527	0.041499279	483	RNA binding motif protein 47 [Source:HGNC Symbol;Acc:30358]	RBM47	54502
ENSG00000159433	8.552303058	12.31508549	3.752031517	3.149216494	-1.46241952	-0.548357233	0.047402942	485	StAR-related lipid transfer (START) domain containing 9 [Source:HGNC Symbol;Acc:19162]	STARD9	57519
ENSG00000183621	30.86881129	22.81127232	9.887970494	7.170237544	1.431735965	0.517765461	0.037278689	500	zinc finger protein 438 [Source:HGNC Symbol;Acc:21029]	ZNF438	220929
ENSG00000100522	7.443962872	10.60810454	3.804945408	3.445336578	-1.442030572	-0.528101751	0.047454807	513	glucosamine-phosphate N-acetyltransferase 1 [Source:HGNC Symbol;Acc:19980]	GNPNAT1	64841
ENSG00000140090	35.32329787	25.91543684	14.42515501	11.44195726	1.439857563	0.525926101	0.045226476	516	solute carrier family 24 (sodium/potassium/calcium exchanger), member 4 [Source:HGNC Symbol;Acc:10978]	SLC24A4	123041
ENSG00000145912	19.77057614	28.75089298	7.935555365	9.251160653	-1.408528697	-0.494188957	0.031961034	544	NHP2 ribonucleoprotein [Source:HGNC Symbol;Acc:14377]	NHP2	55651
ENSG00000138050	9.547319522	13.95045396	3.93221289	5.016585675	-1.429753165	-0.515766099	0.046408501	546	THUMP domain containing 2 [Source:HGNC Symbol;Acc:14890]	THUMPD2	80745
ENSG00000169413	73.09101991	55.32651793	23.6970749	19.1331586	1.404371776	0.489924908	0.040544181	578	ribonuclease, RNase A family, k6 [Source:HGNC Symbol;Acc:10048]	RNASE6	6039
ENSG00000038427	2385.033014	1728.618148	927.4714957	496.2101967	1.405889137	0.491482833	0.044219697	588	versican [Source:HGNC Symbol;Acc:2464]	VCAN	1462
ENSG00000196954	226.0076043	168.0939944	75.05473739	41.89641788	1.391665176	0.476812152	0.036686976	592	caspase 4, apoptosis-related cysteine peptidase [Source:HGNC Symbol;Acc:1505]	CASP4	837
ENSG00000138449	59.3277005	43.06939773	26.1239144	13.39886368	1.406565413	0.492176648	0.046339499	597	solute carrier family 40 (iron-regulated transporter), member 1 [Source:HGNC Symbol;Acc:10909]	SLC40A1	30061
ENSG00000127124	23.82273088	33.85726832	11.40560661	10.51321771	-1.3989526	-0.484347081	0.044443952	612	human immunodeficiency virus type I enhancer binding protein 3 [Source:HGNC Symbol;Acc:13561]	HIVEP3	59269
ENSG00000114450	25.00338647	18.83873752	7.957346257	5.625785206	1.385004271	0.469890425	0.039549633	627	guanine nucleotide binding protein (G protein), beta polypeptide 4 [Source:HGNC Symbol;Acc:20731]	GNB4	59345
ENSG00000143207	41.22710461	31.81808162	11.11623096	10.62131297	1.390193022	0.475285208	0.049342098	646	ring finger and WD repeat domain 2, E3 ubiquitin protein ligase [Source:HGNC Symbol;Acc:17440]	RFWD2	64326
ENSG00000198315	11.99548326	16.91055049	4.568977722	4.706235062	-1.38006701	-0.46473832	0.047603459	676	zinc finger with KRAB and SCAN domains 8 [Source:HGNC Symbol;Acc:12983]	ZKSCAN8	7745

ENSG00000005020	268.7111224	206.6077139	77.86451143	53.50085172	1.370049543	0.454228064	0.041825014	684	src kinase associated phosphoprotein 2 [Source:HGNC Symbol;Acc:15687]	SKAP2	8935
ENSG00000156273	120.6134642	91.56011026	39.20922945	23.17303201	1.367189645	0.451213375	0.047474689	705	BTB and CNC homology 1, basic leucine zipper transcription factor 1 [Source:HGNC Symbol;Acc:935]	BACH1	100379661
ENSG00000126351	30.10942342	42.99403426	8.008425854	10.63916092	-1.362456845	-0.446210534	0.044184059	707	thyroid hormone receptor, alpha [Source:HGNC Symbol;Acc:11796]	THRA	7067
ENSG00000188921	73.75617374	58.4076536	17.21561987	16.92616327	1.354590098	0.437856355	0.038915146	713	protein tyrosine phosphatase-like A domain containing 2 [Source:HGNC Symbol;Acc:20920]	PTPLAD2	401494
ENSG00000135976	83.47980708	117.5002407	24.73696681	26.15215608	-1.353853048	-0.437071152	0.041138468	726	ankyrin repeat domain 36 [Source:HGNC Symbol;Acc:24079]	ANKRD36	375248

Compared populations:
D2T RA vs HC
Fold change describes the size of the difference in gene expression (given in mean and standard deviation, sd) between the compared groups. The values are always in relation to the group used as a base level group (reference). P-value describes the reliability of the change in expression value between the compared groups. Results were considered significant with a fold change of 1.2 and a p-value cutoff of 0.05.

Supplementary Material 18 – Summary of enrichment analysis of DE genes listing the top 25 processes or pathways based on different databases (Gene Ontology and Reactome)

Gene Ontology Molecular Function					
**GO.ID**	**Term**	**Annotated**	**Significant**	**Expected**	**p value**
GO:0004867	serine-type endopeptidase inhibitor acti...	30	3	0.2	0.00101
GO:0098772	molecular function regulator activity	1169	16	7.78	0.00365
GO:0004866	endopeptidase inhibitor activity	62	3	0.41	0.00806
GO:0030414	peptidase inhibitor activity	64	3	0.43	0.00879
GO:0008017	microtubule binding	200	5	1.33	0.0105
GO:0005509	calcium ion binding	287	6	1.91	0.0119
GO:0030234	enzyme regulator activity	875	12	5.82	0.0121
GO:0008092	cytoskeletal protein binding	669	10	4.45	0.0126
GO:0061135	endopeptidase regulator activity	76	3	0.51	0.014
GO:0044325	transmembrane transporter binding	80	3	0.53	0.0161
GO:0005178	integrin binding	82	3	0.55	0.0172
GO:0019887	protein kinase regulator activity	169	4	1.12	0.0259
GO:0061134	peptidase regulator activity	106	3	0.71	0.0334
GO:0015631	tubulin binding	278	5	1.85	0.0374
GO:0019207	kinase regulator activity	194	4	1.29	0.04
GO:0004857	enzyme inhibitor activity	211	4	1.4	0.0517
GO:0005102	signaling receptor binding	736	9	4.9	0.0542
GO:1901981	phosphatidylinositol phosphate binding	136	3	0.9	0.0618
GO:0030546	signaling receptor activator activity	147	3	0.98	0.0744
GO:0019904	protein domain specific binding	471	6	3.13	0.0926
GO:0030545	signaling receptor regulator activity	162	3	1.08	0.0931
GO:0140678	molecular function inhibitor activity	272	4	1.81	0.107
GO:0019901	protein kinase binding	501	6	3.33	0.115
GO:0003779	actin binding	282	4	1.88	0.118
GO:0140097	catalytic activity, acting on DNA	203	3	1.35	0.153

Gene Ontology Cellular Component					
GO.ID	Term	Annotated	Significant	Expected	p value
GO:0043025	neuronal cell body	256	10	1.73	0.0000078
GO:0044297	cell body	293	10	1.98	0.000025
GO:0005576	extracellular region	2061	29	13.93	0.000033
GO:0036477	somatodendritic compartment	443	12	2.99	0.000037
GO:0120025	plasma membrane bounded cell projection	1252	21	8.46	0.000053
GO:0042995	cell projection	1318	21	8.91	0.00011
GO:0043195	terminal bouton	15	3	0.1	0.00013
GO:0043679	axon terminus	42	4	0.28	0.00018
GO:0043005	neuron projection	691	14	4.67	0.00018
GO:0044306	neuron projection terminus	52	4	0.35	0.00041
GO:0045202	synapse	782	14	5.28	0.00065
GO:0150034	distal axon	160	6	1.08	0.00072
GO:0098793	presynapse	296	8	2	0.00083
GO:0071944	cell periphery	2912	31	19.68	0.00293
GO:0030424	axon	365	8	2.47	0.00311
GO:0030054	cell junction	1241	17	8.39	0.00318
GO:0005615	extracellular space	1720	21	11.62	0.0039
GO:0097060	synaptic membrane	156	5	1.05	0.00402
GO:0005886	plasma membrane	2735	29	18.48	0.00466
GO:0005856	cytoskeleton	1563	19	10.56	0.00664
GO:0030425	dendrite	329	7	2.22	0.00664
GO:0097447	dendritic tree	330	7	2.23	0.00675
GO:0099513	polymeric cytoskeletal fiber	430	8	2.91	0.00824
GO:0005923	bicellular tight junction	64	3	0.43	0.00919
GO:0042734	presynaptic membrane	64	3	0.43	0.00919

Reactome				
**Reactome.ID**	**Term**	**GeneRatio**	**BgRatio**	**p value**
R-HSA-983231	Factors involved in megakaryocyte development and platelet production	5/46	107/6910	0.000670642
R-HSA-9013404	RAC2 GTPase cycle	4/46	76/6910	0.001554389
R-HSA-983189	Kinesins	3/46	36/6910	0.001690515
R-HSA-1221632	Meiotic synapsis	3/46	40/6910	0.002296176
R-HSA-9013026	RHOB GTPase cycle	3/46	52/6910	0.004858087
R-HSA-9645723	Diseases of programmed cell death	3/46	53/6910	0.005125774
R-HSA-442755	Activation of NMDA receptors and postsynaptic events	3/46	56/6910	0.005981665
R-HSA-5334118	DNA methylation	2/46	19/6910	0.006898648
R-HSA-1500620	Meiosis	3/46	59/6910	0.006918376
R-HSA-9013106	RHOC GTPase cycle	3/46	59/6910	0.006918376
R-HSA-1251985	Nuclear signaling by ERBB4	2/46	20/6910	0.007632854
R-HSA-389661	Glyoxylate metabolism and glycine degradation	2/46	20/6910	0.007632854
R-HSA-73728	RNA Polymerase I Promoter Opening	2/46	20/6910	0.007632854
R-HSA-171306	Packaging Of Telomere Ends	2/46	21/6910	0.008400767
R-HSA-69473	G2/M DNA damage checkpoint	3/46	64/6910	0.008664099
R-HSA-5625886	Activated PKN1 stimulates transcription of AR (androgen receptor) regulated genes KLK2 and KLK3	2/46	22/6910	0.009201926
R-HSA-5693607	Processing of DNA double-strand break ends	3/46	66/6910	0.009428366
R-HSA-1474165	Reproduction	3/46	67/6910	0.00982485
R-HSA-427359	SIRT1 negatively regulates rRNA expression	2/46	24/6910	0.010902158
R-HSA-6811434	COPI-dependent Golgi-to-ER retrograde traffic	3/46	70/6910	0.011072272
R-HSA-110330	Recognition and association of DNA glycosylase with site containing an affected purine	2/46	25/6910	0.011800331
R-HSA-110331	Cleavage of the damaged purine	2/46	25/6910	0.011800331
R-HSA-68616	Assembly of the ORC complex at the origin of replication	2/46	25/6910	0.011800331
R-HSA-73927	Depurination	2/46	25/6910	0.011800331
R-HSA-9013423	RAC3 GTPase cycle	3/46	74/6910	0.012872319

Compared populations:

D2T RA vs non-D2T RA

Enrichment analysis for the differentially expressed filtered gene lists tests whether the genes within a certain Reactome pathway or GO term are statistically over-represented in a given comparison. Annotated: number of genes associated with the given term. R: Reactome database. Significant: number of statistically significant associated with the given term. Expected: expected number of genes associated with the given term in the DE gene list. Gene ratio: ratio of number of genes in the DE list that overlap with genes associated with the given term, and the number of genes in the DE list which overlap with genes associated with all terms in the Reactome database. Bg ratio: ratio of the number of genes associated with the given term, and the total number of genes associated with Reactome terms.

Supplementary Material 19 - List of differentially expressed (DE) genes

ID	mean_HighInflammation_HighPain	mean_HighInflammation_LowPain	sd_HighInflammation_HighPain	sd_HighInflammation_LowPain	FC	logFC	p value	avgRank	Description	geneName	EntrezID
ENSG00000134321	6.614323944	80.89360183	7.71016597	148.0872497	-7.003004245	-2.807973962	0.001611232	1	radical S-adenosyl methionine domain containing 2 [Source:HGNC Symbol;Acc:30908]	RSAD2	91543
ENSG00000174705	1.194779964	2.791445356	2.04033377	1.617012683	-5.673810231	-2.504317898	0.000838553	2	SH3 and PX domains 2B [Source:HGNC Symbol;Acc:29242]	SH3PXD2B	285590
ENSG00000160360	4.509203423	1.020531155	2.83240686	0.886771848	4.69062297	2.229779543	0.000168395	3	G-protein signaling modulator 1 [Source:HGNC Symbol;Acc:17858]	GPSM1	26086
ENSG00000158106	3.854655382	1.158853822	2.26485127	0.88925053	4.326602487	2.113234577	0.000784005	4	rhophilin, Rho GTPase binding protein 1 [Source:HGNC Symbol;Acc:19973]	RHPN1	114822
ENSG00000112874	2.877579303	1.125370241	1.54431384	1.246402629	4.59524444	2.200141607	0.001377461	5	nudix (nucleoside diphosphate linked moiety X)-type motif 12 [Source:HGNC Symbol;Acc:18826]	NUDT12	83594
ENSG00000088827	2.509886308	45.49724167	2.7358082	84.68829382	-6.307310665	-2.657024994	0.004198781	6	sialic acid binding Ig-like lectin 1, sialoadhesin [Source:HGNC Symbol;Acc:11127]	SIGLEC1	6614
ENSG00000102349	6.358265694	3.010645169	3.3165225	2.779896626	3.935561091	1.976569335	0.002436964	7	Kruppel-like factor 8 [Source:HGNC Symbol;Acc:6351]	KLF8	11279
ENSG00000166582	3.667076238	1.288821001	2.23985951	1.400115275	3.891967899	1.960499811	0.001930892	8	centromere protein V [Source:HGNC Symbol;Acc:29920]	CENPV	201161
ENSG00000188076	3.509747819	0.539596353	3.91793087	0.999139006	6.61783701	2.72635976	0.007492215	9	secretoglobin, family 1C, member 1 [Source:HGNC Symbol;Acc:18394]	SCGB1C1	147199
ENSG00000144792	3.046066905	0.89151425	2.28817984	0.89086156	4.037202799	2.013356059	0.005208221	10	zinc finger protein 660 [Source:HGNC Symbol;Acc:26720]	ZNF660	285349
ENSG00000097096	5.062288451	1.668923839	2.4212943	1.318950581	3.875462274	1.954368409	0.007120947	11	synapse defective 1, Rho GTPase, homolog 2 (C. elegans) [Source:HGNC Symbol;Acc:25841]	SYDE2	84144
ENSG00000173258	4.676829104	1.630912835	2.37691287	1.481201209	3.586801024	1.842697715	0.003595148	12	zinc finger protein 483 [Source:HGNC Symbol;Acc:23384]	ZNF483	158399
ENSG00000105173	0.848721636	2.637907937	0.72432429	1.587756236	-3.76505163	-1.912669649	0.005674206	13	cyclin E1 [Source:HGNC Symbol;Acc:1589]	CCNE1	898
ENSG00000185745	11.02552384	59.65488879	16.7692543	93.20596527	-5.047249071	-2.335497282	0.011495046	14	interferon-induced protein with tetratricopeptide repeats 1 [Source:HGNC Symbol;Acc:5407]	IFIT1	3434
ENSG00000053524	3.339023988	1.177278011	2.17435056	1.102731045	3.658239989	1.871149722	0.004794436	15	MCF.2 cell line derived transforming sequence-like 2 [Source:HGNC Symbol;Acc:30319]	MCF2L2	23101
ENSG00000141696	4.896779098	1.42887882	3.3374035	1.292417239	3.568112874	1.835161254	0.004319667	16	leprecan-like 4 [Source:HGNC Symbol;Acc:16946]	LEPREL4	10609
ENSG00000180071	10.49540529	4.884716645	4.18120424	2.976837603	3.21242155	1.683661223	0.00176424	17	ankyrin repeat domain 18A [Source:HGNC Symbol;Acc:23643]	ANKRD18A	253650
ENSG00000188033	1.55939203	0.481428382	0.92259078	0.714553946	3.769595344	1.914409662	0.012818626	18	zinc finger protein 490 [Source:HGNC Symbol;Acc:23705]	ZNF490	57474
ENSG00000094804	0.498951723	1.749115431	0.65883753	1.204442667	-3.90316316	-1.964643771	0.01696979	19	cell division cycle 6 [Source:HGNC Symbol;Acc:1744]	CDC6	990

ENSG00000132330	0.615138662	2.029781097	0.99541091	1.57210144	-3.486424006	-1.801748035	0.011731958	20	selenocysteine lyase [Source:HGNC Symbol;Acc:18161]	SCLY	51540
ENSG00000101230	2.602559001	0.492917112	2.80449428	0.534745729	3.791365601	1.922717582	0.015936915	21	isthmin 1, angiogenesis inhibitor [Source:HGNC Symbol;Acc:16213]	ISM1	140862
ENSG00000197208	3.663082516	2.845033403	2.06103184	4.480624952	4.210983138	2.074157098	0.020777175	22	solute carrier family 22 (organic cation/zwitterion transporter), member 4 [Source:HGNC Symbol;Acc:10968]	SLC22A4	6583
ENSG00000182636	2.108665829	0.316780842	1.58141238	0.542576339	4.007923876	2.002855107	0.020315291	23	necdin, melanoma antigen (MAGE) family member [Source:HGNC Symbol;Acc:7675]	NDN	4692
ENSG00000184305	3.381995514	2.889747965	2.15512523	4.375544432	3.272714346	1.710487685	0.011133416	24	coiled-coil serine-rich protein 1 [Source:HGNC Symbol;Acc:29349]	CCSER1	401145
ENSG00000117322	6.82490474	4.873139516	2.97363626	4.35612251	3.354984892	1.74630627	0.014005373	25	complement component (3d/Epstein Barr virus) receptor 2 [Source:HGNC Symbol;Acc:2336]	CR2	1380
ENSG00000119917	51.07266858	260.3042976	36.5154197	355.8265621	-3.255098154	-1.702701047	0.012055482	26	interferon-induced protein with tetratricopeptide repeats 3 [Source:HGNC Symbol;Acc:5411]	IFIT3	3437
ENSG00000145632	0.528388374	2.00378284	0.56518113	1.540272652	-3.769166541	-1.914245542	0.021072986	27	polo-like kinase 2 [Source:HGNC Symbol;Acc:19699]	PLK2	10769
ENSG00000198934	5.205393138	2.980140283	2.64984311	2.850538562	2.99408511	1.582115232	0.007482453	28	melanoma antigen family E, 1 [Source:HGNC Symbol;Acc:24934]	MAGEE1	57692
ENSG00000135643	1.844806994	0.546942659	1.30721971	0.632095168	3.341048045	1.740300729	0.018231207	29	potassium large conductance calcium-activated channel, subfamily M, beta member 4 [Source:HGNC Symbol;Acc:6289]	KCNMB4	27345
ENSG00000116151	1.029104235	2.208155867	1.34996532	1.086867908	-3.319588556	-1.731004439	0.017411875	30	MORN repeat containing 1 [Source:HGNC Symbol;Acc:25852]	MORN1	79906
ENSG00000133169	2.373553824	1.183620457	1.92030673	1.773250149	3.331892523	1.736341864	0.018576311	31	brain expressed, X-linked 1 [Source:HGNC Symbol;Acc:1036]	BEX1	55859
ENSG00000119922	66.26633537	279.8924292	46.2466744	376.632263	-2.988867213	-1.579598804	0.011095558	32	interferon-induced protein with tetratricopeptide repeats 2 [Source:HGNC Symbol;Acc:5409]	IFIT2	3433
ENSG00000151023	16.33234649	8.187596964	9.8181111	8.647803596	3.727777182	1.898315629	0.02529914	33	enkurin, TRPC channel interacting protein [Source:HGNC Symbol;Acc:28388]	ENKUR	219670
ENSG00000204882	1.814815662	4.127209664	2.73148751	3.335123049	-3.624008435	-1.857586313	0.025165144	34	G protein-coupled receptor 20 [Source:HGNC Symbol;Acc:4475]	GPR20	2843
ENSG00000153157	0.434664136	2.172733192	0.93088162	2.698124042	-4.268665994	-2.093785282	0.031618143	35	synaptonemal complex protein 2-like [Source:HGNC Symbol;Acc:21537]	SYCP2L	221711
ENSG00000180423	4.503509319	2.343284496	2.87961577	2.0562967	2.817274826	1.494300306	0.008829907	36	harbinger transposase derived 1 [Source:HGNC Symbol;Acc:26522]	HARBI1	283254
ENSG00000176454	5.414769767	2.394752944	3.36388164	1.519438776	2.767943889	1.468814697	0.006840446	37	lysophosphatidylcholine acyltransferase 4 [Source:HGNC Symbol;Acc:30059]	LPCAT4	254531
ENSG00000171657	1.434006695	2.630693381	1.93913835	2.056798925	-3.364398892	-1.750348766	0.027953245	38	G protein-coupled receptor 82 [Source:HGNC Symbol;Acc:4533]	GPR82	27197
ENSG00000126368	3.190857192	1.325910557	2.96068616	1.848426869	3.457826151	1.789865336	0.029406888	39	nuclear receptor subfamily 1, group D, member 1 [Source:HGNC Symbol;Acc:7962]	NR1D1	9572
ENSG00000179941	5.007060443	2.613058004	2.07962881	3.181946687	2.749682655	1.459265124	0.008802593	40	Bardet-Biedl syndrome 10 [Source:HGNC Symbol;Acc:26291]	BBS10	79738

ENSG00000179253	0.423394157	1.625025935	0.44704363	1.148914669	-3.324761408	-1.733250814	0.029143662	41	Uncharacterized protein [Source:UniProtKB/TrEMBL;Acc:Q5VZ43]	RP11-429E11.3	
ENSG00000159761	1.200875788	2.972005772	1.10918479	1.760066125	-2.693166708	-1.429303536	0.004828092	42	chromosome 16 open reading frame 86 [Source:HGNC Symbol;Acc:33755]	C16orf86	388284
ENSG00000145386	3.872078199	1.85120187	2.91548768	1.720250932	3.057374226	1.612293151	0.023572942	43	cyclin A2 [Source:HGNC Symbol;Acc:1578]	CCNA2	890
ENSG00000128641	2.757665032	1.249559681	2.1991218	1.60992694	2.900185308	1.536145085	0.019987196	44	myosin IB [Source:HGNC Symbol;Acc:7596]	MYO1B	4430
ENSG00000106484	1.741191537	0.648644815	1.33912445	0.898480505	3.438795228	1.781903209	0.031997371	45	mesoderm specific transcript [Source:HGNC Symbol;Acc:7028]	MEST	4232
ENSG00000119514	4.511340418	2.398021756	2.20778265	2.46603309	3.064558761	1.615679368	0.025331287	46	UDP-N-acetyl-alpha-D-galactosamine:polypeptide N-acetylgalactosaminyltransferase 12 (GalNAc-T12) [Source:HGNC Symbol;Acc:19877]	GALNT12	79695
ENSG00000177628	0.898916177	2.380366455	1.10599067	2.378651767	-2.961347719	-1.566253901	0.022754287	47	glucosidase, beta, acid [Source:HGNC Symbol;Acc:4177]	GBA	2629
ENSG00000228716	10.88614872	5.698722886	6.90995242	4.311035123	3.321421143	1.731800663	0.031931989	48	dihydrofolate reductase [Source:HGNC Symbol;Acc:2861]	DHFR	1719
ENSG00000197502	4.265734081	2.483276678	2.28710419	2.358317831	2.87571748	1.523921947	0.021835169	49	Uncharacterized protein [Source:UniProtKB/TrEMBL;Acc:H7BY28]	AL627171.1	
ENSG00000174469	3.553650279	1.05463896	3.1948811	1.459223768	3.844721874	1.942879238	0.037382919	50	contactin associated protein-like 2 [Source:HGNC Symbol;Acc:13830]	CNTNAP2	26047
ENSG00000188997	6.23138531	2.788259346	3.50794334	1.541327255	2.656345962	1.409443055	0.010495342	51	potassium channel tetramerization domain containing 21 [Source:HGNC Symbol;Acc:27452]	KCTD21	283219
ENSG00000237541	13.78699327	38.52002935	31.7208423	43.29181409	-10.22897076	-3.354589083	0.042935696	52	major histocompatibility complex, class II, DQ alpha 2 [Source:HGNC Symbol;Acc:4943]	HLA-DQA2	3118
ENSG00000156510	5.186940963	3.708073429	3.09955807	4.798410568	2.872014409	1.522062987	0.023752198	53	hexokinase domain containing 1 [Source:HGNC Symbol;Acc:23302]	HKDC1	80201
ENSG00000169612	5.742381048	3.441184092	2.68388658	2.80523177	2.722871666	1.445128987	0.017745952	54	family with sequence similarity 103, member A1 [Source:HGNC Symbol;Acc:31022]	FAM103A1	83640
ENSG00000105647	4.18420434	2.450246218	2.01050113	2.243586288	2.620578336	1.389885236	0.009747331	55	phosphoinositide-3-kinase, regulatory subunit 2 (beta) [Source:HGNC Symbol;Acc:8980]	PIK3R2	5296
ENSG00000149131	9.310775823	39.01858223	10.6395491	64.56408903	-2.907830728	-1.539943289	0.028199526	56	serpin peptidase inhibitor, clade G (C1 inhibitor), member 1 [Source:HGNC Symbol;Acc:1228]	SERPING1	710
ENSG00000126709	47.58263265	256.8084016	24.7438914	386.0695188	-2.639792452	-1.400424505	0.011022318	57	interferon, alpha-inducible protein 6 [Source:HGNC Symbol;Acc:4054]	IFI6	2537
ENSG00000137720	3.24578422	1.962152419	2.04595781	2.145466293	2.767258105	1.468457212	0.022124758	58	chromosome 11 open reading frame 1 [Source:HGNC Symbol;Acc:1163]	C11orf1	64776
ENSG00000101417	2.928803675	1.702090371	1.64537068	1.895896108	2.787535859	1.478990364	0.023643139	59	peroxisomal membrane protein 4, 24kDa [Source:HGNC Symbol;Acc:15920]	PXMP4	11264
ENSG00000147138	2.158409233	0.720613286	1.60601605	0.648276939	3.169697083	1.664344974	0.03440287	60	G protein-coupled receptor 174 [Source:HGNC Symbol;Acc:30245]	GPR174	84636
ENSG00000050030	2.634817459	0.81554302	1.88768169	0.746905649	3.016902135	1.593067898	0.031887756	61	KIAA2022 [Source:HGNC Symbol;Acc:29433]	KIAA2022	340533
ENSG00000130827	4.664119914	2.281086	3.76936524	2.779514112	2.86778822	1.519938488	0.027661351	62	plexin A3 [Source:HGNC Symbol;Acc:9101]	PLXNA3	55558

ENSG00000172878	3.353091764	2.144165115	1.76117133	2.534724648	2.667609491	1.415547487	0.015645798	63	methionyl aminopeptidase type 1D (mitochondrial) [Source:HGNC Symbol;Acc:32583]	METAP1D	254042
ENSG00000103942	5.299764854	2.711646798	3.26503004	2.086421094	2.647134301	1.404431391	0.014821881	64	homer homolog 2 (Drosophila) [Source:HGNC Symbol;Acc:17513]	HOMER2	9455
ENSG00000135747	0.777454873	1.476052913	0.83819766	0.836547157	-3.051054047	-1.609307736	0.033389963	65	zinc finger protein 670 [Source:HGNC Symbol;Acc:28167]	ZNF670	93474
ENSG00000127585	10.77776746	4.661477222	6.30610322	3.339261008	2.566025368	1.359535433	0.010374636	66	F-box and leucine-rich repeat protein 16 [Source:HGNC Symbol;Acc:14150]	FBXL16	146330
ENSG00000146530	1.56224785	0.303279567	1.71957525	0.754618173	3.159884301	1.659871735	0.035402687	67	von Willebrand factor D and EGF domains [Source:HGNC Symbol;Acc:21897]	VWDE	221806
ENSG00000187801	1.686063518	0.470580944	1.29704382	0.62579152	3.347981715	1.743291649	0.03925817	68	ZFP69 zinc finger protein B [Source:HGNC Symbol;Acc:28053]	ZFP69B	65243
ENSG00000240403	1.229043916	2.818043682	1.95973502	2.362785726	-3.256580309	-1.703357805	0.038567791	69	killer cell immunoglobulin-like receptor, three domains, long cytoplasmic tail, 2 [Source:HGNC Symbol;Acc:6339]	KIR3DL2	727787
ENSG00000182853	1.42208253	3.724854559	1.80166934	3.456389316	-3.240468853	-1.696202567	0.03763959	70	vitelline membrane outer layer 1 homolog (chicken) [Source:HGNC Symbol;Acc:30387]	VMO1	284013
ENSG00000166165	3.111608964	6.632964796	3.16687769	5.798420873	-2.835927111	-1.503820453	0.030095249	71	creatine kinase, brain [Source:HGNC Symbol;Acc:1991]	CKB	1152
ENSG00000146243	2.24751991	5.880493601	1.5646049	3.84044483	-2.620274242	-1.389717815	0.014854768	72	interleukin-1 receptor-associated kinase 1 binding protein 1 [Source:HGNC Symbol;Acc:17368]	IRAK1BP1	134728
ENSG00000017427	1.944660856	1.158126673	1.43616646	1.788919166	3.240308662	1.696131247	0.039039698	73	insulin-like growth factor 1 (somatomedin C) [Source:HGNC Symbol;Acc:5464]	IGF1	3479
ENSG00000143382	3.768030659	7.733642147	2.77537473	4.157291242	-2.56252883	-1.357568236	0.012204511	74	ADAMTS-like 4 [Source:HGNC Symbol;Acc:19706]	ADAMTSL4	54507
ENSG00000157429	3.06293019	1.501374753	1.99228431	1.541320302	2.891365235	1.53175086	0.032612304	75	zinc finger protein 19 [Source:HGNC Symbol;Acc:12981]	ZNF19	7567
ENSG00000243452	1.699235349	0.395344903	2.46597801	0.436371045	3.173944438	1.666276873	0.039482458	76	neuroblastoma breakpoint family, member 15 [Source:HGNC Symbol;Acc:28791]	NBPF15	284565
ENSG00000168062	2.161044706	7.636985623	2.1518972	7.193535415	-3.697893819	-1.8867038	0.044105147	77	basic leucine zipper transcription factor, ATF-like 2 [Source:HGNC Symbol;Acc:25163]	BATF2	116071
ENSG00000168646	9.798338515	5.349996653	3.99349519	4.551954808	2.985137847	1.577797553	0.036861879	78	axin 2 [Source:HGNC Symbol;Acc:904]	AXIN2	8313
ENSG00000135114	4.064978243	17.31310266	2.10731929	25.0998927	-2.734815996	-1.451443769	0.031712414	79	2'-5'-oligoadenylate synthetase-like [Source:HGNC Symbol;Acc:8090]	OASL	8638
ENSG00000107104	8.211576981	3.601911326	7.00014039	2.290394689	2.53101705	1.339717226	0.018039811	80	KN motif and ankyrin repeat domains 1 [Source:HGNC Symbol;Acc:19309]	KANK1	23189
ENSG00000169435	1.94969374	0.419841663	1.39649444	0.732474845	3.883898789	1.957505606	0.049040053	81	Ras association (RalGDS/AF-6) domain family member 6 [Source:HGNC Symbol;Acc:20796]	RASSF6	166824
ENSG00000187583	2.543559964	0.785058801	2.00702265	0.910089229	3.154308707	1.657323861	0.043276156	82	pleckstrin homology domain containing, family N member 1 [Source:HGNC Symbol;Acc:25284]	PLEKHN1	84069
ENSG00000179299	1.229106479	3.264505169	1.48794641	2.2047985	-3.58353156	-1.84138206	0.048700399	83	NOP2/Sun domain family, member 7 [Source:HGNC Symbol;Acc:25857]	NSUN7	79730

ENSG00000137965	21.82062074	58.3513795	19.7501367	76.75725275	-2.543107039	-1.346592186	0.021896656	84	interferon-induced protein 44 [Source:HGNC Symbol;Acc:16938]	IFI44	10561
ENSG00000188290	2.337278687	6.155670995	1.57669416	4.861910297	-2.69987601	-1.432893154	0.033343183	85	hes family bHLH transcription factor 4 [Source:HGNC Symbol;Acc:24149]	HES4	57801
ENSG00000087884	3.583059526	5.550557255	3.53671066	3.321416528	-2.501003433	-1.322507038	0.018687279	86	adipogenesis associated, Mth938 domain containing [Source:HGNC Symbol;Acc:30205]	AAMDC	28971
ENSG00000196126	628.4250605	412.9318782	373.792563	437.6150216	2.553847825	1.352672563	0.024548044	87	major histocompatibility complex, class II, DR beta 1 [Source:HGNC Symbol;Acc:4948]	HLA-DRB1	100996809
ENSG00000062582	1.122277227	1.826229878	1.49983562	0.948819894	-2.816504156	-1.4939056	0.039219267	88	mitochondrial ribosomal protein S24 [Source:HGNC Symbol;Acc:14510]	MRPS24	64951
ENSG00000118113	6.984159821	5.07283227	12.4725414	5.301654408	-3.053364721	-1.610399928	0.043988444	89	matrix metallopeptidase 8 (neutrophil collagenase) [Source:HGNC Symbol;Acc:7175]	MMP8	4317
ENSG00000163599	4.021430595	1.731383434	2.34760496	1.528353223	2.695379249	1.430488279	0.034929524	90	cytotoxic T-lymphocyte-associated protein 4 [Source:HGNC Symbol;Acc:2505]	CTLA4	1493
ENSG00000181754	7.746830423	4.804176882	3.11143973	3.558272773	2.421882577	1.276128919	0.019309159	92	adhesion molecule with Ig-like domain 1 [Source:HGNC Symbol;Acc:20824]	AMIGO1	57463
ENSG00000157077	4.598132633	2.274252524	2.92159789	2.223389272	2.697693985	1.431726704	0.036365592	93	zinc finger, FYVE domain containing 9 [Source:HGNC Symbol;Acc:6775]	ZFYVE9	9372
ENSG00000133106	41.28996874	125.4082315	31.4301137	138.6279957	-2.345013214	-1.229596052	0.01343629	94	epithelial stromal interaction 1 (breast) [Source:HGNC Symbol;Acc:16465]	EPSTI1	94240
ENSG00000204311	0.255060397	1.352204307	0.4475968	1.861339143	-2.881357701	-1.526748773	0.043166426	96	deafness, autosomal recessive 59 [Source:HGNC Symbol;Acc:29502]	DFNB59	494513
ENSG00000157734	17.00087802	11.74390263	11.5945522	9.29943121	2.986733879	1.578568698	0.044903064	97	sorting nexin 22 [Source:HGNC Symbol;Acc:16315]	SNX22	79856
ENSG00000137959	37.60312345	215.8786815	40.9147725	375.7129202	-2.948773869	-1.560115191	0.044001571	98	interferon-induced protein 44-like [Source:HGNC Symbol;Acc:17817]	IFI44L	10964
ENSG00000143502	3.821018427	1.745115872	1.94628978	1.455848198	2.567279584	1.360240418	0.03176168	100	sushi domain containing 4 [Source:HGNC Symbol;Acc:25470]	SUSD4	55061
ENSG00000141497	2.191482127	4.72050527	1.08669998	1.66700372	-2.326814804	-1.218356388	0.01221152	101	zinc finger, MYND-type containing 15 [Source:HGNC Symbol;Acc:20997]	ZMYND15	84225
ENSG00000196372	3.327500144	2.324499984	1.52595892	2.269014041	2.488053197	1.315017332	0.023857177	102	ankyrin repeat and SOCS box containing 13 [Source:HGNC Symbol;Acc:19765]	ASB13	79754
ENSG00000178338	7.456609039	4.82519188	3.8422094	3.468893316	2.401703651	1.264058146	0.020710429	103	zinc finger protein 354B [Source:HGNC Symbol;Acc:17197]	ZNF354B	117608
ENSG00000244617	2.321203184	3.132320791	3.79117378	2.057426962	-2.992248128	-1.581229813	0.047899151	105	aspartic peptidase, retroviral-like 1 [Source:HGNC Symbol;Acc:26321]	ASPRV1	151516
ENSG00000167637	1.180389707	2.853559393	1.34617584	2.485724267	-2.666063311	-1.41471104	0.039376973	107	zinc finger protein 283 [Source:HGNC Symbol;Acc:13077]	ZNF283	284349
ENSG00000149489	2.387534835	0.873026513	2.42890163	0.879438665	2.697717509	1.431739285	0.041809701	108	retinal outer segment membrane protein 1 [Source:HGNC Symbol;Acc:10254]	ROM1	6094

ENSG00000112796	2.071328883	3.445935386	2.15522535	2.593165678	-2.703467486	-1.434811006	0.042995726	109	ectonucleotide pyrophosphatase/phosphodiesterase 5 (putative) [Source:HGNC Symbol;Acc:13717]	ENPP5	59084
ENSG00000100084	3.610189631	1.699606193	2.89200319	1.291390555	2.435872986	1.284438908	0.031232245	110	histone cell cycle regulator [Source:HGNC Symbol;Acc:4916]	HIRA	7290
ENSG00000185630	12.81770917	8.548587998	6.54107177	8.267886228	2.553994911	1.352755651	0.03648169	111	pre-B-cell leukemia homeobox 1 [Source:HGNC Symbol;Acc:8632]	PBX1	5087
ENSG00000120217	5.317922902	10.90137557	5.63783408	7.24201134	-2.507382571	-1.326182136	0.034221154	113	CD274 molecule [Source:HGNC Symbol;Acc:17635]	CD274	29126
ENSG00000182768	2.616359814	4.925698997	2.06480759	2.986306039	-2.490657865	-1.316526856	0.033738654	114	neugrin, neurite outgrowth associated [Source:HGNC Symbol;Acc:18077]	NGRN	51335
ENSG00000167995	14.40416218	25.8764345	11.5627544	12.29455629	-2.387705941	-1.255625172	0.030176508	115	bestrophin 1 [Source:HGNC Symbol;Acc:12703]	BEST1	7439
ENSG00000133131	9.614409557	4.800838082	4.74910231	2.777404998	2.214930524	1.147261446	0.007120645	117	MORC family CW-type zinc finger 4 [Source:HGNC Symbol;Acc:23485]	MORC4	79710
ENSG00000151729	1.786752831	0.940013279	1.18907828	1.373328185	2.786075248	1.478234224	0.049360098	119	solute carrier family 25 (mitochondrial carrier; adenine nucleotide translocator), member 4 [Source:HGNC Symbol;Acc:10990]	SLC25A4	291
ENSG00000134326	10.04238899	28.23901122	9.90229267	34.11531789	-2.578754992	-1.366674709	0.041257756	121	cytidine monophosphate (UMP-CMP) kinase 2, mitochondrial [Source:HGNC Symbol;Acc:27015]	CMPK2	129607
ENSG00000127580	4.76963319	2.084311833	4.0163791	1.974096892	2.647385439	1.404568255	0.043981455	122	WD repeat domain 24 [Source:HGNC Symbol;Acc:20852]	WDR24	84219
ENSG00000154451	120.1288403	283.4804149	69.4399539	194.5607269	-2.203641745	-1.139889698	0.007359266	124	guanylate binding protein 5 [Source:HGNC Symbol;Acc:19895]	GBP5	115362
ENSG00000182809	11.36729593	4.935115557	9.77993037	4.413084256	2.690240112	1.427734944	0.047404591	125	cysteine-rich protein 2 [Source:HGNC Symbol;Acc:2361]	CRIP2	1397
ENSG00000029534	10.73928629	8.182739709	6.46867037	6.415390087	2.310632157	1.208287607	0.028721925	126	ankyrin 1, erythrocytic [Source:HGNC Symbol;Acc:492]	ANK1	286
ENSG00000120896	4.875837659	2.082013672	3.68900204	1.102817409	2.319373503	1.213735165	0.031202646	127	sorbin and SH3 domain containing 3 [Source:HGNC Symbol;Acc:30907]	SORBS3	10174
ENSG00000165821	2.811022803	1.305560137	2.388795	1.133898637	2.679260946	1.421835099	0.048743529	128	spalt-like transcription factor 2 [Source:HGNC Symbol;Acc:10526]	SALL2	6297
ENSG00000148331	6.877224046	4.867982323	1.95922632	2.839149761	2.276394095	1.186750342	0.027292403	129	ankyrin repeat and SOCS box containing 6 [Source:HGNC Symbol;Acc:17181]	ASB6	140459
ENSG00000138646	26.83544095	102.971069	17.2693738	158.474577	-2.26070624	-1.176773538	0.023663505	130	HECT and RLD domain containing E3 ubiquitin protein ligase 5 [Source:HGNC Symbol;Acc:24368]	HERC5	51191
ENSG00000247626	2.994361895	1.906384981	1.79237587	1.988151501	2.678084095	1.421201264	0.049388667	131	methionyl-tRNA synthetase 2, mitochondrial [Source:HGNC Symbol;Acc:25133]	MARS2	92935
ENSG00000126246	3.511304559	2.570036787	2.19025221	2.314290454	2.562720508	1.357676146	0.045034455	132	IGF-like family receptor 1 [Source:HGNC Symbol;Acc:23620]	IGFLR1	79713
ENSG00000155592	4.002408973	2.197924801	1.90066214	1.931900458	2.285908319	1.192767543	0.030060248	133	zinc finger with KRAB and SCAN domains 2 [Source:HGNC Symbol;Acc:25677]	ZKSCAN2	342357
ENSG00000240972	3.11539029	1.421196574	1.76655235	0.974822044	2.300080169	1.201684147	0.03169388	134	macrophage migration inhibitory factor (glycosylation-inhibiting factor) [Source:HGNC Symbol;Acc:7097]	MIF	4282
ENSG00000175906	2.420422309	1.358388843	1.51675356	1.326229932	2.553286346	1.352355342	0.04507066	135	ADP-ribosylation factor-like 4D [Source:HGNC Symbol;Acc:656]	ARL4D	379

ENSG00000105245	3.150778374	2.20509292	1.40917254	1.69435395	2.227700821	1.155555493	0.023601119	136	numb homolog (Drosophila)-like [Source:HGNC Symbol;Acc:8061]	NUMBL	9253
ENSG00000168005	1.280790484	1.835080988	1.80739618	0.913038526	-2.50350771	-1.323950899	0.045284897	140	chromosome 11 open reading frame 84 [Source:HGNC Symbol;Acc:25115]	C11orf84	144097
ENSG00000177706	16.09563725	7.907946236	6.75328797	3.670558607	2.106243005	1.074671895	0.002989513	141	family with sequence similarity 20, member C [Source:HGNC Symbol;Acc:22140]	FAM20C	56975
ENSG00000100372	9.795181864	6.411520606	4.07528561	4.031343038	2.152172134	1.105793471	0.013558108	142	solute carrier family 25 (mitochondrial carrier; peroxisomal membrane protein, 34kDa), member 17 [Source:HGNC Symbol;Acc:10987]	SLC25A17	10478
ENSG00000158321	35.69548123	28.62004255	13.4844491	33.24493556	2.168857057	1.116934972	0.017026518	144	autism susceptibility candidate 2 [Source:HGNC Symbol;Acc:14262]	AUTS2	26053
ENSG00000180509	9.873965991	15.28189539	8.94666075	7.122337572	-2.186745197	-1.128785125	0.021616856	145	potassium voltage-gated channel, Isk-related family, member 1 [Source:HGNC Symbol;Acc:6240]	KCNE1	3753
ENSG00000006432	1.677090125	0.883883224	0.89949284	1.156999705	2.530332566	1.339327013	0.049364863	148	mitogen-activated protein kinase kinase kinase 9 [Source:HGNC Symbol;Acc:6861]	MAP3K9	4293
ENSG00000110921	9.225584257	4.394835317	3.46947254	1.648199812	2.106568563	1.074894872	0.009005784	149	mevalonate kinase [Source:HGNC Symbol;Acc:7530]	MVK	4598
ENSG00000145194	1.429435625	2.487516038	1.61092973	0.739564414	-2.420362987	-1.275223428	0.046862546	151	endothelin converting enzyme 2 [Source:HGNC Symbol;Acc:13275]	ECE2	9718
ENSG00000213213	1.731241818	1.253097743	0.91847676	1.319888054	2.400178793	1.263141878	0.046234021	153	coiled-coil domain containing 183 [Source:HGNC Symbol;Acc:28236]	CCDC183	84960
ENSG00000128039	7.162440208	4.023462043	3.88291496	2.510501123	2.155770502	1.1082036	0.02160646	154	steroid 5 alpha-reductase 3 [Source:HGNC Symbol;Acc:25812]	SRD5A3	79644
ENSG00000157601	59.26022914	265.9902031	41.6814855	453.0984091	-2.279320451	-1.188603768	0.039642651	157	myxovirus (influenza virus) resistance 1, interferon-inducible protein p78 (mouse) [Source:HGNC Symbol;Acc:7532]	MX1	4599
ENSG00000159788	7.471132484	10.98611055	5.3570523	3.829810054	-2.128150731	-1.089600336	0.020218991	158	regulator of G-protein signaling 12 [Source:HGNC Symbol;Acc:9994]	RGS12	6002
ENSG00000183723	5.108815534	3.099352081	2.42669081	2.633504991	2.216705795	1.148417306	0.03369576	160	CKLF-like MARVEL transmembrane domain containing 4 [Source:HGNC Symbol;Acc:19175]	CMTM4	146223
ENSG00000167685	9.535384398	5.416348274	1.53693926	2.356486744	2.030543214	1.021865731	0.002205124	163	zinc finger protein 444 [Source:HGNC Symbol;Acc:16052]	ZNF444	55311
ENSG00000184903	4.654091908	2.295324365	2.06092105	1.290209286	2.173352731	1.11992234	0.031768775	166	IMP2 inner mitochondrial membrane peptidase-like (S. cerevisiae) [Source:HGNC Symbol;Acc:14598]	IMMP2L	83943
ENSG00000182010	43.50645982	22.91132079	15.6946823	12.56684436	2.079958134	1.056554489	0.014850658	167	rhotekin 2 [Source:HGNC Symbol;Acc:19364]	RTKN2	219790
ENSG00000081026	5.287879607	2.51486479	2.83419747	1.239364733	2.204317043	1.140331739	0.035988317	169	membrane associated guanylate kinase, WW and PDZ domain containing 3 [Source:HGNC Symbol;Acc:29647]	MAGI3	260425
ENSG00000221866	5.772352708	3.444018015	2.86408755	3.39816942	2.238505636	1.162535951	0.040670353	172	plexin A4 [Source:HGNC Symbol;Acc:9102]	PLXNA4	91584
ENSG00000242013	6.303829655	4.069751396	2.1834204	3.079041851	2.165431421	1.114654483	0.033869465	178	ubiquitin specific peptidase 27, X-linked [Source:HGNC Symbol;Acc:13486]	USP27X	389856
ENSG00000125650	3.450917289	2.31028997	1.86881698	1.900054267	2.245752381	1.167198864	0.043228575	179	persephin [Source:HGNC Symbol;Acc:9579]	PSPN	5623
ENSG00000130589	34.61023987	79.50987255	18.3873685	63.52225791	-2.012692214	-1.009126568	0.007224964	180	helicase with zinc finger 2, transcriptional coactivator [Source:HGNC Symbol;Acc:30021]	HELZ2	85441

ENSG00000172824	3.381034542	1.75451779	2.68210807	1.405473781	2.275117656	1.185941155	0.046751784	181	carboxylesterase 4A [Source:HGNC Symbol;Acc:26741]	CES4A	283848
ENSG00000103494	3.921377789	2.327643001	1.86472801	1.548349886	2.126350974	1.088379747	0.030913751	182	RPGRIP1-like [Source:HGNC Symbol;Acc:29168]	RPGRIP1L	23322
ENSG00000196440	5.080850234	3.76713548	1.26965418	3.483262195	2.090143744	1.063602163	0.024625081	184	armadillo repeat containing, X-linked 4 [Source:HGNC Symbol;Acc:28615]	ARMCX4	100131755
ENSG00000196220	4.321453637	2.512153548	1.75785689	1.774720558	2.179759779	1.124169151	0.037157208	185	SLIT-ROBO Rho GTPase activating protein 3 [Source:HGNC Symbol;Acc:19744]	SRGAP3	9901
ENSG00000171421	8.16611841	4.704887789	3.35051616	4.167879828	2.185655958	1.128066326	0.042375861	191	mitochondrial ribosomal protein L36 [Source:HGNC Symbol;Acc:14490]	MRPL36	64979
ENSG00000105426	4.742503005	2.549008798	2.79294628	1.887787992	2.189567028	1.130645615	0.042903608	192	protein tyrosine phosphatase, receptor type, S [Source:HGNC Symbol;Acc:9681]	PTPRS	5802
ENSG00000117228	118.0400667	287.1186017	51.6208742	228.3047831	-2.047441115	-1.03382196	0.022089956	193	guanylate binding protein 1, interferon-inducible [Source:HGNC Symbol;Acc:4182]	GBP1	2633
ENSG00000107020	2.40324924	4.037609657	1.80439442	1.712652777	-2.073009539	-1.051726755	0.02753722	194	plasminogen receptor, C-terminal lysine transmembrane protein [Source:HGNC Symbol;Acc:23633]	PLGRKT	55848
ENSG00000147889	3.876974286	3.265262847	2.40172953	3.471159623	2.196019807	1.134891067	0.048159205	207	cyclin-dependent kinase inhibitor 2A [Source:HGNC Symbol;Acc:1787]	CDKN2A	1029
ENSG00000230510	1.340437281	2.66967036	1.23406733	1.616437122	-2.14966787	-1.104113776	0.043855849	209	PPP5 tetratricopeptide repeat domain containing 1 [Source:HGNC Symbol;Acc:44209]	PPP5D1	100506012
ENSG00000166228	3.528651815	6.428457021	3.02457611	4.082087017	-2.081054763	-1.05731493	0.039044043	214	pterin-4 alpha-carbinolamine dehydratase/dimerization cofactor of hepatocyte nuclear factor 1 alpha [Source:HGNC Symbol;Acc:8646]	PCBD1	5092
ENSG00000123552	8.337943776	5.891524833	3.19700724	3.345384081	2.106489971	1.074841047	0.043970862	215	ubiquitin specific peptidase 45 [Source:HGNC Symbol;Acc:20080]	USP45	85015
ENSG00000105352	6.214067826	9.498138083	4.66174689	4.722345903	-2.141144499	-1.098382162	0.04840141	219	carcinoembryonic antigen-related cell adhesion molecule 4 [Source:HGNC Symbol;Acc:1816]	CEACAM4	1089
ENSG00000152439	7.829106015	5.66102506	3.01724579	3.827569583	2.09042611	1.063797049	0.04439163	222	zinc finger protein 773 [Source:HGNC Symbol;Acc:30487]	ZNF773	374928
ENSG00000107779	3.384452841	2.084488733	2.12937154	1.90309236	2.02894195	1.020727589	0.036683518	223	bone morphogenetic protein receptor, type IA [Source:HGNC Symbol;Acc:1076]	BMPR1A	657
ENSG00000204475	3.452480241	6.543620804	2.30356007	3.453217451	-2.050805219	-1.036190474	0.041143576	228	natural cytotoxicity triggering receptor 3 [Source:HGNC Symbol;Acc:19077]	NCR3	259197
ENSG00000116991	15.62993866	28.11089471	11.6500486	14.40570029	-1.989100282	-0.992116012	0.03664229	237	signal-induced proliferation-associated 1 like 2 [Source:HGNC Symbol;Acc:23800]	SIPA1L2	57568
ENSG00000141012	10.52540387	19.10014798	3.18286648	5.614099817	-1.852536119	-0.889501671	0.001900313	239	galactosamine (N-acetyl)-6-sulfate sulfatase [Source:HGNC Symbol;Acc:4122]	GALNS	2588
ENSG00000204954	3.473590364	5.088861753	2.71817078	1.77962884	-2.00549414	-1.003957751	0.040705155	242	chromosome 12 open reading frame 73 [Source:HGNC Symbol;Acc:34450]	C12orf73	728568
ENSG00000187024	3.581195054	6.639253658	2.84822179	4.550490903	-1.969857177	-0.978091032	0.034849619	243	peptidyl-tRNA hydrolase 1 homolog (S. cerevisiae) [Source:HGNC Symbol;Acc:27039]	PTRH1	138428

ENSG00000125434	3.582349192	2.434350903	2.08648154	2.475436182	2.05661733	1.04027338	0.048641944	246	solute carrier family 25, member 35 [Source:HGNC Symbol;Acc:31921]	SLC25A35	399512
ENSG00000204564	8.355442745	5.133255027	2.75077461	4.598393482	1.905560289	0.930215253	0.027035037	254	chromosome 6 open reading frame 136 [Source:HGNC Symbol;Acc:21301]	C6orf136	221545
ENSG00000166261	7.707400255	4.167568614	3.33416386	1.977339335	1.907185352	0.93144506	0.029081137	256	zinc finger protein 202 [Source:HGNC Symbol;Acc:12994]	ZNF202	7753
ENSG00000155906	2.730036636	1.309332156	1.80990649	0.988821332	1.971309704	0.97915445	0.039884884	257	required for meiotic nuclear division 1 homolog (S. cerevisiae) [Source:HGNC Symbol;Acc:21176]	RMND1	55005
ENSG00000139546	10.53512456	6.414744904	2.73307297	3.409784974	1.851317723	0.888552512	0.014533682	259	TAR (HIV-1) RNA binding protein 2 [Source:HGNC Symbol;Acc:11569]	TARBP2	6895
ENSG00000198105	11.47582795	9.21262504	5.20787283	7.631766138	2.002541585	1.001832202	0.048083808	260	zinc finger protein 248 [Source:HGNC Symbol;Acc:13041]	ZNF248	57209
ENSG00000243660	2.31606575	5.340684856	1.7037807	4.787901282	-1.936272492	-0.953281998	0.041000765	262	zinc finger protein 487 [Source:HGNC Symbol;Acc:23488]	ZNF487	
ENSG00000143156	2.520020929	4.75219902	1.75299152	2.033510605	-1.908378027	-0.93234698	0.040925804	270	NME/NM23 family member 7 [Source:HGNC Symbol;Acc:20461]	NME7	29922
ENSG00000131634	36.36926297	23.79624508	14.7643135	17.25956377	1.896024065	0.922977275	0.037856232	271	transmembrane protein 204 [Source:HGNC Symbol;Acc:14158]	TMEM204	79652
ENSG00000136997	8.352541644	4.849284899	5.36151686	2.98933926	1.916578388	0.938533006	0.042715057	272	v-myc avian myelocytomatosis viral oncogene homolog [Source:HGNC Symbol;Acc:7553]	MYC	4609
ENSG00000180758	5.181627195	3.455788049	2.42108634	1.940171305	1.917484621	0.939215007	0.046554788	280	G protein-coupled receptor 157 [Source:HGNC Symbol;Acc:23687]	GPR157	80045
ENSG00000176108	14.79099752	8.437451303	5.2766087	2.82339922	1.781846498	0.833373057	0.007422014	282	charged multivesicular body protein 6 [Source:HGNC Symbol;Acc:25675]	CHMP6	79643
ENSG00000108439	4.292116922	5.920239847	3.92470833	2.835816427	-1.905801507	-0.930397868	0.048282435	283	pyridoxamine 5'-phosphate oxidase [Source:HGNC Symbol;Acc:30260]	PNPO	55163
ENSG00000188215	5.925675056	4.4383019	2.15516482	3.189689348	1.811227557	0.856967813	0.031383592	285	DCN1, defective in cullin neddylation 1, domain containing 3 [Source:HGNC Symbol;Acc:28734]	DCUN1D3	123879
ENSG00000123545	11.26107607	5.77872149	7.32222727	2.457773525	1.808875115	0.855092807	0.039644863	297	NADH dehydrogenase (ubiquinone) complex I, assembly factor 4 [Source:HGNC Symbol;Acc:21034]	NDUFAF4	29078
ENSG00000177946	8.760294052	12.35437175	6.37624355	4.733018459	-1.799603618	-0.847679173	0.039631716	307	CENPB DNA-binding domains containing 1 [Source:HGNC Symbol;Acc:28272]	CENPBD1	92806
ENSG00000101452	7.440992293	4.238511909	2.12336411	1.4016543	1.78025579	0.832084545	0.034951018	313	DEAH (Asp-Glu-Ala-His) box polypeptide 35 [Source:HGNC Symbol;Acc:15861]	DHX35	60625
ENSG00000182093	11.28747364	8.016014564	3.59458472	5.791077412	1.789115541	0.839246559	0.039787996	318	tryptophan rich basic protein [Source:HGNC Symbol;Acc:12790]	WRB	7485
ENSG00000166928	9.114440509	14.87278773	5.4352722	7.670002989	-1.770135188	-0.823859546	0.034012395	320	membrane-spanning 4-domains, subfamily A, member 14 [Source:HGNC Symbol;Acc:30706]	MS4A14	84689
ENSG00000111846	9.185870545	16.42129257	5.35360245	8.005190627	-1.807097538	-0.853674378	0.047559269	322	glucosaminyl (N-acetyl) transferase 2, I-branching enzyme (I blood group) [Source:HGNC Symbol;Acc:4204]	GCNT2	2651
ENSG00000164308	40.96815743	73.44382136	22.3162038	41.87337792	-1.758792908	-0.814585621	0.033857158	327	endoplasmic reticulum aminopeptidase 2 [Source:HGNC Symbol;Acc:29499]	ERAP2	64167

ENSG00000180539	5.281406307	8.615850735	2.88496697	4.411449322	-1.761455455	-0.816767991	0.036376358	333	chromosome 9 open reading frame 139 [Source:HGNC Symbol;Acc:31426]	C9orf139	401563
ENSG00000215788	30.46549118	19.84915549	8.66598867	12.10652454	1.734352114	0.794396829	0.029564977	340	tumor necrosis factor receptor superfamily, member 25 [Source:HGNC Symbol;Acc:11910]	TNFRSF25	8718
ENSG00000130489	34.81103834	74.30319618	17.342777	73.65966906	-1.765253615	-0.819875471	0.040974027	350	SCO2 cytochrome c oxidase assembly protein [Source:HGNC Symbol;Acc:10604]	SCO2	9997
ENSG00000132530	209.6983463	496.9628766	101.139962	556.0853124	-1.773101218	-0.826274896	0.04607866	357	XIAP associated factor 1 [Source:HGNC Symbol;Acc:30932]	XAF1	54739
ENSG00000106367	8.1981314	5.570478791	3.29125873	2.990855272	1.78155243	0.833134942	0.048712895	358	adaptor-related protein complex 1, sigma 1 subunit [Source:HGNC Symbol;Acc:559]	AP1S1	1174
ENSG00000188313	63.36110352	123.5716005	24.5782759	78.95271022	-1.734327308	-0.794376194	0.036300878	362	phospholipid scramblase 1 [Source:HGNC Symbol;Acc:9092]	PLSCR1	5359
ENSG00000089127	40.66671679	82.57226414	18.6811139	70.85409809	-1.71943833	-0.781937372	0.047517496	401	2'-5'-oligoadenylate synthetase 1, 40/46kDa [Source:HGNC Symbol;Acc:8086]	OAS1	4938
ENSG00000115415	304.3916524	547.2435545	145.925419	343.4788076	-1.70383445	-0.768785165	0.047806686	413	signal transducer and activator of transcription 1, 91kDa [Source:HGNC Symbol;Acc:11362]	STAT1	6772
ENSG00000254004	12.49595999	7.736601407	5.21071442	2.723712596	1.674387844	0.743633743	0.042228	425	zinc finger protein 260 [Source:HGNC Symbol;Acc:13499]	ZNF260	339324
ENSG00000168016	50.10103581	82.96646428	16.3307575	25.71617315	-1.631455902	-0.706159993	0.040632626	454	tetratricopeptide repeat and ankyrin repeat containing 1 [Source:HGNC Symbol;Acc:29011]	TRANK1	9881
ENSG00000103064	45.5211978	30.83294393	13.1770131	13.95108145	1.607987964	0.685256608	0.031957959	458	solute carrier family 7 (amino acid transporter light chain, y+L system), member 6 [Source:HGNC Symbol;Acc:11064]	SLC7A6	9057
ENSG00000114388	20.07742073	12.73805273	8.38279386	4.587877331	1.580557628	0.660433638	0.015226991	462	nitrogen permease regulator-like 2 (S. cerevisiae) [Source:HGNC Symbol;Acc:24969]	NPRL2	10641
ENSG00000069966	40.30462028	28.18212804	11.782564	17.85100594	1.633448422	0.707920901	0.044749284	467	guanine nucleotide binding protein (G protein), beta 5 [Source:HGNC Symbol;Acc:4401]	GNB5	10681
ENSG00000177409	114.2586112	216.1878176	32.7033703	161.1652887	-1.629261803	-0.704218447	0.043042076	468	sterile alpha motif domain containing 9-like [Source:HGNC Symbol;Acc:1349]	SAMD9L	219285
ENSG00000086189	12.47732616	8.376053658	4.08463696	4.190811488	1.625737866	0.701094656	0.042506443	470	DIM1 dimethyladenosine transferase 1 homolog (S. cerevisiae) [Source:HGNC Symbol;Acc:30217]	DIMT1	27292
ENSG00000108395	26.08734127	17.49850051	5.59067506	4.242559296	1.5588237	0.640457771	0.021688278	495	tripartite motif containing 37 [Source:HGNC Symbol;Acc:7523]	TRIM37	4591
ENSG00000179152	7.383463447	12.22535165	1.35492821	4.311217884	-1.562485549	-0.643842847	0.036303193	521	T cell activation inhibitor, mitochondrial [Source:HGNC Symbol;Acc:25241]	TCAIM	285343
ENSG00000018610	19.11154869	13.24531416	6.53312107	5.214183748	1.585863316	0.665268432	0.048967589	541	chromosome X open reading frame 56 [Source:HGNC Symbol;Acc:26239]	CXorf56	63932
ENSG00000156804	35.22640365	25.08242182	9.52568743	14.83978999	1.582925789	0.66259362	0.0496427	545	F-box protein 32 [Source:HGNC Symbol;Acc:16731]	FBXO32	114907
ENSG00000112144	24.59770073	16.50174248	5.15279898	4.013254945	1.548133844	0.630530205	0.032755671	547	intestinal cell (MAK-like) kinase [Source:HGNC Symbol;Acc:21219]	ICK	22858

Compared populations:

D2T RA High inflammation high pain vs D2T RA High inflammation low pain

Fold change describes the size of the difference in gene expression (given in mean and standard deviation, sd) between the compared groups. The values are always in relation to the group used as a base level group (reference). P-value describes the reliability of the change in expression value between the compared groups. Results were considered significant with a fold change of 1.2 and a p-value cutoff of 0.05.

Supplementary Material 20 - Summary of enrichment analysis of DE genes listing the top 25 processes or pathways based different databases (Gene Ontology and Reactome)

Gene Ontology Biological Process					
**GO.ID**	**Term**	**Annotated**	**Significant**	**Expected**	**p value**
GO:0051607	defense response to virus	253	18	4.38	0.00000037
GO:0140546	defense response to symbiont	253	18	4.38	0.00000037
GO:0009615	response to virus	321	20	5.56	0.00000069
GO:0070106	interleukin-27-mediated signaling pathwa...	7	4	0.12	0.0000029
GO:0045071	negative regulation of viral genome repl...	46	6	0.8	0.00013
GO:0045069	regulation of viral genome replication	71	7	1.23	0.00022
GO:0048525	negative regulation of viral process	71	7	1.23	0.00022
GO:0045916	negative regulation of complement activa...	8	3	0.14	0.00027
GO:0010812	negative regulation of cell-substrate ad...	35	5	0.61	0.00031
GO:0048146	positive regulation of fibroblast prolif...	36	5	0.62	0.00036
GO:0048532	anatomical structure arrangement	9	3	0.16	0.0004
GO:0002053	positive regulation of mesenchymal cell ...	10	3	0.17	0.00056
GO:0002921	negative regulation of humoral immune re...	10	3	0.17	0.00056
GO:0140374	antiviral innate immune response	41	5	0.71	0.00067
GO:0035456	response to interferon-beta	26	4	0.45	0.00097
GO:0030449	regulation of complement activation	12	3	0.21	0.001
GO:0002831	regulation of response to biotic stimulu...	391	16	6.77	0.00124
GO:0051707	response to other organism	976	30	16.91	0.00134
GO:0043207	response to external biotic stimulus	977	30	16.93	0.00136
GO:0050792	regulation of viral process	126	8	2.18	0.00153
GO:0050896	response to stimulus	5072	108	87.87	0.00154
GO:0006958	complement activation, classical pathway	14	3	0.24	0.00162
GO:0010464	regulation of mesenchymal cell prolifera...	14	3	0.24	0.00162
GO:0032102	negative regulation of response to exter...	291	13	5.04	0.00164

GO:0045087	innate immune response	646	22	11.19	0.00177

Reactome				
**Reactome.ID**	**Term**	**GeneRatio**	**BgRatio**	**p value**
R-HSA-909733	Interferon alpha/beta signaling	10/116	61/6966	4.90136E-08
R-HSA-913531	Interferon Signaling	15/116	215/6966	2.35E-06
R-HSA-1538133	G0 and Early G1	4/116	23/6966	0.000506177
R-HSA-877300	Interferon gamma signaling	6/116	73/6966	0.00128606
R-HSA-9671555	Signaling by PDGFR in disease	3/116	18/6966	0.003059248
R-HSA-201556	Signaling by ALK	3/116	22/6966	0.005500834
R-HSA-69205	G1/S-Specific Transcription	3/116	23/6966	0.006250033
R-HSA-1169410	Antiviral mechanism by IFN-stimulated genes	7/116	132/6966	0.00630065
R-HSA-6804116	TP53 Regulates Transcription of Genes Involved in G1 Cell Cycle Arrest	2/116	10/6966	0.01133732
R-HSA-399956	CRMPs in Sema3A signaling	2/116	11/6966	0.013707057
R-HSA-9673767	Signaling by PDGFRA transmembrane, juxtamembrane and kinase domain mutants	2/116	11/6966	0.013707057
R-HSA-9673770	Signaling by PDGFRA extracellular domain mutants	2/116	11/6966	0.013707057
R-HSA-388841	Costimulation by the CD28 family	4/116	57/6966	0.014641527
R-HSA-399955	SEMA3A-Plexin repulsion signaling by inhibiting Integrin adhesion	2/116	12/6966	0.016271041
R-HSA-399954	Sema3A PAK dependent Axon repulsion	2/116	14/6966	0.021953881
R-HSA-389948	PD-1 signaling	2/116	15/6966	0.025059239
R-HSA-453279	Mitotic G1 phase and G1/S transition	6/116	137/6966	0.026413165
R-HSA-1169408	ISG15 antiviral mechanism	4/116	69/6966	0.027551081
R-HSA-1362277	Transcription of E2F targets under negative control by DREAM complex	2/116	17/6966	0.031765359
R-HSA-9013508	NOTCH3 Intracellular Domain Regulates Transcription	2/116	17/6966	0.031765359
R-HSA-6791312	TP53 Regulates Transcription of Cell Cycle Genes	3/116	43/6966	0.034277054
R-HSA-2559586	DNA Damage/Telomere Stress Induced Senescence	3/116	44/6966	0.036351009
R-HSA-9615017	FOXO-mediated transcription of oxidative stress, metabolic and neuronal genes	2/116	19/6966	0.039090623
R-HSA-9603798	Class I peroxisomal membrane protein import	2/116	20/6966	0.042970504
R-HSA-9669938	Signaling by KIT in disease	2/116	20/6966	0.042970504

Compared populations:

D2T RA High inflammation high pain vs D2T RA High inflammation low pain

Enrichment analysis for the differentially expressed filtered gene lists tests whether the genes within a certain Reactome pathway or GO term are statistically over-represented in a given comparison. Annotated: number of genes associated with the given term. R: Reactome database. Significant: number of statistically significant associated with the given term. Expected: expected number of genes associated with the given term in the DE gene list. Gene ratio: ratio of number of genes in the DE list that overlap with genes associated with the given term, and the number of genes in the DE list which overlap with genes associated with all terms in the Reactome database. Bg ratio: ratio of the number of genes associated with the given term, and the total number of genes associated with Reactome terms.

Supplementary Material 21 - List of differentially expressed (DE) genes

ID	mean_LowInflammation_HighPain	mean_LowInflammation_LowPain	sd_LowInflammation_HighPain	sd_LowInflammation_LowPain	FC	logFC	p value	avg Rank	Description	geneName	EntrezID
ENSG00000183473	6.922959229	2.255984452	3.578447143	1.489253311	3.6750052	1.877746291	0.000778045	1	somatostatin receptor 3 [Source:HGNC Symbol;Acc:11332]	SSTR3	6753
ENSG00000162383	0.387259405	2.287250522	0.794743625	2.252805136	-4.624128436	-2.20918147	0.008126069	2	solute carrier family 1 (glutamate transporter), member 7 [Source:HGNC Symbol;Acc:10945]	SLC1A7	6512
ENSG00000163814	0.950324559	2.229873519	1.228696732	1.435591977	-3.438428113	-1.781749184	0.005716721	3	CUB domain containing protein 1 [Source:HGNC Symbol;Acc:24357]	CDCP1	64866
ENSG00000143942	2.799853552	1.2137484	1.831624205	1.034017757	3.240875248	1.696383488	0.001689396	4	ChaC, cation transport regulator homolog 2 (E. coli) [Source:HGNC Symbol;Acc:32363]	CHAC2	494143
ENSG00000223609	1.57856403	10.69023714	1.995656755	12.6429788	-7.416461139	-2.89073095	0.011719479	5	hemoglobin, delta [Source:HGNC Symbol;Acc:4829]	HBD	3045
ENSG00000143416	0.477220351	2.505516886	0.560005672	2.214633037	-4.150802853	-2.053390411	0.011616033	6	selenium binding protein 1 [Source:HGNC Symbol;Acc:10719]	SELENBP1	8991
ENSG00000182534	0.731547706	2.513151956	0.850043392	1.959662402	-3.212014437	-1.683478377	0.018492665	7	matrix-remodelling associated 7 [Source:HGNC Symbol;Acc:7541]	MXRA7	439921
ENSG00000137310	1.952331936	3.648408553	1.928687123	1.898780871	-2.703113829	-1.434622266	0.009113776	8	transcription factor 19 [Source:HGNC Symbol;Acc:11629]	TCF19	6941
ENSG00000142156	1.937529212	1.084979487	1.946420319	1.89067106	2.871624211	1.521866966	0.018850964	9	collagen, type VI, alpha 1 [Source:HGNC Symbol;Acc:2211]	COL6A1	1291
ENSG00000055732	3.467446235	1.674564148	1.976715843	1.543144273	2.623445984	1.391463086	0.009136263	10	mucolipin 3 [Source:HGNC Symbol;Acc:13358]	MCOLN3	55283
ENSG00000187474	1.159542359	2.932584019	1.081512721	1.809108415	-3.162544383	-1.661085727	0.022179984	11	formyl peptide receptor 3 [Source:HGNC Symbol;Acc:3828]	FPR3	2359
ENSG00000165895	2.829101149	1.330193936	1.730533174	0.970770305	2.647902363	1.404849926	0.017978958	12	Rho GTPase activating protein 42 [Source:HGNC Symbol;Acc:26545]	ARHGAP42	143872
ENSG00000117479	0.740515288	1.980663864	0.903055842	1.691601916	-2.877645868	-1.5248906	0.023365432	13	solute carrier family 19 (thiamine transporter), member 2 [Source:HGNC Symbol;Acc:10938]	SLC19A2	10560
ENSG00000186827	6.951698023	3.705276474	3.220933964	3.004045176	2.482655708	1.311884204	0.005443589	14	tumor necrosis factor receptor superfamily, member 4 [Source:HGNC Symbol;Acc:11918]	TNFRSF4	7293
ENSG00000138180	1.164945029	2.016017875	2.378002596	1.830931703	-2.993517771	-1.581841835	0.026804351	15	centrosomal protein 55kDa [Source:HGNC Symbol;Acc:1161]	CEP55	55165
ENSG00000225614	1.192541362	2.393571336	1.348799982	1.619299961	-2.567733257	-1.360495339	0.016928329	16	zinc finger protein 469 [Source:HGNC Symbol;Acc:23216]	ZNF469	84627

ENSG00000167840	1.42528934	2.966313935	1.186231565	1.850984323	-2.473050711	-1.306291823	0.006338216	17	zinc finger protein 232 [Source:HGNC Symbol;Acc:13026]	ZNF232	7775
ENSG00000142621	0.567681912	2.408501637	0.76797092	3.121225377	-3.166840157	-1.663044051	0.032391899	18	forkhead-associated (FHA) phosphopeptide binding domain 1 [Source:HGNC Symbol;Acc:29408]	FHAD1	114827
ENSG00000074590	1.295554805	3.551979564	1.377782564	2.44938326	-2.838033473	-1.504891605	0.026642035	19	NUAK family, SNF1-like kinase, 1 [Source:HGNC Symbol;Acc:14311]	NUAK1	9891
ENSG00000109099	0.583670164	1.6024155	0.947585319	1.491626201	-3.13959218	-1.650577171	0.033111325	20	peripheral myelin protein 22 [Source:HGNC Symbol;Acc:9118]	PMP22	5376
ENSG00000143847	2.69220638	1.316441036	1.507853212	1.121492539	2.592249971	1.374204844	0.023046446	21	protein tyrosine phosphatase, receptor type, f polypeptide (PTPRF), interacting protein (liprin), alpha 4 [Source:HGNC Symbol;Acc:9248]	PPFIA4	8497
ENSG00000168661	2.84851235	0.996051788	2.135903067	0.723863482	2.639610987	1.400325328	0.02725336	22	zinc finger protein 30 [Source:HGNC Symbol;Acc:13090]	ZNF30	90075
ENSG00000101977	1.671579568	3.583877331	1.4126629	1.543028783	-2.535409925	-1.342219021	0.022918196	23	MCF.2 cell line derived transforming sequence [Source:HGNC Symbol;Acc:6940]	MCF2	4168
ENSG00000111249	0.728817842	1.767507256	0.877967427	1.076567126	-2.7303959	-1.449110153	0.034016939	24	cut-like homeobox 2 [Source:HGNC Symbol;Acc:19347]	CUX2	23316
ENSG00000213213	0.876504874	2.47991502	0.46451911	1.371463597	-2.420896548	-1.27554143	0.015669721	25	coiled-coil domain containing 183 [Source:HGNC Symbol;Acc:28236]	CCDC183	84960
ENSG00000100908	2.060410518	0.924770744	1.108523598	0.773104651	2.422238071	1.276340668	0.021616707	26	ER membrane protein complex subunit 9 [Source:HGNC Symbol;Acc:20273]	EMC9	51016
ENSG00000187990	1.034803825	2.432235243	1.379259063	2.01117235	-2.786157466	-1.478276797	0.042806704	27	histone cluster 1, H2bg [Source:HGNC Symbol;Acc:4746]	HIST1H2BG	8347
ENSG00000196741	1.765214446	0.884570424	1.312767219	0.87991544	2.528962447	1.338545615	0.02974628	28	chromosome X open reading frame 24 [Source:HGNC Symbol;Acc:27333]	CXorf24	
ENSG00000164171	1.496623874	3.102408138	1.699723451	2.94918455	-2.596636927	-1.376644304	0.040130605	29	integrin, alpha 2 (CD49B, alpha 2 subunit of VLA-2 receptor) [Source:HGNC Symbol;Acc:6137]	ITGA2	3673
ENSG00000176945	6.083306825	3.12789351	3.290686437	2.940706497	2.625958042	1.392843865	0.042411698	30	mucin 20, cell surface associated [Source:HGNC Symbol;Acc:23282]	MUC20	200958
ENSG00000130147	0.447791841	1.736692229	0.599589859	1.61962761	-2.827855018	-1.499708156	0.049936722	31	SH3-domain binding protein 4 [Source:HGNC Symbol;Acc:10826]	SH3BP4	23677
ENSG00000170962	2.171308143	4.277109699	2.248576561	1.891035639	-2.508282164	-1.32669965	0.035547604	32	platelet derived growth factor D [Source:HGNC Symbol;Acc:30620]	PDGFD	80310
ENSG00000128268	3.989197896	2.401772372	1.939978799	2.067856779	2.204384174	1.140375675	0.016622321	33	mannosyl (beta-1,4-)-glycoprotein beta-1,4-N-acetylglucosaminyltransferase [Source:HGNC Symbol;Acc:7046]	MGAT3	4248
ENSG00000139197	2.620553034	1.375387813	1.039004008	0.799975855	2.337354758	1.224876719	0.024434421	34	peroxisomal biogenesis factor 5 [Source:HGNC Symbol;Acc:9719]	PEX5	5830
ENSG00000189127	4.078545754	2.174313049	2.382640189	1.95806183	2.436405871	1.284754486	0.037455884	35	ankyrin repeat domain 34B [Source:HGNC Symbol;Acc:33736]	ANKRD34B	340120
ENSG00000129422	7.488096667	2.70819253	7.133374453	1.934868222	2.448415667	1.291848505	0.0422714	36	microtubule associated tumor suppressor 1 [Source:HGNC Symbol;Acc:29789]	MTUS1	57509

ENSG00000158186	2.458807909	5.041624845	1.540938325	2.204024093	-2.219665594	-1.150342342	0.023095874	37	muscle RAS oncogene homolog [Source:HGNC Symbol;Acc:7227]	MRAS	22808
ENSG00000184271	7.675156586	4.523334014	3.397594111	2.767405894	2.18574704	1.128126445	0.024288065	39	POU class 6 homeobox 1 [Source:HGNC Symbol;Acc:9224]	POU6F1	5463
ENSG00000133962	3.286283585	1.799206613	2.543519239	1.992524849	2.468152839	1.303431736	0.046170721	41	catsper channel auxiliary subunit beta [Source:HGNC Symbol;Acc:20500]	CATSPERB	79820
ENSG00000171860	9.863160105	20.80101531	5.561883316	10.98492169	-2.099808321	-1.070257639	0.016430199	42	complement component 3a receptor 1 [Source:HGNC Symbol;Acc:1319]	C3AR1	719
ENSG00000076351	1.108683621	2.443929832	1.151615309	1.560722973	-2.365030868	-1.241859013	0.043411135	46	solute carrier family 46 (folate transporter), member 1 [Source:HGNC Symbol;Acc:30521]	SLC46A1	113235
ENSG00000163449	5.626530989	3.256905292	4.699027652	2.583957973	2.096682283	1.068108262	0.020589179	48	transmembrane protein 169 [Source:HGNC Symbol;Acc:25130]	TMEM169	92691
ENSG00000175352	2.468250518	1.337967688	2.056731458	1.51283599	2.337236888	1.224803964	0.042584988	50	nuclear receptor interacting protein 3 [Source:HGNC Symbol;Acc:1167]	NRIP3	56675
ENSG00000103512	4.076944274	7.117932655	2.86741138	2.064452954	-2.063987574	-1.045434286	0.012751735	51	NODAL modulator 1 [Source:HGNC Symbol;Acc:30060]	NOMO1	101930025
ENSG00000173926	4.172813403	2.664588211	1.816693971	2.276172069	2.164882557	1.114288762	0.036717233	52	membrane-associated ring finger (C3HC4) 3, E3 ubiquitin protein ligase [Source:HGNC Symbol;Acc:28728]	MARCH3	115123
ENSG00000107281	10.94299644	6.153032982	6.082375732	3.967176538	2.032368039	1.023161682	0.012123657	53	neural proliferation, differentiation and control, 1 [Source:HGNC Symbol;Acc:7899]	NPDC1	56654
ENSG00000156172	2.005698053	3.327151806	1.82970384	1.941089897	-2.106952243	-1.075157614	0.03506351	54	chromosome 8 open reading frame 37 [Source:HGNC Symbol;Acc:27232]	C8orf37	157657
ENSG00000100079	26.29773727	50.50396342	18.64186788	32.70161828	-2.104635992	-1.073570733	0.040903917	57	lectin, galactoside-binding, soluble, 2 [Source:HGNC Symbol;Acc:6562]	LGALS2	3957
ENSG00000196812	2.593266215	4.689525864	1.387768276	1.451833941	-2.063253036	-1.044920763	0.026293959	59	zinc finger and SCAN domain containing 16 [Source:HGNC Symbol;Acc:20813]	ZSCAN16	80345
ENSG00000142235	2.789578718	1.669592482	2.105040738	1.240941943	2.142765013	1.099473645	0.0454421	60	lemur tyrosine kinase 3 [Source:HGNC Symbol;Acc:19295]	LMTK3	114783
ENSG00000092208	4.574462051	2.347606943	1.946654527	1.497638328	1.999011956	0.999287101	0.014467484	61	gem (nuclear organelle) associated protein 2 [Source:HGNC Symbol;Acc:10884]	GEMIN2	8487
ENSG00000148468	5.579572828	3.342020581	1.897788055	2.187149371	2.074654494	1.052871095	0.041240342	67	family with sequence similarity 171, member A1 [Source:HGNC Symbol;Acc:23522]	FAM171A1	221061
ENSG00000183742	2.172076662	1.346075406	1.087403722	1.048033808	2.070256414	1.049809465	0.049672692	74	metastasis associated in colon cancer 1 [Source:HGNC Symbol;Acc:30215]	MACC1	346389
ENSG00000174600	17.4544413	32.32144454	11.13325011	14.05132028	-1.911858709	-0.934975908	0.006516431	79	chemokine-like receptor 1 [Source:HGNC Symbol;Acc:2121]	CMKLR1	1240
ENSG00000256618	71.98123109	29.48291133	71.43644125	21.04146687	2.029081655	1.020826924	0.045786603	80	MT-RNR2-like 1 [Source:HGNC Symbol;Acc:37155]	MTRNR2L1	100462977
ENSG00000249992	13.56082542	21.46557572	13.16091873	13.79182613	-1.996383742	-0.99738906	0.041608279	84	transmembrane protein 158 (gene/pseudogene) [Source:HGNC Symbol;Acc:30293]	TMEM158	25907
ENSG00000213949	8.771639563	5.099121366	5.02677374	2.913687236	1.931479959	0.949706709	0.020728946	87	integrin, alpha 1 [Source:HGNC Symbol;Acc:6134]	ITGA1	3672

ENSG00000108439	2.536796129	5.185018938	1.430278407	2.727906882	-1.961672195	-0.97208398	0.031472786	88	pyridoxamine 5'-phosphate oxidase [Source:HGNC Symbol;Acc:30260]	PNPO	55163
ENSG00000141837	8.130518613	4.7518106	4.515224819	3.667484977	1.960632954	0.971319477	0.032588127	92	calcium channel, voltage-dependent, P/Q type, alpha 1A subunit [Source:HGNC Symbol;Acc:1388]	CACNA1A	100507353
ENSG00000081026	6.511463121	3.75403478	4.850554199	2.19893212	1.922005825	0.942612708	0.030321365	98	membrane associated guanylate kinase, WW and PDZ domain containing 3 [Source:HGNC Symbol;Acc:29647]	MAGI3	260425
ENSG00000077585	3.518199228	6.50212368	1.401191378	2.290281319	-1.855479177	-0.89179181	0.013151615	103	G protein-coupled receptor 137B [Source:HGNC Symbol;Acc:11862]	GPR137B	7107
ENSG00000084731	3.645498141	2.41199268	1.65610287	2.106336383	1.912365229	0.93535808	0.045693768	112	kinesin family member 3C [Source:HGNC Symbol;Acc:6321]	KIF3C	3797
ENSG00000137441	44.58356619	84.68540439	25.27332462	40.64669105	-1.828096125	-0.870341932	0.02017647	114	fibroblast growth factor binding protein 2 [Source:HGNC Symbol;Acc:29451]	FGFBP2	83888
ENSG00000171617	5.96322066	11.13542404	2.321567152	6.061715675	-1.848007268	-0.885970431	0.037800803	116	ectodermal-neural cortex 1 (with BTB domain) [Source:HGNC Symbol;Acc:3345]	ENC1	8507
ENSG00000157827	8.142717209	14.03161735	5.110795773	7.208341769	-1.864702763	-0.89894568	0.04359139	118	formin-like 2 [Source:HGNC Symbol;Acc:18267]	FMNL2	114793
ENSG00000182010	61.62908248	34.31725269	37.50830209	14.91587904	1.782914706	0.834237687	0.020672855	125	rhotekin 2 [Source:HGNC Symbol;Acc:19364]	RTKN2	219790
ENSG00000066926	6.094315904	11.23029741	1.624197918	5.438705908	-1.692005284	-0.758734074	0.029435255	154	ferrochelatase [Source:HGNC Symbol;Acc:3647]	FECH	2235
ENSG00000107736	8.674624595	15.03775118	3.385786451	6.359106923	-1.680216763	-0.748647366	0.019971533	156	cadherin-related 23 [Source:HGNC Symbol;Acc:13733]	CDH23	100653137
ENSG00000088038	15.60349948	10.88041463	3.561386983	4.23408763	1.615989311	0.692417655	0.024994934	207	CCR4-NOT transcription complex, subunit 3 [Source:HGNC Symbol;Acc:7879]	CNOT3	4849
ENSG00000121413	20.01480425	13.62904155	9.865858056	8.212178379	1.610521983	0.687528353	0.045394573	218	zinc finger and SCAN domain containing 18 [Source:HGNC Symbol;Acc:21037]	ZSCAN18	65982
ENSG00000103353	6.707707009	10.65918869	3.116910206	2.338600297	-1.607889096	-0.6851679	0.045541886	222	ubiquitin family domain containing 1 [Source:HGNC Symbol;Acc:30565]	UBFD1	56061
ENSG00000170448	30.05287271	18.15052635	20.6556349	7.483524981	1.597349081	0.675679631	0.040851232	225	nuclear transcription factor, X-box binding-like 1 [Source:HGNC Symbol;Acc:18726]	NFXL1	152518
ENSG00000138134	13.96553002	9.556874483	7.112084888	4.686700862	1.568410212	0.649302941	0.042163302	263	STAM binding protein-like 1 [Source:HGNC Symbol;Acc:24105]	STAMBPL1	57559

Compared populations:

D2T RA Low inflammation high pain vs non-D2T RA

Fold change describes the size of the difference in gene expression (given in mean and standar deviation, sd) between the compared groups. The values are always in relation to the group used as a base level group (reference). P-value describes the reliability of the change in expression value between the compared groups. Results were considered significant with a fold change of 1.2 and a p-value cutoff of 0.05.

Supplementary Material 22 - Summary of enrichement analysis of DE genes listing the top 25 processes or pathways based on different databases (Gene Ontology and Reactome)

Gene Ontology Biological Process					
**GO.ID**	**Term**	**Annotated**	**Significant**	**Expected**	**p value**
GO:0002430	complement receptor mediated signaling p...	9	3	0.05	0.000016
GO:0010758	regulation of macrophage chemotaxis	20	3	0.12	0.0002
GO:0071230	cellular response to amino acid stimulus	53	4	0.31	0.00025
GO:0071229	cellular response to acid chemical	59	4	0.35	0.00038
GO:0048246	macrophage chemotaxis	25	3	0.15	0.0004
GO:1905521	regulation of macrophage migration	32	3	0.19	0.00085
GO:0043200	response to amino acid	77	4	0.45	0.00106
GO:0006935	chemotaxis	275	7	1.61	0.00108
GO:0042330	taxis	275	7	1.61	0.00108
GO:1905517	macrophage migration	38	3	0.22	0.0014
GO:0001101	response to acid chemical	84	4	0.49	0.00146
GO:0050920	regulation of chemotaxis	143	5	0.84	0.00148
GO:0097529	myeloid leukocyte migration	149	5	0.87	0.00177
GO:0006835	dicarboxylic acid transport	42	3	0.25	0.00188
GO:0006767	water-soluble vitamin metabolic process	44	3	0.26	0.00215
GO:0050921	positive regulation of chemotaxis	94	4	0.55	0.00221
GO:0002685	regulation of leukocyte migration	157	5	0.92	0.00222
GO:0002687	positive regulation of leukocyte migrati...	102	4	0.6	0.00298
GO:0007200	phospholipase C-activating G protein-cou...	50	3	0.29	0.0031
GO:0050900	leukocyte migration	259	6	1.52	0.00399
GO:0032006	regulation of TOR signaling	120	4	0.7	0.00532
GO:0060326	cell chemotaxis	197	5	1.16	0.00587
GO:0006766	vitamin metabolic process	63	3	0.37	0.00595
GO:0070887	cellular response to chemical stimulus	1697	18	9.96	0.00709
GO:0031929	TOR signaling	140	4	0.82	0.0091

Reactome				
Reactome.ID	Term	GeneRatio	BgRatio	p value
R-HSA-216083	Integrin cell surface interactions	3/37	40/6966	0.001190563
R-HSA-210500	Glutamate Neurotransmitter Release Cycle	2/37	11/6966	0.001465078
R-HSA-3000157	Laminin interactions	2/37	11/6966	0.001465078
R-HSA-75892	Platelet Adhesion to exposed collagen	2/37	12/6966	0.001752219
R-HSA-373076	Class A/1 (Rhodopsin-like receptors)	4/37	113/6966	0.002870085
R-HSA-375276	Peptide ligand-binding receptors	3/37	63/6966	0.004400492
R-HSA-112310	Neurotransmitter release cycle	2/37	22/6966	0.005931269
R-HSA-3000178	ECM proteoglycans	2/37	24/6966	0.00703961
R-HSA-500792	GPCR ligand binding	4/37	151/6966	0.008037449
R-HSA-196849	Metabolism of water-soluble vitamins and cofactors	3/37	90/6966	0.011802638
R-HSA-186797	Signaling by PDGF	2/37	40/6966	0.018863973
R-HSA-372790	Signaling by GPCR	5/37	322/6966	0.026456487
R-HSA-8866654	E3 ubiquitin ligases ubiquitinate target proteins	2/37	48/6966	0.026567047
R-HSA-9013148	CDC42 GTPase cycle	3/37	124/6966	0.027554996
R-HSA-196854	Metabolism of vitamins and cofactors	3/37	125/6966	0.028131057
R-HSA-1474244	Extracellular matrix organization	3/37	126/6966	0.028713575
R-HSA-6806834	Signaling by MET	2/37	52/6966	0.030820324
R-HSA-9816359	Maternal to zygotic transition (MZT)	2/37	53/6966	0.03192339
R-HSA-9013106	RHOC GTPase cycle	2/37	59/6966	0.038861245
R-HSA-9006934	Signaling by Receptor Tyrosine Kinases	5/37	364/6966	0.041743884
R-HSA-418594	G alpha (i) signalling events	3/37	147/6966	0.042426611
R-HSA-8852135	Protein ubiquitination	2/37	67/6966	0.048913162
R-HSA-8851805	MET activates RAS signaling	1/37	10/6966	0.051896134
R-HSA-1474165	Reproduction	2/37	71/6966	0.054257594
R-HSA-373760	L1CAM interactions	2/37	71/6966	0.054257594

Compared populations:

D2T RA Low inflammation high pain vs non-D2T RA

Enrichment analysis for the differentially expressed filtered gene lists tests whether the genes within a certain Reactome pathway or GO term are statistically over-represented in a given comparison. Annotated: number of genes associated with the given term. R: Reactome database. Significant: number of statistically significant associated with the given term. Expected: expected number of genes associated with the given term in the DE gene list. Gene ratio: ratio of number of genes in the DE list that overlap with genes associated with the given term, and the number of genes in the DE list which overlap with genes associated with all terms in the Reactome database. Bg ratio: ratio of the number of genes associated with the given term, and the total number of genes associated with Reactome term

Supplementary Material 23 - List of differentially expressed (DE) genes (sex-adjusted)

ID	mean_RA	mean_Control	sd_RA	sd_Control	FC	logFC	P.Value	avgRank	Description	geneName	EntrezID
ENSG00000244734	3113.03179	1155.37854	3134.30291	2229.79337	5.01716051	2.32687109	3.7975E-06	1	hemoglobin, beta [Source:HGNC Symbol;Acc:4827]	HBB	3043
ENSG00000169877	3.29442262	0.38280226	4.92505856	0.63392344	5.32209258	2.41199361	9.8311E-05	2	alpha hemoglobin stabilizing protein [Source:HGNC Symbol;Acc:18075]	AHSP	51327
ENSG00000206177	6.5250651	1.23144829	9.0767652	1.80420905	5.93509086	2.56927012	0.0002069	3	hemoglobin, mu [Source:HGNC Symbol;Acc:4826]	HBM	3042
ENSG00000144290	5.12471531	12.6637233	6.65849265	16.339091	-3.5820487	-1.8407849	4.9676E-05	4	solute carrier family 4, sodium bicarbonate transporter, member 10 [Source:HGNC Symbol;Acc:13811]	SLC4A10	57282
ENSG00000158578	8.45383713	1.39422151	10.7508797	2.76471307	7.15932878	2.83982434	0.00024201	5	aminolevulinate, delta-, synthase 2 [Source:HGNC Symbol;Acc:397]	ALAS2	212
ENSG00000206172	200.528791	84.7344559	213.959683	131.290964	3.83122024	1.93780396	0.00016731	6	hemoglobin, alpha 1 [Source:HGNC Symbol;Acc:4823]	HBA1	3040
ENSG00000133169	1.70674775	0.35488561	1.63098609	0.55120452	3.76098882	1.91111202	0.0001062	7	brain expressed, X-linked 1 [Source:HGNC Symbol;Acc:1036]	BEX1	55859
ENSG00000223609	7.67352761	1.07968881	11.5423204	1.77616381	5.94956557	2.57278433	0.00068273	8	hemoglobin, delta [Source:HGNC Symbol;Acc:4829]	HBD	3045
ENSG00000143416	2.19441546	0.61392877	2.57841094	1.04208788	3.7878197	1.92136766	0.00049048	9	selenium binding protein 1 [Source:HGNC Symbol;Acc:10719]	SELENBP1	8991
ENSG00000227184	1.3560413	3.26868176	1.78329894	2.37719746	-2.9438871	-1.5577223	0.00013954	10	epiplakin 1 [Source:HGNC Symbol;Acc:15577]	EPPK1	83481
ENSG00000188536	725.398478	370.226899	709.489742	623.36819	3.51216474	1.81236052	0.0002171	11	hemoglobin, alpha 2 [Source:HGNC Symbol;Acc:4824]	HBA2	3040
ENSG00000072195	0.84592934	1.77279893	1.02687854	1.08506981	-2.7556086	-1.462371	0.00021652	12	SPEG complex locus [Source:HGNC Symbol;Acc:16901]	SPEG	100996693
ENSG00000134668	3.49283645	1.16141706	3.05440923	1.17629249	3.28566159	1.7161839	0.00072165	13	SPOC domain containing 1 [Source:HGNC Symbol;Acc:26338]	SPOCD1	90853
ENSG00000047597	5.49765327	2.17170901	5.305473	2.47853265	2.76079073	1.46508153	0.00057792	14	X-linked Kx blood group (McLeod syndrome) [Source:HGNC Symbol;Acc:12811]	XK	7504
ENSG00000198829	5.21510252	1.93308799	5.01707692	1.67530504	2.75113099	1.46002483	0.0005769	15	succinate receptor 1 [Source:HGNC Symbol;Acc:4542]	SUCNR1	56670
ENSG00000109674	4.48030835	2.01492106	2.98626945	1.22693646	2.50953052	1.32741749	0.00053739	16	nei endonuclease VIII-like 3 (E. coli) [Source:HGNC Symbol;Acc:24573]	NEIL3	55247
ENSG00000148498	6.00170575	2.81407373	4.02885418	2.22724482	2.54986678	1.35042188	0.00130418	17	par-3 family cell polarity regulator [Source:HGNC Symbol;Acc:16051]	PARD3	56288
ENSG00000114405	2.25362687	1.29647683	1.72704475	1.56660677	2.63768702	1.39927339	0.0023049	18	chromosome 3 open reading frame 14 [Source:HGNC Symbol;Acc:25024]	C3orf14	57415
ENSG00000072422	12.542174	6.64826572	8.25325374	5.6561545	2.50239659	1.32331045	0.00135985	19	Rho-related BTB domain containing 1 [Source:HGNC Symbol;Acc:18738]	RHOBTB1	9886
ENSG00000172159	27.6964057	15.0683043	14.1607613	9.83920651	2.15615142	1.1084585	6.0901E-05	20	FERM domain containing 3 [Source:HGNC Symbol;Acc:24125]	FRMD3	257019
ENSG00000153162	3.46438972	1.47847307	2.78040228	1.27450465	2.55874001	1.35543356	0.00308901	21	bone morphogenetic protein 6 [Source:HGNC Symbol;Acc:1073]	BMP6	654
ENSG00000136425	1.0251374	1.82072473	1.36378952	1.70073381	-2.4061459	-1.2667241	0.00280997	22	calcium and integrin binding family member 2 [Source:HGNC Symbol;Acc:24579]	CIB2	10518

ENSG00000132970	3.78442297	1.80640219	3.61353032	2.01607679	2.57428659	1.36417268	0.00385825	23	WAS protein family, member 3 [Source:HGNC Symbol;Acc:12734]	WASF3	10810
ENSG00000102010	3.52485584	1.73630081	3.48124582	2.29650182	2.94694459	1.55921994	0.00639239	24	BMX non-receptor tyrosine kinase [Source:HGNC Symbol;Acc:1079]	BMX	660
ENSG00000147650	6.02068516	3.18953792	3.39136417	2.220231	2.20007441	1.13755232	0.0018642	25	low density lipoprotein receptor-related protein 12 [Source:HGNC Symbol;Acc:31708]	LRP12	29967
ENSG00000171241	3.94329055	2.12866434	2.25859385	1.80038289	2.2416345	1.16455106	0.0023524	26	SHC SH2-domain binding protein 1 [Source:HGNC Symbol;Acc:29547]	SHCBP1	79801
ENSG00000136231	13.4577281	6.84541599	8.60037442	4.56307794	2.13880948	1.09680798	0.00113704	27	insulin-like growth factor 2 mRNA binding protein 3 [Source:HGNC Symbol;Acc:28868]	IGF2BP3	10643
ENSG00000062524	1.47769246	3.66819787	1.57525055	3.35933968	-2.2596608	-1.1761062	0.00415633	28	leukocyte receptor tyrosine kinase [Source:HGNC Symbol;Acc:6721]	LTK	4058
ENSG00000180190	2.04677454	1.25558605	1.82823075	1.36125879	2.31204288	1.20916815	0.00526182	29	testis development related protein [Source:HGNC Symbol;Acc:26951]	TDRP	157695
ENSG00000088053	9.30664507	4.95428782	7.0952495	4.32648309	2.24666768	1.16778674	0.00433689	30	glycoprotein VI (platelet) [Source:HGNC Symbol;Acc:14388]	GP6	51206
ENSG00000105289	1.4655656	2.51117387	1.57326287	1.67107638	-2.1897953	-1.130796	0.00376901	31	tight junction protein 3 [Source:HGNC Symbol;Acc:11829]	TJP3	27134
ENSG00000144645	13.0867554	19.7749679	10.6864099	8.15709664	-2.2423539	-1.165014	0.00515149	32	oxysterol binding protein-like 10 [Source:HGNC Symbol;Acc:16395]	OSBPL10	114884
ENSG00000267673	0.90498094	1.73376418	1.08117289	1.42638188	-2.2666006	-1.1805302	0.00617922	33	ferredoxin 1-like [Source:HGNC Symbol;Acc:30546]	FDX1L	112812
ENSG00000115423	1.41502566	2.20643355	1.62439743	1.84157281	-2.2421489	-1.1648821	0.00721693	34	dynein, axonemal, heavy chain 6 [Source:HGNC Symbol;Acc:2951]	DNAH6	1768
ENSG00000103740	1.79544184	0.68805835	1.69642377	0.79268068	2.33978789	1.22637775	0.0140821	35	acyl-CoA synthetase bubblegum family member 1 [Source:HGNC Symbol;Acc:29567]	ACSBG1	23205
ENSG00000184792	3.38993579	1.06349579	3.75204388	1.15122179	2.4806261	1.3107043	0.01533684	36	oxysterol binding protein 2 [Source:HGNC Symbol;Acc:8504]	OSBP2	23762
ENSG00000205639	2.82323527	1.32652024	2.69970325	1.2783593	2.27154204	1.18367201	0.01409244	37	major facilitator superfamily domain containing 2B [Source:HGNC Symbol;Acc:37207]	MFSD2B	388931
ENSG00000100336	1.73380036	0.45537549	1.98760385	0.58452344	2.31746852	1.21254974	0.01479712	38	apolipoprotein L, 4 [Source:HGNC Symbol;Acc:14867]	APOL4	80832
ENSG00000176945	3.9972019	7.09247189	3.28767838	4.80353315	-2.1539653	-1.106995	0.01186563	39	mucin 20, cell surface associated [Source:HGNC Symbol;Acc:23282]	MUC20	200958
ENSG00000102362	10.2153395	6.03921723	6.97525721	4.45481336	2.03797853	1.02713885	0.00694662	40	synaptotagmin-like 4 [Source:HGNC Symbol;Acc:15588]	SYTL4	94121
ENSG00000137807	2.16494209	1.47873518	1.27621955	1.25016221	2.06357384	1.04514506	0.00768216	41	kinesin family member 23 [Source:HGNC Symbol;Acc:6392]	KIF23	9493
ENSG00000118113	4.07582966	1.01699309	7.40511204	1.18748809	2.67706044	1.42064971	0.01730818	42	matrix metallopeptidase 8 (neutrophil collagenase) [Source:HGNC Symbol;Acc:7175]	MMP8	4317
ENSG00000269636	7.13421318	3.21788126	7.27489887	3.20646761	2.41414796	1.2715141	0.01701373	43		AC010441.1	
ENSG00000155755	1.82198222	3.16327296	1.41273653	1.8717985	-1.97273	-0.9801935	0.00531598	44	transmembrane protein 237 [Source:HGNC Symbol;Acc:14432]	TMEM237	65062
ENSG00000163462	1.13435489	1.82346664	1.31872845	1.44255459	-2.0957411	-1.0674605	0.01297619	45	tripartite motif containing 46 [Source:HGNC Symbol;Acc:19019]	TRIM46	80128

ENSG00000196466	5.37841193	3.5979144	3.41130267	2.9440097	2.00106749	1.00076983	0.01051968	46	zinc finger protein 799 [Source:HGNC Symbol;Acc:28071]	ZNF799	90576
ENSG00000003436	5.87620917	3.03616628	4.95545679	3.55208305	2.18895739	1.13024387	0.01611064	47	tissue factor pathway inhibitor (lipoprotein-associated coagulation inhibitor) [Source:HGNC Symbol;Acc:11760]	TFPI	7035
ENSG00000167633	2.27170079	4.13324238	2.8038383	3.02006715	-2.0998518	-1.0702875	0.01491333	48	killer cell immunoglobulin-like receptor, three domains, long cytoplasmic tail, 1 [Source:HGNC Symbol;Acc:6338]	KIR3DL1	3812
ENSG00000070371	2.7712781	1.68352126	1.46535048	1.33144927	2.05057163	1.03602614	0.0131603	49	clathrin, heavy chain-like 1 [Source:HGNC Symbol;Acc:2093]	CLTCL1	8218
ENSG00000162882	1.82494298	1.63925161	1.38850284	2.19176912	2.13743095	1.09587782	0.01682083	50	3-hydroxyanthranilate 3,4-dioxygenase [Source:HGNC Symbol;Acc:4796]	HAAO	23498
ENSG00000198934	4.95449136	7.71111476	3.08435207	3.71575058	-1.8530663	-0.8899145	0.00289971	51	melanoma antigen family E, 1 [Source:HGNC Symbol;Acc:24934]	MAGEE1	57692
ENSG00000204165	3.32720556	5.88153269	2.16747916	2.93570524	-1.8551767	-0.8915566	0.00480328	52	chromosome X open reading frame 65 [Source:HGNC Symbol;Acc:33713]	CXorf65	158830
ENSG00000177324	13.4411641	6.870657	10.6404179	5.37797698	2.03157588	1.02259925	0.01614952	53	BEN domain containing 2 [Source:HGNC Symbol;Acc:28509]	BEND2	139105
ENSG00000038945	12.7644894	6.59514692	9.37202769	3.50485783	1.93849608	0.95493781	0.01243178	54	macrophage scavenger receptor 1 [Source:HGNC Symbol;Acc:7376]	MSR1	4481
ENSG00000115318	4.38626195	2.63980129	2.81637566	2.07528107	1.97654454	0.98298047	0.01536076	55	lysyl oxidase-like 3 [Source:HGNC Symbol;Acc:13869]	LOXL3	84695
ENSG00000137757	22.2165142	10.6902111	19.4789426	7.22443386	1.93699749	0.95382209	0.01281676	56	caspase 5, apoptosis-related cysteine peptidase [Source:HGNC Symbol;Acc:1506]	CASP5	838
ENSG00000145335	21.8966721	9.44969852	18.8822267	6.0576883	1.98653811	0.99025647	0.0156665	57	synuclein, alpha (non A4 component of amyloid precursor) [Source:HGNC Symbol;Acc:11138]	SNCA	6622
ENSG00000065534	28.5674708	17.702701	16.9221661	13.4831022	1.84310439	0.88213778	0.00740049	58	myosin light chain kinase [Source:HGNC Symbol;Acc:7590]	MYLK	4638
ENSG00000167747	2.30563802	3.24346137	2.66713527	1.81735058	-1.9500429	-0.9635058	0.0155397	59	chromosome 19 open reading frame 48 [Source:HGNC Symbol;Acc:29667]	C19orf48	692204
ENSG00000112290	3.09990552	1.76431087	2.49525756	1.45704018	2.02144304	1.01538556	0.01770946	60	WAS protein family, member 1 [Source:HGNC Symbol;Acc:12732]	WASF1	8936
ENSG00000213782	2.09934836	4.03196829	1.85896841	3.52441715	-1.9631271	-0.9731536	0.01628293	61	DEAD (Asp-Glu-Ala-Asp) box polypeptide 47 [Source:HGNC Symbol;Acc:18682]	DDX47	51202
ENSG00000142173	6.76108091	13.8371288	4.93226688	8.73062204	-1.8169985	-0.8615573	0.00401485	62	collagen, type VI, alpha 2 [Source:HGNC Symbol;Acc:2212]	COL6A2	1292
ENSG00000091129	0.92231087	2.28095957	1.1779693	2.41489514	-2.1131796	-1.0794154	0.02212092	63	neuronal cell adhesion molecule [Source:HGNC Symbol;Acc:7994]	NRCAM	4897
ENSG00000166278	2.55247475	1.07133242	2.95853467	1.0928014	2.24992703	1.16987822	0.02727802	64	complement component 2 [Source:HGNC Symbol;Acc:1248]	C2	717
ENSG00000187808	9.14115213	6.64283071	3.570676	4.74125179	1.77448379	0.82739939	0.00223364	65	sosondowah ankyrin repeat domain family member D [Source:HGNC Symbol;Acc:32960]	SOWAHD	347454
ENSG00000162591	2.91857402	5.24050842	2.26723653	3.77015034	-1.8303238	-0.8720989	0.0110002	66	multiple EGF-like-domains 6 [Source:HGNC Symbol;Acc:3232]	MEGF6	1953
ENSG00000123610	17.2357605	10.4075998	16.2506595	11.8554049	2.01273546	1.00915757	0.02038929	67	tumor necrosis factor, alpha-induced protein 6 [Source:HGNC Symbol;Acc:11898]	TNFAIP6	7130
ENSG00000128512	12.7026794	6.93932878	9.0419921	5.53941973	1.91424141	0.93677279	0.01557997	68	dedicator of cytokinesis 4 [Source:HGNC Symbol;Acc:19192]	DOCK4	9732

ENSG00000008300	1.3153614	2.03611599	1.51291905	1.52084379	-1.9157765	-0.9379293	0.01565496	69	cadherin, EGF LAG seven-pass G-type receptor 3 [Source:HGNC Symbol;Acc:3230]	CELSR3	1951
ENSG00000137198	6.29983093	2.72927674	7.5684277	2.51460744	2.16614402	1.11512917	0.02794371	70	guanosine monophosphate reductase [Source:HGNC Symbol;Acc:4376]	GMPR	2766
ENSG00000073536	1.91190807	3.38633143	1.48944538	2.05356711	-1.8286563	-0.8707839	0.01215102	71	notchless homolog 1 (Drosophila) [Source:HGNC Symbol;Acc:19889]	NLE1	54475
ENSG00000140022	7.54117584	4.23916814	3.79637432	2.28796943	1.77075054	0.82436099	0.00310859	72	stonin 2 [Source:HGNC Symbol;Acc:30652]	STON2	85439
ENSG00000126861	3.51453347	1.93137106	2.63668556	2.53111423	2.10931734	1.07677616	0.02570245	73	oligodendrocyte myelin glycoprotein [Source:HGNC Symbol;Acc:8135]	OMG	101927057
ENSG00000145428	1.43255279	2.8861394	2.16308335	3.04051357	-2.140933	-1.0982397	0.02833519	74	ring finger protein 175 [Source:HGNC Symbol;Acc:27735]	RNF175	285533
ENSG00000112299	15.3544865	10.6677606	8.89489694	9.09304534	1.82409743	0.86718279	0.01227588	75	vanin 1 [Source:HGNC Symbol;Acc:12705]	VNN1	8876
ENSG00000133069	5.24665369	2.89221658	4.25800069	1.75931677	1.8837411	0.91360069	0.01627045	76	transmembrane and coiled-coil domain family 2 [Source:HGNC Symbol;Acc:24239]	TMCC2	9911
ENSG00000249992	19.0075731	9.70998216	13.682446	5.98482611	1.82951747	0.87146319	0.01439007	77	transmembrane protein 158 (gene/pseudogene) [Source:HGNC Symbol;Acc:30293]	TMEM158	25907
ENSG00000159231	1.92551451	3.65823331	1.70530769	3.19143214	-1.9993878	-0.9995583	0.0239565	78	carbonyl reductase 3 [Source:HGNC Symbol;Acc:1549]	CBR3	874
ENSG00000171914	1.54118276	0.99822059	1.08898731	1.15297148	1.95059867	0.96391698	0.02093103	79	talin 2 [Source:HGNC Symbol;Acc:15447]	TLN2	83660
ENSG00000119862	21.8185771	11.9427089	13.5060562	5.58457664	1.78871397	0.83892271	0.00788646	80	lectin, galactoside-binding-like [Source:HGNC Symbol;Acc:25012]	LGALSL	29094
ENSG00000149131	14.3435282	6.05473362	24.3459207	7.9749508	2.2875656	1.19381312	0.03245421	81	serpin peptidase inhibitor, clade G (C1 inhibitor), member 1 [Source:HGNC Symbol;Acc:1228]	SERPING1	710
ENSG00000180914	3.12480512	1.64032312	2.70478959	1.43282697	2.01278	1.00918949	0.02609722	82	oxytocin receptor [Source:HGNC Symbol;Acc:8529]	OXTR	5021
ENSG00000123095	2.55320124	3.30409383	2.87403413	2.56240833	-1.9931555	-0.9950542	0.02510467	83	basic helix-loop-helix family, member e41 [Source:HGNC Symbol;Acc:16617]	BHLHE41	79365
ENSG00000078401	1.80693066	3.65299906	2.57037614	4.92893231	-1.9627016	-0.9728409	0.02330311	84	endothelin 1 [Source:HGNC Symbol;Acc:3176]	EDN1	1906
ENSG00000183762	12.4210595	6.25956642	12.0967908	6.73891384	1.99889635	0.99920366	0.0263713	85	kringle containing transmembrane protein 1 [Source:HGNC Symbol;Acc:17550]	KREMEN1	83999
ENSG00000171817	1.91134523	3.10513623	1.65195459	2.26548674	-1.834935	-0.875729	0.01626744	86	zinc finger protein 540 [Source:HGNC Symbol;Acc:25331]	ZNF540	163255
ENSG00000198003	2.1464293	1.42052818	1.87514328	1.6009123	2.07318731	1.05185047	0.03076847	87	coiled-coil domain containing 151 [Source:HGNC Symbol;Acc:28303]	CCDC151	115948
ENSG00000123700	27.7479231	15.1270763	22.9459777	13.7197523	2.09438476	1.0665265	0.03154102	88	potassium inwardly-rectifying channel, subfamily J, member 2 [Source:HGNC Symbol;Acc:6263]	KCNJ2	3759
ENSG00000099256	2.00847286	0.99433086	1.91142435	1.21145427	2.15226532	1.10585594	0.0331612	89	phosphoribosyl transferase domain containing 1 [Source:HGNC Symbol;Acc:23333]	PRTFDC1	56952
ENSG00000096006	7.77566925	3.269998	10.2478732	2.41805878	2.11981905	1.08394112	0.03260993	90	cysteine-rich secretory protein 3 [Source:HGNC Symbol;Acc:16904]	CRISP3	10321

ENSG00000124145	2.52989012	1.79684796	1.53432259	1.41016507	1.86136176	0.89635847	0.02068265	91	syndecan 4 [Source:HGNC Symbol;Acc:10661]	SDC4	6385
ENSG00000109684	2.0484579	3.11702537	1.50278053	1.77189658	-1.7867997	-0.8373779	0.01462872	92	cytokine-dependent hematopoietic cell linker [Source:HGNC Symbol;Acc:17438]	CLNK	116449
ENSG00000082196	1.42617631	2.2556547	1.18354675	1.42744948	-1.8895978	-0.9180792	0.02211808	93	C1q and tumor necrosis factor related protein 3 [Source:HGNC Symbol;Acc:14326]	C1QTNF3	114899
ENSG00000113368	79.9037564	51.7079558	36.8973184	20.3330311	1.68747725	0.75486805	0.00117774	94	lamin B1 [Source:HGNC Symbol;Acc:6637]	LMNB1	4001
ENSG00000150672	1.68977019	3.02610937	1.79086851	2.29957587	-1.9218261	-0.9424778	0.02536897	95	discs, large homolog 2 (Drosophila) [Source:HGNC Symbol;Acc:2901]	DLG2	1740
ENSG00000197933	1.48579155	2.0470787	1.41660507	1.25904338	-1.84643	-0.8847385	0.02093677	96	zinc finger protein 823 [Source:HGNC Symbol;Acc:30936]	ZNF823	55552
ENSG00000189013	1.63892636	0.87066755	2.00220125	1.13794464	2.16482264	1.11424884	0.03702377	97	killer cell immunoglobulin-like receptor, two domains, long cytoplasmic tail, 4 [Source:HGNC Symbol;Acc:6332]	KIR2DL4	3805
ENSG00000107317	8.31341339	16.4776172	7.24971888	11.4549206	-1.8048001	-0.8518391	0.01679283	98	prostaglandin D2 synthase 21kDa (brain) [Source:HGNC Symbol;Acc:9592]	PTGDS	5730
ENSG00000188282	2.69843487	1.53670352	2.37599848	1.24456337	1.98941824	0.99234661	0.03031851	99	RUN and FYVE domain containing 4 [Source:HGNC Symbol;Acc:24804]	RUFY4	285180
ENSG00000091106	78.6265559	52.0815209	33.7977166	23.0992511	1.68265512	0.75073951	0.00095921	100	NLR family, CARD domain containing 4 [Source:HGNC Symbol;Acc:16412]	NLRC4	58484
ENSG00000137098	2.10047441	3.55431904	1.94892974	2.430343	-1.9018442	-0.9273991	0.02663702	101	sperm associated antigen 8 [Source:HGNC Symbol;Acc:14105]	SPAG8	26206
ENSG00000055732	2.49339573	1.07271642	2.10745067	0.93371837	1.96573832	0.97507128	0.03199198	102	mucolipin 3 [Source:HGNC Symbol;Acc:13358]	MCOLN3	55283
ENSG00000088726	12.7541262	6.91799916	9.97532679	4.79798429	1.80200857	0.84960587	0.01995973	103	transmembrane protein 40 [Source:HGNC Symbol;Acc:25620]	TMEM40	55287
ENSG00000213934	2.18944235	0.82902823	3.04725417	1.315702	2.2433096	1.16562874	0.04221554	104	hemoglobin, gamma A [Source:HGNC Symbol;Acc:4831]	HBG1	3047
ENSG00000145685	32.2219176	19.8079182	17.5066573	8.26688737	1.67941695	0.74796046	0.00343909	105	lipoma HMGIC fusion partner-like 2 [Source:HGNC Symbol;Acc:6588]	LHFPL2	10184
ENSG00000184113	9.53744034	4.83878876	8.05890437	4.37219209	1.94612278	0.96060273	0.03085997	106	claudin 5 [Source:HGNC Symbol;Acc:2047]	CLDN5	7122
ENSG00000170425	1.55948078	0.70650048	1.65938024	0.80625752	2.01364691	1.00981073	0.03644174	107	adenosine A2b receptor [Source:HGNC Symbol;Acc:264]	ADORA2B	136
ENSG00000162367	37.9275677	22.1503069	25.7673792	15.6393445	1.80082577	0.84865861	0.02034976	108	T-cell acute lymphocytic leukemia 1 [Source:HGNC Symbol;Acc:11556]	TAL1	6886
ENSG00000110400	5.14681701	3.5821801	3.0150085	2.50801004	1.76883988	0.82280346	0.01694086	109	poliovirus receptor-related 1 (herpesvirus entry mediator C) [Source:HGNC Symbol;Acc:9706]	PVRL1	5818
ENSG00000134827	9.28189995	4.86521236	8.35599563	3.7353379	1.83742644	0.87768649	0.0263487	110	transcobalamin I (vitamin B12 binding protein, R binder family) [Source:HGNC Symbol;Acc:11652]	TCN1	6947
ENSG00000243452	1.59284382	2.72615528	1.84422755	1.75231532	-1.8612006	-0.8962335	0.02746116	111	neuroblastoma breakpoint family, member 15 [Source:HGNC Symbol;Acc:28791]	NBPF15	284565
ENSG00000166428	2.41293318	4.40417469	2.1968438	2.91887033	-1.8192662	-0.8633567	0.02285297	112	phospholipase D family, member 4 [Source:HGNC Symbol;Acc:23792]	PLD4	122618
ENSG00000143878	43.3131398	30.769088	25.4154114	24.0912416	1.74295626	0.80153636	0.01538447	113	ras homolog family member B [Source:HGNC Symbol;Acc:668]	RHOB	388

ENSG00000135378	44.7052617	27.8635055	25.198965	15.1915541	1.68898186	0.75615383	0.00985709	114	proline rich Gla (G-carboxyglutamic acid) 4 (transmembrane) [Source:HGNC Symbol;Acc:30799]	PRRG4	79056
ENSG00000080493	4.06929689	7.18019559	2.48186855	4.06565712	-1.7626699	-0.8177624	0.0172226	115	solute carrier family 4 (sodium bicarbonate cotransporter), member 4 [Source:HGNC Symbol;Acc:11030]	SLC4A4	8671
ENSG00000143185	2.13690152	3.26662359	2.32688735	2.95862549	-1.9483244	-0.9622339	0.03292622	116	chemokine (C motif) ligand 2 [Source:HGNC Symbol;Acc:10646]	XCL2	6846
ENSG00000108387	2.23136421	1.21594839	1.99300109	0.99723127	1.99609794	0.99718251	0.03740417	117	septin 4 [Source:HGNC Symbol;Acc:9165]	SEPT4	5414
ENSG00000163126	1.12978214	1.60811377	1.60560645	1.27054025	-1.9474683	-0.9615998	0.03357993	118	ankyrin repeat domain 23 [Source:HGNC Symbol;Acc:24470]	ANKRD23	200539
ENSG00000174080	3.93665687	6.63958476	2.09919283	2.8632222	-1.6685748	-0.7386163	0.00442852	119	cathepsin F [Source:HGNC Symbol;Acc:2531]	CTSF	8722
ENSG00000173372	8.4033181	5.4144398	7.5888409	4.10749714	1.83050611	0.87224259	0.02862333	120	complement component 1, q subcomponent, A chain [Source:HGNC Symbol;Acc:1241]	C1QA	712
ENSG00000090920	2.96575181	5.25649658	2.60641236	3.97445754	-1.8654047	-0.8994886	0.03077293	121	Fc fragment of IgG binding protein [Source:HGNC Symbol;Acc:13572]	FCGBP	8857
ENSG00000100678	1.938896	0.58174805	2.4655947	0.71856766	2.11339208	1.07956044	0.04429267	122	solute carrier family 8 (sodium/calcium exchanger), member 3 [Source:HGNC Symbol;Acc:11070]	SLC8A3	6547
ENSG00000115289	4.66354987	3.1066065	2.38458603	1.52081505	1.67875174	0.74738889	0.00848388	123	polycomb group ring finger 1 [Source:HGNC Symbol;Acc:17615]	PCGF1	84759
ENSG00000163430	7.85090187	4.72758536	9.85284107	8.03721761	2.12664361	1.08857828	0.04524979	124	follistatin-like 1 [Source:HGNC Symbol;Acc:3972]	FSTL1	11167
ENSG00000143297	21.4176085	29.4039465	15.4684646	21.9547394	-1.986664	-0.9903479	0.03938882	125	Fc receptor-like 5 [Source:HGNC Symbol;Acc:18508]	FCRL5	83416
ENSG00000183960	1.115262	2.01588576	1.31912832	2.14029222	-1.9613293	-0.9718318	0.03845742	126	potassium voltage-gated channel, subfamily H (eag-related), member 8 [Source:HGNC Symbol;Acc:18864]	KCNH8	131096
ENSG00000187951	4.22195295	3.04741886	2.05482831	1.80930687	1.74773235	0.80548426	0.01891903	127	Rho GTPase activating protein 11B [Source:HGNC Symbol;Acc:15782]	ARHGAP11B	100288637
ENSG00000123405	86.8080975	56.2310342	41.3155318	28.0611892	1.65516833	0.72697794	0.00527592	128	nuclear factor, erythroid 2 [Source:HGNC Symbol;Acc:7780]	NFE2	4778
ENSG00000173210	10.4231144	5.83777273	8.81008413	4.43850597	1.81322574	0.85855855	0.0289311	129	actin binding LIM protein family, member 3 [Source:HGNC Symbol;Acc:29132]	ABLIM3	22885
ENSG00000034053	24.5564809	36.516268	11.844073	12.0799498	-1.6319533	-0.7065997	0.0015552	130	amyloid beta (A4) precursor protein-binding, family A, member 2 [Source:HGNC Symbol;Acc:579]	APBA2	321
ENSG00000127920	124.65307	71.3485208	94.077821	54.0391419	1.75994533	0.81553061	0.0244881	131	guanine nucleotide binding protein (G protein), gamma 11 [Source:HGNC Symbol;Acc:4403]	GNG11	2791
ENSG00000185245	15.2365901	9.39822736	10.5834256	6.86176146	1.76901542	0.82294663	0.02731473	132	glycoprotein Ib (platelet), alpha polypeptide [Source:HGNC Symbol;Acc:4439]	GP1BA	2811
ENSG00000214595	4.34853398	5.52231297	4.31797993	2.45002887	-1.8194112	-0.8634716	0.0321686	133	echinoderm microtubule associated protein like 6 [Source:HGNC Symbol;Acc:35412]	EML6	400954
ENSG00000181631	170.286503	116.808552	71.8028608	53.8554781	1.62743562	0.70260048	0.00344765	134	purinergic receptor P2Y, G-protein coupled, 13 [Source:HGNC Symbol;Acc:4537]	P2RY13	53829
ENSG00000110104	4.55496269	7.54762303	3.05387595	3.41557414	-1.6580246	-0.7294654	0.01269098	135	coiled-coil domain containing 86 [Source:HGNC Symbol;Acc:28359]	CCDC86	79080

ENSG00000204475	4.62137566	8.535486	3.25727996	5.39701742	-1.6728683	-0.7423239	0.01537666	136	natural cytotoxicity triggering receptor 3 [Source:HGNC Symbol;Acc:19077]	NCR3	259197
ENSG00000064763	13.7487403	9.37944599	6.6534825	4.566373	1.64753785	0.72031161	0.01004771	137	fatty acyl CoA reductase 2 [Source:HGNC Symbol;Acc:25531]	FAR2	55711
ENSG00000073737	9.8293491	7.05726121	5.87189558	4.79818841	1.69641233	0.76248687	0.0192397	138	dehydrogenase/reductase (SDR family) member 9 [Source:HGNC Symbol;Acc:16888]	DHRS9	10170
ENSG00000106686	1.51068992	2.44937012	1.48142973	1.7824687	-1.8522763	-0.8892993	0.03817752	139	spermatogenesis associated 6-like [Source:HGNC Symbol;Acc:25472]	SPATA6L	55064
ENSG00000108576	2.05275536	1.15299498	1.96329757	1.89122518	2.03319604	1.02374933	0.04920239	140	solute carrier family 6 (neurotransmitter transporter), member 4 [Source:HGNC Symbol;Acc:11050]	SLC6A4	6532
ENSG00000139610	1.0737529	1.5851988	1.32401851	1.10482822	-1.8084185	-0.8547286	0.03300591	142	chymotrypsin-like elastase family, member 1 [Source:HGNC Symbol;Acc:3308]	CELA1	1990
ENSG00000198692	15.0468429	22.3554572	45.8898259	34.3020802	1.71921224	0.78174766	0.02361372	143	eukaryotic translation initiation factor 1A, Y-linked [Source:HGNC Symbol;Acc:3252]	EIF1AY	101060318
ENSG00000136869	103.326637	68.4058767	45.1106063	22.1048365	1.61234621	0.68916156	0.00197377	144	toll-like receptor 4 [Source:HGNC Symbol;Acc:11850]	TLR4	7099
ENSG00000120896	3.90736698	5.93872342	2.53834314	2.15100887	-1.6546813	-0.7265533	0.01526226	145	sorbin and SH3 domain containing 3 [Source:HGNC Symbol;Acc:30907]	SORBS3	10174
ENSG00000101162	348.777478	206.5292	230.012529	148.52903	1.70275744	0.76787293	0.0232357	146	tubulin, beta 1 class VI [Source:HGNC Symbol;Acc:16257]	TUBB1	81027
ENSG00000005961	46.1219609	26.7943422	37.2700314	17.2981723	1.75206656	0.80905759	0.02882336	147	integrin, alpha 2b (platelet glycoprotein IIb of IIb/IIIa complex, antigen CD41) [Source:HGNC Symbol;Acc:6138]	ITGA2B	3674
ENSG00000162545	3.8285004	5.79843816	2.93048019	2.9371641	-1.725632	-0.7871248	0.02715087	148	calcium/calmodulin-dependent protein kinase II inhibitor 1 [Source:HGNC Symbol;Acc:24190]	CAMK2N1	55450
ENSG00000185222	5.29141759	2.88216129	3.8381879	2.18763797	1.82920649	0.87121794	0.03928858	149	WW domain binding protein 5 [Source:HGNC Symbol;Acc:30084]	WBP5	51186
ENSG00000150681	177.124698	121.791	75.1291885	69.7256025	1.61336253	0.69007066	0.00580638	150	regulator of G-protein signaling 18 [Source:HGNC Symbol;Acc:14261]	RGS18	64407
ENSG00000141736	4.79392602	8.53961103	3.245201	5.12298671	-1.670889	-0.7406159	0.0203032	151	v-erb-b2 avian erythroblastic leukemia viral oncogene homolog 2 [Source:HGNC Symbol;Acc:3430]	ERBB2	2064
ENSG00000174482	0.94616509	1.9537929	1.16319907	1.88185893	-1.9069883	-0.931296	0.04811359	152	leucine rich repeat and Ig domain containing 2 [Source:HGNC Symbol;Acc:21207]	LINGO2	158038
ENSG00000079308	2.66064559	1.5296473	2.28969297	1.65857867	1.94766494	0.96174551	0.04981399	153	tensin 1 [Source:HGNC Symbol;Acc:11973]	TNS1	7145
ENSG00000085733	24.6639946	15.3132271	18.2441503	14.7622085	1.76945831	0.82330777	0.03505011	154	cortactin [Source:HGNC Symbol;Acc:3338]	CTTN	2017
ENSG00000204301	1.39103304	2.30680649	1.36872833	1.36271882	-1.771407	-0.8248957	0.03644543	155	notch 4 [Source:HGNC Symbol;Acc:7884]	NOTCH4	4855
ENSG00000137124	2.30350179	3.99484397	1.55594756	2.29573476	-1.6973416	-0.7632769	0.02745156	156	aldehyde dehydrogenase 1 family, member B1 [Source:HGNC Symbol;Acc:407]	ALDH1B1	219
ENSG00000143995	7.1430144	3.94510835	5.86138497	2.98238189	1.83595209	0.87652841	0.04712217	159	Meis homeobox 1 [Source:HGNC Symbol;Acc:7000]	MEIS1	4211

ENSG00000273173	1.27883804	2.07593892	1.13321655	1.54236997	-1.8009926	-0.8487922	0.04085306	160	SNRPN upstream reading frame protein [Source:UniProtKB/Swiss-Prot;Acc:Q9Y675]	SNURF	8926
ENSG00000117115	59.3688808	41.0268596	38.2164856	28.8705671	1.67496824	0.74413374	0.02400245	161	peptidyl arginine deiminase, type II [Source:HGNC Symbol;Acc:18341]	PADI2	11240
ENSG00000266967	2.43186901	3.50904551	2.30756414	2.29276099	-1.7717401	-0.825167	0.03882425	163	alanyl-tRNA synthetase domain containing 1 [Source:HGNC Symbol;Acc:28417]	AARSD1	80755
ENSG00000166313	8.98916117	14.0663714	5.06718211	7.53593437	-1.5925585	-0.6713464	0.00535647	164	amyloid beta (A4) precursor protein-binding, family B, member 1 (Fe65) [Source:HGNC Symbol;Acc:581]	APBB1	322
ENSG00000259207	20.162006	12.1093108	16.6351514	8.89944599	1.74785491	0.80558543	0.03533714	165	integrin, beta 3 (platelet glycoprotein IIIa, antigen CD61) [Source:HGNC Symbol;Acc:6156]	ITGB3	3690
ENSG00000204420	68.534284	39.0413615	53.9487416	27.0505283	1.72649572	0.78784676	0.03337553	166	chromosome 6 open reading frame 25 [Source:HGNC Symbol;Acc:13937]	C6orf25	80739
ENSG00000170545	3.76658736	5.87356621	3.07702817	3.34710782	-1.6380293	-0.7119612	0.01760987	167	small cell adhesion glycoprotein [Source:HGNC Symbol;Acc:26918]	SMAGP	57228
ENSG00000157240	4.7656308	2.98505998	3.11808431	2.15685841	1.7202887	0.7826507	0.03246191	168	frizzled family receptor 1 [Source:HGNC Symbol;Acc:4038]	FZD1	8321
ENSG00000249437	8.63853825	5.69755881	5.29566537	3.62125501	1.72637095	0.7877425	0.03437719	169	NLR family, apoptosis inhibitory protein [Source:HGNC Symbol;Acc:7634]	NAIP	4671
ENSG00000148346	18.5314242	11.7859283	19.0122925	12.6163618	1.76548459	0.82006423	0.04032235	170	lipocalin 2 [Source:HGNC Symbol;Acc:6526]	LCN2	3934
ENSG00000061918	20.3137181	11.6646619	14.6119295	7.59280475	1.68651489	0.75404506	0.03032434	172	guanylate cyclase 1, soluble, beta 3 [Source:HGNC Symbol;Acc:4687]	GUCY1B3	2983
ENSG00000176714	2.14427411	1.41975497	1.79704179	1.0283524	1.76270077	0.81778759	0.04133257	173	coiled-coil domain containing 121 [Source:HGNC Symbol;Acc:25833]	CCDC121	79635
ENSG00000197959	22.0879462	14.4567579	14.0995119	12.6141327	1.70155295	0.76685205	0.03471672	174	dynamin 3 [Source:HGNC Symbol;Acc:29125]	DNM3	26052
ENSG00000121858	243.311264	161.414871	165.531639	64.8554337	1.58381264	0.66340168	0.01098865	178	tumor necrosis factor (ligand) superfamily, member 10 [Source:HGNC Symbol;Acc:11925]	TNFSF10	8743
ENSG00000110848	35.1701318	66.4689152	28.1736196	65.7504422	-1.6971334	-0.7631	0.0364675	179	CD69 molecule [Source:HGNC Symbol;Acc:1694]	CD69	969
ENSG00000197903	183.305657	108.264069	112.09653	62.7785852	1.68747595	0.75486694	0.0353109	180	histone cluster 1, H2bk [Source:HGNC Symbol;Acc:13954]	HIST1H2BK	85236
ENSG00000019991	11.7427257	7.72231835	6.31113644	2.7994412	1.5836138	0.66322055	0.01234655	182	hepatocyte growth factor (hepapoietin A; scatter factor) [Source:HGNC Symbol;Acc:4893]	HGF	3082
ENSG00000198948	24.3278202	14.680201	17.6702591	10.6539595	1.69836157	0.76414363	0.03830574	184	microfibrillar-associated protein 3-like [Source:HGNC Symbol;Acc:29083]	MFAP3L	101928198
ENSG00000115828	42.0007181	28.0858301	26.4082393	15.8250381	1.62644398	0.70172113	0.02423144	186	glutaminyl-peptide cyclotransferase [Source:HGNC Symbol;Acc:9753]	QPCT	25797
ENSG00000129422	4.38739232	5.72842717	5.02341586	3.63152367	-1.7579741	-0.8139138	0.04749346	187	microtubule associated tumor suppressor 1 [Source:HGNC Symbol;Acc:29789]	MTUS1	57509
ENSG00000136014	3.8598658	5.98095337	2.824637	3.53016596	-1.649234	-0.7217961	0.02962205	188	ubiquitin specific peptidase 44 [Source:HGNC Symbol;Acc:20064]	USP44	84101
ENSG00000129595	2.81675112	5.12339874	2.21796496	3.73956482	-1.7352272	-0.7951246	0.0461704	190	erythrocyte membrane protein band 4.1 like 4A [Source:HGNC Symbol;Acc:13278]	EPB41L4A	64097

ENSG00000146243	3.75690281	5.6872077	2.70819657	2.95343413	-1.6515804	-0.7238472	0.03153948	191	interleukin-1 receptor-associated kinase 1 binding protein 1 [Source:HGNC Symbol;Acc:17368]	IRAK1BP1	134728
ENSG00000184205	29.0884518	46.264976	13.9654319	24.5374875	-1.5706943	-0.6514025	0.01382286	196	TSPY-like 2 [Source:HGNC Symbol;Acc:24358]	TSPYL2	64061
ENSG00000225614	1.76920919	2.72266787	1.50688477	2.02432175	-1.6821909	-0.7503414	0.04036159	199	zinc finger protein 469 [Source:HGNC Symbol;Acc:23216]	ZNF469	84627
ENSG00000198478	31.4930351	19.4708849	22.8922357	15.0971906	1.67065549	0.74041426	0.03818033	200	SH3 domain binding glutamic acid-rich protein like 2 [Source:HGNC Symbol;Acc:15567]	SH3BGRL2	83699
ENSG00000135838	28.389345	20.1231182	13.9797527	8.78572603	1.56430934	0.64552583	0.01499012	203	N-acetylneuraminate pyruvate lyase (dihydrodipicolinate synthase) [Source:HGNC Symbol;Acc:16781]	NPL	80896
ENSG00000088826	11.6133026	7.17583734	7.6766763	4.24363536	1.67654132	0.74548804	0.04112462	205	spermine oxidase [Source:HGNC Symbol;Acc:15862]	SMOX	54498
ENSG00000125459	4.07092475	6.68767784	2.3296545	3.33375433	-1.6344903	-0.7088408	0.03159685	206	misato 1, mitochondrial distribution and morphology regulator [Source:HGNC Symbol;Acc:29678]	MSTO1	55154
ENSG00000137720	2.70452476	4.19330607	1.89764631	2.43668156	-1.6693617	-0.7392966	0.03931203	207	chromosome 11 open reading frame 1 [Source:HGNC Symbol;Acc:1163]	C11orf1	64776
ENSG00000255103	4.48360448	7.52308772	2.76182285	3.3770128	-1.5502615	-0.6325116	0.01117202	209	KIAA0754 [Source:HGNC Symbol;Acc:29111]	KIAA0754	643314
ENSG00000134461	3.09720617	4.22893691	2.4411078	2.00241162	-1.674023	-0.7433194	0.0417388	211	ankyrin repeat domain 16 [Source:HGNC Symbol;Acc:23471]	ANKRD16	54522
ENSG00000072952	42.1135831	28.5054928	25.4622288	19.6615466	1.60676346	0.68415756	0.02704581	212	murine retrovirus integration site 1 homolog [Source:HGNC Symbol;Acc:7237]	MRVI1	10335
ENSG00000118690	3.42139839	5.21987277	2.06823635	2.4564275	-1.5845383	-0.6640625	0.02079359	213	armadillo repeat containing 2 [Source:HGNC Symbol;Acc:23045]	ARMC2	84071
ENSG00000005249	198.083923	123.714277	146.954991	98.4831642	1.66512786	0.73563296	0.04081437	214	protein kinase, cAMP-dependent, regulatory, type II, beta [Source:HGNC Symbol;Acc:9392]	PRKAR2B	5577
ENSG00000047648	18.2053386	10.2657465	12.2437012	5.62173098	1.68465082	0.7524496	0.04513321	215	Rho GTPase activating protein 6 [Source:HGNC Symbol;Acc:676]	ARHGAP6	395
ENSG00000151458	22.5710527	15.5370154	10.509025	6.24703838	1.54962965	0.63192347	0.01240657	216	ankyrin repeat domain 50 [Source:HGNC Symbol;Acc:29223]	ANKRD50	57182
ENSG00000183837	7.00644018	11.0875087	4.798039	7.30194199	-1.6449186	-0.7180162	0.0366483	217	paraneoplastic Ma antigen 3 [Source:HGNC Symbol;Acc:18742]	PNMA3	29944
ENSG00000198590	2.12409877	3.18322806	1.58473311	1.39450997	-1.6630697	-0.7338486	0.04151758	218	chromosome 3 open reading frame 35 [Source:HGNC Symbol;Acc:24082]	C3orf35	339883
ENSG00000141696	3.06479489	4.157838	2.72293647	2.45940922	-1.6632563	-0.7340105	0.04443216	221	leprecan-like 4 [Source:HGNC Symbol;Acc:16946]	LEPREL4	10609
ENSG00000049246	4.53108676	7.42787982	2.69258067	3.26961252	-1.5621867	-0.6435669	0.01912786	223	period circadian clock 3 [Source:HGNC Symbol;Acc:8847]	PER3	8863
ENSG00000204131	9.67550922	8.09474535	4.60092301	5.81450564	1.66062365	0.73172515	0.04493591	225	NHS-like 2 [Source:HGNC Symbol;Acc:33737]	NHSL2	340527
ENSG00000183019	26.3455509	18.5707398	15.9902296	9.54112202	1.56859513	0.64947302	0.02137902	226	chromosome 19 open reading frame 59 [Source:HGNC Symbol;Acc:27291]	C19orf59	199675
ENSG00000174669	3.23454152	4.4166424	2.31733381	2.03503744	-1.6245272	-0.7000199	0.03865538	228	solute carrier family 29 (equilibrative nucleoside transporter), member 2 [Source:HGNC Symbol;Acc:11004]	SLC29A2	3177

ENSG00000159496	9.14953405	5.95374691	5.72174444	3.82238577	1.65392378	0.72589275	0.04745137	231	ral guanine nucleotide dissociation stimulator-like 4 [Source:HGNC Symbol;Acc:31911]	RGL4	266747
ENSG00000184702	52.1610449	29.9867935	39.5902926	19.0353034	1.66368252	0.73438015	0.0489084	234	septin 5 [Source:HGNC Symbol;Acc:9164]	SEPT5	5413
ENSG00000162722	18.8219972	11.4903071	12.1348306	7.00622502	1.63373203	0.70817136	0.04298037	237	tripartite motif containing 58 [Source:HGNC Symbol;Acc:24150]	TRIM58	25893
ENSG00000152213	38.5874664	28.2686115	14.3472818	11.6841605	1.52055475	0.60459776	0.01072959	238	ADP-ribosylation factor-like 11 [Source:HGNC Symbol;Acc:24046]	ARL11	115761
ENSG00000174428	3.88570352	5.91223102	2.12432138	2.48358362	-1.5907127	-0.6696733	0.03239204	239	GTF2I repeat domain containing 2B [Source:HGNC Symbol;Acc:33125]	GTF2IRD2B	389524
ENSG00000121807	68.5116169	44.1023307	41.4273028	19.142979	1.589053	0.66816724	0.03250551	241	chemokine (C-C motif) receptor 2 [Source:HGNC Symbol;Acc:1603]	CCR2	729230
ENSG00000170345	783.10839	579.893104	433.607385	344.159859	1.59780344	0.67608994	0.03747859	246	FBJ murine osteosarcoma viral oncogene homolog [Source:HGNC Symbol;Acc:3796]	FOS	2353
ENSG00000166262	3.25977691	5.18822629	1.89523597	2.63501583	-1.566585	-0.6476231	0.02894408	248	family with sequence similarity 227, member B [Source:HGNC Symbol;Acc:26543]	FAM227B	196951
ENSG00000154814	55.6546697	82.4977946	20.239112	31.9319033	-1.507067	-0.5917435	0.00698666	250	oxidoreductase NAD-binding domain containing 1 [Source:HGNC Symbol;Acc:25128]	OXNAD1	92106
ENSG00000120915	9.54031951	13.721937	6.12888862	6.48226552	-1.5292455	-0.6128201	0.01724656	253	epoxide hydrolase 2, cytoplasmic [Source:HGNC Symbol;Acc:3402]	EPHX2	2053
ENSG00000111817	67.0005141	47.8762958	19.4122555	11.9441669	1.49264506	0.57787115	0.00293947	255	dermatan sulfate epimerase [Source:HGNC Symbol;Acc:21144]	DSE	29940
ENSG00000134755	31.8228633	18.8838085	21.2650648	9.89570007	1.60956718	0.68667279	0.04455202	258	desmocollin 2 [Source:HGNC Symbol;Acc:3036]	DSC2	1824
ENSG00000154027	5.66910584	8.26565837	3.30214544	4.97432886	-1.558851	-0.640483	0.03150366	260	adenylate kinase 5 [Source:HGNC Symbol;Acc:365]	AK5	26289
ENSG00000187116	101.805731	70.2966498	43.1295122	26.5717511	1.52080139	0.60483175	0.01756556	261	leukocyte immunoglobulin-like receptor, subfamily A (with TM domain), member 5 [Source:HGNC Symbol;Acc:16309]	LILRA5	353514
ENSG00000112394	8.01546125	11.0257792	4.84476335	5.94357125	-1.5775299	-0.6576673	0.03824341	265	solute carrier family 16 (aromatic amino acid transporter), member 10 [Source:HGNC Symbol;Acc:17027]	SLC16A10	117247
ENSG00000168209	18.7353585	32.0306424	12.8861359	20.6406497	-1.5642174	-0.6454411	0.03602144	266	DNA-damage-inducible transcript 4 [Source:HGNC Symbol;Acc:24944]	DDIT4	54541
ENSG00000051128	34.796217	25.0527018	15.7811836	9.29282427	1.50667868	0.59137178	0.01547469	268	homer homolog 3 (Drosophila) [Source:HGNC Symbol;Acc:17514]	HOMER3	9454
ENSG00000182183	12.150658	18.605952	6.76768533	12.0249161	-1.5878058	-0.6670344	0.04478672	269	family with sequence similarity 159, member A [Source:HGNC Symbol;Acc:28757]	FAM159A	348378
ENSG00000007264	14.2970099	22.6186035	8.00885724	11.1835492	-1.530305	-0.6138192	0.02560836	272	megakaryocyte-associated tyrosine kinase [Source:HGNC Symbol;Acc:6906]	MATK	4145
ENSG00000130830	67.3870492	48.0623207	22.0626338	13.2745165	1.48745961	0.5728505	0.00718973	273	membrane protein, palmitoylated 1, 55kDa [Source:HGNC Symbol;Acc:7219]	MPP1	4354
ENSG00000174125	98.9730912	70.744484	42.8825098	30.2838379	1.50963177	0.59419669	0.0199997	277	toll-like receptor 1 [Source:HGNC Symbol;Acc:11847]	TLR1	7096
ENSG00000170677	10.5115374	7.31150662	4.47074543	4.46257931	1.53477762	0.61802963	0.02974931	279	suppressor of cytokine signaling 6 [Source:HGNC Symbol;Acc:16833]	SOCS6	9306

ENSG00000130150	45.8355264	33.9205793	20.4081806	14.7678971	1.50613143	0.59084767	0.01865103	280	motile sperm domain containing 2 [Source:HGNC Symbol;Acc:28381]	MOSPD2	158747
ENSG00000162614	50.6725618	33.1195057	33.593729	21.4444036	1.58018042	0.66008929	0.04883448	282	nexilin (F actin binding protein) [Source:HGNC Symbol;Acc:29557]	NEXN	91624
ENSG00000175538	126.747346	94.7225233	67.3773741	52.4544818	1.5415112	0.62434537	0.03763328	287	potassium voltage-gated channel, Isk-related family, member 3 [Source:HGNC Symbol;Acc:6243]	KCNE3	10008
ENSG00000120306	17.0726301	12.1354378	10.9795818	7.1635239	1.55746928	0.6392037	0.04506007	290	cysteine-rich transmembrane module containing 1 [Source:HGNC Symbol;Acc:30239]	CYSTM1	84418
ENSG00000136514	9.34961199	7.49076916	7.757583	4.58980032	1.54587198	0.62842084	0.04155664	294	receptor (chemosensory) transporter protein 4 [Source:HGNC Symbol;Acc:23992]	RTP4	64108
ENSG00000188313	80.9754985	54.7075214	58.5271194	24.4455005	1.5092453	0.59382731	0.02869073	298	phospholipid scramblase 1 [Source:HGNC Symbol;Acc:9092]	PLSCR1	5359
ENSG00000156026	11.2160221	8.74345441	4.37689937	4.020087	1.49771252	0.58276073	0.02334578	303	mitochondrial calcium uniporter [Source:HGNC Symbol;Acc:23526]	MCU	90550
ENSG00000121413	16.1399362	22.1906788	8.78795653	7.88238489	-1.4814483	-0.5670083	0.01684346	304	zinc finger and SCAN domain containing 18 [Source:HGNC Symbol;Acc:21037]	ZSCAN18	65982
ENSG00000139187	59.0234362	87.6401303	32.9837046	40.8127921	-1.5082653	-0.5928902	0.0292854	307	killer cell lectin-like receptor subfamily G, member 1 [Source:HGNC Symbol;Acc:6380]	KLRG1	10219
ENSG00000172543	32.7523923	53.6822672	17.1920231	26.4235791	-1.5099767	-0.5945263	0.03220778	310	cathepsin W [Source:HGNC Symbol;Acc:2546]	CTSW	1521
ENSG00000163563	1344.41885	965.896201	713.33331	422.081624	1.49875448	0.58376407	0.02922261	314	myeloid cell nuclear differentiation antigen [Source:HGNC Symbol;Acc:7183]	MNDA	4332
ENSG00000100385	29.4760085	42.4996243	13.8190774	15.372572	-1.4793964	-0.5650087	0.02058509	316	interleukin 2 receptor, beta [Source:HGNC Symbol;Acc:6009]	IL2RB	3560
ENSG00000175471	98.3543208	71.8188992	28.7899545	21.408754	1.45692816	0.54292974	0.01210719	323	multiple C2 domains, transmembrane 1 [Source:HGNC Symbol;Acc:26183]	MCTP1	79772
ENSG00000105717	5.56460176	8.32566414	2.45675883	3.63504404	-1.4769642	-0.5626349	0.02240107	324	pre-B-cell leukemia homeobox 4 [Source:HGNC Symbol;Acc:13403]	PBX4	80714
ENSG00000159128	81.8960177	61.5111961	33.0010528	29.7920466	1.483334	0.56884348	0.02841436	328	interferon gamma receptor 2 (interferon gamma transducer 1) [Source:HGNC Symbol;Acc:5440]	IFNGR2	3460
ENSG00000121316	126.743875	96.2601991	54.3245646	38.7709516	1.46489749	0.55079971	0.01939424	333	phospholipase B domain containing 1 [Source:HGNC Symbol;Acc:26215]	PLBD1	79887
ENSG00000161381	6.27085662	9.34290966	3.93235385	4.13661285	-1.5088706	-0.593469	0.04232244	336	plexin domain containing 1 [Source:HGNC Symbol;Acc:20945]	PLXDC1	57125
ENSG00000163492	19.3347216	25.9556014	8.98527119	9.76644139	-1.4597411	-0.5457126	0.02100733	341	coiled-coil domain containing 141 [Source:HGNC Symbol;Acc:26821]	CCDC141	285025
ENSG00000073464	12.2176692	8.90157289	5.04531064	4.78808555	1.51084496	0.59535562	0.04908534	343	chloride channel, voltage-sensitive 4 [Source:HGNC Symbol;Acc:2022]	CLCN4	1183
ENSG00000115271	222.638985	158.291589	117.667759	80.5028847	1.49871785	0.58372881	0.04369296	345	grancalcin, EF-hand calcium binding protein [Source:HGNC Symbol;Acc:15990]	GCA	25801
ENSG00000116729	16.230434	10.6771637	8.02055107	4.46097752	1.50778356	0.59242935	0.04845024	346	wntless Wnt ligand secretion mediator [Source:HGNC Symbol;Acc:30238]	WLS	79971
ENSG00000140090	33.7675493	25.8065194	12.8416399	11.4677049	1.46314715	0.54907487	0.02804402	352	solute carrier family 24 (sodium/potassium/calcium exchanger), member 4 [Source:HGNC Symbol;Acc:10978]	SLC24A4	123041

ENSG00000181274	178.978428	126.445078	103.503837	78.2684763	1.5029451	0.58779231	0.04886398	356	frequently rearranged in advanced T-cell lymphomas 2 [Source:HGNC Symbol;Acc:16048]	FRAT2	23401
ENSG00000188906	412.146635	295.448295	187.695805	122.100478	1.47635816	0.56204276	0.03660486	359	leucine-rich repeat kinase 2 [Source:HGNC Symbol;Acc:18618]	LRRK2	120892
ENSG00000173511	6.9476532	9.84781346	3.24503497	3.12693976	-1.4951112	-0.5802528	0.04808772	365	vascular endothelial growth factor B [Source:HGNC Symbol;Acc:12681]	VEGFB	7423
ENSG00000167775	7.56919545	11.3367892	3.48120736	4.5583269	-1.4624284	-0.548366	0.03042328	367	CD320 molecule [Source:HGNC Symbol;Acc:16692]	CD320	51293
ENSG00000138449	61.3562862	44.0599345	21.7636362	13.0653849	1.4417789	0.52784994	0.0198054	373	solute carrier family 40 (iron-regulated transporter), member 1 [Source:HGNC Symbol;Acc:10909]	SLC40A1	30061
ENSG00000185453	5.55119646	8.58612382	2.31034024	3.69402488	-1.4858608	-0.5712989	0.0483833	376	chromosome 19 open reading frame 68 [Source:HGNC Symbol;Acc:34495]	C19orf68	374920
ENSG00000122862	1462.71015	1026.62452	777.467256	450.43342	1.471536	0.55732284	0.04217417	380	serglycin [Source:HGNC Symbol;Acc:9361]	SRGN	5552
ENSG00000169398	14.8912295	9.62799727	7.49289857	4.19751931	1.47324592	0.55899827	0.04542799	391	protein tyrosine kinase 2 [Source:HGNC Symbol;Acc:9611]	PTK2	5747
ENSG00000113532	120.921058	92.0453894	35.6861458	24.6559863	1.417722	0.50357466	0.01178461	392	ST8 alpha-N-acetyl-neuraminide alpha-2,8-sialyltransferase 4 [Source:HGNC Symbol;Acc:10871]	ST8SIA4	7903
ENSG00000160856	27.1409775	41.9224853	11.6657429	17.6989475	-1.4573365	-0.5433341	0.03986718	399	Fc receptor-like 3 [Source:HGNC Symbol;Acc:18506]	FCRL3	115352
ENSG00000143207	42.1832398	32.2943489	12.856976	11.1404386	1.43127773	0.51730365	0.02209724	400	ring finger and WD repeat domain 2, E3 ubiquitin protein ligase [Source:HGNC Symbol;Acc:17440]	RFWD2	64326
ENSG00000260916	36.5755781	27.024337	14.6408818	12.5066726	1.4609278	0.54688488	0.04340409	402	cell cycle progression 1 [Source:HGNC Symbol;Acc:24227]	CCPG1	9236
ENSG00000186260	6.04048977	8.72771623	3.37136166	3.82553201	-1.461947	-0.547891	0.04495115	406	MKL/myocardin-like 2 [Source:HGNC Symbol;Acc:29819]	MKL2	57496
ENSG00000114541	53.1636492	39.6570703	18.3979769	12.0360893	1.41876663	0.5046373	0.01590955	407	FERM domain containing 4B [Source:HGNC Symbol;Acc:24886]	FRMD4B	23150
ENSG00000174130	71.3712457	53.0144065	32.0450653	20.8339176	1.44794656	0.53400835	0.03981976	418	toll-like receptor 6 [Source:HGNC Symbol;Acc:16711]	TLR6	10333
ENSG00000130956	11.9184638	16.4191742	5.16005009	5.02478805	-1.4304973	-0.5165168	0.02864639	426	hyaluronan binding protein 4 [Source:HGNC Symbol;Acc:17062]	HABP4	22927
ENSG00000161920	10.9001025	8.27132941	4.84197367	3.41179015	1.44493688	0.53100647	0.04214055	428	mediator complex subunit 11 [Source:HGNC Symbol;Acc:32687]	MED11	400569
ENSG00000205664	4.46020734	6.40818861	2.39700075	3.75856198	-1.457111	-0.5431108	0.0496172	430	HCG1981372, isoform CRA_c; Uncharacterized protein [Source:UniProtKB/TrEMBL;Acc:B1B108]	RP11-706O15.1	101930102
ENSG00000114450	25.3203598	19.6217719	7.64399738	5.7181932	1.41085347	0.49656816	0.0176707	437	guanine nucleotide binding protein (G protein), beta polypeptide 4 [Source:HGNC Symbol;Acc:20731]	GNB4	59345
ENSG00000131067	6.56799655	9.28745544	3.02069615	3.70553843	-1.4406872	-0.5267572	0.04801554	449	gamma-glutamyltransferase 7 [Source:HGNC Symbol;Acc:4259]	GGT7	2686
ENSG00000183621	31.1311782	23.4537921	9.28685505	7.02398134	1.41396439	0.49974579	0.03164333	461	zinc finger protein 438 [Source:HGNC Symbol;Acc:21029]	ZNF438	220929
ENSG00000126351	30.4777874	43.7095674	7.91067258	10.748305	-1.3892863	-0.474344	0.01280569	464	thyroid hormone receptor, alpha [Source:HGNC Symbol;Acc:11796]	THRA	7067

ENSG00000168461	278.126346	213.635119	86.2217377	53.1618344	1.39670861	0.48203107	0.017355	466	RAB31, member RAS oncogene family [Source:HGNC Symbol;Acc:9771]	RAB31	11031
ENSG00000143409	28.3514956	21.5028115	11.7614119	8.65610593	1.436256	0.52231292	0.04934999	467	family with sequence similarity 63, member A [Source:HGNC Symbol;Acc:25648]	FAM63A	55793
ENSG00000169413	71.6633428	56.2864417	24.7692771	19.8061905	1.40341683	0.48894357	0.02847814	471	ribonuclease, RNase A family, k6 [Source:HGNC Symbol;Acc:10048]	RNASE6	6039
ENSG00000132357	25.2729908	19.7583817	9.70984224	7.48448556	1.41476556	0.500563	0.04069515	480	caspase recruitment domain family, member 6 [Source:HGNC Symbol;Acc:16394]	CARD6	84674
ENSG00000139132	91.1921189	71.1265014	35.3994983	23.1857004	1.40696717	0.49258867	0.03707647	484	FYVE, RhoGEF and PH domain containing 4 [Source:HGNC Symbol;Acc:19125]	FGD4	121512
ENSG00000070190	161.995889	125.007246	58.7496495	52.6640281	1.42260795	0.50853813	0.04942735	485	dual adaptor of phosphotyrosine and 3-phosphoinositides [Source:HGNC Symbol;Acc:16500]	DAPP1	27071
ENSG00000109458	15.563748	11.6844256	5.24844528	4.2318939	1.4128073	0.49856471	0.04399679	489	GRB2-associated binding protein 1 [Source:HGNC Symbol;Acc:4066]	GAB1	2549
ENSG00000138050	10.3713614	14.9143238	3.93069749	5.49596194	-1.4126133	-0.4983666	0.04727461	499	THUMP domain containing 2 [Source:HGNC Symbol;Acc:14890]	THUMPD2	80745
ENSG00000005020	266.364356	207.003457	67.0153534	50.8801664	1.38599179	0.47091871	0.02128541	501	src kinase associated phosphoprotein 2 [Source:HGNC Symbol;Acc:15687]	SKAP2	8935
ENSG00000138293	319.064846	246.981773	95.5809865	69.7043269	1.38723242	0.47220953	0.02567212	508	nuclear receptor coactivator 4 [Source:HGNC Symbol;Acc:7671]	NCOA4	8031
ENSG00000104093	203.623247	159.619851	60.2258363	45.8343155	1.38949129	0.47455679	0.0297577	519	Dmx-like 2 [Source:HGNC Symbol;Acc:2938]	DMXL2	23312
ENSG00000055483	55.878359	80.9379676	19.9505218	28.0126483	-1.3891458	-0.4741981	0.02935679	520	ubiquitin specific peptidase 36 [Source:HGNC Symbol;Acc:20062]	USP36	57602
ENSG00000156273	118.790395	92.3442717	31.4161017	21.9105284	1.38070374	0.4654038	0.02225494	524	BTB and CNC homology 1, basic leucine zipper transcription factor 1 [Source:HGNC Symbol;Acc:935]	BACH1	100379661
ENSG00000107833	13.7178929	19.766413	5.01196279	7.57664338	-1.4046998	-0.4902618	0.04788079	528	nucleophosmin/nucleoplasmin 3 [Source:HGNC Symbol;Acc:7931]	NPM3	10360
ENSG00000101457	44.343815	34.7738437	14.2571881	14.5674797	1.39401126	0.47924222	0.04955573	580	deoxynucleotidyltransferase, terminal, interacting protein 1 [Source:HGNC Symbol;Acc:16160]	DNTTIP1	116092
ENSG00000025039	28.9419896	21.948042	9.47768556	7.11491991	1.39094251	0.47606279	0.0488197	584	Ras-related GTP binding D [Source:HGNC Symbol;Acc:19903]	RRAGD	58528
ENSG00000164305	24.8416714	19.491333	8.95543913	7.64088426	1.38700411	0.47197207	0.04837364	592	caspase 3, apoptosis-related cysteine peptidase [Source:HGNC Symbol;Acc:1504]	CASP3	836
ENSG00000013725	54.8884703	74.6835811	20.7192842	25.0480331	-1.377396	-0.4619434	0.04439015	613	CD6 molecule [Source:HGNC Symbol;Acc:1691]	CD6	923
ENSG00000068366	92.5691186	71.9322991	31.5184981	21.2461881	1.3773223	0.46186619	0.04865572	624	acyl-CoA synthetase long-chain family member 4 [Source:HGNC Symbol;Acc:3571]	ACSL4	2182
ENSG00000120594	193.386461	154.808134	45.575503	41.1851125	1.35634444	0.43972359	0.03719019	653	plexin domain containing 2 [Source:HGNC Symbol;Acc:21013]	PLXDC2	84898
ENSG00000187231	67.8419212	54.4825345	17.4443232	14.9448342	1.34694568	0.42969167	0.04888774	705	SEC14 and spectrin domains 1 [Source:HGNC Symbol;Acc:18379]	SESTD1	91404
ENSG00000111885	41.981869	33.9792968	9.65398082	7.96370262	1.33345572	0.41516992	0.048076	754	mannosidase, alpha, class 1A, member 1 [Source:HGNC Symbol;Acc:6821]	MAN1A1	4121

Compared populations:

ALL RA vs HCs (sex-adjusted)

Fold change describes the size of the difference in gene expression (given in mean and standard deviation, sd) between the compared groups. The values are always in relation to the group used as a base level group (reference). P-value describes the reliability of the change in expression value between the compared groups. Results were considered significant with a fold change of 1.2 and a p-value cutoff of 0.05.

Supplementary Material 24 - List of differentially expressed (DE) genes (sex-adjusted)

ID	mean_D2TRA	mean_Control	sd_D2TRA	sd_Control	FC	logFC	P.Value	avgRank	Description	geneName	EntrezID
ENSG00000169877	3.47824512	0.38280226	5.62173009	0.63392344	5.63285522	2.49386639	0.00013579	1	alpha hemoglobin stabilizing protein [Source:HGNC Symbol;Acc:18075]	AHSP	51327
ENSG00000158578	8.27570027	1.39422151	9.72455279	2.76471307	7.24487345	2.85696049	0.00040812	2	aminolevulinate, delta-, synthase 2 [Source:HGNC Symbol;Acc:397]	ALAS2	212
ENSG00000244734	2828.3627	1155.37854	2825.757	2229.79337	4.52661564	2.17843281	6.9413E-05	3	hemoglobin, beta [Source:HGNC Symbol;Acc:4827]	HBB	3043
ENSG00000144290	5.35182209	12.6637233	7.57307746	16.339091	-4.2773895	-2.0967306	7.2534E-05	4	solute carrier family 4, sodium bicarbonate transporter, member 10 [Source:HGNC Symbol;Acc:13811]	SLC4A10	57282
ENSG00000134668	3.54275114	1.16141706	3.12594344	1.17629249	3.55837931	1.8312203	0.0005626	5	SPOC domain containing 1 [Source:HGNC Symbol;Acc:26338]	SPOCD1	90853
ENSG00000206177	6.33683933	1.23144829	9.77527504	1.80420905	5.43467775	2.44219449	0.00079822	6	hemoglobin, mu [Source:HGNC Symbol;Acc:4826]	HBM	3042
ENSG00000133169	1.62911127	0.35488561	1.64262679	0.55120452	3.46212757	1.79165889	0.00067485	7	brain expressed, X-linked 1 [Source:HGNC Symbol;Acc:1036]	BEX1	55859
ENSG00000132970	4.2045429	1.80640219	3.83513891	2.01607679	3.07926705	1.62258699	0.00072705	8	WAS protein family, member 3 [Source:HGNC Symbol;Acc:12734]	WASF3	10810
ENSG00000227184	1.5725828	3.26868176	2.08233084	2.37719746	-2.9381585	-1.5549122	0.00077558	9	epiplakin 1 [Source:HGNC Symbol;Acc:15577]	EPPK1	83481
ENSG00000206172	181.017066	84.7344559	176.210819	131.290964	3.49312956	1.80452015	0.00109095	10	hemoglobin, alpha 1 [Source:HGNC Symbol;Acc:4823]	HBA1	3040
ENSG00000136425	0.77274466	1.82072473	0.96679442	1.70073381	-3.0155523	-1.5924222	0.00083086	11	calcium and integrin binding family member 2 [Source:HGNC Symbol;Acc:24579]	CIB2	10518
ENSG00000088053	10.6267166	4.95428782	7.61023427	4.32648309	2.64474458	1.4031284	0.00070072	12	glycoprotein VI (platelet) [Source:HGNC Symbol;Acc:14388]	GP6	51206
ENSG00000155755	1.57511129	3.16327296	1.42477157	1.8717985	-2.4902551	-1.3162935	0.0004149	13	transmembrane protein 237 [Source:HGNC Symbol;Acc:14432]	TMEM237	65062
ENSG00000072195	0.89960388	1.77279893	1.14865551	1.08506981	-2.7884777	-1.4794777	0.00090344	14	SPEG complex locus [Source:HGNC Symbol;Acc:16901]	SPEG	100996693
ENSG00000188536	644.643297	370.226899	588.627982	623.36819	3.07278635	1.61954746	0.00231563	15	hemoglobin, alpha 2 [Source:HGNC Symbol;Acc:4824]	HBA2	3040
ENSG00000062524	1.39930446	3.66819787	1.83756453	3.35933968	-2.7170168	-1.4420235	0.00172435	16	leukocyte receptor tyrosine kinase [Source:HGNC Symbol;Acc:6721]	LTK	4058
ENSG00000109674	4.78588499	2.01492106	3.38021591	1.22693646	2.53910459	1.34431983	0.00098326	17	nei endonuclease VIII-like 3 (E. coli) [Source:HGNC Symbol;Acc:24573]	NEIL3	55247
ENSG00000198829	5.02325958	1.93308799	4.23907361	1.67530504	2.61399877	1.38625846	0.00210137	18	succinate receptor 1 [Source:HGNC Symbol;Acc:4542]	SUCNR1	56670

ENSG00000102010	3.88766843	1.73630081	3.80339633	2.29650182	3.20843697	1.68187064	0.00537068	19	BMX non-receptor tyrosine kinase [Source:HGNC Symbol;Acc:1079]	BMX	660
ENSG00000223609	6.58854095	1.07968881	11.8271749	1.77616381	4.52736223	2.17867074	0.00666574	20	hemoglobin, delta [Source:HGNC Symbol;Acc:4829]	HBD	3045
ENSG00000163462	1.05254675	1.82346664	1.41870713	1.44255459	-2.5905356	-1.3732504	0.00391823	21	tripartite motif containing 46 [Source:HGNC Symbol;Acc:19019]	TRIM46	80128
ENSG00000144645	12.2080991	19.7749679	10.1662655	8.15709664	-2.5528361	-1.3521009	0.00414214	22	oxysterol binding protein-like 10 [Source:HGNC Symbol;Acc:16395]	OSBPL10	114884
ENSG00000072422	12.122805	6.64826572	7.72700623	5.6561545	2.42883351	1.2802636	0.0035422	23	Rho-related BTB domain containing 1 [Source:HGNC Symbol;Acc:18738]	RHOBTB1	9886
ENSG00000214595	3.67155932	5.52231297	3.61562527	2.45002887	-2.3741023	-1.2473821	0.00398618	24	echinoderm microtubule associated protein like 6 [Source:HGNC Symbol;Acc:35412]	EML6	400954
ENSG00000047597	5.14087794	2.17170901	5.67081122	2.47853265	2.43413416	1.28340869	0.00457492	25	X-linked Kx blood group (McLeod syndrome) [Source:HGNC Symbol;Acc:12811]	XK	7504
ENSG00000143416	2.01598098	0.61392877	2.81402592	1.04208788	2.93796361	1.55481653	0.00764548	26	selenium binding protein 1 [Source:HGNC Symbol;Acc:10719]	SELENBP1	8991
ENSG00000148498	6.04556086	2.81407373	4.30298988	2.22724482	2.37273796	1.24655278	0.0051115	27	par-3 family cell polarity regulator [Source:HGNC Symbol;Acc:16051]	PARD3	56288
ENSG00000225614	1.3328699	2.72266787	1.19368173	2.02432175	-2.2415495	-1.1644964	0.00201112	28	zinc finger protein 469 [Source:HGNC Symbol;Acc:23216]	ZNF469	84627
ENSG00000136231	13.9756968	6.84541599	8.95580445	4.56307794	2.17319321	1.11981645	0.0017508	29	insulin-like growth factor 2 mRNA binding protein 3 [Source:HGNC Symbol;Acc:28868]	IGF2BP3	10643
ENSG00000123095	2.29006987	3.30409383	2.9300176	2.56240833	-2.4277619	-1.279627	0.00950334	30	basic helix-loop-helix family, member e41 [Source:HGNC Symbol;Acc:16617]	BHLHE41	79365
ENSG00000183960	0.92660728	2.01588576	1.27278734	2.14029222	-2.5164977	-1.3314173	0.01064093	31	potassium voltage-gated channel, subfamily H (eag-related), member 8 [Source:HGNC Symbol;Acc:18864]	KCNH8	131096
ENSG00000003436	5.9307401	3.03616628	4.69642502	3.55208305	2.37838436	1.24998188	0.01031452	32	tissue factor pathway inhibitor (lipoprotein-associated coagulation inhibitor) [Source:HGNC Symbol;Acc:11760]	TFPI	7035
ENSG00000137098	1.84182554	3.55431904	1.79254008	2.430343	-2.2989165	-1.2009541	0.00886786	33	sperm associated antigen 8 [Source:HGNC Symbol;Acc:14105]	SPAG8	26206
ENSG00000172159	26.834434	15.0683043	13.6293622	9.83920651	2.05060752	1.03605139	0.00049618	34	FERM domain containing 3 [Source:HGNC Symbol;Acc:24125]	FRMD3	257019
ENSG00000204301	1.30383091	2.30680649	1.58064892	1.36271882	-2.2188663	-1.1498227	0.00604581	35	notch 4 [Source:HGNC Symbol;Acc:7884]	NOTCH4	4855

ENSG00000146072	1.36090319	3.18329077	1.41129707	2.63953888	-2.2765813	-1.186869	0.00876454	36	tumor necrosis factor receptor superfamily, member 21 [Source:HGNC Symbol;Acc:13469]	TNFRSF21	27242
ENSG00000118113	4.75104055	1.01699309	8.92360272	1.18748809	2.88282974	1.52748564	0.01560954	37	matrix metallopeptidase 8 (neutrophil collagenase) [Source:HGNC Symbol;Acc:7175]	MMP8	4317
ENSG00000269636	6.98117565	3.21788126	6.47118936	3.20646761	2.56971823	1.36161018	0.01446548	38		AC010441.1	
ENSG00000066923	1.29669089	2.33684981	1.0903448	1.32246936	-2.159248	-1.110529	0.00616284	39	stromal antigen 3 [Source:HGNC Symbol;Acc:11356]	STAG3	10734
ENSG00000204311	0.87983151	1.5379885	1.31308785	1.34792762	-2.2465797	-1.1677303	0.00984287	40	deafness, autosomal recessive 59 [Source:HGNC Symbol;Acc:29502]	DFNB59	494513
ENSG00000167747	2.08979697	3.24346137	2.44630648	1.81735058	-2.2339893	-1.1596223	0.00895726	41	chromosome 19 open reading frame 48 [Source:HGNC Symbol;Acc:29667]	C19orf48	692204
ENSG00000187808	10.0387804	6.64283071	3.61378174	4.74125179	1.94776556	0.96182004	0.00043021	42	sosondowah ankyrin repeat domain family member D [Source:HGNC Symbol;Acc:32960]	SOWAHD	347454
ENSG00000149131	14.5800686	6.05473362	26.2417887	7.9749508	2.60997375	1.3840353	0.01562119	43	serpin peptidase inhibitor, clade G (C1 inhibitor), member 1 [Source:HGNC Symbol;Acc:1228]	SERPING1	710
ENSG00000180190	2.17520524	1.25558605	2.14737591	1.36125879	2.25244892	1.17149439	0.01146365	44	testis development related protein [Source:HGNC Symbol;Acc:26951]	TDRP	157695
ENSG00000080493	3.64673659	7.18019559	2.38622598	4.06565712	-2.0715122	-1.0506843	0.00443218	45	solute carrier family 4 (sodium bicarbonate cotransporter), member 4 [Source:HGNC Symbol;Acc:11030]	SLC4A4	8671
ENSG00000198934	4.57797025	7.71111476	2.54361894	3.71575058	-1.9893129	-0.9922702	0.00277534	46	melanoma antigen family E, 1 [Source:HGNC Symbol;Acc:24934]	MAGEE1	57692
ENSG00000114405	1.99587842	1.29647683	1.57969768	1.56660677	2.30296124	1.20349013	0.01443274	47	chromosome 3 open reading frame 14 [Source:HGNC Symbol;Acc:25024]	C3orf14	57415
ENSG00000213077	2.96288627	1.5785249	2.57042701	2.85794755	2.8406811	1.50623688	0.01752698	48	family with sequence similarity 106, member A [Source:HGNC Symbol;Acc:25682]	FAM106A	
ENSG00000213782	1.98269568	4.03196829	1.92064649	3.52441715	-2.2053268	-1.1409924	0.01093028	49	DEAD (Asp-Glu-Ala-Asp) box polypeptide 47 [Source:HGNC Symbol;Acc:18682]	DDX47	51202
ENSG00000197933	1.42982069	2.0470787	1.53551729	1.25904338	-2.1358564	-1.0948147	0.00870233	50	zinc finger protein 823 [Source:HGNC Symbol;Acc:30936]	ZNF823	55552
ENSG00000137757	23.6170229	10.6902111	18.8001361	7.22443386	2.1025832	1.07216289	0.00736026	51	caspase 5, apoptosis-related cysteine peptidase [Source:HGNC Symbol;Acc:1506]	CASP5	838
ENSG00000055732	2.90230939	1.07271642	2.27186453	0.93371837	2.24880295	1.16915725	0.01395784	52	mucolipin 3 [Source:HGNC Symbol;Acc:13358]	MCOLN3	55283

ENSG00000128512	12.9062833	6.93932878	8.19233688	5.53941973	2.09039384	1.06377478	0.00725422	53	dedicator of cytokinesis 4 [Source:HGNC Symbol;Acc:19192]	DOCK4	9732
ENSG00000105559	0.86524645	1.79840539	1.01511754	1.56027492	-2.3139717	-1.2103712	0.01613752	54	pleckstrin homology domain containing, family A (phosphoinositide binding specific) member 4 [Source:HGNC Symbol;Acc:14339]	PLEKHA4	57664
ENSG00000215883	1.37724366	2.62281763	1.34563012	1.76691776	-2.1911274	-1.1316734	0.01215268	55	cytochrome b5 reductase-like [Source:HGNC Symbol;Acc:32220]	CYB5RL	606495
ENSG00000147650	5.7894807	3.18953792	3.46871679	2.220231	2.07050966	1.04998593	0.00738151	56	low density lipoprotein receptor-related protein 12 [Source:HGNC Symbol;Acc:31708]	LRP12	29967
ENSG00000156738	236.465099	283.304564	162.989959	82.3349449	-2.9322547	-1.5520104	0.02095972	57	membrane-spanning 4-domains, subfamily A, member 1 [Source:HGNC Symbol;Acc:7315]	MS4A1	931
ENSG00000150672	1.63068591	3.02610937	2.01904788	2.29957587	-2.2087403	-1.1432238	0.01419174	58	discs, large homolog 2 (Drosophila) [Source:HGNC Symbol;Acc:2901]	DLG2	1740
ENSG00000070371	2.90168015	1.68352126	1.49631783	1.33144927	2.15806438	1.10973791	0.01177242	59	clathrin, heavy chain-like 1 [Source:HGNC Symbol;Acc:2093]	CLTCL1	8218
ENSG00000198003	2.55400519	1.42052818	2.13931387	1.6009123	2.34351966	1.2286769	0.01676574	60	coiled-coil domain containing 151 [Source:HGNC Symbol;Acc:28303]	CCDC151	115948
ENSG00000189127	4.17155174	1.91316457	3.32090118	1.97281077	2.33224711	1.22172065	0.01672069	61	ankyrin repeat domain 34B [Source:HGNC Symbol;Acc:33736]	ANKRD34B	340120
ENSG00000082196	1.25482228	2.2556547	1.05169292	1.42744948	-2.1570791	-1.1090791	0.01236636	62	C1q and tumor necrosis factor related protein 3 [Source:HGNC Symbol;Acc:14326]	C1QTNF3	114899
ENSG00000153162	3.18606467	1.47847307	2.80113024	1.27450465	2.255863	1.17367945	0.01625294	63	bone morphogenetic protein 6 [Source:HGNC Symbol;Acc:1073]	BMP6	654
ENSG00000129595	2.61003499	5.12339874	2.37417772	3.73956482	-2.1168757	-1.0819365	0.01138459	64	erythrocyte membrane protein band 4.1 like 4A [Source:HGNC Symbol;Acc:13278]	EPB41L4A	64097
ENSG00000109684	1.77337943	3.11702537	1.28137378	1.77189658	-2.0043853	-1.0031598	0.00767572	65	cytokine-dependent hematopoietic cell linker [Source:HGNC Symbol;Acc:17438]	CLNK	116449
ENSG00000171241	3.69835153	2.12866434	2.23531939	1.80038289	2.0741658	1.05253122	0.01041842	66	SHC SH2-domain binding protein 1 [Source:HGNC Symbol;Acc:29547]	SHCBP1	79801
ENSG00000266967	2.08482955	3.50904551	1.8464193	2.29276099	-2.1250727	-1.0875122	0.01312297	67	alanyl-tRNA synthetase domain containing 1 [Source:HGNC Symbol;Acc:28417]	AARSD1	80755
ENSG00000162882	1.90431538	1.63925161	1.40720328	2.19176912	2.24260065	1.16517274	0.01669594	68	3-hydroxyanthranilate 3,4-dioxygenase [Source:HGNC Symbol;Acc:4796]	HAAO	23498
ENSG00000125459	3.52898994	6.68767784	2.36416743	3.33375433	-1.9245425	-0.9445155	0.00684758	69	misato 1, mitochondrial distribution and morphology	MSTO1	55154

									regulator [Source:HGNC Symbol;Acc:29678]		
ENSG00000115423	1.41993603	2.20643355	1.66576513	1.84157281	-2.2292368	-1.1565499	0.01722122	70	dynein, axonemal, heavy chain 6 [Source:HGNC Symbol;Acc:2951]	DNAH6	1768
ENSG00000137807	2.13336999	1.47873518	1.06204442	1.25016221	2.05604662	1.03987298	0.01272215	71	kinesin family member 23 [Source:HGNC Symbol;Acc:6392]	KIF23	9493
ENSG00000130147	1.02068351	1.57679183	1.92688898	1.10425085	-2.2780956	-1.1878283	0.02075546	72	SH3-domain binding protein 4 [Source:HGNC Symbol;Acc:10826]	SH3BP4	23677
ENSG00000112299	16.3442847	10.6677606	9.32365768	9.09304534	1.92668301	0.94611923	0.00886837	73	vanin 1 [Source:HGNC Symbol;Acc:12705]	VNN1	8876
ENSG00000091129	0.88592989	2.28095957	1.14795006	2.41489514	-2.301715	-1.2027092	0.02163846	74	neuronal cell adhesion molecule [Source:HGNC Symbol;Acc:7994]	NRCAM	4897
ENSG00000196092	25.4991015	33.6358179	18.5048802	12.9021011	-2.355517	-1.2360437	0.02358569	75	paired box 5 [Source:HGNC Symbol;Acc:8619]	PAX5	5079
ENSG00000167633	2.28958931	4.13324238	3.21659709	3.02006715	-2.2201487	-1.1506563	0.02002797	76	killer cell immunoglobulin-like receptor, three domains, long cytoplasmic tail, 1 [Source:HGNC Symbol;Acc:6338]	KIR3DL1	3812
ENSG00000134461	2.67617704	4.22893691	2.21742265	2.00241162	-1.9726649	-0.9801459	0.01203441	77	ankyrin repeat domain 16 [Source:HGNC Symbol;Acc:23471]	ANKRD16	54522
ENSG00000204165	3.51094923	5.88153269	2.41728482	2.93570524	-1.8942522	-0.9216284	0.00853713	78	chromosome X open reading frame 65 [Source:HGNC Symbol;Acc:33713]	CXorf65	158830
ENSG00000267673	0.94129024	1.73376418	1.05573066	1.42638188	-2.1626381	-1.1127922	0.0198192	79	ferredoxin 1-like [Source:HGNC Symbol;Acc:30546]	FDX1L	112812
ENSG00000103740	1.82620364	0.68805835	1.67384508	0.79268068	2.29737914	1.19998896	0.02449907	80	acyl-CoA synthetase bubblegum family member 1 [Source:HGNC Symbol;Acc:29567]	ACSBG1	23205
ENSG00000113368	84.0441381	51.7079558	35.7775753	20.3330311	1.78475342	0.83572477	0.00060867	81	lamin B1 [Source:HGNC Symbol;Acc:6637]	LMNB1	4001
ENSG00000184305	3.91953069	5.76283104	3.32835384	3.23872788	-1.9703823	-0.9784756	0.01510037	82	coiled-coil serine-rich protein 1 [Source:HGNC Symbol;Acc:29349]	CCSER1	401145
ENSG00000183092	2.12696971	1.23992314	2.14193977	1.73514014	2.34907644	1.23209366	0.02685411	83	brain-enriched guanylate kinase-associated [Source:HGNC Symbol;Acc:24163]	BEGAIN	57596
ENSG00000143297	21.3607419	29.4039465	16.2248401	21.9547394	-2.2830736	-1.1909774	0.02608132	84	Fc receptor-like 5 [Source:HGNC Symbol;Acc:18508]	FCRL5	83416
ENSG00000159496	9.87684757	5.95374691	5.58946018	3.82238577	1.88939671	0.91792565	0.01161039	85	ral guanine nucleotide dissociation stimulator-like 4 [Source:HGNC Symbol;Acc:31911]	RGL4	266747
ENSG00000132704	7.04790021	9.95149041	5.59009096	4.10985869	-2.1126289	-1.0790394	0.02052278	86	Fc receptor-like 2 [Source:HGNC Symbol;Acc:14875]	FCRL2	79368
ENSG00000183019	30.2779568	18.5707398	18.0573145	9.54112202	1.78679343	0.83737285	0.00230022	87	chromosome 19 open reading frame 59 [Source:HGNC Symbol;Acc:27291]	C19orf59	199675
ENSG00000123700	28.6584499	15.1270763	24.4418571	13.7197523	2.23503337	1.16029637	0.02600689	88	potassium inwardly-rectifying channel, subfamily J, member 2 [Source:HGNC Symbol;Acc:6263]	KCNJ2	3759

ENSG00000213213	1.41099098	2.242539	1.06066861	1.22105006	-1.8892584	-0.91782	0.01281315	89	coiled-coil domain containing 183 [Source:HGNC Symbol;Acc:28236]	CCDC183	84960
ENSG00000133328	1.19784639	2.15504467	2.74218425	3.57448467	-2.4470537	-1.2910457	0.02999283	90	HRAS-like suppressor 2 [Source:HGNC Symbol;Acc:17824]	HRASLS2	54979
ENSG00000165626	4.27747799	2.39118797	3.81975934	2.25684991	2.17276327	1.11953099	0.02416582	91	BEN domain containing 7 [Source:HGNC Symbol;Acc:23514]	BEND7	222389
ENSG00000184557	25.648605	15.7523809	18.431316	16.3303195	2.0201549	1.01446592	0.02102951	92	suppressor of cytokine signaling 3 [Source:HGNC Symbol;Acc:19391]	SOCS3	9021
ENSG00000064763	14.7163511	9.37944599	6.90977666	4.566373	1.77229424	0.82561814	0.00388805	93	fatty acyl CoA reductase 2 [Source:HGNC Symbol;Acc:25531]	FAR2	55711
ENSG00000107317	8.27631674	16.4776172	8.09910964	11.4549206	-1.9212794	-0.9420673	0.01714883	94	prostaglandin D2 synthase 21kDa (brain) [Source:HGNC Symbol;Acc:9592]	PTGDS	5730
ENSG00000137124	2.10260045	3.99484397	1.43756476	2.29573476	-1.8767425	-0.9082307	0.01541465	95	aldehyde dehydrogenase 1 family, member B1 [Source:HGNC Symbol;Acc:407]	ALDH1B1	219
ENSG00000174482	0.86948524	1.9537929	1.21900143	1.88185893	-2.2118143	-1.1452302	0.02829069	96	leucine rich repeat and Ig domain containing 2 [Source:HGNC Symbol;Acc:21207]	LINGO2	158038
ENSG00000173210	11.2254666	5.83777273	9.74995838	4.43850597	1.93949814	0.95568339	0.01994243	97	actin binding LIM protein family, member 3 [Source:HGNC Symbol;Acc:29132]	ABLIM3	22885
ENSG00000115289	4.842331	3.1066065	2.1390566	1.52081505	1.76884171	0.82280495	0.00512155	98	polycomb group ring finger 1 [Source:HGNC Symbol;Acc:17615]	PCGF1	84759
ENSG00000174080	3.93226714	6.63958476	2.46296609	2.8632222	-1.7526323	-0.8095233	0.00411739	99	cathepsin F [Source:HGNC Symbol;Acc:2531]	CTSF	8722
ENSG00000145428	1.31867704	2.8861394	2.35169028	3.04051357	-2.285256	-1.1923558	0.03446731	100	ring finger protein 175 [Source:HGNC Symbol;Acc:27735]	RNF175	285533
ENSG00000198590	1.88904093	3.18322806	1.50016322	1.39450997	-1.8900346	-0.9184127	0.01776856	101	chromosome 3 open reading frame 35 [Source:HGNC Symbol;Acc:24082]	C3orf35	339883
ENSG00000273173	1.26753108	2.07593892	1.33456028	1.54236997	-2.0264398	-1.0189473	0.02474321	102	SNRPN upstream reading frame protein [Source:UniProtKB/Swiss-Prot;Acc:Q9Y675]	SNURF	8926
ENSG00000099256	2.30137583	0.99433086	2.16597437	1.21145427	2.25745572	1.17469769	0.03339738	103	phosphoribosyl transferase domain containing 1 [Source:HGNC Symbol;Acc:23333]	PRTFDC1	56952
ENSG00000105289	1.66895039	2.51117387	1.78622715	1.67107638	-1.9801579	-0.9856155	0.023624	104	tight junction protein 3 [Source:HGNC Symbol;Acc:11829]	TJP3	27134
ENSG00000130584	1.295216	2.01044335	1.68943622	1.40233418	-1.9862486	-0.9900462	0.02470204	105	zinc finger and BTB domain containing 46 [Source:HGNC Symbol;Acc:16094]	ZBTB46	140685

ENSG00000135636	50.4722614	29.5187439	42.8974496	22.4188218	1.8337494	0.87479649	0.01544338	106	dysferlin [Source:HGNC Symbol;Acc:3097]	DYSF	8291
ENSG00000166278	2.58303241	1.07133242	2.86270011	1.0928014	2.26890879	1.18199862	0.03669103	107	complement component 2 [Source:HGNC Symbol;Acc:1248]	C2	717
ENSG00000166428	2.3846649	4.40417469	2.0672633	2.91887033	-1.9302796	-0.9488098	0.02316349	108	phospholipase D family, member 4 [Source:HGNC Symbol;Acc:23792]	PLD4	122618
ENSG00000183762	13.6746814	6.25956642	13.7634768	6.73891384	2.06572238	1.04664637	0.02791048	109	kringle containing transmembrane protein 1 [Source:HGNC Symbol;Acc:17550]	KREMEN1	83999
ENSG00000138002	2.06677017	3.25510335	1.66803652	1.4801638	-1.8685147	-0.9018919	0.01918132	110	intraflagellar transport 172 homolog (Chlamydomonas) [Source:HGNC Symbol;Acc:30391]	IFT172	26160
ENSG00000168970	3.29015088	4.27477549	3.59305661	3.49780596	-2.1185624	-1.0830856	0.02987817	111	JMJD7-PLA2G4B readthrough [Source:HGNC Symbol;Acc:34449]	JMJD7-PLA2G4B	8681
ENSG00000133069	5.46182752	2.89221658	4.68287495	1.75931677	1.8965235	0.92335725	0.02222177	112	transmembrane and coiled-coil domain family 2 [Source:HGNC Symbol;Acc:24239]	TMCC2	9911
ENSG00000181631	179.514037	116.808552	72.2030342	53.8554781	1.72095463	0.78320906	0.00186687	113	purinergic receptor P2Y, G-protein coupled, 13 [Source:HGNC Symbol;Acc:4537]	P2RY13	53829
ENSG00000110104	4.53743907	7.54762303	3.31837865	3.41557414	-1.7635079	-0.8184481	0.00984051	114	coiled-coil domain containing 86 [Source:HGNC Symbol;Acc:28359]	CCDC86	79080
ENSG00000006432	1.75372415	2.73702216	1.60205828	1.80416458	-1.8521334	-0.889188	0.01995593	115	mitogen-activated protein kinase kinase kinase 9 [Source:HGNC Symbol;Acc:6861]	MAP3K9	4293
ENSG00000112796	2.42526118	3.65868884	2.22215448	2.71381759	-1.9966585	-0.9975876	0.02821112	116	ectonucleotide pyrophosphatase/phosphodiesterase 5 (putative) [Source:HGNC Symbol;Acc:13717]	ENPP5	59084
ENSG00000136869	111.013735	68.4058767	49.8085994	22.1048365	1.71190779	0.77560499	0.00079772	117	toll-like receptor 4 [Source:HGNC Symbol;Acc:11850]	TLR4	7099
ENSG00000096006	9.02397511	3.269998	12.3473231	2.41805878	2.18131504	1.12519815	0.03564463	118	cysteine-rich secretory protein 3 [Source:HGNC Symbol;Acc:16904]	CRISP3	10321
ENSG00000122420	3.03199942	1.08443416	3.9966381	2.068486	2.34670176	1.2306345	0.04173279	119	prostaglandin F receptor (FP) [Source:HGNC Symbol;Acc:9600]	PTGFR	5737
ENSG00000134755	35.1341498	18.8838085	22.0780804	9.89570007	1.78873722	0.83894146	0.01549228	120	desmocollin 2 [Source:HGNC Symbol;Acc:3036]	DSC2	1824
ENSG00000091106	81.2649642	52.0815209	38.7303212	23.0992511	1.71069099	0.77457918	0.00151671	121	NLR family, CARD domain containing 4 [Source:HGNC Symbol;Acc:16412]	NLRC4	58484
ENSG00000088726	13.5069604	6.91799916	10.9802557	4.79798429	1.85162229	0.88878983	0.0214962	122	transmembrane protein 40 [Source:HGNC Symbol;Acc:25620]	TMEM40	55287
ENSG00000108387	2.45914292	1.21594839	2.21967527	0.99723127	2.11613143	1.08142923	0.03362343	123	septin 4 [Source:HGNC Symbol;Acc:9165]	SEPT4	5414

ENSG00000205639	2.98418208	1.32652024	3.09672867	1.2783593	2.11938879	1.08364827	0.03519203	124	major facilitator superfamily domain containing 2B [Source:HGNC Symbol;Acc:37207]	MFSD2B	388931
ENSG00000143878	44.0045699	30.769088	24.143957	24.0912416	1.79355804	0.84282443	0.016527	125	ras homolog family member B [Source:HGNC Symbol;Acc:668]	RHOB	388
ENSG00000145685	33.8983491	19.8079182	20.0237949	8.26688737	1.7145984	0.7778707	0.00424244	126	lipoma HMGIC fusion partner-like 2 [Source:HGNC Symbol;Acc:6588]	LHFPL2	10184
ENSG00000127586	2.72417626	3.88921411	2.65265394	2.44325719	-1.8481577	-0.8860878	0.02235552	127	CTF18, chromosome transmission fidelity factor 18 homolog (S. cerevisiae) [Source:HGNC Symbol;Acc:18435]	CHTF18	63922
ENSG00000141736	4.68832009	8.53961103	3.6500515	5.12298671	-1.7860429	-0.8367667	0.01643616	128	v-erb-b2 avian erythroblastic leukemia viral oncogene homolog 2 [Source:HGNC Symbol;Acc:3430]	ERBB2	2064
ENSG00000140022	7.37554356	4.23916814	3.48738035	2.28796943	1.7308756	0.79150204	0.00843509	129	stonin 2 [Source:HGNC Symbol;Acc:30652]	STON2	85439
ENSG00000175544	2.49910968	4.12435186	2.38840611	2.34993824	-1.9862484	-0.9900461	0.03184315	130	calcium binding protein 4 [Source:HGNC Symbol;Acc:1386]	CABP4	57010
ENSG00000196466	5.21735433	3.5979144	3.44958739	2.9440097	1.89787882	0.92438788	0.02760692	131	zinc finger protein 799 [Source:HGNC Symbol;Acc:28071]	ZNF799	90576
ENSG00000049247	1.28811543	3.10275865	1.87257348	4.96082894	-2.1979907	-1.1361853	0.04148761	132	urotensin 2 [Source:HGNC Symbol;Acc:12636]	UTS2	10911
ENSG00000128536	1.39851935	2.42510497	1.75664671	1.6944258	-2.0871102	-1.0615068	0.03685996	133	cadherin-related family member 3 [Source:HGNC Symbol;Acc:26308]	CDHR3	222256
ENSG00000100336	1.44931939	0.45537549	1.76226256	0.58452344	2.15922208	1.11051164	0.04060508	134	apolipoprotein L, 4 [Source:HGNC Symbol;Acc:14867]	APOL4	80832
ENSG00000170425	1.72509041	0.70650048	1.80916063	0.80625752	2.09322824	1.06572963	0.03790195	135	adenosine A2b receptor [Source:HGNC Symbol;Acc:264]	ADORA2B	136
ENSG00000119782	3.4080482	2.11405462	2.38990155	1.68068728	1.87913406	0.91006799	0.02808258	136	FK506 binding protein 1B, 12.6 kDa [Source:HGNC Symbol;Acc:3712]	FKBP1B	2281
ENSG00000259207	21.6409052	12.1093108	17.6766007	8.89944599	1.85218187	0.88922577	0.02675079	137	integrin, beta 3 (platelet glycoprotein IIIa, antigen CD61) [Source:HGNC Symbol;Acc:6156]	ITGB3	3690
ENSG00000129682	1.45494917	0.97555868	1.68712344	1.4478799	2.25749279	1.17472138	0.04797567	138	fibroblast growth factor 13 [Source:HGNC Symbol;Acc:3670]	FGF13	2258
ENSG00000112290	3.2014723	1.76431087	2.93569032	1.45704018	1.95058326	0.96390558	0.03535408	139	WAS protein family, member 1 [Source:HGNC Symbol;Acc:12732]	WASF1	8936
ENSG00000123405	90.1119788	56.2310342	41.5083722	28.0611892	1.69760268	0.76349884	0.006079	140	nuclear factor, erythroid 2 [Source:HGNC Symbol;Acc:7780]	NFE2	4778
ENSG00000121858	258.501947	161.414871	177.697873	64.8554337	1.69069256	0.75761434	0.00515858	141	tumor necrosis factor (ligand) superfamily, member 10 [Source:HGNC Symbol;Acc:11925]	TNFSF10	8743

ENSG00000117115	62.8748958	41.0268596	41.7686237	28.8705671	1.76239879	0.81754041	0.01858641	142	peptidyl arginine deiminase, type II [Source:HGNC Symbol;Acc:18341]	PADI2	11240
ENSG00000135838	30.6690215	20.1231182	14.7774998	8.78572603	1.6881883	0.75547583	0.00486751	143	N-acetylneuraminate pyruvate lyase (dihydrodipicolinate synthase) [Source:HGNC Symbol;Acc:16781]	NPL	80896
ENSG00000159231	2.05862906	3.65823331	1.80197702	3.19143214	-2.0112441	-1.0080882	0.04048859	144	carbonyl reductase 3 [Source:HGNC Symbol;Acc:1549]	CBR3	874
ENSG00000110400	5.40970525	3.5821801	3.42801642	2.50801004	1.78669048	0.83728973	0.02250972	145	poliovirus receptor-related 1 (herpesvirus entry mediator C) [Source:HGNC Symbol;Acc:9706]	PVRL1	5818
ENSG00000084731	3.87687317	2.33151671	1.72930182	1.24607994	1.7516628	0.80872508	0.01900925	146	kinesin family member 3C [Source:HGNC Symbol;Acc:6321]	KIF3C	3797
ENSG00000173369	2.13994639	1.01557515	2.35158245	1.03427376	2.12299098	1.08609824	0.04617223	147	complement component 1, q subcomponent, B chain [Source:HGNC Symbol;Acc:1242]	C1QB	713
ENSG00000134504	1.1896011	1.78010977	1.14921447	1.11478183	-1.8281429	-0.8703788	0.02857306	148	potassium channel tetramerization domain containing 1 [Source:HGNC Symbol;Acc:18249]	KCTD1	284252
ENSG00000102362	9.28290934	6.03921723	5.91627822	4.45481336	1.85167244	0.88882891	0.03071697	149	synaptotagmin-like 4 [Source:HGNC Symbol;Acc:15588]	SYTL4	94121
ENSG00000005961	49.6926193	26.7943422	42.5054373	17.2981723	1.81514885	0.86008786	0.02850865	150	integrin, alpha 2b (platelet glycoprotein IIb of IIb/IIIa complex, antigen CD41) [Source:HGNC Symbol;Acc:6138]	ITGA2B	3674
ENSG00000118690	3.18618506	5.21987277	1.99741988	2.4564275	-1.7073782	-0.7717826	0.01189144	151	armadillo repeat containing 2 [Source:HGNC Symbol;Acc:23045]	ARMC2	84071
ENSG00000086506	1.81613477	0.72622554	1.65731714	0.91288135	2.12231588	1.0856394	0.04813523	152	hemoglobin, theta 1 [Source:HGNC Symbol;Acc:4833]	HBQ1	3049
ENSG00000121316	141.932963	96.2601991	57.5058184	38.7709516	1.65346942	0.72549637	0.00093045	153	phospholipase B domain containing 1 [Source:HGNC Symbol;Acc:26215]	PLBD1	79887
ENSG00000180855	2.17884639	0.893209	2.46338035	1.07244211	2.06460273	1.0458642	0.04669355	154	zinc finger protein 443 [Source:HGNC Symbol;Acc:20878]	ZNF443	10224
ENSG00000243772	1.4869141	2.49550702	2.00737799	2.18843273	-2.0108715	-1.0078209	0.045529	155	killer cell immunoglobulin-like receptor, two domains, long cytoplasmic tail, 3 [Source:HGNC Symbol;Acc:6331]	KIR2DL3	100653050
ENSG00000179088	2.91737909	4.00667073	2.0349239	1.94267066	-1.7843964	-0.8354361	0.02655616	156	chromosome 12 open reading frame 42 [Source:HGNC Symbol;Acc:24729]	C12orf42	374470
ENSG00000019991	12.6791554	7.72231835	7.21258775	2.7994412	1.67015848	0.73998501	0.00666345	157	hepatocyte growth factor (hepapoietin A; scatter factor) [Source:HGNC Symbol;Acc:4893]	HGF	3082
ENSG00000121807	74.5676063	44.1023307	44.7617126	19.142979	1.71471942	0.77797253	0.01589276	158	chemokine (C-C motif) receptor 2 [Source:HGNC Symbol;Acc:1603]	CCR2	729230

ENSG00000167618	2.35823975	5.364106	2.24496644	4.98790329	-1.9441949	-0.9591728	0.0427247	159	leukocyte-associated immunoglobulin-like receptor 2 [Source:HGNC Symbol;Acc:6478]	LAIR2	3904
ENSG00000088826	12.2886601	7.17583734	7.51078906	4.24363536	1.78447182	0.83549712	0.02749342	161	spermine oxidase [Source:HGNC Symbol;Acc:15862]	SMOX	54498
ENSG00000137198	6.45791701	2.72927674	8.03249283	2.51460744	2.09359059	1.06597935	0.04991937	162	guanosine monophosphate reductase [Source:HGNC Symbol;Acc:4376]	GMPR	2766
ENSG00000176945	4.5231017	7.09247189	3.4754074	4.80353315	-1.9551097	-0.9672496	0.04665072	163	mucin 20, cell surface associated [Source:HGNC Symbol;Acc:23282]	MUC20	200958
ENSG00000157985	5.38925704	9.57938659	3.22752209	5.68101111	-1.7101039	-0.774084	0.01667398	164	ArfGAP with GTPase domain, ankyrin repeat and PH domain 1 [Source:HGNC Symbol;Acc:16922]	AGAP1	116987
ENSG00000171766	1.62356188	2.71243071	1.48288981	1.80930837	-1.9032179	-0.9284408	0.0437223	165	glycine amidinotransferase (L-arginine:glycine amidinotransferase) [Source:HGNC Symbol;Acc:4175]	GATM	2628
ENSG00000181027	3.30810348	5.1714742	2.05406697	2.54207616	-1.7501652	-0.8074911	0.02565895	166	fukutin related protein [Source:HGNC Symbol;Acc:17997]	FKRP	79147
ENSG00000139187	55.4478071	87.6401303	35.5337227	40.8127921	-1.6621248	-0.7330287	0.00749107	167	killer cell lectin-like receptor subfamily G, member 1 [Source:HGNC Symbol;Acc:6380]	KLRG1	10219
ENSG00000169704	14.0005645	7.88153354	12.4469566	6.48622412	1.81046426	0.8563597	0.03507545	169	glycoprotein IX (platelet) [Source:HGNC Symbol;Acc:4444]	GP9	2815
ENSG00000205784	5.09389671	7.81023843	3.7408967	3.17393196	-1.6660797	-0.7364574	0.01038373	170	arrestin domain containing 5 [Source:HGNC Symbol;Acc:31407]	ARRDC5	645432
ENSG00000243452	1.70076105	2.72615528	2.11921831	1.75231532	-1.871438	-0.9041473	0.04397806	172	neuroblastoma breakpoint family, member 15 [Source:HGNC Symbol;Acc:28791]	NBPF15	284565
ENSG00000162591	3.02627117	5.24050842	2.41594653	3.77015034	-1.772173	-0.8255194	0.03063794	173	multiple EGF-like-domains 6 [Source:HGNC Symbol;Acc:3232]	MEGF6	1953
ENSG00000134827	10.2799974	4.86521236	9.93159087	3.7353379	1.82013488	0.86404536	0.03991176	174	transcobalamin I (vitamin B12 binding protein, R binder family) [Source:HGNC Symbol;Acc:11652]	TCN1	6947
ENSG00000106686	1.49541178	2.44937012	1.53885825	1.7824687	-1.9160046	-0.938101	0.0485312	175	spermatogenesis associated 6-like [Source:HGNC Symbol;Acc:25472]	SPATA6L	55064
ENSG00000128218	6.60221171	8.2508473	5.32599709	4.05243778	-1.8712128	-0.9039736	0.04501153	176	pre-B lymphocyte 3 [Source:HGNC Symbol;Acc:12710]	VPREB3	29802
ENSG00000007402	2.4664524	4.11678023	2.06418512	2.42041625	-1.7881062	-0.8384324	0.03670148	177	calcium channel, voltage-dependent, alpha 2/delta subunit 2 [Source:HGNC Symbol;Acc:1400]	CACNA2D2	9254
ENSG00000148346	19.8934846	11.7859283	21.0710475	12.6163618	1.81852394	0.86276792	0.04090362	178	lipocalin 2 [Source:HGNC Symbol;Acc:6526]	LCN2	3934

ENSG00000038945	11.5648194	6.59514692	8.17063424	3.50485783	1.80303765	0.85042953	0.04009196	179	macrophage scavenger receptor 1 [Source:HGNC Symbol;Acc:7376]	MSR1	4481
ENSG00000142089	217.282028	121.799693	346.722202	147.595637	1.87394085	0.90607541	0.04735492	180	interferon induced transmembrane protein 3 [Source:HGNC Symbol;Acc:5414]	IFITM3	10410
ENSG00000181652	1.9507183	3.08390137	1.7099679	1.91145962	-1.8219981	-0.8655215	0.04173834	181	autophagy related 9B [Source:HGNC Symbol;Acc:21899]	ATG9B	285973
ENSG00000072952	44.682869	28.5054928	26.4011882	19.6615466	1.68918189	0.75632469	0.01944012	182	murine retrovirus integration site 1 homolog [Source:HGNC Symbol;Acc:7237]	MRVI1	10335
ENSG00000244482	49.7728613	31.4190119	23.3642041	13.5551172	1.64380513	0.71703928	0.01018898	183	leukocyte immunoglobulin-like receptor, subfamily A (with TM domain), member 6 [Source:HGNC Symbol;Acc:15495]	LILRA6	
ENSG00000090920	3.12274864	5.25649658	2.92230313	3.97445754	-1.8772554	-0.9086249	0.04918551	184	Fc fragment of IgG binding protein [Source:HGNC Symbol;Acc:13572]	FCGBP	8857
ENSG00000177324	12.5327459	6.870657	9.72750811	5.37797698	1.86730155	0.90095493	0.04822578	185	BEN domain containing 2 [Source:HGNC Symbol;Acc:28509]	BEND2	139105
ENSG00000065534	26.3408943	17.702701	13.1190116	13.4831022	1.72794566	0.78905785	0.02792089	186	myosin light chain kinase [Source:HGNC Symbol;Acc:7590]	MYLK	4638
ENSG00000239961	7.52346241	11.8265183	5.01770746	4.57184529	-1.7335979	-0.7937693	0.02983518	188	leukocyte immunoglobulin-like receptor, subfamily A (with TM domain), member 4 [Source:HGNC Symbol;Acc:15503]	LILRA4	23547
ENSG00000183837	6.76058702	11.0875087	4.53865286	7.30194199	-1.7422733	-0.800971	0.0322525	190	paraneoplastic Ma antigen 3 [Source:HGNC Symbol;Acc:18742]	PNMA3	29944
ENSG00000174428	3.72703064	5.91223102	1.89781859	2.48358362	-1.6922604	-0.7589516	0.02360736	191	GTF2I repeat domain containing 2B [Source:HGNC Symbol;Acc:33125]	GTF2IRD2B	389524
ENSG00000170456	6.50741858	8.85419453	4.94889741	3.9131633	-1.8386748	-0.8786663	0.04923825	193	DENN/MADD domain containing 5B [Source:HGNC Symbol;Acc:28338]	DENND5B	160518
ENSG00000161911	20.5383508	11.9007854	17.1409107	8.59598265	1.74680258	0.80471657	0.03810057	197	triggering receptor expressed on myeloid cells-like 1 [Source:HGNC Symbol;Acc:20434]	TREML1	340205
ENSG00000157240	5.04932234	2.98505998	3.59842276	2.15685841	1.74575618	0.80385208	0.03795624	198	frizzled family receptor 1 [Source:HGNC Symbol;Acc:4038]	FZD1	8321
ENSG00000116133	3.54623404	5.67278901	2.83752953	3.34071663	-1.7372144	-0.7967758	0.03530637	199	24-dehydrocholesterol reductase [Source:HGNC Symbol;Acc:2859]	DHCR24	1718
ENSG00000124145	2.51152651	1.79684796	1.62547273	1.41016507	1.78288742	0.83421561	0.04535139	201	syndecan 4 [Source:HGNC Symbol;Acc:10661]	SDC4	6385
ENSG00000034053	24.6745498	36.516268	11.4810918	12.0799498	-1.6009528	-0.6789308	0.00638464	203	amyloid beta (A4) precursor protein-binding, family A, member 2 [Source:HGNC Symbol;Acc:579]	APBA2	321

ENSG00000204420	73.1089622	39.0413615	60.6926909	27.0505283	1.75056908	0.80782399	0.04144863	204	chromosome 6 open reading frame 25 [Source:HGNC Symbol;Acc:13937]	C6orf25	80739
ENSG00000007264	13.6395654	22.6186035	8.22681448	11.1835492	-1.6338657	-0.7082894	0.0147812	206	megakaryocyte-associated tyrosine kinase [Source:HGNC Symbol;Acc:6906]	MATK	4145
ENSG00000073536	2.07569409	3.38633143	1.70444416	2.05356711	-1.7306839	-0.7913422	0.04052496	209	notchless homolog 1 (Drosophila) [Source:HGNC Symbol;Acc:19889]	NLE1	54475
ENSG00000115828	43.5602511	28.0858301	29.1980873	15.8250381	1.66487562	0.7354144	0.02589148	211	glutaminyl-peptide cyclotransferase [Source:HGNC Symbol;Acc:9753]	QPCT	25797
ENSG00000147180	4.20340453	6.29026249	2.91453761	3.21464466	-1.6842608	-0.7521155	0.02978651	212	zinc finger protein 711 [Source:HGNC Symbol;Acc:13128]	ZNF711	7552
ENSG00000206561	2.68304638	4.40882703	1.8504564	2.48130614	-1.7401512	-0.7992127	0.04373295	213	collagen-like tail subunit (single strand of homotrimer) of asymmetric acetylcholinesterase [Source:HGNC Symbol;Acc:2226]	COLQ	8292
ENSG00000136514	10.0063101	7.49076916	7.92487452	4.58980032	1.64762592	0.72038873	0.02232407	216	receptor (chemosensory) transporter protein 4 [Source:HGNC Symbol;Acc:23992]	RTP4	64108
ENSG00000184205	28.3107724	46.264976	13.5150549	24.5374875	-1.6171585	-0.6934611	0.0151079	218	TSPY-like 2 [Source:HGNC Symbol;Acc:24358]	TSPYL2	64061
ENSG00000142173	7.17601057	13.8371288	5.30538021	8.73062204	-1.6674198	-0.7376174	0.0285684	220	collagen, type VI, alpha 2 [Source:HGNC Symbol;Acc:2212]	COL6A2	1292
ENSG00000170345	810.323071	579.893104	430.774594	344.159859	1.67280221	0.74226687	0.03144719	223	FBJ murine osteosarcoma viral oncogene homolog [Source:HGNC Symbol;Acc:3796]	FOS	2353
ENSG00000103056	7.77729529	14.4282102	4.68326411	10.7798592	-1.6745111	-0.7437399	0.03212359	226	sphingomyelin phosphodiesterase 3, neutral membrane (neutral sphingomyelinase II) [Source:HGNC Symbol;Acc:14240]	SMPD3	55512
ENSG00000166262	2.99091691	5.18822629	1.75307736	2.63501583	-1.6428795	-0.7162267	0.02648147	227	family with sequence similarity 227, member B [Source:HGNC Symbol;Acc:26543]	FAM227B	196951
ENSG00000049246	4.17586647	7.42787982	2.1971192	3.26961252	-1.6179602	-0.6941761	0.01996194	229	period circadian clock 3 [Source:HGNC Symbol;Acc:8847]	PER3	8863
ENSG00000161920	11.7062711	8.27132941	5.16859498	3.41179015	1.57156494	0.65220189	0.00956672	232	mediator complex subunit 11 [Source:HGNC Symbol;Acc:32687]	MED11	400569
ENSG00000091490	55.9354231	83.233007	26.9308595	21.3674541	-1.5583835	-0.6400503	0.00568374	233	sel-1 suppressor of lin-12-like 3 (C. elegans) [Source:HGNC Symbol;Acc:29108]	SEL1L3	23231
ENSG00000119862	21.3595713	11.9427089	14.8172556	5.58457664	1.66146911	0.73245947	0.03347925	234	lectin, galactoside-binding-like [Source:HGNC Symbol;Acc:25012]	LGALSL	29094

ENSG00000051128	36.9841917	25.0527018	18.3395907	9.29282427	1.56725536	0.64824026	0.01030171	235	homer homolog 3 (Drosophila) [Source:HGNC Symbol;Acc:17514]	HOMER3	9454
ENSG00000130150	48.1774956	33.9205793	21.4614371	14.7678971	1.57156782	0.65220453	0.01194046	236	motile sperm domain containing 2 [Source:HGNC Symbol;Acc:28381]	MOSPD2	158747
ENSG00000157168	11.4164105	7.06787329	5.86334167	3.84818323	1.64233404	0.71574759	0.0287478	237	neuregulin 1 [Source:HGNC Symbol;Acc:7997]	NRG1	3084
ENSG00000176473	6.39637203	4.48714065	3.3009033	2.39842937	1.65809523	0.72952687	0.03466334	240	WD repeat domain 25 [Source:HGNC Symbol;Acc:21064]	WDR25	79446
ENSG00000221909	10.5849999	6.50426762	6.05933355	3.0566423	1.62835606	0.70341619	0.02602273	241	family with sequence similarity 200, member A [Source:HGNC Symbol;Acc:25401]	FAM200A	221786
ENSG00000132386	3.10140847	4.79767205	2.06415677	2.42895337	-1.6637961	-0.7344786	0.03877525	243	serpin peptidase inhibitor, clade F (alpha-2 antiplasmin, pigment epithelium derived factor), member 1 [Source:HGNC Symbol;Acc:8824]	SERPINF1	5176
ENSG00000150681	175.239648	121.791	76.7801216	69.7256025	1.580288	0.6601875	0.01648641	244	regulator of G-protein signaling 18 [Source:HGNC Symbol;Acc:14261]	RGS18	64407
ENSG00000174125	103.387487	70.744484	44.8593189	30.2838379	1.57067462	0.65138435	0.01446207	246	toll-like receptor 1 [Source:HGNC Symbol;Acc:11847]	TLR1	7096
ENSG00000160408	3.91430362	6.05918593	2.0949519	2.36261055	-1.6081819	-0.6854306	0.02300711	247	ST6 (alpha-N-acetyl-neuraminyl-2,3-beta-galactosyl-1,3)-N-acetylgalactosaminide alpha-2,6-sialyltransferase 6 [Source:HGNC Symbol;Acc:23364]	ST6GALNAC6	30815
ENSG00000110987	17.6920932	24.0278712	11.3521202	8.27289778	-1.6597139	-0.7309346	0.03867721	248	B-cell CLL/lymphoma 7A [Source:HGNC Symbol;Acc:1004]	BCL7A	605
ENSG00000156026	11.7231716	8.74345441	4.36727165	4.020087	1.56485518	0.64602915	0.01353478	249	mitochondrial calcium uniporter [Source:HGNC Symbol;Acc:23526]	MCU	90550
ENSG00000135378	43.7836169	27.8635055	25.3288312	15.1915541	1.62752636	0.70268091	0.02813798	250	proline rich Gla (G-carboxyglutamic acid) 4 (transmembrane) [Source:HGNC Symbol;Acc:30799]	PRRG4	79056
ENSG00000187554	31.5356912	20.965846	13.0757914	6.18766904	1.53685334	0.6199795	0.00528705	253	toll-like receptor 5 [Source:HGNC Symbol;Acc:11851]	TLR5	7100
ENSG00000185697	110.767946	170.977388	54.7802859	66.8853176	-1.5748106	-0.6551783	0.01680479	257	v-myb avian myeloblastosis viral oncogene homolog-like 1 [Source:HGNC Symbol;Acc:7547]	MYBL1	4603
ENSG00000131876	19.840839	32.1107292	10.410564	15.9682213	-1.5905384	-0.6695152	0.02176864	258	small nuclear ribonucleoprotein polypeptide A' [Source:HGNC Symbol;Acc:11152]	SNRPA1	6627
ENSG00000185453	5.2349831	8.58612382	2.25111659	3.69402488	-1.5983045	-0.6765423	0.02451731	259	chromosome 19 open reading frame 68 [Source:HGNC Symbol;Acc:34495]	C19orf68	374920

ENSG00000165795	16.6090999	26.0529412	8.15037633	12.312138	-1.5800044	-0.6599286	0.02004919	261	NDRG family member 2 [Source:HGNC Symbol;Acc:14460]	NDRG2	57447
ENSG00000168209	17.1562924	32.0306424	10.7291127	20.6406497	-1.6364363	-0.7105575	0.03248615	262	DNA-damage-inducible transcript 4 [Source:HGNC Symbol;Acc:24944]	DDIT4	54541
ENSG00000120896	4.00746617	5.93872342	2.87182799	2.15100887	-1.6355731	-0.7097963	0.03291039	263	sorbin and SH3 domain containing 3 [Source:HGNC Symbol;Acc:30907]	SORBS3	10174
ENSG00000111817	69.6562047	47.8762958	22.6032381	11.9441669	1.52363533	0.60751765	0.00308573	268	dermatan sulfate epimerase [Source:HGNC Symbol;Acc:21144]	DSE	29940
ENSG00000151458	23.6065651	15.5370154	12.1691713	6.24703838	1.56339469	0.64468204	0.01656379	269	ankyrin repeat domain 50 [Source:HGNC Symbol;Acc:29223]	ANKRD50	57182
ENSG00000100385	28.3102635	42.4996243	13.8189013	15.372572	-1.5446305	-0.6272617	0.01441302	273	interleukin 2 receptor, beta [Source:HGNC Symbol;Acc:6009]	IL2RB	3560
ENSG00000132357	27.2754571	19.7583817	9.71762324	7.48448556	1.53158667	0.61502701	0.00793189	274	caspase recruitment domain family, member 6 [Source:HGNC Symbol;Acc:16394]	CARD6	84674
ENSG00000150337	13.3326953	7.28750547	10.5999066	2.94269979	1.64013972	0.71381872	0.04058426	276	Fc fragment of IgG, high affinity Ia, receptor (CD64) [Source:HGNC Symbol;Acc:3613]	FCGR1A	2210
ENSG00000243477	3.91597923	6.5680549	1.73946516	3.22908125	-1.6412243	-0.7147724	0.04087026	278	N-acetyltransferase 6 (GCN5-related) [Source:HGNC Symbol;Acc:30252]	NAT6	24142
ENSG00000143546	2465.51583	1656.55143	1297.67937	714.381101	1.57721643	0.65738064	0.0231441	279	S100 calcium binding protein A8 [Source:HGNC Symbol;Acc:10498]	S100A8	6279
ENSG00000061918	21.3826299	11.6646619	16.1664807	7.59280475	1.66369192	0.7343883	0.04884874	280	guanylate cyclase 1, soluble, beta 3 [Source:HGNC Symbol;Acc:4687]	GUCY1B3	2983
ENSG00000152213	39.823634	28.2686115	16.1805834	11.6841605	1.53896268	0.62195825	0.01373718	282	ADP-ribosylation factor-like 11 [Source:HGNC Symbol;Acc:24046]	ARL11	115761
ENSG00000173511	6.77516785	9.84781346	3.53153188	3.12693976	-1.5943718	-0.6729881	0.02864731	283	vascular endothelial growth factor B [Source:HGNC Symbol;Acc:12681]	VEGFB	7423
ENSG00000114374	6.7972435	38.6139991	19.981384	63.3033714	-1.6421049	-0.7155463	0.04450592	284	ubiquitin specific peptidase 9, Y-linked [Source:HGNC Symbol;Acc:12633]	USP9Y	8287
ENSG00000135469	5.30559615	7.28499867	3.3434342	3.16302648	-1.6387439	-0.7125904	0.0451664	289	coenzyme Q10 homolog A (S. cerevisiae) [Source:HGNC Symbol;Acc:26515]	COQ10A	93058
ENSG00000120306	18.2579922	12.1354378	12.1044144	7.1635239	1.61293512	0.68968841	0.03914476	291	cysteine-rich transmembrane module containing 1 [Source:HGNC Symbol;Acc:30239]	CYSTM1	84418
ENSG00000155906	2.62160759	3.57674872	1.71230737	1.17252849	-1.571037	-0.6517171	0.02659697	293	required for meiotic nuclear division 1 homolog (S. cerevisiae) [Source:HGNC Symbol;Acc:21176]	RMND1	55005

ENSG00000103043	4.44549012	7.10988896	2.38914114	3.02249928	-1.5652055	-0.6463521	0.02477561	295	Vac14 homolog (S. cerevisiae) [Source:HGNC Symbol;Acc:25507]	VAC14	55697
ENSG00000187116	104.045221	70.2966498	42.239342	26.5717511	1.5462572	0.62878032	0.02055156	296	leukocyte immunoglobulin-like receptor, subfamily A (with TM domain), member 5 [Source:HGNC Symbol;Acc:16309]	LILRA5	353514
ENSG00000112394	8.19945489	11.0257792	5.4070818	5.94357125	-1.6231355	-0.6987834	0.04202126	300	solute carrier family 16 (aromatic amino acid transporter), member 10 [Source:HGNC Symbol;Acc:17027]	SLC16A10	117247
ENSG00000110002	7.51084767	5.47948147	4.49709209	2.93431529	1.62009345	0.69607703	0.04310898	301	von Willebrand factor A domain containing 5A [Source:HGNC Symbol;Acc:6658]	VWA5A	4013
ENSG00000166313	9.53755478	14.0663714	5.74126283	7.53593437	-1.5446764	-0.6273046	0.02102463	302	amyloid beta (A4) precursor protein-binding, family B, member 1 (Fe65) [Source:HGNC Symbol;Acc:581]	APBB1	322
ENSG00000159128	87.1308586	61.5111961	38.0060268	29.7920466	1.54181638	0.62463096	0.02052065	303	interferon gamma receptor 2 (interferon gamma transducer 1) [Source:HGNC Symbol;Acc:5440]	IFNGR2	3460
ENSG00000255103	4.51133199	7.52308772	3.18651485	3.3770128	-1.5475422	-0.6299787	0.02263663	308	KIAA0754 [Source:HGNC Symbol;Acc:29111]	KIAA0754	643314
ENSG00000115271	235.316792	158.291589	121.872554	80.5028847	1.56835213	0.64924951	0.03145499	312	grancalcin, EF-hand calcium binding protein [Source:HGNC Symbol;Acc:15990]	GCA	25801
ENSG00000138050	9.80537397	14.9143238	4.11025832	5.49596194	-1.5167786	-0.6010105	0.01634779	321	THUMP domain containing 2 [Source:HGNC Symbol;Acc:14890]	THUMPD2	80745
ENSG00000163492	18.6346152	25.9556014	8.95070879	9.76644139	-1.5135961	-0.5979803	0.01652254	326	coiled-coil domain containing 141 [Source:HGNC Symbol;Acc:26821]	CCDC141	285025
ENSG00000204475	4.85922251	8.535486	3.39277696	5.39701742	-1.5967406	-0.6751299	0.04891326	331	natural cytotoxicity triggering receptor 3 [Source:HGNC Symbol;Acc:19077]	NCR3	259197
ENSG00000188313	82.9689014	54.7075214	56.8596649	24.4455005	1.54118718	0.62404209	0.02989331	332	phospholipid scramblase 1 [Source:HGNC Symbol;Acc:9092]	PLSCR1	5359
ENSG00000116729	16.937451	10.6771637	8.23035171	4.46097752	1.5616774	0.64309646	0.03881434	334	wntless Wnt ligand secretion mediator [Source:HGNC Symbol;Acc:30238]	WLS	79971
ENSG00000172716	45.052851	32.3151012	16.9886673	12.5036582	1.4981699	0.58320124	0.01423666	337	schlafen family member 11 [Source:HGNC Symbol;Acc:26633]	SLFN11	91607
ENSG00000170677	10.8048694	7.31150662	4.55317547	4.46257931	1.54914087	0.63146834	0.03549386	338	suppressor of cytokine signaling 6 [Source:HGNC Symbol;Acc:16833]	SOCS6	9306
ENSG00000140090	35.7045303	25.8065194	14.9332647	11.4677049	1.51137362	0.59586034	0.02121747	346	solute carrier family 24 (sodium/potassium/calcium exchanger), member 4 [Source:HGNC Symbol;Acc:10978]	SLC24A4	123041

ENSG00000168461	297.207186	213.635119	99.459607	53.1618344	1.47272686	0.55848988	0.00478777	356	RAB31, member RAS oncogene family [Source:HGNC Symbol;Acc:9771]	RAB31	11031
ENSG00000105374	93.3430641	144.136073	77.0489734	64.4294049	-1.5339826	-0.6172821	0.03991981	364	natural killer cell group 7 sequence [Source:HGNC Symbol;Acc:7830]	NKG7	4818
ENSG00000130830	68.2735492	48.0623207	24.8035423	13.2745165	1.47937544	0.56498823	0.01497928	369	membrane protein, palmitoylated 1, 55kDa [Source:HGNC Symbol;Acc:7219]	MPP1	4354
ENSG00000181274	183.81874	126.445078	105.243529	78.2684763	1.54355965	0.62626123	0.047174	375	frequently rearranged in advanced T-cell lymphomas 2 [Source:HGNC Symbol;Acc:16048]	FRAT2	23401
ENSG00000164885	10.0540322	7.09757003	4.18057814	2.68159554	1.49357305	0.5787678	0.02173122	376	cyclin-dependent kinase 5 [Source:HGNC Symbol;Acc:1774]	CDK5	1020
ENSG00000110318	10.3821413	15.8522657	4.71590446	5.79955309	-1.4997044	-0.5846782	0.02741362	379	KIAA1377 [Source:HGNC Symbol;Acc:29264]	KIAA1377	57562
ENSG00000169413	75.0419822	56.2864417	25.2715892	19.8061905	1.46852727	0.55437005	0.01263683	385	ribonuclease, RNase A family, k6 [Source:HGNC Symbol;Acc:10048]	RNASE6	6039
ENSG00000163221	244.239562	172.282916	143.504761	99.4561375	1.53531856	0.61853802	0.04895388	394	S100 calcium binding protein A12 [Source:HGNC Symbol;Acc:10489]	S100A12	6283
ENSG00000154814	57.1345885	82.4977946	21.7468209	31.9319033	-1.4799215	-0.5655206	0.02060122	395	oxidoreductase NAD-binding domain containing 1 [Source:HGNC Symbol;Acc:25128]	OXNAD1	92106
ENSG00000164125	61.2741352	45.2083104	30.6554235	24.502882	1.5193786	0.60348141	0.04111363	397	family with sequence similarity 198, member B [Source:HGNC Symbol;Acc:25312]	FAM198B	51313
ENSG00000067365	6.21349167	4.42034374	3.6791952	2.25876824	1.53132495	0.61478046	0.04708719	398	methyltransferase like 22 [Source:HGNC Symbol;Acc:28368]	METTL22	79091
ENSG00000105717	5.51476118	8.32566414	2.53135069	3.63504404	-1.4965288	-0.5816201	0.02996348	401	pre-B-cell leukemia homeobox 4 [Source:HGNC Symbol;Acc:13403]	PBX4	80714
ENSG00000135249	7.48186722	10.7935843	4.02584358	3.34638687	-1.4990552	-0.5840535	0.03197366	402	RAD50 interactor 1 [Source:HGNC Symbol;Acc:21876]	RINT1	60561
ENSG00000204252	17.9369169	26.7458385	9.13512989	9.6526731	-1.5081525	-0.5927823	0.04019804	409	major histocompatibility complex, class II, DO alpha [Source:HGNC Symbol;Acc:4936]	HLA-DOA	3111
ENSG00000172543	33.1670897	53.6822672	18.4753043	26.4235791	-1.5182319	-0.6023922	0.04572636	413	cathepsin W [Source:HGNC Symbol;Acc:2546]	CTSW	1521
ENSG00000174130	74.9404693	53.0144065	36.0230142	20.8339176	1.49447704	0.57964073	0.03155837	414	toll-like receptor 6 [Source:HGNC Symbol;Acc:16711]	TLR6	10333
ENSG00000204516	27.9416945	20.5785485	15.3042513	8.20825267	1.49734574	0.58240739	0.03733068	424	MHC class I polypeptide-related sequence B [Source:HGNC Symbol;Acc:7091]	MICB	4277
ENSG00000163563	1340.16868	965.896201	654.024169	422.081624	1.50348808	0.58831343	0.04186542	426	myeloid cell nuclear differentiation antigen [Source:HGNC Symbol;Acc:7183]	MNDA	4332

ENSG00000186407	112.135305	76.9553083	58.6892066	25.7858179	1.47565671	0.56135713	0.02727134	427	CD300e molecule [Source:HGNC Symbol;Acc:28874]	CD300E	342510
ENSG00000159433	9.0587948	13.0469168	3.95463261	3.39714571	-1.4896308	-0.5749548	0.035198	429	StAR-related lipid transfer (START) domain containing 9 [Source:HGNC Symbol;Acc:19162]	STARD9	57519
ENSG00000198734	47.6303449	33.454639	23.8061553	14.7233728	1.49561323	0.58073713	0.04359124	437	coagulation factor V (proaccelerin, labile factor) [Source:HGNC Symbol;Acc:3542]	F5	2153
ENSG00000114450	26.1794281	19.6217719	8.23831269	5.7181932	1.44202362	0.5280948	0.01451588	441	guanine nucleotide binding protein (G protein), beta polypeptide 4 [Source:HGNC Symbol;Acc:20731]	GNB4	59345
ENSG00000175471	99.7003009	71.8188992	32.5301069	21.408754	1.45413131	0.54015755	0.0215501	442	multiple C2 domains, transmembrane 1 [Source:HGNC Symbol;Acc:26183]	MCTP1	79772
ENSG00000163694	78.6248717	56.3810602	31.5566263	23.6766447	1.4711874	0.55698102	0.03704429	452	RNA binding motif protein 47 [Source:HGNC Symbol;Acc:30358]	RBM47	54502
ENSG00000143207	42.6794657	32.2943489	12.137082	11.1404386	1.44965192	0.53570653	0.02475372	454	ring finger and WD repeat domain 2, E3 ubiquitin protein ligase [Source:HGNC Symbol;Acc:17440]	RFWD2	64326
ENSG00000113532	122.483847	92.0453894	37.4487475	24.6559863	1.42879203	0.51479594	0.01604913	461	ST8 alpha-N-acetyl-neuraminide alpha-2,8-sialyltransferase 4 [Source:HGNC Symbol;Acc:10871]	ST8SIA4	7903
ENSG00000038427	2131.01399	1551.62471	874.16656	495.886877	1.44857946	0.53463883	0.0293655	469	versican [Source:HGNC Symbol;Acc:2464]	VCAN	1462
ENSG00000020633	232.002963	342.845007	99.5939116	109.309603	-1.4341405	-0.5201864	0.02091872	471	runt-related transcription factor 3 [Source:HGNC Symbol;Acc:10473]	RUNX3	864
ENSG00000184083	7.03490439	10.6914481	3.5624352	4.25612442	-1.464172	-0.5500851	0.04132259	475	family with sequence similarity 120C [Source:HGNC Symbol;Acc:16949]	FAM120C	54954
ENSG00000156475	11.7442598	18.4529804	6.88364881	8.18511856	-1.4655506	-0.5514428	0.0452885	479	protein phosphatase 2, regulatory subunit B, beta [Source:HGNC Symbol;Acc:9305]	PPP2R2B	5521
ENSG00000183621	31.9517906	23.4537921	10.1424537	7.02398134	1.44184938	0.52792046	0.02905236	480	zinc finger protein 438 [Source:HGNC Symbol;Acc:21029]	ZNF438	220929
ENSG00000104497	11.7557963	8.63176399	6.17218472	4.04228992	1.46514681	0.55104523	0.04577165	482	sorting nexin 16 [Source:HGNC Symbol;Acc:14980]	SNX16	64089
ENSG00000116701	595.152667	438.576336	232.209188	156.561409	1.4434434	0.52951454	0.03214289	484	neutrophil cytosolic factor 2 [Source:HGNC Symbol;Acc:7661]	NCF2	4688
ENSG00000163162	209.546424	147.968853	100.804645	52.4237078	1.45715112	0.54315051	0.04614167	501	ring finger protein 149 [Source:HGNC Symbol;Acc:23137]	RNF149	284996
ENSG00000104043	22.8347876	17.9768675	8.18245278	5.48855857	1.42233327	0.50825954	0.02649983	522	ATPase, class I, type 8B, member 4 [Source:HGNC Symbol;Acc:13536]	ATP8B4	79895

ENSG00000114541	53.6049724	39.6570703	19.5193562	12.0360893	1.41974033	0.50562709	0.02501227	527	FERM domain containing 4B [Source:HGNC Symbol;Acc:24886]	FRMD4B	23150
ENSG00000025039	29.8048786	21.948042	10.0233207	7.11491991	1.43064119	0.51666189	0.03584449	530	Ras-related GTP binding D [Source:HGNC Symbol;Acc:19903]	RRAGD	58528
ENSG00000107833	13.6257138	19.766413	5.30519309	7.57664338	-1.4383326	-0.5243973	0.04440549	536	nucleophosmin/nucleoplasmin 3 [Source:HGNC Symbol;Acc:7931]	NPM3	10360
ENSG00000005020	272.485263	207.003457	75.4171471	50.8801664	1.40434036	0.48989264	0.02296685	559	src kinase associated phosphoprotein 2 [Source:HGNC Symbol;Acc:15687]	SKAP2	8935
ENSG00000109458	15.7837709	11.6844256	5.1728415	4.2318939	1.43097034	0.51699377	0.04591288	564	GRB2-associated binding protein 1 [Source:HGNC Symbol;Acc:4066]	GAB1	2549
ENSG00000145912	20.33264	29.5808269	8.19197631	9.47081291	-1.4138312	-0.4996098	0.03222501	572	NHP2 ribonucleoprotein [Source:HGNC Symbol;Acc:14377]	NHP2	55651
ENSG00000196954	226.131586	167.410632	80.6230556	44.4460301	1.40868732	0.49435142	0.02964949	575	caspase 4, apoptosis-related cysteine peptidase [Source:HGNC Symbol;Acc:1505]	CASP4	837
ENSG00000132825	34.6782872	26.6824898	10.1707873	10.2733301	1.42738276	0.51337225	0.04699834	576	protein phosphatase 1, regulatory subunit 3D [Source:HGNC Symbol;Acc:9294]	PPP1R3D	5509
ENSG00000133318	156.550268	118.933748	53.1505977	38.6641258	1.41224284	0.49798819	0.04083937	593	reticulon 3 [Source:HGNC Symbol;Acc:10469]	RTN3	10313
ENSG00000108861	54.7291013	40.9451446	17.6960331	10.2140181	1.4023759	0.4878731	0.0363234	606	dual specificity phosphatase 3 [Source:HGNC Symbol;Acc:3069]	DUSP3	1845
ENSG00000235568	130.758374	101.211357	42.7130428	34.2505398	1.40561399	0.49120046	0.04415156	624	NFAT activating protein with ITAM motif 1 [Source:HGNC Symbol;Acc:29872]	NFAM1	150372
ENSG00000055483	55.5042351	80.9379676	19.5724142	28.0126483	-1.3983477	-0.4837231	0.03822072	627	ubiquitin specific peptidase 36 [Source:HGNC Symbol;Acc:20062]	USP36	57602
ENSG00000112182	69.9404805	94.4615175	33.8244856	28.9215802	-1.4032357	-0.4887574	0.04855855	643	BTB and CNC homology 1, basic leucine zipper transcription factor 2 [Source:HGNC Symbol;Acc:14078]	BACH2	60468
ENSG00000156273	120.033315	92.3442717	33.6279123	21.9105284	1.38442914	0.46929122	0.03203301	653	BTB and CNC homology 1, basic leucine zipper transcription factor 1 [Source:HGNC Symbol;Acc:935]	BACH1	100379661
ENSG00000126351	30.8279723	43.7095674	7.8573066	10.748305	-1.3740356	-0.4584194	0.03175709	681	thyroid hormone receptor, alpha [Source:HGNC Symbol;Acc:11796]	THRA	7067
ENSG00000198315	12.7118518	17.9088668	4.91694886	5.11693044	-1.38765	-0.4726438	0.04827355	696	zinc finger with KRAB and SCAN domains 8 [Source:HGNC Symbol;Acc:12983]	ZKSCAN8	7745
ENSG00000120594	197.557581	154.808134	53.1392049	41.1851125	1.36968943	0.45384881	0.04106982	716	plexin domain containing 2 [Source:HGNC Symbol;Acc:21013]	PLXDC2	84898
ENSG00000188921	75.9187974	58.5515133	20.9783794	16.5284438	1.36611702	0.45008107	0.0441609	737	protein tyrosine phosphatase-like A domain containing 2	PTPLAD2	401494

									[Source:HGNC Symbol;Acc:20920]		
ENSG00000135976	85.6851185	116.726873	25.11959	22.7517188	-1.3409934	-0.4233022	0.04899382	849	ankyrin repeat domain 36 [Source:HGNC Symbol;Acc:24079]	ANKRD36	375248
ENSG00000188641	150.047661	118.582397	34.0269824	24.1066827	1.32796782	0.40922018	0.0471273	889	dihydropyrimidine dehydrogenase [Source:HGNC Symbol;Acc:3012]	DPYD	1806

Compared populations:

D2T RA vs HCs (sex-adjusted)

Fold change describes the size of the difference in gene expression (given in mean and standard deviation, sd) between the compared groups. The values are always in relation to the group used as a base level group (reference). P-value describes the reliability of the change in expression value between the compared groups. Results were considered significant with a fold change of 1.2 and a p-value cutoff of 0.05.

Supplementary Material 25 - List of differentially expressed (DE) genes (sex-adjusted)

ID	mean_D2T RA	mean_Normal_RA	sd_D2T RA	sd_Normal_RA	FC	logFC	P.Value	avgRank	Description	geneName	EntrezID
ENSG00000204311	0.87983151	1.65584524	1.31308785	1.09116982	-2.675009	-1.4195437	0.00317972	1	deafness, autosomal recessive 59 [Source:HGNC Symbol;Acc:29502]	DFNB59	494513
ENSG00000142621	0.73377834	2.41499764	1.03310576	3.00033668	-3.0527986	-1.6101324	0.00512654	2	forkhead-associated (FHA) phosphopeptide binding domain 1 [Source:HGNC Symbol;Acc:29408]	FHAD1	114827
ENSG00000135747	1.24928301	2.79792054	1.27236952	2.29679626	-2.6199993	-1.3895664	0.00415177	3	zinc finger protein 670 [Source:HGNC Symbol;Acc:28167]	ZNF670	93474
ENSG00000117479	1.19537976	2.26682122	1.60674683	2.271306	-2.6199335	-1.3895302	0.00589741	4	solute carrier family 19 (thiamine transporter), member 2 [Source:HGNC Symbol;Acc:10938]	SLC19A2	10560
ENSG00000100908	2.47741677	1.0154091	1.71997893	0.80038253	2.48201642	1.31151266	0.00354667	5	ER membrane protein complex subunit 9 [Source:HGNC Symbol;Acc:20273]	EMC9	51016
ENSG00000160307	2.14566383	5.62709609	2.63252675	5.36455259	-3.0739837	-1.6201095	0.01356327	6	S100 calcium binding protein B [Source:HGNC Symbol;Acc:10500]	S100B	6285
ENSG00000111249	0.97085657	1.82480312	1.17246116	1.1228518	-2.4969809	-1.3201848	0.00831636	7	cut-like homeobox 2 [Source:HGNC Symbol;Acc:19347]	CUX2	23316
ENSG00000187990	1.49072776	2.50411165	2.27392002	2.0680503	-2.5741193	-1.3640789	0.01060714	8	histone cluster 1, H2bg [Source:HGNC Symbol;Acc:4746]	HIST1H2BG	8347
ENSG00000133424	1.80617456	3.2231488	1.65816888	2.57407533	-2.4651946	-1.3017015	0.00903484	9	like-glycosyltransferase [Source:HGNC Symbol;Acc:6511]	LARGE	9215
ENSG00000197705	2.54212133	3.91504597	2.47378779	2.58855166	-2.4326597	-1.2825345	0.01153142	10	kelch-like family member 14 [Source:HGNC Symbol;Acc:29266]	KLHL14	57565
ENSG00000149554	2.3001236	4.28621414	1.92295986	3.43619645	-2.3491075	-1.2321128	0.00786626	11	checkpoint kinase 1 [Source:HGNC Symbol;Acc:1925]	CHEK1	1111
ENSG00000066923	1.29669089	2.51996913	1.0903448	1.84259227	-2.2717141	-1.1837813	0.00707485	12	stromal antigen 3 [Source:HGNC Symbol;Acc:11356]	STAG3	10734
ENSG00000163806	3.33838499	4.58976602	4.72500558	3.26542269	-2.3750231	-1.2479416	0.01249212	13	speedy/RINGO cell cycle regulator family member A	SPDYA	245711

									[Source:HGNC Symbol;Acc:30613]		
ENSG00000163687	1.45245095	2.6676476	1.74334512	2.56835117	-2.4702794	-1.3046742	0.01569027	14	deoxyribonuclease I-like 3 [Source:HGNC Symbol;Acc:2959]	DNASE1L3	1776
ENSG00000213213	1.41099098	2.77477579	1.06066861	1.59410026	-2.1819358	-1.1256087	0.00352944	15	coiled-coil domain containing 183 [Source:HGNC Symbol;Acc:28236]	CCDC183	84960
ENSG00000159403	0.88174368	1.6661825	1.29724938	1.29809756	-2.4875379	-1.3147185	0.01879819	16	complement component 1, r subcomponent [Source:HGNC Symbol;Acc:1246]	C1R	715
ENSG00000079482	1.96546151	3.08665343	2.04352536	1.8663484	-2.2729059	-1.184538	0.01202977	17	oligophrenin 1 [Source:HGNC Symbol;Acc:8148]	OPHN1	4983
ENSG00000101977	2.30091172	3.81879717	1.87198422	1.68603143	-2.2522794	-1.1713858	0.0076608	18	MCF.2 cell line derived transforming sequence [Source:HGNC Symbol;Acc:6940]	MCF2	4168
ENSG00000225614	1.3328699	2.53280295	1.19368173	1.72256276	-2.1946508	-1.1339914	0.00655017	19	zinc finger protein 469 [Source:HGNC Symbol;Acc:23216]	ZNF469	84627
ENSG00000137269	1.95119329	3.4304291	1.59903547	1.74090002	-2.2476008	-1.1683858	0.00774759	20	leucine rich repeat containing 1 [Source:HGNC Symbol;Acc:14307]	LRRC1	55227
ENSG00000074590	1.89097325	3.69237841	1.82309022	2.28488388	-2.28813	-1.194169	0.01497646	21	NUAK family, SNF1-like kinase, 1 [Source:HGNC Symbol;Acc:14311]	NUAK1	9891
ENSG00000196169	2.01619468	3.8980915	2.51910844	4.3186191	-2.4116152	-1.2699998	0.02031442	22	kinesin family member 19 [Source:HGNC Symbol;Acc:26735]	KIF19	124602
ENSG00000186868	1.50535309	2.42754282	1.89308876	1.93476316	-2.2739353	-1.1851912	0.01629671	23	microtubule-associated protein tau [Source:HGNC Symbol;Acc:6893]	MAPT	4137
ENSG00000171385	0.94782094	2.44491634	1.13288639	2.41380006	-2.4595723	-1.2984075	0.02279423	24	potassium voltage-gated channel, Shal-related subfamily, member 3 [Source:HGNC Symbol;Acc:6239]	KCND3	3752
ENSG00000172260	2.11995347	3.77977085	2.01941424	2.78532382	-2.350436	-1.2329284	0.02165956	25	neuronal growth regulator 1 [Source:HGNC Symbol;Acc:17302]	NEGR1	257194
ENSG00000013573	7.54418517	10.9428981	7.52944142	6.64458827	-2.2638781	-1.1787963	0.01731416	26	DEAD/H (Asp-Glu-Ala-Asp/His) box helicase 11 [Source:HGNC Symbol;Acc:2736]	DDX11	1663

ENSG00000163814	1.35862778	2.42923709	1.53047439	1.52723823	-2.1877563	-1.129452	0.01681432	27	CUB domain containing protein 1 [Source:HGNC Symbol;Acc:24357]	CDCP1	64866
ENSG00000166025	1.55328832	2.63466585	1.26215137	1.70413646	-2.1293374	-1.0904046	0.01545182	28	angiomotin like 1 [Source:HGNC Symbol;Acc:17811]	AMOTL1	154810
ENSG00000166762	1.76933377	3.8724464	1.63488145	3.11307376	-2.1786692	-1.1234472	0.0225934	29	cation channel, sperm associated 2 [Source:HGNC Symbol;Acc:18810]	CATSPER2	101930343
ENSG00000109099	0.88982004	1.70245182	1.10254996	1.65828933	-2.3029877	-1.2035067	0.03616379	30	peripheral myelin protein 22 [Source:HGNC Symbol;Acc:9118]	PMP22	5376
ENSG00000175544	2.49910968	3.87304971	2.38840611	2.54774179	-2.1532048	-1.1064855	0.02614546	31	calcium binding protein 4 [Source:HGNC Symbol;Acc:1386]	CABP4	57010
ENSG00000185614	1.95753291	0.90201271	2.20654795	1.34796916	2.3686646	1.24407393	0.03876649	32	family with sequence similarity 212, member A [Source:HGNC Symbol;Acc:32480]	FAM212A	389119
ENSG00000171766	1.62356188	2.40922695	1.48288981	1.26689455	-2.1385582	-1.0966385	0.02701523	33	glycine amidinotransferase (L-arginine:glycine amidinotransferase) [Source:HGNC Symbol;Acc:4175]	GATM	2628
ENSG00000130147	1.02068351	1.90069444	1.92688898	1.8666661	-2.2619088	-1.1775407	0.0360495	34	SH3-domain binding protein 4 [Source:HGNC Symbol;Acc:10826]	SH3BP4	23677
ENSG00000134489	1.02606197	2.15289849	0.97216514	2.06124175	-2.1807141	-1.1248007	0.03220571	35	histamine receptor H4 [Source:HGNC Symbol;Acc:17383]	HRH4	59340
ENSG00000164330	9.74940067	13.816209	7.63081981	8.56692016	-2.1138858	-1.0798974	0.02856935	36	early B-cell factor 1 [Source:HGNC Symbol;Acc:3126]	EBF1	1879
ENSG00000179088	2.91737909	4.72071663	2.0349239	2.8033529	-1.9714256	-0.9792392	0.01409924	37	chromosome 12 open reading frame 42 [Source:HGNC Symbol;Acc:24729]	C12orf42	374470
ENSG00000130584	1.295216	1.97702879	1.68943622	1.64878546	-2.0897366	-1.0633211	0.02728549	38	zinc finger and BTB domain containing 46 [Source:HGNC Symbol;Acc:16094]	ZBTB46	140685
ENSG00000161800	1.94029166	3.21175681	1.76256994	1.92501521	-2.0481994	-1.0343561	0.02221074	39	Rac GTPase activating protein 1 [Source:HGNC Symbol;Acc:9804]	RACGAP1	29127
ENSG00000214595	3.67155932	5.53323964	3.61562527	5.25027012	-2.0799367	-1.0565396	0.02976978	40	echinoderm microtubule associated protein like 6 [Source:HGNC Symbol;Acc:35412]	EML6	400954

ENSG00000117016	1.06710873	1.79642809	1.32492864	1.27243631	-2.1496877	-1.1041271	0.03637052	41	regulating synaptic membrane exocytosis 3 [Source:HGNC Symbol;Acc:21292]	RIMS3	9783
ENSG00000128340	2.30654578	1.23759237	1.35917716	1.12555437	2.01423445	1.01023162	0.02373896	42	ras-related C3 botulinum toxin substrate 2 (rho family, small GTP binding protein Rac2) [Source:HGNC Symbol;Acc:9802]	RAC2	5880
ENSG00000124713	1.08626214	2.05044069	1.43025681	2.04582068	-2.2093608	-1.1436291	0.04669504	43	glycine N-methyltransferase [Source:HGNC Symbol;Acc:4415]	GNMT	27232
ENSG00000184305	3.91953069	5.39086314	3.32835384	2.95917638	-1.9817735	-0.9867921	0.02488012	44	coiled-coil serine-rich protein 1 [Source:HGNC Symbol;Acc:29349]	CCSER1	401145
ENSG00000167550	2.39019258	4.25494586	1.68751619	1.90600488	-1.9778783	-0.9839537	0.02580186	45	Ras homolog enriched in brain like 1 [Source:HGNC Symbol;Acc:21166]	RHEBL1	121268
ENSG00000164171	1.73200206	3.27946624	1.54819454	3.19256982	-2.0628206	-1.0446183	0.03807007	46	integrin, alpha 2 (CD49B, alpha 2 subunit of VLA-2 receptor) [Source:HGNC Symbol;Acc:6137]	ITGA2	3673
ENSG00000172264	6.40346772	8.14448088	8.27678244	4.78093785	-2.0249522	-1.0178879	0.03489307	47	MACRO domain containing 2 [Source:HGNC Symbol;Acc:16126]	MACROD2	140733
ENSG00000154380	1.12463664	2.21832719	1.13024525	2.30793302	-2.0758088	-1.0536735	0.03966166	49	enabled homolog (Drosophila) [Source:HGNC Symbol;Acc:18271]	ENAH	55740
ENSG00000132386	3.10140847	4.99261938	2.06415677	2.83720096	-1.8678178	-0.9013537	0.01543	52	serpin peptidase inhibitor, clade F (alpha-2 antiplasmin, pigment epithelium derived factor), member 1 [Source:HGNC Symbol;Acc:8824]	SERPINF1	5176
ENSG00000155755	1.57511129	2.25400635	1.42477157	1.32459733	-1.9288244	-0.9477218	0.02778615	53	transmembrane protein 237 [Source:HGNC Symbol;Acc:14432]	TMEM237	65062
ENSG00000140939	2.91502391	1.74512162	1.97442204	1.53186415	1.93613736	0.95318131	0.03845352	58	nucleolar protein 3 (apoptosis repressor with CARD domain) [Source:HGNC Symbol;Acc:7869]	NOL3	8996
ENSG00000108515	3.39275629	1.6677308	3.09450478	1.43044306	1.93900579	0.95531711	0.0476352	62	enolase 3 (beta, muscle) [Source:HGNC Symbol;Acc:3354]	ENO3	2027

ENSG00000092067	5.46229001	7.02287984	5.36544388	4.72878782	-1.9325172	-0.9504813	0.04272519	64	CCAAT/enhancer binding protein (C/EBP), epsilon [Source:HGNC Symbol;Acc:1836]	CEBPE	1053
ENSG00000136059	5.10734699	3.15253009	4.10821514	3.43508995	1.93107606	0.94940499	0.04285745	66	villin-like [Source:HGNC Symbol;Acc:30906]	VILL	50853
ENSG00000157168	11.4164105	6.66562615	5.86334167	4.61374423	1.80734428	0.85387135	0.02072764	67	neuregulin 1 [Source:HGNC Symbol;Acc:7997]	NRG1	3084
ENSG00000177483	2.51430083	3.60501928	1.84239669	1.31703296	-1.8209171	-0.8646652	0.03005557	69	RNA binding motif protein 44 [Source:HGNC Symbol;Acc:24756]	RBM44	375316
ENSG00000135482	4.53629106	2.66905153	2.57175323	1.85203853	1.82000046	0.86393881	0.03237361	72	zinc finger CCCH-type containing 10 [Source:HGNC Symbol;Acc:25893]	ZC3H10	84872
ENSG00000116678	8.92424456	12.7783657	8.6207599	7.39829998	-1.7973868	-0.8459009	0.03010138	74	leptin receptor [Source:HGNC Symbol;Acc:6554]	LEPR	54741
ENSG00000154655	4.22512533	5.16856481	4.07953558	2.61288716	-1.8104504	-0.8563487	0.03875913	77	l(3)mbt-like 4 (Drosophila) [Source:HGNC Symbol;Acc:26677]	L3MBTL4	91133
ENSG00000198692	10.7948039	22.4879114	33.536752	62.6063121	-1.7549322	-0.8114153	0.02218853	81	eukaryotic translation initiation factor 1A, Y-linked [Source:HGNC Symbol;Acc:3252]	EIF1AY	101060318
ENSG00000168152	1.67108038	2.778286	1.10693174	1.56781216	-1.8045329	-0.8516255	0.04719616	82	THAP domain containing 9 [Source:HGNC Symbol;Acc:23192]	THAP9	79725
ENSG00000104863	5.78437712	3.43982817	3.49720047	2.68778645	1.77169562	0.82513076	0.03504008	84	lin-7 homolog B (C. elegans) [Source:HGNC Symbol;Acc:17788]	LIN7B	64130
ENSG00000129696	6.11671492	3.50564007	3.37849072	2.61761086	1.73784096	0.79729606	0.02007242	86	TELO2 interacting protein 2 [Source:HGNC Symbol;Acc:26262]	TTI2	80185
ENSG00000138002	2.06677017	2.94783002	1.66803652	1.59489273	-1.795207	-0.8441502	0.04783685	87	intraflagellar transport 172 homolog (Chlamydomonas) [Source:HGNC Symbol;Acc:30391]	IFT172	26160
ENSG00000084731	3.87687317	2.67850622	1.72930182	2.28614321	1.75037648	0.80766526	0.03868552	92	kinesin family member 3C [Source:HGNC Symbol;Acc:6321]	KIF3C	3797
ENSG00000100084	3.44432114	4.96189945	2.4986937	2.26683606	-1.7431611	-0.8017059	0.03703838	93	histone cell cycle regulator	HIRA	7290

									[Source:HGNC Symbol;Acc:4916]		
ENSG00000050438	5.04372844	7.06234299	3.16392881	3.02238394	-1.6905795	-0.7575179	0.01962752	97	solute carrier family 4, sodium bicarbonate cotransporter, member 8 [Source:HGNC Symbol;Acc:11034]	SLC4A8	9498
ENSG00000117009	9.62475975	14.5201252	4.79682758	6.60026794	-1.7358588	-0.7956496	0.04269158	105	kynurenine 3-monooxygenase (kynurenine 3-hydroxylase) [Source:HGNC Symbol;Acc:6381]	KMO	8564
ENSG00000102796	13.6473805	7.74216235	7.18628987	2.95765831	1.6567275	0.72833633	0.01401354	112	dehydrogenase/reductase (SDR family) member 12 [Source:HGNC Symbol;Acc:25832]	DHRS12	79758
ENSG00000221838	4.99286422	7.48599241	2.83713859	2.04729365	-1.6587752	-0.7301183	0.01609952	113	adaptor-related protein complex 4, mu 1 subunit [Source:HGNC Symbol;Acc:574]	AP4M1	9179
ENSG00000090674	7.39624865	10.4641563	4.13289084	3.6415832	-1.6449634	-0.7180555	0.03075679	129	mucolipin 1 [Source:HGNC Symbol;Acc:13356]	MCOLN1	57192
ENSG00000163221	244.239562	134.808029	143.504761	61.9775744	1.64313992	0.71645534	0.03421707	134	S100 calcium binding protein A12 [Source:HGNC Symbol;Acc:10489]	S100A12	6283
ENSG00000188215	5.61378059	8.06048303	2.77759651	2.41051337	-1.5946918	-0.6732777	0.01528721	141	DCN1, defective in cullin neddylation 1, domain containing 3 [Source:HGNC Symbol;Acc:28734]	DCUN1D3	123879
ENSG00000113749	34.8561245	20.0130707	21.2401335	9.4795282	1.62033761	0.69629444	0.0404399	148	histamine receptor H2 [Source:HGNC Symbol;Acc:5183]	HRH2	3274
ENSG00000166483	6.20286854	9.42305543	4.11274352	5.87221193	-1.6063869	-0.6838194	0.04036032	155	WEE1 G2 checkpoint kinase [Source:HGNC Symbol;Acc:12761]	WEE1	7465
ENSG00000137441	55.0851935	88.1569811	28.992701	45.6484899	-1.5876268	-0.6668718	0.03185233	161	fibroblast growth factor binding protein 2 [Source:HGNC Symbol;Acc:29451]	FGFBP2	83888
ENSG00000139182	10.2060681	14.4505607	6.8032528	4.54631435	-1.5908567	-0.6698039	0.04019816	167	calsyntenin 3 [Source:HGNC Symbol;Acc:18371]	CLSTN3	9746
ENSG00000136319	10.69041	15.3744234	4.24834381	4.90419771	-1.5057374	-0.5904702	0.03887405	230	tetratricopeptide repeat domain 5 [Source:HGNC Symbol;Acc:19274]	TTC5	91875

ENSG00000164885	10.0540322	6.87353514	4.18057814	3.14388109	1.49594027	0.58105257	0.04104268	242	cyclin-dependent kinase 5 [Source:HGNC Symbol;Acc:1774]	CDK5	1020

Compared populations:

D2T RA vs non-D2T RA (sex-adjusted)

Fold change describes the size of the difference in gene expression (given in mean and standard deviation, sd) between the compared groups. The values are always in relation to the group used as a base level group (reference). P-value describes the reliability of the change in expression value between the compared groups. Results were considered significant with a fold change of 1.2 and a p-value cutoff of 0.05.

Supplementary Material 26 - List of differentially expressed (DE) genes (sex-adjusted)

ID	mean_HighInflammation_HighPain	mean_HighInflammation_LowPain	sd_HighInflammation_HighPain	sd_HighInflammation_LowPain	FC	logFC	P.Value	avgRank	Description	geneName	Entrez ID
ENSG00000174705	1.44886547	2.91181052	2.62688049	1.61068453	-5.2171559	-2.3832636	0.00201593	1	SH3 and PX domains 2B [Source:HGNC Symbol;Acc:29242]	SH3PXD2B	285590
ENSG00000166582	4.09168028	1.3645782	2.30845557	1.4798942	4.20714281	2.07284079	0.00068473	2	centromere protein V [Source:HGNC Symbol;Acc:29920]	CENPV	201161
ENSG00000134321	6.17402557	71.2942671	7.08654066	126.847929	-5.6602677	-2.5008703	0.00530377	3	radical S-adenosyl methionine domain containing 2 [Source:HGNC Symbol;Acc:30908]	RSAD2	91543
ENSG00000144792	3.72905959	0.85596755	3.2209365	0.95104728	4.89889259	2.29245566	0.00356711	4	zinc finger protein 660 [Source:HGNC Symbol;Acc:26720]	ZNF660	285349
ENSG00000112874	3.15964631	1.17921263	1.8233626	1.23886886	4.22661513	2.07950275	0.00288077	5	nudix (nucleoside diphosphate linked moiety X)-type motif 12 [Source:HGNC Symbol;Acc:18826]	NUDT12	83594
ENSG00000160360	4.71558741	1.09158995	2.86147193	0.88743788	4.13078242	2.04641507	0.00073776	6	G-protein signaling modulator 1 [Source:HGNC Symbol;Acc:17858]	GPSM1	26086
ENSG00000182636	2.42140245	0.33839728	1.87701143	0.5853332	4.90195416	2.29335699	0.00728274	7	necdin, melanoma antigen (MAGE) family member [Source:HGNC Symbol;Acc:7675]	NDN	4692
ENSG00000188076	3.61989628	0.46616821	4.42456234	0.86568759	6.14369194	2.61910588	0.00905926	8	secretoglobin, family 1C, member 1 [Source:HGNC Symbol;Acc:18394]	SCGB1C1	147199
ENSG00000102349	6.64098797	3.19482086	3.4676653	2.99064619	3.79245025	1.92313026	0.00334845	9	Kruppel-like factor 8 [Source:HGNC Symbol;Acc:6351]	KLF8	11279
ENSG00000158106	4.01403829	1.32344618	2.37059116	0.96779694	3.44829391	1.78588275	0.00254636	10	rhophilin, Rho GTPase binding protein 1 [Source:HGNC Symbol;Acc:19973]	RHPN1	114822
ENSG00000188033	1.7678329	0.54169444	1.02350586	0.82241335	3.85160304	1.94545902	0.01206362	11	zinc finger protein 490 [Source:HGNC Symbol;Acc:23705]	ZNF490	57474
ENSG00000053524	3.60094753	1.35819058	2.33651721	1.29897703	3.5048036	1.8093336	0.00746697	12	MCF.2 cell line derived transforming sequence-like 2 [Source:HGNC Symbol;Acc:30319]	MCF2L2	23101
ENSG00000133169	2.42224394	1.23124006	1.86547748	1.82428882	3.66320229	1.87310537	0.01128636	13	brain expressed, X-linked 1 [Source:HGNC Symbol;Acc:1036]	BEX1	55859
ENSG00000146530	1.80590832	0.32618932	1.95853831	0.81171007	3.97645539	1.99148299	0.01277411	14	von Willebrand factor D and EGF domains [Source:HGNC Symbol;Acc:21897]	VWDE	221806

ENSG00000197208	3.78462557	3.42806285	2.50874686	6.07399166	4.6105544	2.20494024	0.01667269	15	solute carrier family 22 (organic cation/zwitterion transporter), member 4 [Source:HGNC Symbol;Acc:10968]	SLC22A4	6583
ENSG00000094804	0.51045817	1.92841313	0.67052226	1.22502879	-4.1607279	-2.0568359	0.01655942	16	cell division cycle 6 [Source:HGNC Symbol;Acc:1744]	CDC6	990
ENSG00000088827	2.80127358	37.2816676	3.27430772	65.4937804	-4.7807251	-2.2572295	0.01927674	17	sialic acid binding Ig-like lectin 1, sialoadhesin [Source:HGNC Symbol;Acc:11127]	SIGLEC1	6614
ENSG00000180071	10.9940847	5.20788456	4.32269894	3.2096668	3.21141771	1.68321033	0.00273333	18	ankyrin repeat domain 18A [Source:HGNC Symbol;Acc:23643]	ANKRD18A	253650
ENSG00000145632	0.51653841	2.19787316	0.55291835	1.60460808	-3.9064874	-1.965872	0.01794806	19	polo-like kinase 2 [Source:HGNC Symbol;Acc:19699]	PLK2	10769
ENSG00000105173	0.90507434	2.78686035	0.79802229	1.67398546	-3.414176	-1.7715374	0.0114888	20	cyclin E1 [Source:HGNC Symbol;Acc:1589]	CCNE1	898
ENSG00000097096	5.08975957	1.85525379	2.31020319	1.4423742	3.51338424	1.81286137	0.01264108	21	synapse defective 1, Rho GTPase, homolog 2 (C. elegans) [Source:HGNC Symbol;Acc:25841]	SYDE2	84144
ENSG00000173258	5.00674411	1.82619072	2.58054077	1.50212531	3.2237026	1.68871866	0.00769708	22	zinc finger protein 483 [Source:HGNC Symbol;Acc:23384]	ZNF483	158399
ENSG00000132330	0.67084432	2.21067533	1.09725552	1.7613322	-3.4512624	-1.7871241	0.01445881	23	selenocysteine lyase [Source:HGNC Symbol;Acc:18161]	SCLY	51540
ENSG00000185745	11.0990206	45.9630783	18.2168941	66.2579027	-4.2228598	-2.0782204	0.02550272	24	interferon-induced protein with tetratricopeptide repeats 1 [Source:HGNC Symbol;Acc:5407]	IFIT1	3434
ENSG00000017427	2.22656045	1.17618995	1.58730205	1.77685999	3.65695772	1.87064395	0.02293628	25	insulin-like growth factor 1 (somatomedin C) [Source:HGNC Symbol;Acc:5464]	IGF1	3479
ENSG00000116151	1.13853531	2.40355335	1.45531471	1.15420084	-3.3771737	-1.7558164	0.01760513	26	MORN repeat containing 1 [Source:HGNC Symbol;Acc:25852]	MORN1	79906
ENSG00000179253	0.44594914	1.77042867	0.47497703	1.21566657	-3.5442773	-1.8254915	0.02272822	27	Uncharacterized protein [Source:UniProtKB/TrEMBL;Acc:Q5VZ43]	RP11-429E11.3	
ENSG00000247626	3.16150932	2.06441821	1.88897473	2.19794195	3.32403093	1.73293381	0.01669613	28	methionyl-tRNA synthetase 2, mitochondrial [Source:HGNC Symbol;Acc:25133]	MARS2	92935
ENSG00000141696	5.20972665	1.69420976	3.45392971	1.43757105	3.00280012	1.58630844	0.00868806	29	leprecan-like 4 [Source:HGNC Symbol;Acc:16946]	LEPREL4	10609
ENSG00000243452	1.84159405	0.44656933	2.48424737	0.50974029	3.53515704	1.8217743	0.02334677	30	neuroblastoma breakpoint family, member 15	NBPF15	284565

									[Source:HGNC Symbol;Acc:28791]		
ENSG00000188997	6.6878143	2.99228985	3.61845857	1.72284676	2.88884379	1.5304922	0.00606313	31	potassium channel tetramerization domain containing 21 [Source:HGNC Symbol;Acc:27452]	KCTD21	283219
ENSG00000187801	1.98383878	0.52776447	1.55894301	0.70276053	3.72697157	1.89800381	0.0272957	32	ZFP69 zinc finger protein B [Source:HGNC Symbol;Acc:28053]	ZFP69B	65243
ENSG00000204882	1.88851502	4.23225121	2.79797608	3.32681729	-3.6373418	-1.8628845	0.02626119	33	G protein-coupled receptor 20 [Source:HGNC Symbol;Acc:4475]	GPR20	2843
ENSG00000228716	11.4458109	5.38274909	7.66445848	4.08207478	3.58617742	1.84244687	0.025954	34	dihydrofolate reductase [Source:HGNC Symbol;Acc:2861]	DHFR	1719
ENSG00000197502	4.54744939	2.62155949	2.11304117	2.50121801	3.00636751	1.58802138	0.01261317	35	Uncharacterized protein [Source:UniProtKB/TrEMBL;Acc:H7BY28]	AL627171.1	
ENSG00000101230	2.73917308	0.59450478	2.95089788	0.6963887	3.47480501	1.79693202	0.02683008	36	isthmin 1, angiogenesis inhibitor [Source:HGNC Symbol;Acc:16213]	ISM1	140862
ENSG00000117322	6.69716257	4.99298917	3.86203362	5.28404027	3.39051753	1.7615055	0.02561944	37	complement component (3d/Epstein Barr virus) receptor 2 [Source:HGNC Symbol;Acc:2336]	CR2	1380
ENSG00000184305	3.59378279	2.94255156	2.34909462	4.39934732	3.09946206	1.63201785	0.01997262	38	coiled-coil serine-rich protein 1 [Source:HGNC Symbol;Acc:29349]	CCSER1	401145
ENSG00000135643	2.00637078	0.65931559	1.37162134	0.81214428	3.2526669	1.70162308	0.02485558	39	potassium large conductance calcium-activated channel, subfamily M, beta member 4 [Source:HGNC Symbol;Acc:6289]	KCNMB4	27345
ENSG00000174469	3.91253721	1.12485718	3.50306466	1.50035845	3.90857254	1.96664181	0.03370653	40	contactin associated protein-like 2 [Source:HGNC Symbol;Acc:13830]	CNTNAP2	26047
ENSG00000153157	0.44597934	2.12890111	0.95650454	2.57529347	-4.1732807	-2.061182	0.03500703	41	synaptonemal complex protein 2-like [Source:HGNC Symbol;Acc:21537]	SYCP2L	221711
ENSG00000159761	1.30301665	3.22920144	1.19651512	1.86811107	-2.7180067	-1.442549	0.00523677	42	chromosome 16 open reading frame 86 [Source:HGNC Symbol;Acc:33755]	C16orf86	388284
ENSG00000130827	5.11054368	2.45276446	4.48925951	3.06415848	2.98790193	1.5791328	0.0177406	43	plexin A3 [Source:HGNC Symbol;Acc:9101]	PLXNA3	55558
ENSG00000147138	2.49126288	0.78208291	1.72607276	0.72231192	3.36053925	1.74869275	0.02806026	44	G protein-coupled receptor 174 [Source:HGNC Symbol;Acc:30245]	GPR174	84636
ENSG00000198934	5.46084479	3.1951386	2.60576737	2.94925789	2.78954245	1.4800285	0.01112662	45	melanoma antigen family E, 1 [Source:HGNC Symbol;Acc:24934]	MAGEE1	57692

ENSG00000119917	52.1562059	212.48614	45.4800261	245.1382	-3.036405	-1.6023642	0.02418354	46	interferon-induced protein with tetratricopeptide repeats 3 [Source:HGNC Symbol;Acc:5411]	IFIT3	3437
ENSG00000180423	4.8646312	2.47752689	3.5147388	2.16622007	2.77713511	1.47359737	0.01238614	47	harbinger transposase derived 1 [Source:HGNC Symbol;Acc:26522]	HARBI1	283254
ENSG00000145386	4.40782871	1.82094212	3.71784327	1.70937377	3.13155386	1.64687869	0.02796345	48	cyclin A2 [Source:HGNC Symbol;Acc:1578]	CCNA2	890
ENSG00000177628	1.03738148	2.51554158	1.2411281	2.26896772	-2.9330036	-1.5523789	0.02458567	49	glucosidase, beta, acid [Source:HGNC Symbol;Acc:4177]	GBA	2629
ENSG00000119922	69.3556126	248.277052	57.4924138	278.811566	-2.8237455	-1.4976101	0.02034394	50	interferon-induced protein with tetratricopeptide repeats 2 [Source:HGNC Symbol;Acc:5409]	IFIT2	3433
ENSG00000204311	0.28930006	1.44645315	0.47974833	1.73294349	-3.1548545	-1.6575735	0.03128194	51	deafness, autosomal recessive 59 [Source:HGNC Symbol;Acc:29502]	DFNB59	494513
ENSG00000126368	3.39297537	1.49189521	3.10823105	2.0318696	3.36392283	1.75014461	0.03524677	52	nuclear receptor subfamily 1, group D, member 1 [Source:HGNC Symbol;Acc:7962]	NR1D1	9572
ENSG00000119514	4.71106914	2.62758695	2.19483456	2.64929348	3.01698717	1.59310856	0.0287333	53	UDP-N-acetyl-alpha-D-galactosamine:polypeptide N-acetylgalactosaminyltransferase 12 (GalNAc-T12) [Source:HGNC Symbol;Acc:19877]	GALNT12	79695
ENSG00000196126	554.130249	361.400213	320.492489	379.130041	2.69785846	1.43181466	0.01511939	54	major histocompatibility complex, class II, DR beta 1 [Source:HGNC Symbol;Acc:4948]	HLA-DRB1	100996809
ENSG00000105647	4.44980096	2.65464526	2.18827338	2.40958032	2.60942742	1.38373328	0.00995472	55	phosphoinositide-3-kinase, regulatory subunit 2 (beta) [Source:HGNC Symbol;Acc:8980]	PIK3R2	5296
ENSG00000103942	5.56718053	2.94524633	3.04454439	2.22797005	2.65727818	1.40994926	0.01479674	56	homer homolog 2 (Drosophila) [Source:HGNC Symbol;Acc:17513]	HOMER2	9455
ENSG00000168005	1.34340318	2.07634563	1.88618017	0.98144977	-2.8337834	-1.5027295	0.02497469	57	chromosome 11 open reading frame 84 [Source:HGNC Symbol;Acc:25115]	C11orf84	144097
ENSG00000151023	15.8658613	7.7426913	10.5190258	8.81172522	3.57681712	1.83867636	0.04245182	58	enkurin, TRPC channel interacting protein [Source:HGNC Symbol;Acc:28388]	ENKUR	219670
ENSG00000181754	8.36328957	4.68694973	3.80554146	3.36621419	2.58462451	1.3699547	0.0102189	59	adhesion molecule with Ig-like domain 1 [Source:HGNC Symbol;Acc:20824]	AMIGO1	57463

ENSG00000100024	1.8396304	0.65406491	1.50591163	1.23016211	3.61427532	1.85370641	0.04482903	60	ureidopropionase, beta [Source:HGNC Symbol;Acc:16297]	UPB1	51733
ENSG00000197561	18.5931805	4.0162318	45.792747	3.76252984	3.0437478	1.60584883	0.03767771	61	elastase, neutrophil expressed [Source:HGNC Symbol;Acc:3309]	ELANE	1991
ENSG00000154309	2.22681182	1.3304049	1.56758887	1.79291677	2.8064811	1.48876235	0.03000818	62	dispatched homolog 1 (Drosophila) [Source:HGNC Symbol;Acc:19711]	DISP1	84976
ENSG00000135747	0.58085377	1.61388917	0.78628484	1.03166726	-3.0250769	-1.5969718	0.03865507	63	zinc finger protein 670 [Source:HGNC Symbol;Acc:28167]	ZNF670	93474
ENSG00000115526	1.58286513	0.63510431	1.25459524	0.89470264	3.08669218	1.62606162	0.03986959	64	carbohydrate sulfotransferase 10 [Source:HGNC Symbol;Acc:19650]	CHST10	9486
ENSG00000101417	3.287604	1.74049546	1.91908492	1.84253616	2.7986558	1.48473406	0.03150846	65	peroxisomal membrane protein 4, 24kDa [Source:HGNC Symbol;Acc:15920]	PXMP4	11264
ENSG00000240403	1.27524705	3.03920541	2.06761176	2.62946217	-3.2301913	-1.6916196	0.04315148	66	killer cell immunoglobulin-like receptor, three domains, long cytoplasmic tail, 2 [Source:HGNC Symbol;Acc:6339]	KIR3DL2	727787
ENSG00000187583	2.73471844	0.87660376	2.05022335	0.99817171	3.20438287	1.68004653	0.04314455	67	pleckstrin homology domain containing, family N member 1 [Source:HGNC Symbol;Acc:25284]	PLEKHN1	84069
ENSG00000137720	3.42448634	2.01271366	2.21453687	2.09686558	2.70194697	1.43399936	0.02805716	68	chromosome 11 open reading frame 1 [Source:HGNC Symbol;Acc:1163]	C11orf1	64776
ENSG00000128641	2.92770302	1.40062676	2.3156161	1.7294113	2.72936707	1.44856644	0.0296052	69	myosin IB [Source:HGNC Symbol;Acc:7596]	MYO1B	4430
ENSG00000062582	1.18303329	2.04218323	1.60539725	1.08312149	-2.8987849	-1.5354483	0.03787035	70	mitochondrial ribosomal protein S24 [Source:HGNC Symbol;Acc:14510]	MRPS24	64951
ENSG00000127585	11.3378047	5.04747021	6.2600735	3.4522661	2.45467244	1.29553052	0.00858948	71	F-box and leucine-rich repeat protein 16 [Source:HGNC Symbol;Acc:14150]	FBXL16	146330
ENSG00000157077	4.91378418	2.39039738	3.28642856	2.38565467	2.73582397	1.45197541	0.03117011	72	zinc finger, FYVE domain containing 9 [Source:HGNC Symbol;Acc:6775]	ZFYVE9	9372
ENSG00000182853	1.41370121	3.62340477	1.77398903	3.21531652	-3.1542169	-1.6572819	0.0439017	73	vitelline membrane outer layer 1 homolog (chicken) [Source:HGNC Symbol;Acc:30387]	VMO1	284013
ENSG00000106484	1.89633015	0.71268493	1.36487362	1.00290543	2.99900912	1.58448591	0.04293845	74	mesoderm specific transcript [Source:HGNC Symbol;Acc:7028]	MEST	4232
ENSG00000176454	5.56669749	2.65489831	3.44853544	1.54723465	2.49163755	1.31709422	0.01563465	75	lysophosphatidylcholine acyltransferase 4	LPCAT4	254531

									[Source:HGNC Symbol;Acc:30059]		
ENSG00000074590	1.33914087	3.13188812	1.3585166	2.33247728	-2.8812454	-1.5266925	0.04133298	76	NUAK family, SNF1-like kinase, 1 [Source:HGNC Symbol;Acc:14311]	NUAK1	9891
ENSG00000143382	3.9704929	8.12799512	2.97744723	4.50219405	-2.4907644	-1.3165886	0.0165678	77	ADAMTS-like 4 [Source:HGNC Symbol;Acc:19706]	ADAMTSL4	54507
ENSG00000171657	1.54624883	2.6800873	2.18183504	1.9954562	-3.0255175	-1.5971819	0.04661571	78	G protein-coupled receptor 82 [Source:HGNC Symbol;Acc:4533]	GPR82	27197
ENSG00000157429	3.44289375	1.6809478	2.25838093	1.72112921	2.77074631	1.47027462	0.03821108	80	zinc finger protein 19 [Source:HGNC Symbol;Acc:12981]	ZNF19	7567
ENSG00000182768	2.56760868	5.62649915	2.10286481	4.02615612	-2.6278949	-1.3939076	0.03173659	81	neugrin, neurite outgrowth associated [Source:HGNC Symbol;Acc:18077]	NGRN	51335
ENSG00000127580	5.23111569	2.26532644	4.64866675	2.20290305	2.69199035	1.42867324	0.03622278	82	WD repeat domain 24 [Source:HGNC Symbol;Acc:20852]	WDR24	84219
ENSG00000156510	5.30314574	4.26916151	3.94216563	7.06602309	2.81660068	1.49395504	0.04253094	83	hexokinase domain containing 1 [Source:HGNC Symbol;Acc:23302]	HKDC1	80201
ENSG00000179941	5.43010421	2.9296342	2.54496726	3.82293599	2.47261557	1.30603795	0.02177318	84	Bardet-Biedl syndrome 10 [Source:HGNC Symbol;Acc:26291]	BBS10	79738
ENSG00000149489	2.71310035	1.02743344	2.99553225	1.03185569	2.73590347	1.45201733	0.0416565	85	retinal outer segment membrane protein 1 [Source:HGNC Symbol;Acc:10254]	ROM1	6094
ENSG00000155265	3.54315893	1.90095474	3.1821932	3.10833092	2.87828193	1.52520791	0.04870335	87	golgin A7 family, member B [Source:HGNC Symbol;Acc:31668]	GOLGA7B	401647
ENSG00000188290	2.61307067	6.47679882	1.9113034	5.01623416	-2.6359082	-1.3983001	0.03950306	88	hes family bHLH transcription factor 4 [Source:HGNC Symbol;Acc:24149]	HES4	57801
ENSG00000112796	2.13672262	3.58884123	2.22895523	2.46645692	-2.7082186	-1.4373442	0.04410573	91	ectonucleotide pyrophosphatase/phosphodiesterase 5 (putative) [Source:HGNC Symbol;Acc:13717]	ENPP5	59084
ENSG00000100372	11.2641792	6.07797228	6.64349757	4.12807554	2.30572281	1.20521909	0.01127065	92	solute carrier family 25 (mitochondrial carrier; peroxisomal membrane protein, 34kDa), member 17 [Source:HGNC Symbol;Acc:10987]	SLC25A17	10478
ENSG00000171421	9.05294237	5.68427206	4.07014867	7.22171066	2.50856978	1.32686507	0.03395739	93	mitochondrial ribosomal protein L36 [Source:HGNC Symbol;Acc:14490]	MRPL36	64979

ENSG00000242013	6.76668206	4.16449176	2.12835148	3.43590347	2.41441703	1.27167488	0.02732164	95	ubiquitin specific peptidase 27, X-linked [Source:HGNC Symbol;Acc:13486]	USP27X	389856
ENSG00000087884	3.92017026	5.93651572	3.9863642	3.75410989	-2.4177068	-1.2736393	0.02889018	97	adipogenesis associated, Mth938 domain containing [Source:HGNC Symbol;Acc:30205]	AAMDC	28971
ENSG00000120217	5.29093622	10.5224121	5.06934564	6.09240239	-2.3699141	-1.2448348	0.02484745	98	CD274 molecule [Source:HGNC Symbol;Acc:17635]	CD274	29126
ENSG00000120896	5.31666035	2.3367521	3.89879069	1.19551649	2.33996305	1.22648575	0.02455058	99	sorbin and SH3 domain containing 3 [Source:HGNC Symbol;Acc:30907]	SORBS3	10174
ENSG00000146243	2.4407345	6.14744854	1.55088768	3.94231365	-2.3880757	-1.2558486	0.02837058	101	interleukin-1 receptor-associated kinase 1 binding protein 1 [Source:HGNC Symbol;Acc:17368]	IRAK1BP1	134728
ENSG00000169612	5.36492504	3.41119689	3.09239706	3.27920932	2.53650532	1.34284219	0.04337765	107	family with sequence similarity 103, member A1 [Source:HGNC Symbol;Acc:31022]	FAM103A1	83640
ENSG00000196372	3.51898368	2.6001444	1.53189075	2.37126896	2.38032932	1.25116119	0.03063351	110	ankyrin repeat and SOCS box containing 13 [Source:HGNC Symbol;Acc:19765]	ASB13	79754
ENSG00000178338	7.76521582	4.81482218	4.47974234	3.51932177	2.4006709	1.26343764	0.03416238	111	zinc finger protein 354B [Source:HGNC Symbol;Acc:17197]	ZNF354B	117608
ENSG00000006432	1.83972903	0.99464191	0.93709612	1.26383163	2.51610223	1.33119054	0.04834198	116	mitogen-activated protein kinase kinase kinase 9 [Source:HGNC Symbol;Acc:6861]	MAP3K9	4293
ENSG00000167995	14.9282934	26.4483635	11.6019575	12.1807061	-2.3293652	-1.2199369	0.03245595	117	bestrophin 1 [Source:HGNC Symbol;Acc:12703]	BEST1	7439
ENSG00000100084	3.92239187	1.89394052	3.29410905	1.40160755	2.36431689	1.24142342	0.03593398	118	histone cell cycle regulator [Source:HGNC Symbol;Acc:4916]	HIRA	7290
ENSG00000135114	4.1004822	17.8894388	2.04439024	26.065882	-2.5064047	-1.3256194	0.04773371	119	2'-5'-oligoadenylate synthetase-like [Source:HGNC Symbol;Acc:8090]	OASL	8638
ENSG00000138002	2.704287	1.81573105	1.63967386	1.7939083	2.44750987	1.29131468	0.04388968	122	intraflagellar transport 172 homolog (Chlamydomonas) [Source:HGNC Symbol;Acc:30391]	IFT172	26160
ENSG00000240972	3.47888759	1.54624457	1.97439472	0.98907228	2.30643157	1.20566249	0.03447488	123	macrophage migration inhibitory factor (glycosylation-inhibiting factor) [Source:HGNC Symbol;Acc:7097]	MIF	4282

ENSG00000182183	14.7117842	9.31597706	8.03918426	6.22262299	2.29948318	1.20130964	0.03419873	124	family with sequence similarity 159, member A [Source:HGNC Symbol;Acc:28757]	FAM159A	348378
ENSG00000154451	120.002553	282.356509	69.308177	185.477256	-2.144961	-1.1009514	0.01144826	125	guanylate binding protein 5 [Source:HGNC Symbol;Acc:19895]	GBP5	115362
ENSG00000172824	3.61955532	1.96910854	2.8697961	1.63439893	2.32175487	1.21521566	0.03955587	130	carboxylesterase 4A [Source:HGNC Symbol;Acc:26741]	CES4A	283848
ENSG00000230510	1.50027492	2.94328508	1.21951698	1.71212642	-2.2125885	-1.1457352	0.027785	131	PPP5 tetratricopeptide repeat domain containing 1 [Source:HGNC Symbol;Acc:44209]	PPP5D1	100506012
ENSG00000128039	7.41868603	4.1639821	4.08761692	2.52344716	2.16845253	1.11666586	0.02023899	132	steroid 5 alpha-reductase 3 [Source:HGNC Symbol;Acc:25812]	SRD5A3	79644
ENSG00000141497	2.35401139	5.18581004	1.09271728	1.82146627	-2.1684037	-1.1166334	0.02173753	136	zinc finger, MYND-type containing 15 [Source:HGNC Symbol;Acc:20997]	ZMYND15	84225
ENSG00000138646	24.1659709	100.250743	18.0823876	172.528796	-2.2469592	-1.1679739	0.03518662	138	HECT and RLD domain containing E3 ubiquitin protein ligase 5 [Source:HGNC Symbol;Acc:24368]	HERC5	51191
ENSG00000159788	7.91604728	11.7270351	5.80035628	3.58627804	-2.1621238	-1.1124491	0.02234469	141	regulator of G-protein signaling 12 [Source:HGNC Symbol;Acc:9994]	RGS12	6002
ENSG00000133131	10.3077866	5.23613013	4.78191055	2.87812262	2.09686287	1.06823251	0.00888239	143	MORC family CW-type zinc finger 4 [Source:HGNC Symbol;Acc:23485]	MORC4	79710
ENSG00000110921	9.79260275	4.65746997	3.64192117	1.68997841	2.10209609	1.07182862	0.01048944	145	mevalonate kinase [Source:HGNC Symbol;Acc:7530]	MVK	4598
ENSG00000172878	3.60977494	2.44174497	1.85198991	2.83276773	2.21166009	1.14512968	0.0324283	150	methionyl aminopeptidase type 1D (mitochondrial) [Source:HGNC Symbol;Acc:32583]	METAP1D	254042
ENSG00000105426	5.10404503	2.76527193	2.76697604	1.93338165	2.22907562	1.15644556	0.03672971	154	protein tyrosine phosphatase, receptor type, S [Source:HGNC Symbol;Acc:9681]	PTPRS	5802
ENSG00000148331	7.32325794	4.94648112	2.51293284	2.75270315	2.25857621	1.17541359	0.0404081	155	ankyrin repeat and SOCS box containing 6 [Source:HGNC Symbol;Acc:17181]	ASB6	140459
ENSG00000125650	3.63244684	2.41228834	2.05346142	1.97403753	2.22662895	1.15486117	0.04190674	159	persephin [Source:HGNC Symbol;Acc:9579]	PSPN	5623
ENSG00000167685	10.2817702	5.9421557	1.53522758	2.50133063	2.01653705	1.01187991	0.00236907	162	zinc finger protein 444 [Source:HGNC Symbol;Acc:16052]	ZNF444	55311
ENSG00000177706	17.021089	8.57330557	6.90350357	3.73804847	2.02524707	1.01809792	0.00446481	163	family with sequence similarity 20, member C [Source:HGNC Symbol;Acc:22140]	FAM20C	56975

ENSG00000133106	40.1348883	110.837661	28.9938429	111.675382	-2.1191823	-1.0835077	0.03928688	172	epithelial stromal interaction 1 (breast) [Source:HGNC Symbol;Acc:16465]	EPSTI1	94240
ENSG00000130589	33.7650928	76.3083343	17.5224324	54.2630918	-1.9951465	-0.9964947	0.01224269	176	helicase with zinc finger 2, transcriptional coactivator [Source:HGNC Symbol;Acc:30021]	HELZ2	85441
ENSG00000168939	3.15603107	4.17623141	2.66147401	1.53742624	-2.161422	-1.1119808	0.0495295	181	sprouty homolog 3 (Drosophila) [Source:HGNC Symbol;Acc:11271]	SPRY3	10251
ENSG00000180509	10.4262085	15.8658398	9.55578917	7.30144519	-2.0637666	-1.0452798	0.03680857	188	potassium voltage-gated channel, Isk-related family, member 1 [Source:HGNC Symbol;Acc:6240]	KCNE1	3753
ENSG00000182010	44.8107761	23.7702005	15.9242237	13.0753473	1.9763849	0.98286394	0.02040497	194	rhotekin 2 [Source:HGNC Symbol;Acc:19364]	RTKN2	219790
ENSG00000105245	3.37072604	2.44249933	1.67981311	1.84555855	2.08843683	1.06242351	0.04255565	195	numb homolog (Drosophila)-like [Source:HGNC Symbol;Acc:8061]	NUMBL	9253
ENSG00000155592	4.35419373	2.58531349	2.26139914	2.29637105	2.12597309	1.08812334	0.04996747	196	zinc finger with KRAB and SCAN domains 2 [Source:HGNC Symbol;Acc:25677]	ZKSCAN2	342357
ENSG00000196220	4.51293016	2.64924047	1.86995374	1.90826669	2.11113743	1.0780205	0.04732125	197	SLIT-ROBO Rho GTPase activating protein 3 [Source:HGNC Symbol;Acc:19744]	SRGAP3	9901
ENSG00000158321	37.8934189	31.1427783	14.6602657	36.5500396	2.00618925	1.0044577	0.03552901	209	autism susceptibility candidate 2 [Source:HGNC Symbol;Acc:14262]	AUTS2	26053
ENSG00000166228	3.93199751	6.9704512	3.15920599	4.30889091	-2.0079981	-1.0057579	0.03787364	212	pterin-4 alpha-carbinolamine dehydratase/dimerization cofactor of hepatocyte nuclear factor 1 alpha [Source:HGNC Symbol;Acc:8646]	PCBD1	5092
ENSG00000103494	4.34549937	2.55112013	2.01074421	1.71075207	2.04194057	1.02994088	0.04537199	215	RPGRIP1-like [Source:HGNC Symbol;Acc:29168]	RPGRIP1L	23322
ENSG00000117228	120.677164	290.189406	51.8642969	224.001937	-1.968191	-0.9768702	0.03223106	218	guanylate binding protein 1, interferon-inducible [Source:HGNC Symbol;Acc:4182]	GBP1	2633
ENSG00000204475	3.69097792	7.07816771	2.54940062	3.94061768	-2.0376037	-1.0268735	0.04757996	221	natural cytotoxicity triggering receptor 3 [Source:HGNC Symbol;Acc:19077]	NCR3	259197
ENSG00000197714	2.91745213	4.76925287	1.95572485	1.82744979	-2.0145108	-1.0104295	0.04363374	224	zinc finger protein 460 [Source:HGNC Symbol;Acc:21628]	ZNF460	10794
ENSG00000186318	12.9823395	7.35830684	6.79110792	3.95300788	2.01160375	1.00834615	0.04564958	227	beta-site APP-cleaving enzyme 1 [Source:HGNC Symbol;Acc:933]	BACE1	23621

ENSG00000180758	5.56213816	3.7109253	2.32256852	2.01818512	1.9549044	0.96709806	0.03746438	230	G protein-coupled receptor 157 [Source:HGNC Symbol;Acc:23687]	GPR157	80045
ENSG00000155906	3.02809792	1.51234637	1.7230058	1.18701917	1.95733639	0.96889172	0.03822415	231	required for meiotic nuclear division 1 homolog (S. cerevisiae) [Source:HGNC Symbol;Acc:21176]	RMND1	55005
ENSG00000184903	5.0305237	2.54026617	2.24668167	1.27341082	2.00265516	1.00191402	0.04681991	233	IMP2 inner mitochondrial membrane peptidase-like (S. cerevisiae) [Source:HGNC Symbol;Acc:14598]	IMMP2L	83943
ENSG00000116991	15.9281011	28.5127655	11.9163581	14.6166413	-1.9551599	-0.9672866	0.04251123	243	signal-induced proliferation-associated 1 like 2 [Source:HGNC Symbol;Acc:23800]	SIPA1L2	57568
ENSG00000166261	8.21253688	4.56543144	3.46965742	2.08677138	1.87862825	0.90967961	0.02674449	246	zinc finger protein 202 [Source:HGNC Symbol;Acc:12994]	ZNF202	7753
ENSG00000198692	14.8391157	19.1056621	46.8411518	35.3768642	1.86182	0.8967136	0.02225394	247	eukaryotic translation initiation factor 1A, Y-linked [Source:HGNC Symbol;Acc:3252]	EIF1AY	101060318
ENSG00000243660	2.651677	5.71875706	1.89316707	4.75401054	-1.8970466	-0.9237551	0.03959583	250	zinc finger protein 487 [Source:HGNC Symbol;Acc:23488]	ZNF487	
ENSG00000204564	9.0883092	5.63356381	2.9859328	4.9306818	1.86887694	0.90217158	0.03257229	256	chromosome 6 open reading frame 136 [Source:HGNC Symbol;Acc:21301]	C6orf136	221545
ENSG00000139546	11.2797271	7.08406561	2.81470261	3.76203091	1.81514442	0.86008434	0.01495354	257	TAR (HIV-1) RNA binding protein 2 [Source:HGNC Symbol;Acc:11569]	TARBP2	6895
ENSG00000136997	8.85482215	5.2481418	5.37538165	3.13844763	1.89331471	0.92091424	0.04626243	269	v-myc avian myelocytomatosis viral oncogene homolog [Source:HGNC Symbol;Acc:7553]	MYC	4609
ENSG00000204186	11.6771362	7.46147742	4.86835582	4.51432126	1.8513741	0.88859645	0.04228899	283	zinc finger, DBF-type containing 2 [Source:HGNC Symbol;Acc:29313]	ZDBF2	57683
ENSG00000141012	11.1094734	20.0727856	3.32523283	6.11608717	-1.759465	-0.8151368	0.00553155	284	galactosamine (N-acetyl)-6-sulfate sulfatase [Source:HGNC Symbol;Acc:4122]	GALNS	2588
ENSG00000130489	33.3510791	76.728612	17.0962473	80.8232417	-1.8253987	-0.8682116	0.04143538	291	SCO2 cytochrome c oxidase assembly protein [Source:HGNC Symbol;Acc:10604]	SCO2	9997
ENSG00000187024	3.844054	6.83976528	2.95599745	4.43808464	-1.8547744	-0.8912438	0.04979294	294	peptidyl-tRNA hydrolase 1 homolog (S. cerevisiae) [Source:HGNC Symbol;Acc:27039]	PTRH1	138428

ENSG00000176108	16.1741738	9.06256339	6.53181528	2.88756452	1.74691446	0.80480896	0.01228386	298	charged multivesicular body protein 6 [Source:HGNC Symbol;Acc:25675]	CHMP6	79643
ENSG00000101452	7.83197896	4.59134525	2.17541847	1.49379596	1.75875864	0.81455751	0.03979398	338	DEAH (Asp-Glu-Ala-His) box polypeptide 35 [Source:HGNC Symbol;Acc:15861]	DHX35	60625
ENSG00000123545	11.9992578	6.30838387	7.63224924	2.55848033	1.76469599	0.81941967	0.04272141	339	NADH dehydrogenase (ubiquinone) complex I, assembly factor 4 [Source:HGNC Symbol;Acc:21034]	NDUFAF4	29078
ENSG00000164308	40.821823	71.0121169	23.345177	38.7380981	-1.7431308	-0.8016809	0.04460786	370	endoplasmic reticulum aminopeptidase 2 [Source:HGNC Symbol;Acc:29499]	ERAP2	64167
ENSG00000215788	31.1458551	20.189336	8.5399138	11.825102	1.68230191	0.75043664	0.02859737	383	tumor necrosis factor receptor superfamily, member 25 [Source:HGNC Symbol;Acc:11910]	TNFRSF25	8718
ENSG00000166928	9.75975384	15.9178026	5.83443496	8.22836833	-1.7204669	-0.7828001	0.04597851	385	membrane-spanning 4-domains, subfamily A, member 14 [Source:HGNC Symbol;Acc:30706]	MS4A14	84689
ENSG00000100983	20.1131047	12.1669817	8.35120165	5.06216689	1.67469497	0.74389834	0.04754107	422	glutathione synthetase [Source:HGNC Symbol;Acc:4624]	GSS	2937
ENSG00000088387	16.8628231	11.0698398	6.09546471	5.73354106	1.65041194	0.72282617	0.03979	428	dedicator of cytokinesis 9 [Source:HGNC Symbol;Acc:14132]	DOCK9	23348
ENSG00000086189	13.2958542	9.03412055	4.4048419	4.48016374	1.63476894	0.70908674	0.02931912	430	DIM1 dimethyladenosine transferase 1 homolog (S. cerevisiae) [Source:HGNC Symbol;Acc:30217]	DIMT1	27292
ENSG00000168016	52.0405413	88.5124301	16.4259479	34.0078162	-1.6337282	-0.708168	0.03344118	437	tetratricopeptide repeat and ankyrin repeat containing 1 [Source:HGNC Symbol;Acc:29011]	TRANK1	9881
ENSG00000152240	23.70219	15.1832388	8.14315435	6.17207669	1.64691461	0.71976575	0.04249685	439	HAUS augmin-like complex, subunit 1 [Source:HGNC Symbol;Acc:25174]	HAUS1	115106
ENSG00000114388	21.0902173	13.509506	8.6499024	5.15186934	1.58649408	0.66584214	0.01806102	461	nitrogen permease regulator-like 2 (S. cerevisiae) [Source:HGNC Symbol;Acc:24969]	NPRL2	10641
ENSG00000103064	47.2853698	31.5582443	14.7498967	14.0410219	1.58206682	0.66181054	0.02661458	485	solute carrier family 7 (amino acid transporter light chain, y+L system), member 6 [Source:HGNC Symbol;Acc:11064]	SLC7A6	9057

ENSG00000108395	27.0008574	18.1357182	5.86989177	4.2738317	1.55317385	0.63521932	0.02531088	516	tripartite motif containing 37 [Source:HGNC Symbol;Acc:7523]	TRIM37	4591
ENSG00000112144	25.7189117	17.380292	5.42733642	3.99647775	1.5517923	0.63393547	0.02970916	529	intestinal cell (MAK-like) kinase [Source:HGNC Symbol;Acc:21219]	ICK	22858
ENSG00000179152	7.71044964	13.0265346	1.90825586	5.0155261	-1.5522953	-0.634403	0.04778723	557	T cell activation inhibitor, mitochondrial [Source:HGNC Symbol;Acc:25241]	TCAIM	285343

Compared populations:

D2T RA High inflammation high pain vs D2T RA High inflammation low pain (sex-adjusted)

Fold change describes the size of the difference in gene expression (given in mean and standard deviation, sd) between the compared groups. The values are always in relation to the group used as a base level group (reference). P-value describes the reliability of the change in expression value between the compared groups. Results were considered significant with a fold change of 1.2 and a p-value cutoff of 0.05.

Supplementary Material 27 - List of differentially expressed (DE) genes (sex-adjusted)

ID	mean_LowInflammation_HighPain	mean_LowInflammation_LowPain	sd_LowInflammation_HighPain	sd_LowInflammation_LowPain	FC	logFC	P.Value	avgRank	Description	geneName	EntrezID
ENSG00000183473	7.18214017	2.26355157	3.99133242	1.59951034	4.05877495	2.02104435	0.00034964	1	somatostatin receptor 3 [Source:HGNC Symbol;Acc:11332]	SSTR3	6753
ENSG00000143942	2.93841149	1.28562964	1.90508594	1.15977242	3.22240226	1.6881366	0.00230941	2	ChaC, cation transport regulator homolog 2 (E. coli) [Source:HGNC Symbol;Acc:32363]	CHAC2	494143
ENSG00000163814	1.03848422	2.42923709	1.37309068	1.52723823	-3.5054738	-1.8096095	0.006062	3	CUB domain containing protein 1 [Source:HGNC Symbol;Acc:24357]	CDCP1	64866
ENSG00000162383	0.39865781	2.38826789	0.82148837	2.34822457	-4.3140792	-2.1090527	0.01170796	4	solute carrier family 1 (glutamate transporter), member 7 [Source:HGNC Symbol;Acc:10945]	SLC1A7	6512
ENSG00000223609	1.50879397	9.57225426	1.91055864	11.139186	-6.5801096	-2.7181116	0.01337761	5	hemoglobin, delta [Source:HGNC Symbol;Acc:4829]	HBD	3045
ENSG00000143416	0.49716695	2.50667581	0.58944926	2.15429965	-3.8570868	-1.9475116	0.01585601	6	selenium binding protein 1 [Source:HGNC Symbol;Acc:10719]	SELENBP1	8991
ENSG00000168661	3.58012146	0.78723305	4.24765936	0.69349172	3.1552436	1.65775139	0.01476597	7	zinc finger protein 30 [Source:HGNC Symbol;Acc:13090]	ZNF30	90075
ENSG00000213213	0.93707798	2.77477579	0.47051521	1.59410026	-2.6415877	-1.4014053	0.00522756	8	coiled-coil domain containing 183 [Source:HGNC Symbol;Acc:28236]	CCDC183	84960
ENSG00000137310	2.10860454	3.82750318	2.13658301	2.01878987	-2.7138263	-1.4403284	0.00979483	9	transcription factor 19 [Source:HGNC Symbol;Acc:11629]	TCF19	6941
ENSG00000100908	2.19523986	1.0154091	1.14200559	0.80038253	2.66791479	1.41571259	0.00704576	10	ER membrane protein complex subunit 9 [Source:HGNC Symbol;Acc:20273]	EMC9	51016
ENSG00000225614	1.28257572	2.53280295	1.45127623	1.72256276	-2.7140373	-1.4404405	0.01042561	11	zinc finger protein 469 [Source:HGNC Symbol;Acc:23216]	ZNF469	84627
ENSG00000182534	0.75615016	2.56631089	0.88047399	1.9543452	-3.089201	-1.6272337	0.0232069	12	matrix-remodelling associated 7 [Source:HGNC Symbol;Acc:7541]	MXRA7	439921

ENSG00000187474	1.1629224	3.09810479	1.10485065	1.98461809	-3.1587651	-1.6593607	0.02597751	13	formyl peptide receptor 3 [Source:HGNC Symbol;Acc:3828]	FPR3	2359
ENSG00000117479	0.76728534	2.26682122	1.01273607	2.271306	-2.9890694	-1.5796964	0.02283908	14	solute carrier family 19 (thiamine transporter), member 2 [Source:HGNC Symbol;Acc:10938]	SLC19A2	10560
ENSG00000142156	2.01562598	1.11855167	1.90158244	1.89308162	2.83997364	1.50587754	0.02257096	15	collagen, type VI, alpha 1 [Source:HGNC Symbol;Acc:2211]	COL6A1	1291
ENSG00000165895	3.05373228	1.41964412	1.87294061	1.01716251	2.69046702	1.42785662	0.01737704	16	Rho GTPase activating protein 42 [Source:HGNC Symbol;Acc:26545]	ARHGAP42	143872
ENSG00000109099	0.60225763	1.70245182	0.98171942	1.65828933	-3.215137	-1.6848802	0.03355268	17	peripheral myelin protein 22 [Source:HGNC Symbol;Acc:9118]	PMP22	5376
ENSG00000164171	1.54126136	3.27946624	1.76917954	3.19256982	-2.8320333	-1.5018382	0.025531	18	integrin, alpha 2 (CD49B, alpha 2 subunit of VLA-2 receptor) [Source:HGNC Symbol;Acc:6137]	ITGA2	3673
ENSG00000111249	0.752985	1.82480312	0.90894893	1.1228518	-2.8642844	-1.5181747	0.02734063	19	cut-like homeobox 2 [Source:HGNC Symbol;Acc:19347]	CUX2	23316
ENSG00000167840	1.54238189	3.15155895	1.27268866	1.96696304	-2.4351259	-1.2839964	0.00558053	20	zinc finger protein 232 [Source:HGNC Symbol;Acc:13026]	ZNF232	7775
ENSG00000101977	1.75120064	3.81879717	1.4587208	1.68603143	-2.5894196	-1.3726288	0.02124063	21	MCF.2 cell line derived transforming sequence [Source:HGNC Symbol;Acc:6940]	MCF2	4168
ENSG00000139197	2.81129064	1.39570435	1.23056991	0.82908443	2.52539218	1.33650745	0.01599919	22	peroxisomal biogenesis factor 5 [Source:HGNC Symbol;Acc:9719]	PEX5	5830
ENSG00000066735	3.66073498	1.93697297	2.72924569	1.87219773	2.77916605	1.47465204	0.03089539	23	kinesin family member 26A [Source:HGNC Symbol;Acc:20226]	KIF26A	26153
ENSG00000138180	1.22689988	2.08971738	2.52612298	1.91736212	-2.7797689	-1.4749649	0.03600723	24	centrosomal protein 55kDa [Source:HGNC Symbol;Acc:1161]	CEP55	55165
ENSG00000171860	8.97894078	20.6437222	6.2698237	12.1450097	-2.3360586	-1.2240765	0.01038282	25	complement component 3a receptor	C3AR1	719

									1 [Source:HGNC Symbol;Acc:1319]		
ENSG00000142621	0.59111459	2.41499764	0.81500459	3.00033668	-2.9399359	-1.5557847	0.046088	26	forkhead-associated (FHA) phosphopeptide binding domain 1 [Source:HGNC Symbol;Acc:29408]	FHAD1	114827
ENSG00000189127	4.45788658	2.17528632	3.00618477	1.8992722	2.56204366	1.35729506	0.03199197	27	ankyrin repeat domain 34B [Source:HGNC Symbol;Acc:33736]	ANKRD34B	340120
ENSG00000170955	0.64560029	1.47005288	1.23384868	1.15215375	-3.0849502	-1.6252472	0.04852983	28	protein kinase C, delta binding protein [Source:HGNC Symbol;Acc:9400]	PRKCDBP	112464
ENSG00000213077	3.40382547	1.90300487	2.60397483	2.19086622	2.94821219	1.55984036	0.04784653	29	family with sequence similarity 106, member A [Source:HGNC Symbol;Acc:25682]	FAM106A	
ENSG00000143847	2.93631874	1.41023714	1.73127031	1.19106369	2.50812075	1.32660681	0.02932425	30	protein tyrosine phosphatase, receptor type, f polypeptide (PTPRF), interacting protein (liprin), alpha 4 [Source:HGNC Symbol;Acc:9248]	PPFIA4	8497
ENSG00000074590	1.45007373	3.69237841	1.40386117	2.28488388	-2.5775183	-1.3659827	0.03726645	31	NUAK family, SNF1-like kinase, 1 [Source:HGNC Symbol;Acc:14311]	NUAK1	9891
ENSG00000055732	3.59014366	1.77779684	2.08518716	1.60718869	2.32537601	1.21746401	0.02178171	32	mucolipin 3 [Source:HGNC Symbol;Acc:13358]	MCOLN3	55283
ENSG00000178222	1.65740697	0.68281492	1.27681256	0.60643891	2.47329143	1.30643224	0.03358235	33	ring finger protein 212 [Source:HGNC Symbol;Acc:27729]	RNF212	285498
ENSG00000170962	2.17571986	4.26122268	2.58488677	1.98154663	-2.5364427	-1.3428066	0.0418183	34	platelet derived growth factor D [Source:HGNC Symbol;Acc:30620]	PDGFD	80310
ENSG00000186827	7.12668535	3.7799334	2.86445349	2.79413437	2.20060145	1.13789788	0.00975883	35	tumor necrosis factor receptor superfamily, member 4 [Source:HGNC Symbol;Acc:11918]	TNFRSF4	7293
ENSG00000196741	1.94181633	0.96395752	1.57827379	0.94443704	2.49200394	1.31730635	0.03850218	36	chromosome X open reading frame 24 [Source:HGNC Symbol;Acc:27333]	CXorf24	

ENSG00000158186	2.5300555	5.206679	1.57011536	2.31836022	-2.2723783	-1.184203	0.01954937	37	muscle RAS oncogene homolog [Source:HGNC Symbol;Acc:7227]	MRAS	22808
ENSG00000072195	1.77575496	0.75199889	1.33725577	0.79568546	2.41004729	1.26906146	0.03398867	38	SPEG complex locus [Source:HGNC Symbol;Acc:16901]	SPEG	100996693
ENSG00000092208	4.96018001	2.54682568	2.10647006	1.67220562	2.05596469	1.03981549	0.00949854	44	gem (nuclear organelle) associated protein 2 [Source:HGNC Symbol;Acc:10884]	GEMIN2	8487
ENSG00000175352	2.8361202	1.52396602	2.95665347	1.90686651	2.31956048	1.21385146	0.04549651	49	nuclear receptor interacting protein 3 [Source:HGNC Symbol;Acc:1167]	NRIP3	56675
ENSG00000129422	7.67895537	2.81101523	7.66720804	1.97036787	2.33685319	1.2245671	0.04848861	50	microtubule associated tumor suppressor 1 [Source:HGNC Symbol;Acc:29789]	MTUS1	57509
ENSG00000128268	4.34410698	2.61813863	2.33248782	2.36420044	2.127227	1.08897399	0.02499889	51	mannosyl (beta-1,4-)-glycoprotein beta-1,4-N-acetylglucosaminyltransferase [Source:HGNC Symbol;Acc:7046]	MGAT3	4248
ENSG00000064199	0.93482894	1.97312853	0.8452897	1.3012227	-2.3393234	-1.2260913	0.04886831	52	sperm autoantigenic protein 17 [Source:HGNC Symbol;Acc:11210]	SPA17	53340
ENSG00000076351	1.24197546	2.60028092	1.27363693	1.64214254	-2.3295984	-1.2200813	0.04859185	54	solute carrier family 46 (folate transporter), member 1 [Source:HGNC Symbol;Acc:30521]	SLC46A1	113235
ENSG00000183742	2.33077181	1.37881041	1.37170769	1.19972784	2.22667068	1.1548882	0.04207199	55	metastasis associated in colon cancer 1 [Source:HGNC Symbol;Acc:30215]	MACC1	346389
ENSG00000163449	5.62296962	3.46598547	4.24271843	3.01678272	2.07263506	1.05146611	0.02405562	56	transmembrane protein 169 [Source:HGNC Symbol;Acc:25130]	TMEM169	92691
ENSG00000198924	2.20382408	4.12400811	1.65481636	2.21995524	-2.1870927	-1.1290143	0.04373645	58	DNA cross-link repair 1A [Source:HGNC Symbol;Acc:17660]	DCLRE1A	9937
ENSG00000077585	3.45485303	7.07720937	1.56941331	3.05673332	-1.9700164	-0.9782076	0.00711453	59	G protein-coupled receptor 137B [Source:HGNC Symbol;Acc:11862]	GPR137B	7107

ENSG00000173926	4.34743164	2.73164679	1.91228628	2.29506087	2.13590923	1.09485034	0.04666345	62	membrane-associated ring finger (C3HC4) 3, E3 ubiquitin protein ligase [Source:HGNC Symbol;Acc:28728]	MARCH3	115123
ENSG00000148468	5.69119895	3.31571315	2.10850392	2.1941152	2.09228814	1.06508155	0.04432528	65	family with sequence similarity 171, member A1 [Source:HGNC Symbol;Acc:23522]	FAM171A1	221061
ENSG00000156172	2.06110983	3.44405366	1.87223172	2.06419948	-2.069159	-1.0490445	0.04250185	66	chromosome 8 open reading frame 37 [Source:HGNC Symbol;Acc:27232]	C8orf37	157657
ENSG00000184271	7.99588108	4.67554501	3.75203551	2.89382407	2.0367203	1.02624787	0.03629641	67	POU class 6 homeobox 1 [Source:HGNC Symbol;Acc:9224]	POU6F1	5463
ENSG00000100079	26.3526702	49.7344121	17.972887	31.1589292	-2.0386183	-1.0275917	0.04726163	73	lectin, galactoside-binding, soluble, 2 [Source:HGNC Symbol;Acc:6562]	LGALS2	3957
ENSG00000107281	11.1689818	6.39794603	5.61490821	3.86125705	1.93777124	0.95439826	0.01671493	75	neural proliferation, differentiation and control, 1 [Source:HGNC Symbol;Acc:7899]	NPDC1	56654
ENSG00000249992	13.0656575	20.915501	12.3218781	13.448537	-1.9950944	-0.996457	0.04063095	77	transmembrane protein 158 (gene/pseudogene) [Source:HGNC Symbol;Acc:30293]	TMEM158	25907
ENSG00000141837	8.35442207	4.96222323	4.58982144	3.64564724	1.94889128	0.96265361	0.0328377	82	calcium channel, voltage-dependent, P/Q type, alpha 1A subunit [Source:HGNC Symbol;Acc:1388]	CACNA1A	100507353
ENSG00000157827	8.10275768	15.4529436	5.82595332	8.99535962	-1.9562664	-0.9681029	0.03568497	83	formin-like 2 [Source:HGNC Symbol;Acc:18267]	FMNL2	114793
ENSG00000196812	2.73444919	4.86651082	1.35979527	1.56195329	-1.9514657	-0.9645581	0.0405462	86	zinc finger and SCAN domain containing 16 [Source:HGNC Symbol;Acc:20813]	ZSCAN16	80345
ENSG00000213949	9.04970049	5.23506206	5.15988692	2.92146043	1.90416511	0.92915858	0.02245391	88	integrin, alpha 1 [Source:HGNC Symbol;Acc:6134]	ITGA1	3672
ENSG00000108439	2.56349167	5.32798355	1.63920725	2.84048194	-1.94292	-0.9582265	0.04451122	91	pyridoxamine 5'-phosphate oxidase [Source:HGNC Symbol;Acc:30260]	PNPO	55163

ENSG00000103512	4.54890145	7.64375313	2.94120664	2.11282036	-1.8891112	-0.9177076	0.02219003	93	NODAL modulator 1 [Source:HGNC Symbol;Acc:30060]	NOMO1	101930025
ENSG00000084731	3.90569763	2.67850622	1.65278277	2.28614321	1.88663279	0.91581364	0.04425229	102	kinesin family member 3C [Source:HGNC Symbol;Acc:6321]	KIF3C	3797
ENSG00000107736	8.40892194	15.496201	3.89383965	6.59221605	-1.7923871	-0.8418822	0.01298952	109	cadherin-related 23 [Source:HGNC Symbol;Acc:13733]	CDH23	100653137
ENSG00000174600	17.9793793	32.3888603	10.7900059	13.266595	-1.7732454	-0.8263922	0.01440201	118	chemokine-like receptor 1 [Source:HGNC Symbol;Acc:2121]	CMKLR1	1240
ENSG00000066926	6.19876909	12.1897153	1.94518829	6.78856618	-1.7777967	-0.8300903	0.01838233	119	ferrochelatase [Source:HGNC Symbol;Acc:3647]	FECH	2235
ENSG00000137441	45.1366044	88.1569811	27.7847651	45.6484899	-1.7821206	-0.833595	0.02714206	122	fibroblast growth factor binding protein 2 [Source:HGNC Symbol;Acc:29451]	FGFBP2	83888
ENSG00000182010	63.5471459	35.4987996	38.4727552	15.5728957	1.71797344	0.78070773	0.02742257	152	rhotekin 2 [Source:HGNC Symbol;Acc:19364]	RTKN2	219790
ENSG00000103043	3.24725174	6.07611948	1.49738269	2.37441536	-1.7514662	-0.8085631	0.04760084	153	Vac14 homolog (S. cerevisiae) [Source:HGNC Symbol;Acc:25507]	VAC14	55697
ENSG00000103353	6.92354281	11.2083962	3.41832747	2.56687339	-1.6437178	-0.7169627	0.03472065	196	ubiquitin family domain containing 1 [Source:HGNC Symbol;Acc:30565]	UBFD1	56061
ENSG00000184634	19.3981388	14.1436355	3.69272631	7.67646064	1.58481973	0.66431875	0.02853776	231	mediator complex subunit 12 [Source:HGNC Symbol;Acc:11957]	MED12	9968
ENSG00000121413	20.8089577	14.1399296	9.97563139	8.60496421	1.57995351	0.65988211	0.04607054	244	zinc finger and SCAN domain containing 18 [Source:HGNC Symbol;Acc:21037]	ZSCAN18	65982
ENSG00000170448	30.795083	18.7989437	20.9132529	7.70734526	1.57338699	0.65387356	0.04751917	252	nuclear transcription factor, X-box binding-like 1 [Source:HGNC Symbol;Acc:18726]	NFXL1	152518
ENSG00000088038	16.0973434	11.4522643	3.44322006	4.09572837	1.55081252	0.63302429	0.0361203	260	CCR4-NOT transcription complex, subunit 3 [Source:HGNC Symbol;Acc:7879]	CNOT3	4849

Compared populations:

D2T RA Low inflammation high pain vs non-D2T RA (sex-adjusted)

Fold change describes the size of the difference in gene expression (given in mean and standar deviation, sd) between the compared groups. The values are always in relation to the group used as a base level group (reference). P-value describes the reliability of the change in expression value between the compared groups. Results were considered significant with a fold change of 1.2 and a p-value cutoff of 0.05.
